# Temporal and Sex-Linked Protein Expression Dynamics in a Familial Model of Alzheimer’s Disease

**DOI:** 10.1016/j.mcpro.2022.100280

**Published:** 2022-08-06

**Authors:** Filipa Blasco Tavares Pereira Lopes, Daniela Schlatzer, Rihua Wang, Xiaolin Li, Emily Feng, Mehmet Koyutürk, Xin Qi, Mark R. Chance

**Affiliations:** 1 Department of Nutrition, School of Medicine, Case Western Reserve University, Cleveland, Ohio, USA; 2 Center for Proteomics and Bioinformatics, School of Medicine, Case Western Reserve University, Cleveland, Ohio, USA; 3 Department of Physiology & Biophysics, School of Medicine, Case Western Reserve University, Cleveland, Ohio, USA; 4 Center for Mitochondrial Diseases, School of Medicine, Case Western Reserve University, Cleveland, Ohio, USA; 5 Department of Computer and Data Sciences, Case School of Engineering, Case Western Reserve University, Cleveland, Ohio, USA

**Keywords:** Alzheimer's Disease, 5XFAD, Proteome, Temporal dynamics, Sex differences, Pathway analysis, Aß, amyloid-beta, ACLY, ATP-citrate synthase, AD, Alzheimer’s disease, APP, Aβ precursor protein, GO, gene ontology, PRM, parallel reaction monitoring, ROS, reactive oxygen species

## Abstract

Mouse models of Alzheimer’s disease (AD) show progression through stages reflective of human pathology. Proteomics identification of temporal and sex-linked factors driving AD-related pathways can be used to dissect initiating and propagating events of AD stages to develop biomarkers or design interventions. In the present study, we conducted label-free proteome measurements of mouse hippocampus tissue with variables of time (3, 6, and 9 months), genetic background (5XFAD *versus* WT), and sex (equal males and females). These time points are associated with well-defined phenotypes with respect to the following: Aβ42 plaque deposition, memory deficits, and neuronal loss, allowing correlation of proteome-based molecular signatures with the mouse model stages. Our data show 5XFAD mice exhibit increases in known human AD biomarkers as amyloid-beta peptide, APOE, GFAP, and ITM2B are upregulated across all time points/stages. At the same time, 23 proteins are here newly associated with Alzheimer’s pathology as they are also dysregulated in 5XFAD mice. At a pathways level, the 5XFAD-specific upregulated proteins are significantly enriched for DNA damage and stress-induced senescence at 3-month only, while at 6-month, the AD-specific proteome signature is altered and significantly enriched for membrane trafficking and vesicle-mediated transport protein annotations. By 9-month, AD-specific dysregulation is also characterized by significant neuroinflammation with innate immune system, platelet activation, and hyper-reactive astrocyte–related enrichments. Aside from these temporal changes, analysis of sex-linked differences in proteome signatures uncovered novel sex and AD-associated proteins. Pathway analysis revealed sex-linked differences in the 5XFAD model to be involved in the regulation of well-known human AD-related processes of amyloid fibril formation, wound healing, lysosome biogenesis, and DNA damage. Verification of the discovery results by Western blot and parallel reaction monitoring confirm the fundamental conclusions of the study and poise the 5XFAD model for further use as a molecular tool for understanding AD.

## Highlights

• 5XFAD mice have a dramatic protein expression increase overtime *versus* WT.
• At 3 months, DNA damage and cellular senescence dominate AD proteomic signature.
• Starting at 6 months, neuro-inflammation signatures further sustain AD.
• 5XFAD mice replicate sex-specific proteomic changes characteristic of human AD.

## Graphical Abstract

**Figure:**
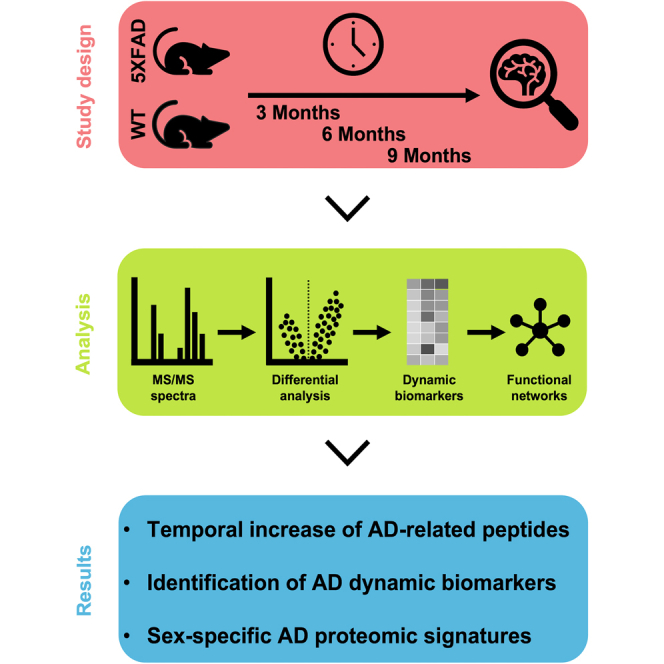

## In Brief

Alzheimer’s disease still lacks effective therapies. In this study, we provide a longitudinal proteomic characterization of 5XFAD mice hippocampus hoping to identify new clinical intervention targets. Our proteomics approach reveals a striking increase in protein expression in 5XFAD mice overtime, which includes 23 proteins here newly associated with Alzheimer’s disease. Globally, our data reveals initial temporal signatures of DNA damage and senescence transitioning into neuroinflammatory signatures concurrent with disease progression.

Alzheimer’s disease (AD) is a leading cause of dementia and has long been characterized by its heterogeneity and complexity regarding risk factors, progression, and response to treatment (1, 2, 3, 4). Further challenges to the field include that AD is traditionally diagnosed by histopathological amyloid-beta (Aβ) examination postmortem, but abnormal Aβ deposition precedes neurodegeneration and cognitive decline (5, 6). A molecular understanding of the protein-mediated mechanisms that lead to abnormal Aβ deposition, as well as the arrangement and timing of the initiating and propagating pathways, could provide clues to biomarkers and intervention points to predict or control progression of AD in individual patents (7).

AD progression is clearly regulated at all DNA, RNA, and protein levels. The complexity of AD regulation is illustrated by transcriptomic and proteomic crossover studies that report mRNA to protein correlations of 0.45 (8). Proteome-wide studies to date have primarily focused on postmortem brain samples, which are limited for describing progressive molecular changes in AD (2, 3). Mouse models are highly suited to examining changes in temporal features of AD progression. For example, a longitudinal study that probed hippocampus perfusates of a gold standard amyloid-driven AD model (5XFAD mice) showed glucose and lipid metabolism dysregulation prior to AD pathogenesis, providing a proof of concept for distinguishing early from late events in the molecular pathology of progression (9). The present research also utilizes 5XFAD mice to better understand the temporal variation in protein expression in mouse brain for WT compared to the AD-like mouse model at various time points relevant to disease progression hallmarks in humans. 5XFAD mice coexpress five familial AD mutations (amyloid precursor protein and presenilin 1 genes) that cause accumulation of amyloidogenic Aβ_42_ that drives a set of pathogenic changes, many of which mimic features of human AD. For example, 5XFAD mice show a dramatic increase in Aβ_42_, which is reflected by an early onset of plaque deposition (around 2 months of age) that spreads to cover most of the brain in parallel with astrocytosis and microgliosis (around 4 months of age). The mice later develop neuron loss around 9 months of age at the cortex and subiculum. A caveat of the 5XFAD mouse model with respect to known human pathology is that the Aβ plaques trigger neuritic tau plaque aggregates instead of neurofibrillary tangles (an essential neuropathological AD hallmark (10, 11)). Nevertheless, the similarities to human pathology induced us to design a temporal and sex-linked study to provide novel insights into known biomarkers as well as potentially identify novel AD targets. We coupled the discovery studies with validation studies using Western blots and peptide parallel reaction monitoring. Overall, our results confirm temporal and sex-linked variation for known and novel AD biomarkers in hippocampus and cortex tissue.

## Experimental Procedures

### Experimental Design and Statistical Rationale

The experimental design included hippocampus tissue collection from 16 mice at each of 3 months, 6 months, and 9 months. Each time point featured eight WT C57BL/6 mice and eight 5XFAD mice (four male and four female biological replicates). All samples were randomized for processing and data acquisition. In the 3 months dataset, a principal component analysis assessment identified two potential outliers (none were detected for the 6- or 9-month samples). A Levene test was performed to assess the equality of variance within-group. The test was significant (*p*-value = 2.2 × 10^−16^). A leave-one-out strategy was used to identify the drivers of this variation. Sample 3156 was identified and removed from the analysis (all other samples were utilized). An unpaired *t* test, with individual variances computed for each comparison test was performed between WT and 5XFAD groups using GraphPad Prism version 9 (GraphPad Software). Peptides with a *p*-values ≤0.1 were initially selected and then a differentially expressed peptide set was further downselected based on 5XFAD/WT 0.5 ≥ fold change ≥ 2 criteria.

### 5XFAD AD Transgenic Mice

All animal experiments were conducted in accordance with protocols approved by the Institutional Animal Care and Use Committee of Case Western Reserve University and performed according to the National Institutes of Health Guide for the Care and Use of Laboratory Animals. Sufficient procedures were employed to reduce the pain and discomfort of the mice during the experiments. The mice were mated, bred, and genotyped in the animal facility of Case Western Reserve University. All mice were maintained under a 12 h/12 h light/dark cycle (light on at 6 AM and off at 6 PM). All mice used in this study were maintained on a C57BL/6J background. 5XFAD transgenic mice [Tg(APPSwFlLon,PSEN1∗M146L∗L286V)6799Vas, JAX Stock No: 34840] breeders were purchased from Jackson Laboratory.

### Behavioral Analysis

All behavioral analyses were conducted by an experimenter who was blinded to the genotypes and treatment groups. All mice were subjected to a series of behavioral measurements to spontaneous spatial working memory (Y-maze test) and long-term spatial learning and memory functions (Barnes maze test).

#### Y-Maze Test

On the test day, mice (6 months old) were brought to the testing room 1 h before performing the Y-maze test to allow habituation. The mice were placed in the middle of the Y-maze and allowed to explore the three arms for 6 min. During exploration, the arm entries were recorded. The equipment was cleaned after every test to avoid odor disturbance. Spontaneous alternation was defined as a successive entry into three different arms on overlapping triplet sets.

#### Barnes Maze Test

On the test day, the mice were brought to the testing room 30 min before performing the Barnes maze test to allow habituation. Briefly, all the testing mice received three consecutive days of trials, with three trials each day. After being placed in the center of the platform at the beginning of each trial, the mice were allowed to explore for 3 min to find the target escape box. Mice that failed to enter the target escape hole in the given time were led to it by the operator. Mice were allowed to remain in the target hole for 2 min before returning to the home cage. After completing the 3-day trials, the mice were examined on day 5 and 12 with one test to monitor the long-term spatial learning and memory activities. The maze and the escape box were cleaned carefully after each trial to avoid odor disturbance. All the trials and tests were recorded with a video system. The total time to enter the target escape box (latency to the target box) and the number of times the wrong holes were explored (the total errors) were recorded.

### Immunofluorescence

Mice were deeply anesthetized and transcardially perfused with 4% paraformaldehyde in PBS. Brain sections were permeabilized with 0.2% Triton X-100 in TBS-T buffer, followed by blocking with 5% normal goat serum. The brain sections were incubated with the indicated primary antibodies (anti-6E10, 1:5000 dilution) overnight at 4 °C and then stained with secondary antibodies. Images of the staining were acquired using a Fluoview FV 1000 confocal microscope (Olympus). All quantification of immunostaining was performed using ImageJ software (https://imagej.nih.gov/ij/index.html). The same image exposure times and threshold settings were used for all sections from all the experimental groups. Quantitation was performed blinded to the experimental groups.

### Label-Free Quantitative Proteomic

Hippocampus tissue samples from 5XFAD and WT mice at different time points (3, 6, and 9 months) were isolated and lysed with a 2% SDS solution supplemented with protease inhibitor cocktail (Cat#P2714, Sigma) and PhosphoSTOP (Cat#04906837001, Roche). Cell lysates had their protein concentration determined by using Bio-Rad Protein Assay Kit – BSA (Cat#5000002, Bio-Rad). Lysates were processed by filter-aided sample preparation as per previous procedures (1), digested using dual LysC (Cat#125-05061, Wako) and trypsin (Cat# 90057, Thermo Fisher) endoproteinase and desalted using C18 cartridges. Samples were normalized to 300 ng of digest, blinded, and randomized for LC-MS/MS acquisition by Waters NanoAcquity UPLC chromatography system coupled to a Thermo Scientific Orbitrap Elite mass spectrometer (Thermo Fisher Scientific). Raw LC-MS/MS data was processed using Peaks v10.0 Software (Bioinformatics Solutions) as described (12, 13). Peptide identification was performed within Peaks using UNIPROT database (UNIPROT_MOUSE_091219, # entries= 17,026). PEAKS search parameters were set to determine the following: mass error tolerance for precursor ions of 10 ppm, mass tolerance for fragment ions of 0.6 Da, trypsin enzyme specificity and included carbamidomethylation as a fixed variation plus methionine oxidation as a variable modification, and one missed cleavage. Label-free peptide identification was performed using default target decoy approach, which included PEAKS peptide score (−10logP) ≥15 and FDR threshold of 1%. Individual peptide abundance was determined by area under the curve (supplemental Materials).

### Parallel Reaction Monitoring

#### Sample Preparation for PRM Assay

Three peptides (supplemental Table S1) were selected for monitoring three proteins (tier 2) based on our discovery study. Supplemental Table S1 highlights peptides that were verified for selected proteins by this assay. The stable-isotope–labeled peptide (C-terminal arginine or lysine residue labeled with 13C and 15N) was synthesized (Thermo Fisher Scientific, AQUA Basic-grade, >95% purity) and used as the standard for confirmation and quantitation (supplemental Figs. S5–S7). A mixture with all heavy-labeled peptides was spiked into the digest to yield a final concentration of 16.67 fmol/μl for each standard peptide. A total of 32 hippocampus and 48 cortex digest samples from 5XFAD and WT mice at different time points (3, 6, and 9 months) were analyzed using parallel reaction monitoring (PRM) method.

#### Development and Analytical Validation Targeted MS Assays/Measurements

Five hundred nanograms total protein of each digest containing 50 fmol of each standard peptide were analyzed by LC/MS using a Waters NanoACQUITY Ultra performance liquid chromatography system (Waters) and an Orbitrap Exploris 480 mass spectrometer (Thermo). The platform was operated in the positive nano-LC mode using the standard nano-electrospray atmospheric pressure ionization stack fitted with a PicoTip emitter (uncoated fitting, 10 μm spray orifice; New Objective). The digests were first desalted on a reversed-phase C18 trapping column (ACQUITY UPLC Symmetry C18 NanoACQUITY 2G-V/MTrap column, 5 μm, 180 μm × 20 mm, Waters) by washing with 0.1% formic acid at 10 μl/min for 4 min. Then the trapping column was switched in-line with a reversed-phase C18 column (ACQUITY UPLC Peptide BEH C18 NanoACQUITY column, 1.7 μm, 75 μm × 250 mm, Waters), and peptides were separated from other endogenous components first using a linear gradient of acetonitrile with 0.1% formic acid from 1% to 5% in 1 min followed by another linear gradient from 5% to 30% over a period of 29 min at a flow rate of 0.35 μl/min. PRM experiment was employed to detect both the isotopically labeled standard and the native peptide. The PRM approach was accomplished by specifying the parent mass of each peptide to be quantified for MS/MS fragmentation and then selectively monitoring for its fragment ions (supplemental Table S2). This technique is very sensitive and can typically quantify peptides at concentrations of fmol/μl. This is illustrated by the quantification ranges and linearity in supplemental Table S3. The acquired data is processed and analyzed using Xcalibur 4.1.31.9 Quan Browser software (Thermo Fisher Scientific). Each endogenous peptide was confirmed by comparing its retention time, the top three abundant product ion masses, and their fragmentation patterns to the synthesized peptide. The ratio of endogenous to synthetic peptide signal intensity which was the summed peak area of the top three product ions was used for quantification.

### Western Blot

Hippocampus and cortex lysates were normalized to 30 μg of protein and separated by SDS-PAGE using NuPage 4 to 12% Bis-Tris gel (Cat#NP0321BOX, ThermoFischer). Protein transfer to polyvinylidene difluoride membranes (Cat#1704156, Bio-Rad) was performed by using the Trans-Blot Transfer system (Cat#1704150, Bio-Rad). Polyvinylidene difluoride membranes were blocked with 3% bovine serum albumin in TBS containing 0.1% Tween-20 (Cat#37520, Cat#28360, ThermoFischer) for 1 h. The membranes were subsequently incubated for 90 min, with the respective primary antibodies: glial fibrillary acidic protein (1:7500 – Cat#16825-1-AP, Proteintech) and GAPDH (1:5000 - Cat#PA1-987, ThermoFischer). The membranes were then incubated for 30 min in horseradish peroxidase–conjugated secondary antibody (1:4000 - Cat#A27036, ThermoFischer). Membranes were developed for 1 min, using ECL Substrate (Cat#WP20005, Invitrogen), and chemiluminescence was captured and quantified by iBright CL1500 Imaging System (Cat#A44240, Invitrogen). Relative protein levels were normalized against the expression of GAPDH, which served as a loading control.

### Bioinformatic Analysis

#### DisGeNET

Gene-disease association data relative to Alzheimer’s disease (CUI: C0002395) was retrieved from DisGeNET v6.0 (http://www.disgenet.org/), Integrative Biomedical Informatics Group GRIB/IMIM/UPF. [April, 2022] (14).

#### Enrichment Pathway Analysis

A PANTHER Overrepresentation Test with Reactome pathways annotation (Fischer’s exact test and FDR correction FDR *p* < 0.05) was performed on highly upregulated peptides in AD mice (5XFAD/WT Log2FC ≥ 4 and *p* ≤ 0.1, unpaired *t* test) (15).

#### Functional Enrichment Analysis

We used the ClueGO v2.5.8 (16)/Cluepedia v1.5. (17) plugin from Cytoscape v3.8. (18) to visualize functionally grouped networks. We tested our sex-specific differentially upregulated peptides (5XFAD/WT 0.5 ≥ FC ≥ 2 and *p* ≤ 0.1, unpaired *t* test and subsequent 5XFAD male *versus* 5XFAD female *p* ≤ 0.05, unpaired *t* test) against the gene ontology (GO) and Reactome reference sets to yield enriched terms using a two-sided hypergeometric test with Bonferroni step-down correction and only showed enriched terms with a *p* ≤ 0.05. Furthermore, we defined network connectivity to a kappa score ≥0.4, GO tree levels ranging from 6 to 12 and utilized the GO term fusion feature to eliminate parent-child term redundancy.

## Results

We performed global proteome profiling of 5XFAD mice hippocampus across different time points reflecting hallmarks of AD. Mouse colonies were initiated for all experiments at one time, then 16 M/F animals were randomly selected for sacrifice and processing of hippocampus tissue at the 3-, 6-, and 9-month intervals, respectively. Label-free LC-MS was carried out on each set of 16 samples run contemporaneously without fractionation as the samples were collected during the study. Although an alternative study design would save all samples until the end and run them in blocks to better permit cross time point analysis, the decision was made instead to process samples quickly in a fresh state comparable at specific time points (no more than a few weeks after sacrifice) and focus the statistical analysis on WT *versus* AD followed by sex-linkage analysis at the three individual time points. This advantage of processing all samples from a time point shortly after sacrifice in batches was however balanced against difficulties in comparing individual groups across the time points, due to challenges in absolute standardization of these different batches across time points in the studies.

### Global Features of Protein Expression in 5XFAD Mouse Model Over Time

**Fig. 1:**
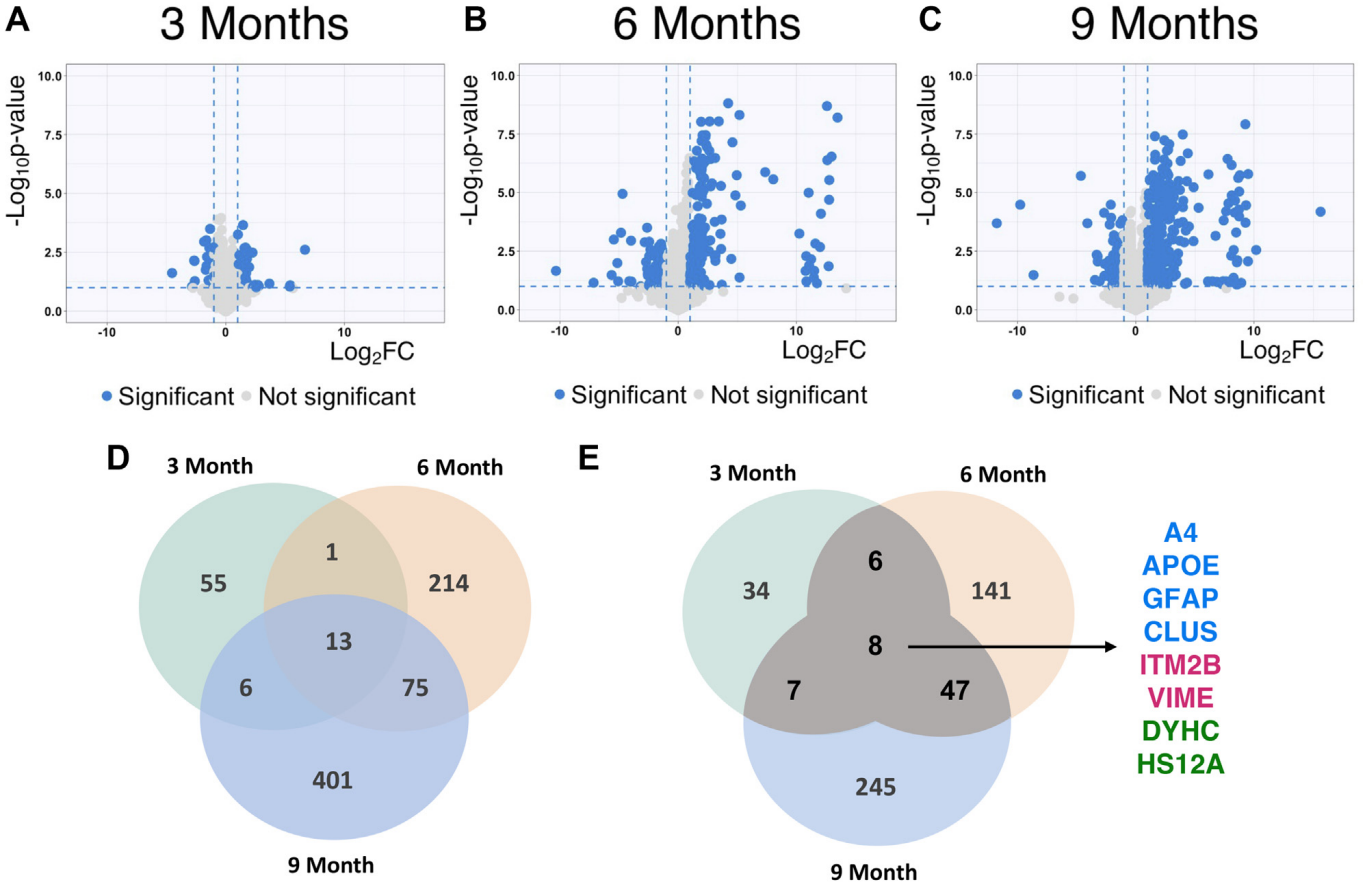
**Global protein expression patterns highlight substantial increase of significantly expressed peptides in AD.***A*–*C*, significantly expressed peptides in AD mice; volcano plot represents the proteome with significant peptides in *blue* (5XFAD/WT 0.5 ≥ fold change ≥ 2 and *p* ≤ 0.1, unpaired *t* test) (3 months, 6 months, and 9 months). *D*, the Venn diagram shows the number of shared peptides that are significantly expressed at all time-points (5XFAD/WT 0.5 ≥ fold change ≥ 2 and *p* ≤ 0.1, unpaired *t* test). *E*, the Venn diagram shows the number of proteins that are significantly expressed at all time-points (5XFAD/WT 0.5 ≥ fold change ≥ 2 and *p* ≤ 0.1, unpaired *t* test). AD, Alzheimer’s disease.

We identified 11,038 total peptides in the 3-month dataset, 14,362 peptides in the 6-month dataset, and 18,332 peptides in the 9-month dataset reaching a total hippocampal protein coverage of 2974 proteins (Fig. 1, *A*–*C*). Our coverage is comparable to other studies without fractionation that characterize the brain proteome (19). The volcano plots for each dataset highlight a dramatic accumulation of significantly differentially expressed peptides with progression of time (5XFAD/WT 0.5 ≥ FC ≥ 2 and *p* ≤ 0.1, depicted as blue dots) (Fig. 1, *A*–*C*). In the 3-month, 6-month, and 9-month time points, 52, 212, and 328 peptides were upregulated, respectively, while 23, 91, and 167 peptides were downregulated, respectively (Fig. 1, *A*–*D*). Thus, two-thirds of these differentially expressed peptides are upregulated in the 5XFAD mice (Fig. 1, *A*–*D*).

We also identify 13 peptides that are differentially expressed across all three time points (Fig. 1*E*), which annotate to eight different proteins including Aβ precursor protein (APP), apolipoprotein E, glial fibrillary acidic protein, clusterin, integral membrane protein 2B, vimentin, cytoplasmic dynein 1 heavy chain, and heat shock protein 70 kDa protein 12A (Fig. 1*E*). Upon comparison of the levels of regulation of these proteins over time, we can distinguish three patterns for these potential AD biomarker candidates. The first four are consistently upregulated and increase their upregulation with time including the following: Aβ precursor protein, apolipoprotein E, glial fibrillary acidic protein, and clusterin (highlighted in blue text, Fig. 1*E*). A second group is upregulated but increased their upregulation more gradually and included integral membrane protein 2B and vimentin (highlighted in purple). A third group has variable but significant decreases in expression and includes the following: cytoplasmic dynein 1 heavy chain and heat shock protein 70 KDa protein 12A (highlighted in green). The consistently upregulated protein group includes well-known AD biomarker candidates from the DisGeNET database (CUI: C0002395). Integral membrane protein 2B is another evidence-based DisGeNET AD biomarker, whereas vimentin is a known phenotypic marker of glial cells and endothelial cells. Alongside glial fibrillary acidic protein, vimentin doubles as a classical astrogliosis marker (20, 21), which in turn plays a central role in AD neuroinflammation (22, 23).

### Neuroinflammation Pathway Signatures Correlate With 5XFAD Stages of Progression

To further characterize the biological pathways that transiently drive AD, we performed separate enrichment pathway analysis for the peptides exclusively dysregulated at 3-, 6-, or 9-month time points. Indeed, we found different pathways to be overrepresented at different time points (supplemental Fig. S1). Only at the third month, DNA damage (*p*-value = 4.3e-2) and cellular senescence (*p*-value = 2.3e-2) are seen to be significantly dysregulated. At month-6 and onward, membrane (*p*-value = 3.2e-2) and vesicle-mediated transport (*p*-value = 3.3e-2) are significantly enriched, this significance is increased at 9-month (supplemental Fig. S1). Disruption in membrane and vesicle trafficking have long been associated with an increase in both amyloid precursor protein processing and Aβ production (24, 25). Thus, the increases in APP and Aβ protein levels by 6-month seen in supplemental Fig. S2 are consistent with these findings. Aβ aggregates continuously activate microglia and establish a neurotoxic environment; this chronic state of inflammation disrupts Aβ clearance, further exacerbating Aβ accumulation (25). Lastly, the innate immune system (*p*-value = 2.57e-7) and neutrophil degranulation pathways (*p*-value = 3.25e-6) are significantly enriched, but only by month-9 (supplemental Fig. S1). Both neutrophil recruitment and platelet activation are known immune modulators essential for setting the immune milieu in AD (22, 23). These data suggest that AD is under a complex and time-sensitive proteomic regulation, with dramatic shifts from early signatures of senescence and DNA damage to neuroinflammation signatures evident at sixth and ninth month. Moreover, the results underly the relevance of longitudinal studies to fully characterize the molecular mechanisms mediating or blocking AD progression.

### Female 5XFAD Mice Show Proteomic Signatures Reflecting Increased Amyloid Burden

**Fig. 2:**
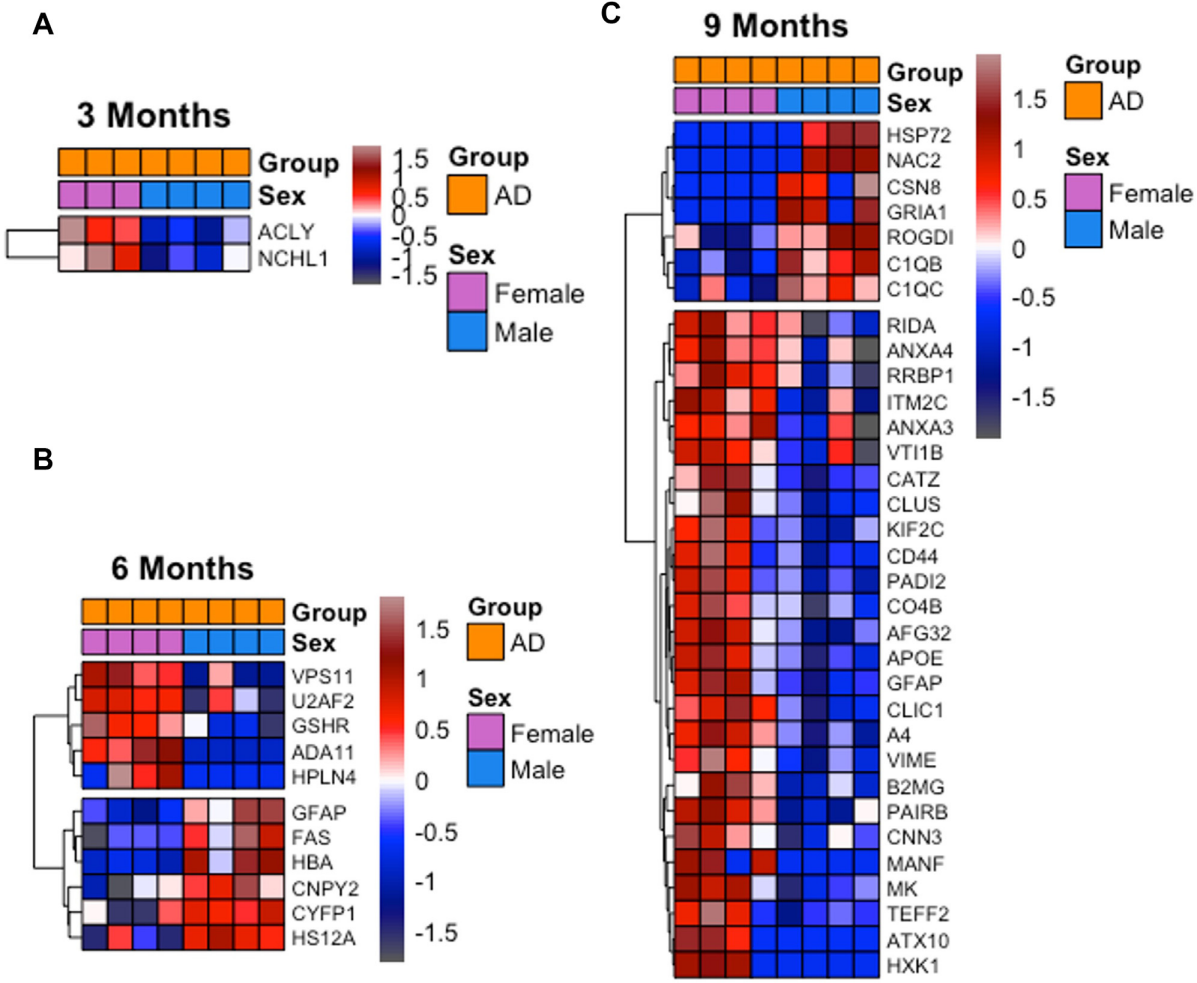
**Sex disparities in 5XFAD’s mice proteomic signatures across time.** Label-free proteomics analysis of sex-specific differentially expressed peptides in the hippocampus of 5XFAD mice; the heatmaps show the average intensity of peptides comparing female *versus* male 5XFAD mice at *A*, 3 months, *B*, 6 months, and *C*, 9 months. AD, Alzheimer’s disease.

Brain structure and function as well as brain disease progression are often known to be different between males and females (26) and our studies are designed to reveal specific sex differences in the mouse model. We investigated whether the differentially expressed peptides at each time point (Fig. 1*D*) included proteins that were differentially expressed between females *versus* males (5XFAD male *versus* 5XFAD female *p* ≤ 0.05, unpaired *t* test). We find an increasingly larger cluster of peptides to be significantly upregulated in female 5XFAD mice (Fig. 2). Specifically, we identified 2, 12, and 80 differentially expressed peptides between males and females in 5XFAD at 3, 6, and 9 months, respectively (Fig. 2 and supplemental Table S4). These annotate to 2, 11, and 33 annotated proteins at 3, 6, and 9 months, respectively (supplemental Table S4). In fact, of the total 94 sex-specific peptides identified as differentially expressed across our datasets, 81 are upregulated in female 5XFAD mice. Moreover, we matched our sex-specific protein list (supplemental Table S4) against DisGeNET’s AD biomarker database (CUI: C002395) and we found six novel AD-associated proteins (AFG32, ATX10, CSN8, KIF2C, ROGDI, VPS11); of these, only CSN8 and ROGDI are upregulated in males (Table 1).

**Table 1:** AD biomarkers tracking across AD progression

Time point	Up/down	Sex-specific	Protein name	Uniprot	GO:MF
3 Months	↑		Histone H1.4	P43274	nucleotide binding
			Plastin-3	Q99K51	calcium ion binding
3 Months	↓		Clathrin interactor 1	Q99KN9	protein binding
6 Months	↑		Lysophosphatidylserine lipase ABHD12	Q99LR1	hydrolase activity
			BTB/POZ domain-containing protein 8	D3YUB6	protein binding
		YES	ATPase GET3	O54984	nucleotide binding
			Protein kinase c-binding protein NELL2	Q61220	calcium ion binding
			Protein PALS2	Q9JLB0	protein binding
			UDP-N-acetylhexosamine pyrophosphorylase-like protein 1	Q3TW96	transferase activity
			Vacuolar protein-sorting associated protein 11 homolog	Q91W86	nucleotide binding
6 Months	↓		Glutamine amidotransferase-like class 1 domain-containing protein 1	Q8BFQ8	--
			Maestro heat-like repeat-containing protein family member 2B	Q7M6Y6	protein binding
9 Months	↑	YES	AFG3-like protein 2	Q8JZQ2	protein binding
		YES	COP9 signalosome complex subunit 8	Q8VBV7	protein binding
			Endoplasmic reticulum-Golgi intermediate compartment protein 3	Q9CQE7	protein self-association
		YES	Kinesin-like protein KIF2C	Q922S8	nucleotide binding
			Glycerol kinase	Q64516	protein binding
			Ragulator complex protein LAMTOR5	Q9D1L9	protein binding
			Arginine--tRNA ligase, cytoplasmic	Q9D0I9	nucleotide binding
		YES	Protein rogdi homolog	Q3TDK6	--
			RUN domain-containing protein 3B	Q96NL0	--
			Vacuolar protein-sorting associated protein 11 homolog	Q91W86	nucleotide binding
9 Months	↓	YES	Ataxin 10	P28658	protein binding
			3′(2′),5′-bisphosphate nucleotidase 1	Q9Z0S1	hydrolase activity
			ATPase GET3	O54984	nucleotide binding

List of differentially expressed proteins without a previous link to AD. The data was generated by Label-free proteomics analysis of 5XFAD mice hippocampus. The table includes the following: timepoint, directionality of dysregulation, sex-specific protein, name of annotated protein, Uniprot accession number.

**Fig. 3:**
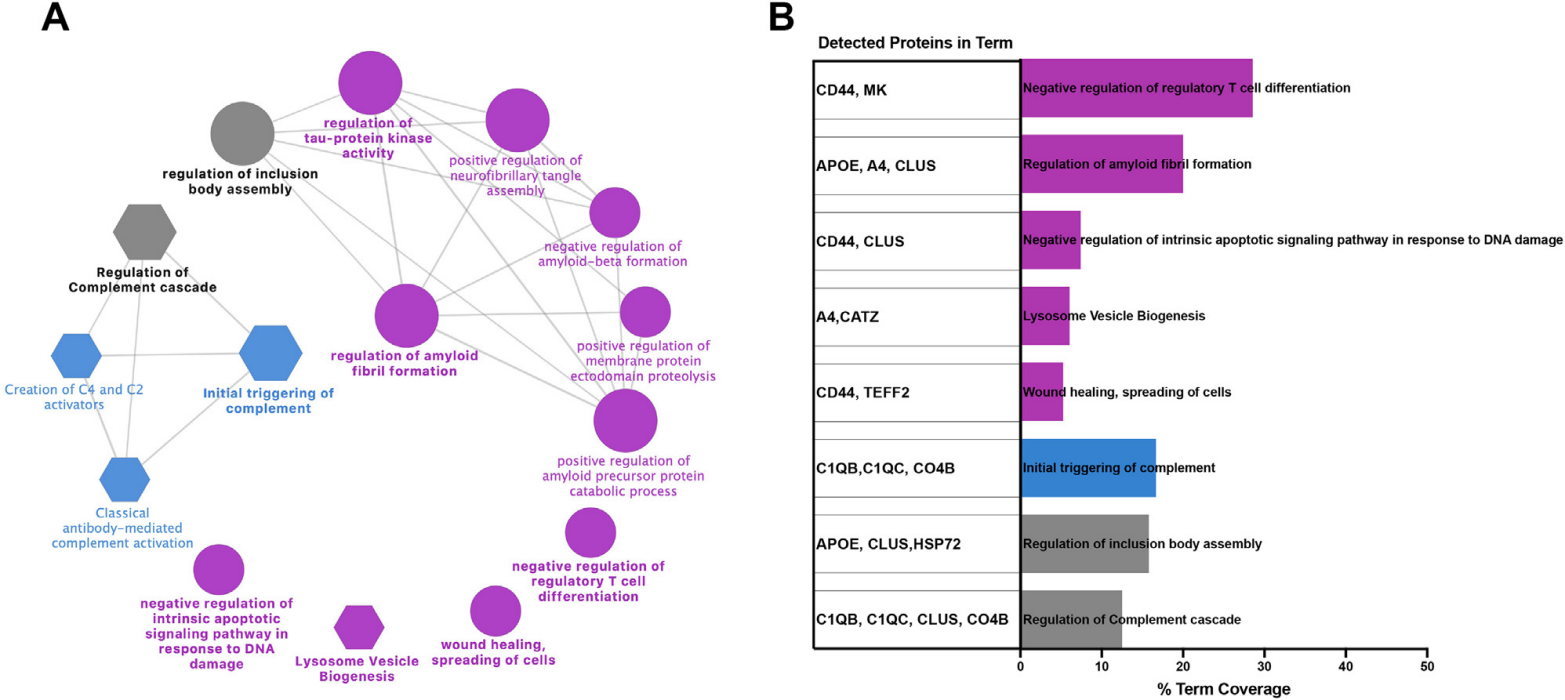
**Sex-specific characteristics of 5XFAD mice.** Label-free proteomics analysis of sex-specific differentially expressed peptides in the hippocampus of 5XFAD mice. *A*, enriched functional networks of sex-specific molecular signatures in 5XFAD, networks are inferred from sex-specific DEPs and functionally annotated to GO terms and Reactome pathways. Node color denotes origin of dataset (*blue* and *purple* for male and female differentially upregulated peptides, respectively), node shape differentiates term origin (*circle* = GO biological process; *hexagon* = Reactome pathway), and node size determines significance of GO/Reactome terms (terms with *p*-value ≤ 0.05 shown). Term significance was calculated *via* two-sided hypergeometric test and Bonferroni step-down correction. *B*, the barplot indicates percentage of term coverage per term identified, bar color denotes enriched group (*blue*, *purple*, and *gray* for male, female, and both); the left panel details the proteins identified in each term. AD, Alzheimer’s disease; DEP, differentially expressed protein; GO, gene ontology.

To further understand the functionally grouped networks that correlate with sex-linked proteomic signatures, we performed an enrichment analysis of our sex-specific peptide datasets. We segregated the male and female upregulated peptide datasets and performed a GO plus Reactome pathway enrichment analysis using the ClueGo/CluePedia plugins available in the Cytoscape software (https://cytoscape.org). Altogether, we identified nine GO terms and one pathway significantly enriched in the female upregulated dataset (purple nodes) clustered in a total of five distinct functional groups including regulation of amyloid fibril formation, wound healing/spreading of cells, lysosome vesicle biogenesis, negative regulation of intrinsic apoptotic signaling pathway in response to DNA damage, and negative regulation of regulatory T cell differentiation (Fig. 3, *A* and *B*). For the peptide datasets from males, we identified three pathways significantly enriched (blue nodes) clustered in a single functional group: initial triggering of complement (Fig. 3, *A* and *B*). Additionally, we identify one GO term (regulation of inclusion body assembly) and one pathway (regulation of complement cascade) which are shared between both female and male datasets (gray nodes) (Fig. 3, *A* and *B*). Regulation of the complement cascade is enriched in both datasets, this is expected as Aβ_40_ and Aβ_42_ and are known instigators of the complement cascades (27, 28) (Fig. 3, *A* and *B* and supplemental Table S4). Overall, this analysis reveals molecular signatures that are differentially enriched between female and male 5XFAD mice and provides hypotheses for verification, for example, increased amyloid burden of females *versus* males.

### Western Blots Confirm Increased GFAP Expression in 5XFAD Mouse Hippocampus and Cortex Over Time

**Fig. 4:**
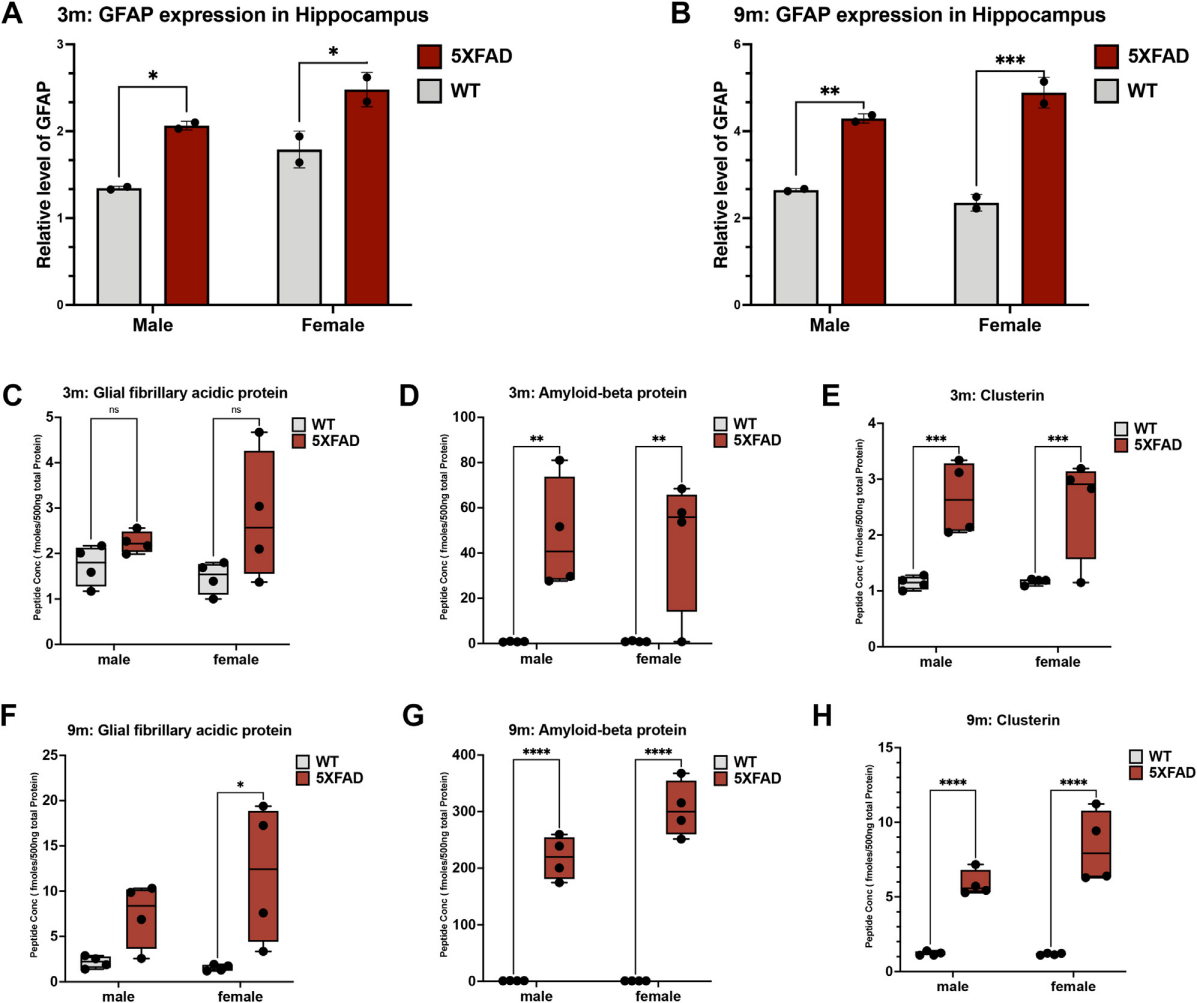
**Progressive neuroinflammation in 5XFAD mice hippocampus.** Western blot analysis of glial fibrillary acidic protein expression. *A*, hippocampus of WT and 5XFAD mice at 3 months (WT M *versus* 5XFAD M: *p*-value = 0.0274; WT F *versus* 5XFAD F: *p*-value = 0.0317). *B*, hippocampus of WT and 5XFAD mice at 9 months (WT M *versus* 5XFAD M: *p*-value = 0.0048; WT F *versus* 5XFAD F: *p*-value = 0.0009), PRM tracking of AD biomarkers in the hippocampal tissue of WT and 5XFAD mice at 3 months. *C*, GFAP monitoring. *D*, Aβ monitoring (WT M *versus* 5XFAD M: *p*-value = 0.0016; WT F *versus* 5XFAD F: *p*-value = 0.0016). *E*, clusterin monitoring (WT M *versus* 5XFAD M: *p*-value = 0.0009; WT F *versus* 5XFAD F: *p*-value = 0.0009). PRM tracking of AD biomarkers in the hippocampal tissue of WT and 5XFAD mice at 9 months. *F*, GFAP monitoring (WT F *versus* 5XFAD F: *p*-value = 0.0215). *G*, Aβ monitoring (WT M *versus* 5XFAD M: *p*-value < 0.0001; WT F *versus* 5XFAD F: *p*-value < 0.0001). *H*, clusterin monitoring (WT M *versus* 5XFAD M: *p*-value < 0.0001; WT F *versus* 5XFAD F: *p*-value < 0.0001). Aβ, amyloid-beta; AD, Alzheimer’s disease; PRM, parallel reaction monitoring.

**Fig. 5:**
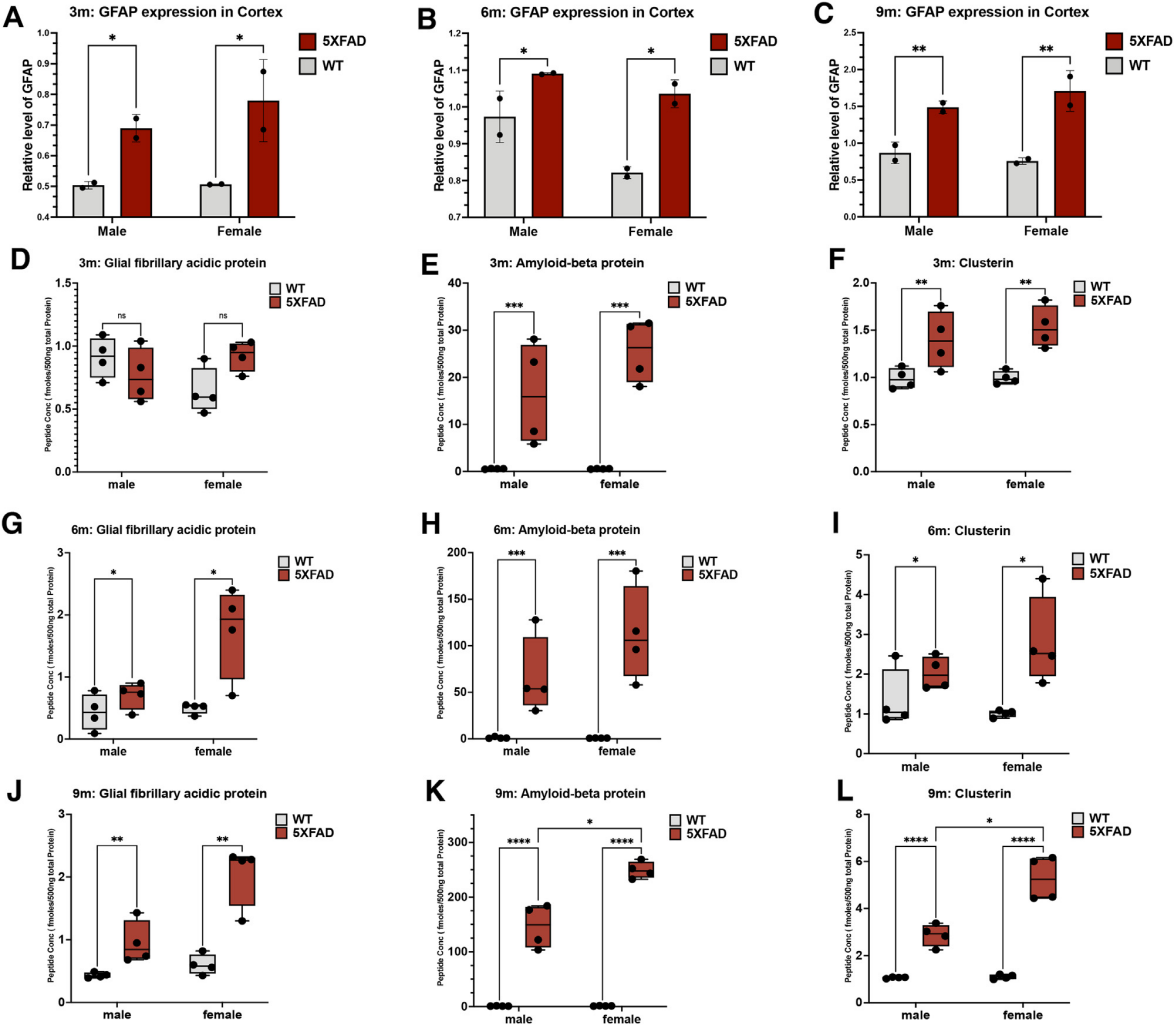
**Progressive neuroinflammation in 5XFAD mice cortex.** Western blot analysis of glial fibrillary acidic protein expression in WT and 5XFAD mice cortex. *A*, 3 months (WT M *versus* 5XFAD M: *p*-value = 0.0197; WT F *versus* 5XFAD F: *p*-value = 0.0197). *B*, 6 months (WT M *versus* 5XFAD M: *p*-value = 0.0163; WT F *versus* 5XFAD F: *p*-value = 0.0163). *C*, 9 months (WT M *versus* 5XFAD M: *p*-value = 0.0062; WT F *versus* 5XFAD F: *p*-value = 0.0062). PRM tracking of AD biomarkers in the cortical tissue of WT and 5XFAD mice at 3 months. *D*, GFAP monitoring. *E*, Aβ monitoring (WT M *versus* 5XFAD M: *p*-value = 0.0002; WT F *versus* 5XFAD F: *p*-value = 0.0002). *F*, clusterin monitoring (WT M *versus* 5XFAD M: *p*-value = 0.0015; WT F *versus* 5XFAD F: *p*-value = 0.0015). PRM tracking of AD biomarkers in the hippocampal tissue of WT and 5XFAD mice at 6 months. *G*, GFAP monitoring (WT M *versus* 5XFAD M: *p*-value = 0.0348; WT F *versus* 5XFAD F: *p*-value = 0.0348). *H*, Aβ monitoring (WT M *versus* 5XFAD M: *p*-value = 0.0010; WT F *versus* 5XFAD F: *p*-value = 0.0010;). *I*, clusterin monitoring (WT M *versus* 5XFAD M: *p*-value = 0.0248; WT F *versus* 5XFAD F: *p*-value = 0.0248). PRM tracking of AD biomarkers in the hippocampal tissue of WT and 5XFAD mice at 9 months. *J*, GFAP monitoring (WT M *versus* 5XFAD M: *p*-value = 0.0013; WT F *versus* 5XFAD F: *p*-value = 0.0013; 5XFAD F *versus* 5XFAD M: *p*-value = 0.0308). *K*, Aβ monitoring (WT M *versus* 5XFAD M: *p*-value < 0.0001; WT F *versus* 5XFAD F: *p*-value < 0.0001; 5XFAD F *versus* 5XFAD M: *p*-value = 0.0499). *L*, clusterin monitoring (WT M *versus* 5XFAD M: *p*-value < 0.0001; WT F *versus* 5XFAD F: *p*-value < 0.0001; 5XFAD F *versus* 5XFAD M: *p*-value = 0.0492). Aβ, amyloid-beta; AD, Alzheimer’s disease; PRM, parallel reaction monitoring.

To verify the temporal and sex-linked changes in significantly differentially expressed peptides in the 5XFAD mice, we selected Western blots to measure expression levels of glial fibrillary acidic protein at 3- and 9-month time points in hippocampus and at all time points in cortical tissue originally dissected from the same mice (Figs. 4, *A* and *B* and 5, *A*–*C*). In the hippocampus, we found 5XFAD mice to have significantly higher levels of glial fibrillary acidic protein than WT for both males and females at 3 months (FC_5XFAD/WT_ = 1.45 and *p*-value = 0.026) and 9 months (FC_5XFAD/WT_ = 1.84 and *p*-value ≤ 0.0001) (Fig. 4, *A* and *B*) consistent with mass spectrometry–based discovery data. Next, we examined the 3, 6, and 9, months changes in the cortex using Western and found higher levels of levels of glial fibrillary acidic protein in 5XFAD male and female mice, culminating in a highly significant two-fold increase at month 9 (3 months: FC_5XFAD/WT_ = 1.19 and *p*-value = 0.34; 6 months: FC_5XFAD/WT_ = 1.46 and *p*-value = 0.091; 9 months: FC_5XFAD/WT_ = 1.97 and *p*-value ≤ 0.0001) (Fig. 5, *A*–*C*). This overall validates the discovery data and workflows in general and shows the conclusions confirmed in hippocampus are robust when examined in adjacent tissue by an orthogonal method to the original discovery.

### PRM Analysis for High Throughput Verification of Differentially Expressed Peptides

To further confirm the results and to establish a high throughput method of validating multiple targets in many mouse samples simultaneously, we utilized PRM (supplemental Tables S1–S3) and first utilized it to measure GFAP to compare Western and PRM results. In PRM data, 5XFAD mouse levels of LEAENNLAAYRQEADEATLAR trend higher at 3-month in hippocampus tissue but are nonsignificant (Figs. 4*C* and 5*D*). However, significant increases were seen at 6 months (Fig. 5*G*) and 9 months (Fig. 5*J*) for cortex in WT *versus* AD (males and females) and significant increases at 9 months were seen for females in hippocampus while the PRM data is trending higher for males (Fig. 4*F*) ($\bar{X}$_WT_:3 months = 1.603 fmol/500 ng total protein and 9 months = 1.860 fmol/500 ng total protein). In summary, there is a 1.57 and 5.19 FC_5XFAD/WT_ increase of LEAENNLAAYRQEADEATLAR concentration in 5XFAD mice at 3 months and 9 months respectively; plus, we quantified a 3.83 FC_9M/3M_ temporal increase of LEAENNLAAYRQEADEATLAR in 5XFAD mice overall. Overall, these data confirm a temporal increase of glial fibrillary acidic protein over time in both hippocampal and cortical tissues for both female and male 5XFAD mice showing consistency with discovery data.

We verified Aβ^‘^s expression changes in hippocampus in a series of PRM experiments seen in Figure 4, *D* and *G*. While we found Aβ^‘^s LVFFAEDVGSNK peptide levels to be constant in WT hippocampus over time ($\bar{X}$_WT_:3 months = 0.9063 fmol/500 ng total protein and 9 months = 1.078 fmol/500 ng total protein), we detected highly upregulated levels of this peptide in hippocampus of 5XFAD mice over time, consistent with discovery studies ($\bar{X}$_5XFAD_: 3 months = 46.37 fmol/500 ng total protein and 9 months = 261.60 fmol/500 ng total protein) (Fig. 4, *D* and *G*). Overall, there is a 52.16 and 242.68 FC_5XFAD/WT_ increase of LVFFAEDVGSNK concentration in 5XFAD mice at 3 months and 9 months, respectively, over WT mice. Furthermore, there is a 5.64 FC_9M/3M_ increase in accumulated LVFFAEDVGSNK in 9-month 5XFAD mice *versus* 3 months.

We verified the discovery results on clusterin in hippocampus as seen in Figure 4, *E* and *H*. No difference was found between ASGIIDTLFQDR levels in WT mice at any time point ($\bar{X}$_WT_:3 months = 1.158 fmol/500 ng total protein and 9 months = 1.189 fmol/500 ng total protein) (Fig. 4, *E* and *H*). However, there is a significant increase of ASGIIDTLFQDR in 5XFAD mice *versus* WT over time in hippocampus ($\bar{X}$_5XFAD_: 3 months = 2.601 fmol/500 ng total protein and 9 months = 7.120 fmol/500 ng total protein) (Fig. 4, *E* and *H*). We calculated a 2.25 and 5.99 FC_5XFAD/WT_ increase of ASGIIDTLFQDR concentration in 5XFAD mice at 3 months and 9 months, respectively; plus, we quantified a 2.74 FC_9M/3M_ temporal increase of ASGIIDTLFQDR in the hippocampus of 5XFAD mice over this time. Overall, these PRM results demonstrate the reliability and consistency of the differentially expressed peptides observed in the discovery set seen in Figure 1 as well as confirm important sex and time-linked changes in AD.

### Sex-Linked Differences in AD Burden Over Time in Cortex Verified Using PRM

To verify the generality of sex-linked changes in proteomic variation, we also measured the levels of Aβ and clusterin in cortex using PRM. As for hippocampus, we found a steep temporal increase of Aβ^‘^s LVFFAEDVGSNK levels in 5XFAD mice ($\bar{X}$_5XFAD_: 3 months = 20.98 fmol/500 ng total protein, $\bar{X}$_5XFAD_: 6 months = 89.42 fmol/500 ng total protein, and 9 months = 197.9 fmol/500 ng total protein) (Fig. 5, *E*, *H* and *K*), whereas WT mice maintained Aβ levels below 1 fmol/500 ng total protein across the whole study. Overall, there is a 37.71, 89.42, and 204.55 FC_5XFAD/WT_ increase of LVFFAEDVGSNK concentration in 5XFAD mice at 3 months, 6 months, and 9 months respectively. Furthermore, there is a 9.43 FC_9M/3M_ increase in accumulated LVFFAEDVGSNK in 9-month 5XFAD mice. Of interest is that Aβ peptide levels of females are significantly higher than matched 5XFAD males by 9-month time (Fig. 5*K*).

Using ASGIIDTLFQDR levels as a surrogate for clusterin, we see levels in WT mouse cortex tissue varied very little ($\bar{X}$_WT_:3 months = 0.9913 fmol/500 ng total protein, $\bar{X}$_WT_:6 months = 1.180 fmol/500 ng total protein, and 9 months = 1.084 fmol/500 ng total protein) (Fig. 5, *F*, *I* and *L*). On the other hand, we observed an increase of ASGIIDTLFQDR in 5XFAD mice over time ($\bar{X}$_5XFAD_: 3 months = 1.466 fmol/500 ng total protein, $\bar{X}$_5XFAD_: 6 months = 2.418 fmol/500 ng total protein, and 9 months = 4.073 fmol/500 ng total protein) (Fig. 5, *F*, *I* and *L*). Overall, there is a 2.78 FC_9M/3M_ temporal increase of ASGIIDTLFQDR in 5XFAD mice. As seen for Aβ, clusterin peptide levels for females are significantly higher than matched 5XFAD males by 9-month time.

### Highly Overexpressed Protein Set Drives Neuroinflammation

While examining the volcano plots, we identified a group of peptides characterized by a high Log_2_FC_(5XFAD/WT)_ (>10) that becomes evident starting at month 6 (Fig. 1, *A*–*C*). We were curious to examine these top dysregulated signatures in 5XFAD mice, therefore, we created a class of highly expressed peptides for further analysis of their joint characteristics (FC_(5XFAD/WT)_ ≥ 16 and *p* ≤ 0.1, unpaired *t* test per time point) (supplemental Table S5). This screen identifies 85 highly expressed peptides that annotate to 43 proteins, all of which belong to the 99th percentile of their corresponding time point’s dysregulated set. In terms of specific upregulated peptides at 3-month, we identify three highly expressed peptides, this number increases to 31 peptides at 6-month and goes up to 51 peptides at 9 months. Of these, only an Aβ peptide (LVFFAEDVGSNK) is detected at all time points (supplemental Table S5 and supplemental Fig. S1, *A*–*C*). Additionally, seven peptides that annotate to apolipoprotein E, glial fibrillary acidic protein, midkine, and testican-2 are identified at both 6- and 9-month (supplemental Table S5). Apolipoprotein E and glial fibrillary acidic protein are known biomarkers of AD as indicated above (29, 30); the first plays a role in early amyloidosis, cholesterol metabolism, and inflammatory response, and the latter is an established marker of glial-specific neuroinflammation. Midkine is a heparine-binding cytokine, which regulates processes like tissue protection and inflammation, additionally, it is present in senile plaques of AD and therefore is suggested as a potential clinical target (31, 32). Testican-2 is a component of the extracellular matrix known for its calcium-binding properties (33) and it has been previously described to be accumulated in the brain of AD patients (34, 35). Interestingly, all these proteins are known to be upregulated in AD patients and have been explored as AD biomarkers (35, 36, 37). Thus, this mouse model is seen to recapitulate these molecular characteristics of the disease.

Starting at month-6, this upregulated protein set includes neuroinflammatory-related proteins like complement C4-B, glial fibrillary acidic protein, protein-arginine deiminase type-2, vimentin, and vitronectin that have long been associated with Alzheimer’s disease (21, 38, 39, 40, 41, 42) (supplemental Table S5). Also starting at month-6, we observe the emergence of proteins involved in platelet activation and aggregation pathways like platelet-activating factor acetylhydrolase IB subunit alpha, alpha-actinin-4, mesencephalic astrocyte-derived neurotrophic factor, clusterin, and Aβ precursor protein (supplemental Table S5). Platelet pathways are well described in AD, as Aβ_40_ is known to induce platelet activation and aggregation pathways, *via* hyperactivation of the cPLA2–PAF axis (43, 44).

**Fig. 6:**
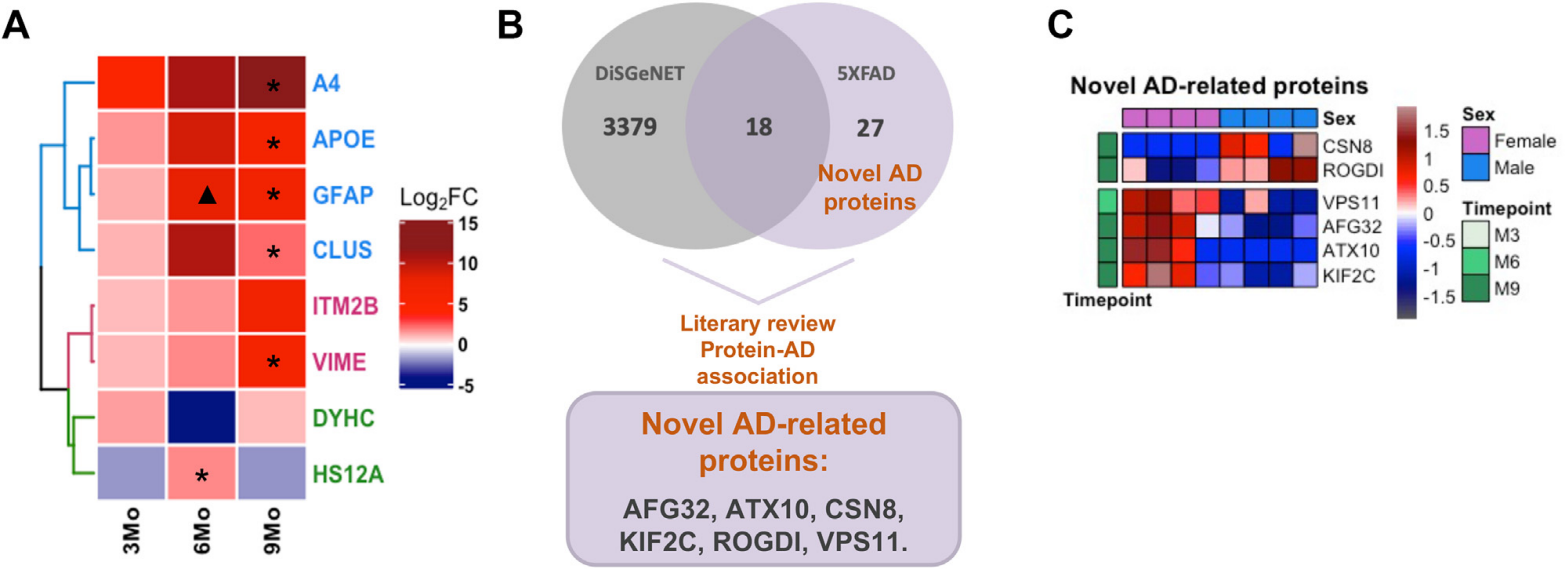
**AD biomarkers tracking across AD progression.***A*, label-free proteomics analysis of WT and AD mice hippocampus; the heatmap shows the average 5XFAD/WT Log_2_Fold change of the eight protein significantly expressed proteins across our longitudinal study (3 months, 6 months, and 9 months). The dendrogram based on the euclidean distance matrix identified three distinct clusters: *blue* (swift and consistent upregulation), *magenta* (gradual upregulation), and *green* (variable expression). Significant sex differences are annotated with ∗ (significantly upregulated in female mice) or ▲ (significantly upregulated in male mice). *B*, the Venn diagram shows the overlap between DiSGeNET’s Alzheimer Disease and gene-disease association dataset (#C0002395) and our list of sex-specific differentially expressed peptides. *C*, the heatmap shows the average intensity of the novel AD-related peptides comparing female *versus* male *5XF*AD mice. AD, Alzheimer’s disease.

Our highly upregulated protein list (supplemental Table S5) includes known AD biomarkers when matched with DisGeNET’s AD biomarker database (CUI: C002395), including Aβ precursor protein, apolipoprotein E (29, 45), glial fibrillary acidic protein (30), serine protease HTRA1, mesencephalic astrocyte-derived neurotrophic factor, protein-arginine deiminase type-2 (40), and integral membrane protein 2B. Further, out of the eight dysregulated proteins detected across all time points in the 5XFAD mice (Figs. 1*E* and 6*A*), five proteins (APP, apolipoprotein E, glial fibrillary acidic protein, clusterin, and integral membrane protein 2B) are also defined as AD biomarkers in DisGeNET’s AD biomarker database. On the other hand, we also expected novel AD-related proteins would be identified in our 5XFAD study. To this end, we also compared which of differentially expressed protein list were not represented in DisGeNET's AD dataset. We followed-up with a thorough literature review to eliminate any proteins with an established link to AD. The result includes 23 novel AD-related proteins (Table 1). To better understand the role of these proteins in AD dysregulation, we performed a functional annotation analysis focusing on molecular function GO terms. The 23 proteins were annotated to GO terms, including *protein binding* (AFG3-like protein 2, ataxin 10, BTB/POZ domain-containing protein 8, clathrin interactor 1, COP9 signalosome complex subunit 8, glycerol kinase, regulator complex protein LAMTOR5, maestro heat-like repeat-containing protein family member 2B, protein PALS2) and *nucleotide-binding* (ATPase GET3, H1.4 linker histone, Kinesin-like protein KIF2C, arginine-tRNA ligase, cytoplasmic, vacuolar protein-sorting associated protein 11 homolog), calcium ion binding (protein kinase c-binding protein NELL2, plastin-3), hydrolase activity (lysophosphatidylserine lipase ABHD12, 3′(2′),5′-bisphosphate nucleotidase 1) (Table 1).

### 5XFAD Recapitulates Molecular Features of the Human AD Proteome

A characteristic of clinically useful animal-based disease models is the ability to recapitulate molecular mechanisms that modulate disease progression in humans. In particular, accurate AD models should reflect robust expression of human pathological biomarkers. With that in mind, we further examined our datasets for established AD biomarkers. We start by tracking hippocampal Aβ, the most reliable core biomarker of human AD to date (46). Indeed, in the discovery datasets, we found Aβ‘s LVFFAEDVGSNK peptide to be increasingly upregulated in 5XFAD mice over time (Log_2_FC: 3 months = 6.7, 6 months = 8.0, and 9 months = 15.6) (supplemental Fig. S2, *A*–*C* and supplemental Table S5). This peptide is a standard measure of APP levels, further this tryptic fragment of mouse is identical to human Aβ (17–28) and is present in both Aβ_40_ and Aβ_42_ (2, 10, 46, 47). It is noteworthy that LVFFAEDVGSNK detected at month 9 is the single most upregulated peptide in 5XFAD mice across the whole study. To further capture the amyloid burden in our model, we also focused on the APP. We detect a total of 4, 10, and 15 APP peptides at month 3, 6, and 9, respectively (supplemental Fig. S2, *D*–*F*). The sum intensity of APP peptides is not significant at 3-month for 5XFAD mice when compared to WT mice, but APP overall increases in 5XFAD significantly over time (*p*-value: 3 months = 0.7264, 6 months = <0.0001, and 9 months = <0.0001, Šídák's multiple comparisons test) (supplemental Fig. S2, *D*–*F*). Specifically, APP’s fold change $\left({\bar{\text{X}}}_{\text{5XFAD}}\text{/}{\bar{\text{X}}}_{\text{WT}}\right)$ as time progresses goes from 2.73 at 3 months to 3.50 at 6 months and 5.59 at 9 months revealing a temporal accumulation of both APP and Aβ in 5XFAD mice which mimics the increased amyloid burden characteristic of human AD.

### Animal Phenotype Assessment

To confirm that the above-mentioned proteomic changes in 5XFAD mice are due to the onset of AD, we performed a phenotypic assessment of our mice. Y-maze is a known behavioral test used to evaluate short-term cognitive activity in mice, which is related to hippocampal damage. Mice normally tend to explore a new arm of the maze rather than visiting recently explored arms, a behavior tightly controlled by the hippocampus, prefrontal cortex, and basal forebrain. Y-maze behavior test was performed on 6-month-old mice; 5XFAD mice had a significantly lower alternation ratio than WT mice (*p*-value < 0.0001) (supplemental Fig. S3*A*). This experiment points to a higher hippocampal damage in 6-month-old 5XFAD mice than WT mice. Barnes maze is a known behavioral test used to evaluate hippocampal-dependent spatial learning and long-term cognitive activity in mice. Barnes maze behavior test was performed on 9-month-old mice (supplemental Fig. S3*B*); 5XFAD mice had a significantly higher latency to the target box than WT mice (*p*-value < 0.0001) at both day 5 and day 12. 5XFAD mice also had a significantly higher total errors than WT mice (*p*-value < 0.0001) at both day 5 and day 12 (supplemental Fig. S3*C*). Altogether, the data indicates that at 6-month, 5XFAD mice already carry higher hippocampal damage than age-matched WT mice. Lastly, we performed immunofluorescence staining to measure the amyloid burden in the brain of both 5XFAD and WT mice. 5XFAD mice showed a significantly higher amyloid plaque area and amyloid density than WT mice (supplemental Fig. S4, *A* and *B*). Altogether, the phenotypic differences between 5XFAD and WT mice are concordant with AD carrying mice.

## Discussion

Published almost 30 years ago, the amyloid cascade hypothesis (48) still dominates the AD field. Continued progress has identified multiple genetic risk factors and biomarkers (49), but therapeutic progress to prevent or reverse brain degeneration or cognitive decline related to Alzheimer’s disease has been modest, although a recent approved medication has potential to slow disease progression (50, 51). Longitudinal studies that monitor AD progression are imperative to better understand the initiating and propagating phases of AD. Studies of this nature are complicated to perform in humans due to the logistical constraints of retrieving multiple brain biopsies from healthy subjects. Thus, the field has heavily relied on relevant and suitable AD animal models to provide novel discovery of new targets, biomarkers, and pathways. The 5XFAD mouse model offers a variety of AD hallmarks related to both amyloid and tau-mediated molecular signatures. In this study, we saw an opportunity to uniquely explore the sex- and time-linked changes in proteins as a function of disease to both better understand these variables in a gold standard mouse model of AD as well as to uncover new pathways governing AD progression.

### Dramatically Increased Amyloid Burden in 5XFAD Mouse Hippocampus at 6- and 9-Month

Using label-free proteomics of hippocampus, we identify 488 differentially expressed proteins in AD when compared to age-matched WT mice at all time points. Of these, 93 proteins are differentially expressed between male and female 5XFAD mice. Comparing the proteomes across all three time points, we find a dramatic accumulation of differentially expressed peptides with progression of time where roughly two-thirds of these peptides are upregulated in the 5XFAD mice and 43 of the upregulated proteins are increased by 10-fold or more (Fig. 1, *A*–*C* and supplemental Table S5). Looking at the intersection of the data from the three time points (Fig. 1*E*), we find that eight proteins are always significantly differentially expressed with APP, apolipoprotein E, glial fibrillary acidic protein, clusterin progressively upregulated significantly, while integral membrane protein 2B and vimentin show less aggressive increases and cytoplasmic dynein 1 heavy chain, and heat shock protein 70 KDa protein 12A are attenuated (Fig. 6*A*). The first five are evidence-based biomarker candidates according to DisGeNET’s AD database, whereas vimentin, a phenotypic marker of glial cells and endothelial cells, is also a classical marker of astrogliosis, a phenomenon which is tightly related with neuronal loss. We confirmed the progressive temporal increases for clusterin, GFAP, and Aβ predicted by the discovery dataset with a combination of Western blots and PRM (Figs. 4 and 5). We also identified 23 novel AD-related proteins which were differentially expressed (Table 1). Functional annotation analysis shows that these proteins are mostly involved in protein or nucleotide binding (Table 1). Furthermore, seven proteins (PSL3 (52), ABHD12 (53), VPS11 (54, 55), AFG3L2 (56), GK (57), ROGDI (58), and ATX10 (59)) have established causal links to other diseases (supplemental Table S6). Of these, ABHD12, VPS11, ATX10, AFG3L2, and ROGDI are all associated with neurological disorders (respectively, PHARC syndrome (53), hypomyelinating leukodystrophy 12 (54), spinocerebellar ataxia type 10 (59) and 28 (56, 60), and Kohlschutter’s syndrome (58)) (supplemental Table S6).

### Temporal Proteomic Signatures of DNA Damage and Senescence Transition to Neuroinflammation Enrichments by 6 Months

Pathway analysis of differentially expressed proteins at various time points reveals DNA damage and cellular senescence pathways to be enriched at 3-month (supplemental Fig. S1). This is consistent with recent reports of increased SSB and DSB repeatedly found in the brain of AD patients (61, 62) where these studies implicate reactive oxygen species (ROS) and overall oxidative DNA damage as a major source of DNA damage early on in AD (63). Consistent with this, the activity of various DNA repair pathways are decreased in diverse brain regions of AD patients (64, 65, 66, 67). Cellular senescence has also been associated with higher incidence of AD (68), plus brain tissues from AD patients show upregulation of senescence-associated secretory phenotypes (69). Interestingly, DNA damage and oxidative-induced stress are known inducers of senescent astrocytes (70) reinforcing the importance of this finding.

Overall, protein expression changes at 3-month (55 protein changing) are modest compared to 6 and 9 months (202 and 307 proteins changing, respectively), but the remarkable increase of protein expression by 6 months is accompanied by a dramatic shift in pathway enrichment to vesicle/membrane transport dysregulation enrichments at 6- and 9-month, while the 9-month signature is dominated by enrichment of both innate immune and neutrophil degranulation pathways, while the initial DNA damage and cellular senescence signatures have faded. The enrichment of the innate immune system pathway is expected as the accumulation of Aβ creates a continuous state of neuroinflammation which constantly activates the innate immune system (71). It’s of note that the proteomic data demonstrates the progressive increases in APP and other neuroinflammatory mediators, but it’s clear that by 3-month, most of these processes are nascent in this model (supplemental Fig. S2; Figs. 4*C* and 5*D*). Thus, the data point to an initial model of ROS-type DNA damage signature in the first phase followed by enhanced neuroinflammation in a second phase well underway by 6-month.

### Proteomic Signatures of Neuroinflammation Enhanced in Female Mice

Higher AD prevalence and disease burden in females is well known, however, it remains poorly explained. We explored our data for sex-based differences of protein expression in 5XFAD mice with 81 upregulated peptides in female to 13 upregulated peptides in males at all time points (supplemental Table S4 and Fig. 2). Of these, six proteins are here newly associated with AD (AFG32, ATX10, CSN8, KIF2C, ROGDI, VPS11); and only CSN8 and ROGDI are upregulated in males (Table 1, Fig. 6, *B* and *C*). The remarkable increase in sex-linked differences with time in the model is reflective of the increased amyloid burden overall in females. Indeed, distinct inflammatory signals start as early as 3 months with 5XFAD female mice expressing higher levels of ATP-citrate synthase (ACLY), a metabolic (*de novo* lipogenesis) and transcription mediator. It has recently been reported that DNA damage (72) can induce an ATM–ACLY–NF-KB axis which initiates a pro-inflammatory response (73). It would be interesting to understand how early upregulation of ACLY in female 5XFAD mice impacts later inflammatory events and whether inhibiting ACLY could hinder the AD-related switch from DNA damage to neuroinflammatory signatures. Currently, *de novo* lipogenesis inhibitors are being investigated as a new class of therapeutics for multiple diseases ranging from cardiovascular diseases to AD (74). Bempedoic acid is the first approved drug of its class and therefore it would be interesting to test whether early administration of bempedoic acid would dampen or delay inflammatory signatures in 5XFAD female mice.

Our PRM studies verified the increases of many of these consistently upregulated proteins for all mice in 5XFAD comparisons *versus* WT over time, with the levels of clusterin and Aβ significantly higher in females *versus* matched 5XFAD males in cortex. The functional group network analysis (Fig. 3) of sex-linked differences identified five functional networks enriched in female 5XFAD mice inducing regulation of amyloid fibril formation, wound healing/spreading of cells, lysosome vesicle biogenesis, negative regulation of intrinsic apoptotic signaling pathway in response to DNA damage, and negative regulation of regulatory T cell differentiation. In addition, one functional network (initial triggering of the complement) was seen enriched in male mice 5XFAD (Fig. 3). Regulation of the complement cascade pathway is enriched in both female and male sex-specific upregulated peptide datasets (Fig. 3). As glucose and lipid metabolism dysregulation have been proposed to occur in the 5XFAD model prior to AD pathogenesis (9), and as the DNA damage signature, which is enhanced in females, is seen to be triggered early in our data, investigation of ROS *versus* neuroinflammation targets appears to be a promising point for further investigation in this model.

## Conclusion

Our data provide a wealth of novel stage- and sex-specific pathway information for the 5XFAD mouse model. At 3-month, disease progression has modest but significant effects on the proteome, with DNA damage and senescence signatures evident but quickly replaced by amyloid-driven neuroinflammation by 6-month followed by large increases in amyloid burden by 9-month. These proteomic burdens vary in cortex *versus* hippocampus and in males *versus* females, with consistently increased amyloid-related burden in 5XFAD females by 9-month. Overall, these data provide a roadmap for designing and testing protein-targeted interventions in this model to better understand AD pathology, biomarkers, and molecular outcomes.

## Data Availability

The datasets supporting the conclusions of this article are available in publicly available repositories. The mass spectrometry proteomics data relative to label free mass spectra have been deposited to the ProteomeXchange (75) Consortium *via* the PRIDE (76) partner repository with the dataset identifier PXD030161 and can be found at https://doi.org/10.6019/PXD30161. PRM data have been deposited to MassIVE with identifier MSV000088507 and can be accessed at https://doi.org/10.25345/C5TP2M.

## Supplemental data

This article contains supplemental data.

## Conflict of interest

The authors declare no competing interests.
