# Supplementary material for: Temporal and Sex-Linked Protein Expression Dynamics in a Familial Model of Alzheimer’s Disease
Source: Mol Cell Proteomics. 2022 Aug 6;21(9):100280. doi: 10.1016/j.mcpro.2022.100280 (PMC9483563; doi:10.1016/j.mcpro.2022.100280)
Supplement: Supplemental Figures S1–S7 [file mmc1.pdf]

SUPPLEMENTARY FIGURES: Temporal and sex-linked protein expression dynamics in a familial model of Alzheimer's Disease

TABLE OF CONTENTS

Supplementary Figures Legends	page 2
Supplementary Figure 1	page 3
Supplementary Figure 2	page 4
Supplementary Figure 3	page 5
Supplementary Figure 4	page 6
Supplementary Figure 5	page 7
Supplementary Figure 6	page 8
Supplementary Figure 7	page 9
Supplementary materials provided as separate files	page 10

SUPPLEMENTARY FIGURES LEGENDS

SUPPLEMENTARY FIGURE 1. Temporally exclusive proteins show that distinct pathways regulate AD progression. A PANTHER Overrepresentation test with Reactome pathways annotation (Fischer's exact test and FDR correction FDR $P < 0.05$) was performed on significantly expressed proteins in 5XFAD mice that are exclusive for a single timepoint (3 months U 6 months U 9 months). The Table shows the significantly overrepresented pathways.

SUPPLEMENTARY FIGURE 2. Amyloid-beta precursor protein (APP) an Aβ peptide tracking across the study. Label-free proteomics analysis of WT and 5XFAD mice hippocampus; The bar graph bar shows the intensity of Aβ peptide (LVFFAEDVGSNK) in WT *versus* AD mice at: **A.** 3 Months (A**β**: p- value=<0.0001), **B**. 6 Months (A**β**: p-value=<0.0001), and **C.** 9 Months (A**β**: p-value=<0.0001). The bar graph bar shows the intensity of APP and Aβ (LVFFAEDVGSNK) peptides in WT *versus* AD mice at: **D.** 3 Months (APP: WT vs 5XFAD: p-value=0.7264) **E**. 6 Months (APP: WT vs 5XFAD: p-value=<0.0001), and **F.** 9 Months (APP: WT vs 5XFAD: p-value=<0.0001); 2way ANOVA with Sídák's correction.

SUPPLEMENTARY FIGURE 3. Cognitive activity assessment of 5XFAD mice. A, Short-term cognitive activity was assessed in six-month-old mice of the indicated genotypes using the Y-maze (n = 18–30 mice/group). Data are presented as the mean ± min and max values and compared by unpaired student t test (WT vs 5XFAD: p-value=<0.0001). **B and C,** Long-term cognitive activity was evaluated in nine-month-old mice of the indicated genotypes using the Barnes maze test (n = 13–21 mice/group). **B,** Latency to the target box (WT vs 5XFAD: Day 5 p-value=0.0015 and Day 12 p-value=0.0017 compared by 2way ANOVA with Šídák's correction). **C,** Total errors (WT vs 5XFAD: Day 5 p-value=0.0034 and Day 12 p-value=0.0037 compared by 2way ANOVA with Šídák's correction).

SUPPLEMENTARY FIGURE 4. Amyloid beta accumulation in 5XFAD mouse brains. Brain cortex sections were prepared from nine-month-old WT and 5XFAD mice. Brain sections were stained with anti-6E10 antibody (red) to label amyloid deposits. Scale bar: 100 μm. Representative images from at least three independent experiments are shown. The area covered by $6E10^{+}$ Aβ plaques (**A**) and the immunodensity of anti-6E10 (**B**) in the cortex were quantified. All data are presented as the mean ± min and max values and compared by unpaired student t test (**A –** WT vs 5XFAD: p-value=<0.0001. **B –** WT vs 5XFAD: p-value=<0.0001).

SUPPLEMENTARY FIGURE 5. PRM assay standard curve for LVFFAEDVGSNK (amyloid-beta precursor protein).

SUPPLEMENTARY FIGURE 6. PRM assay standard curve for LEAENNLAAYRQEADEATLAR (glial fibrillary acidic protein).

SUPPLEMENTARY FIGURE 7. PRM assay standard curve for ASGIIDTLFQDR (clusterin).

SUPPLEMENTARY FIGURES

Supplementary Figure 1

Reactome Pathways	Fold Enrichment	P-value	Timepoint
DNA Damage/Telomere Stress Induced Senescence	42.81	4.30E-02	3 months
Cellular Senescence	27.31	2.33E-02	3 months
Membrane Trafficking	4.84	3.16E-02	6 months
Vesicle-mediated transport	4.26	3.27E-02	6 months
Innate Immune System	4.34	2.57E-07	9 months
Neutrophil degranulation	5.57	3.25E-06	9 months
Membrane Trafficking	4.21	1.59E-03	9 months
Vesicle-mediated transport	3.98	1.66E-03	9 months
Cellular responses to stress	4.54	7.31E-03	9 months

Supplementary Figure 2

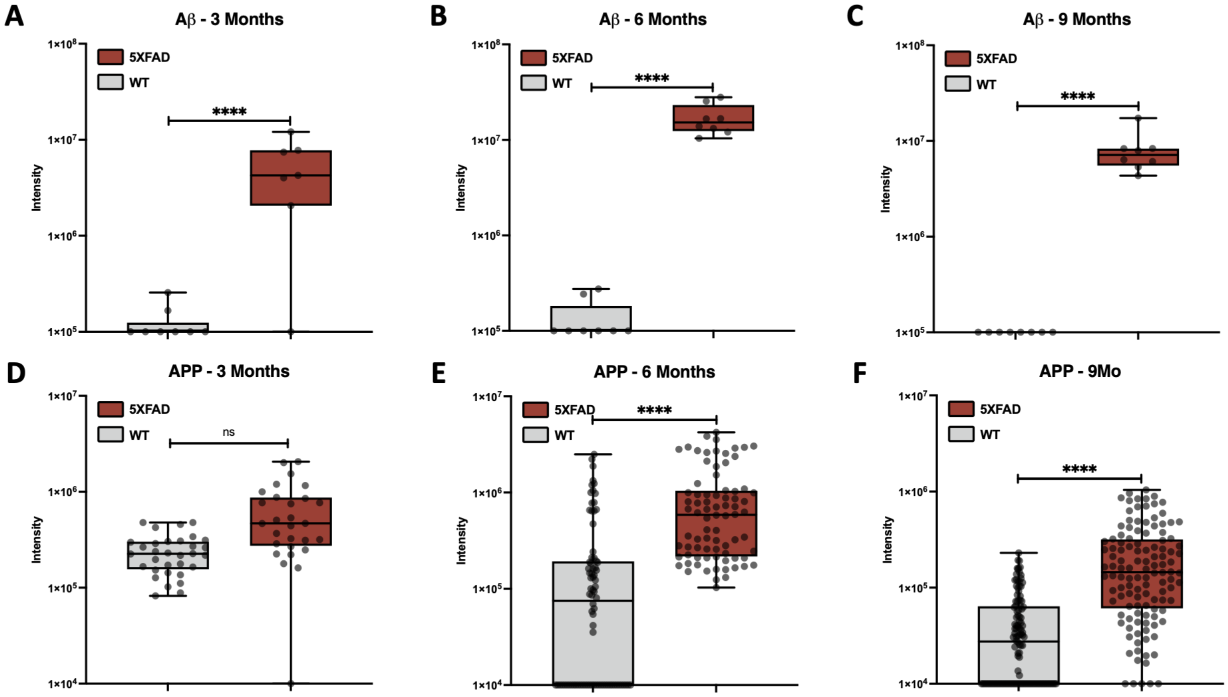

Supplementary Figure 3

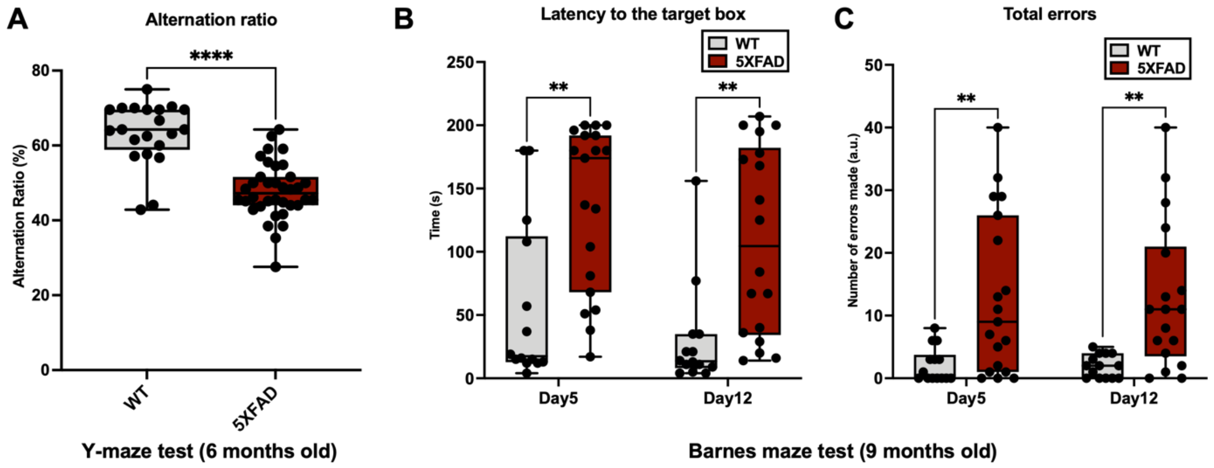

Supplementary Figure 4

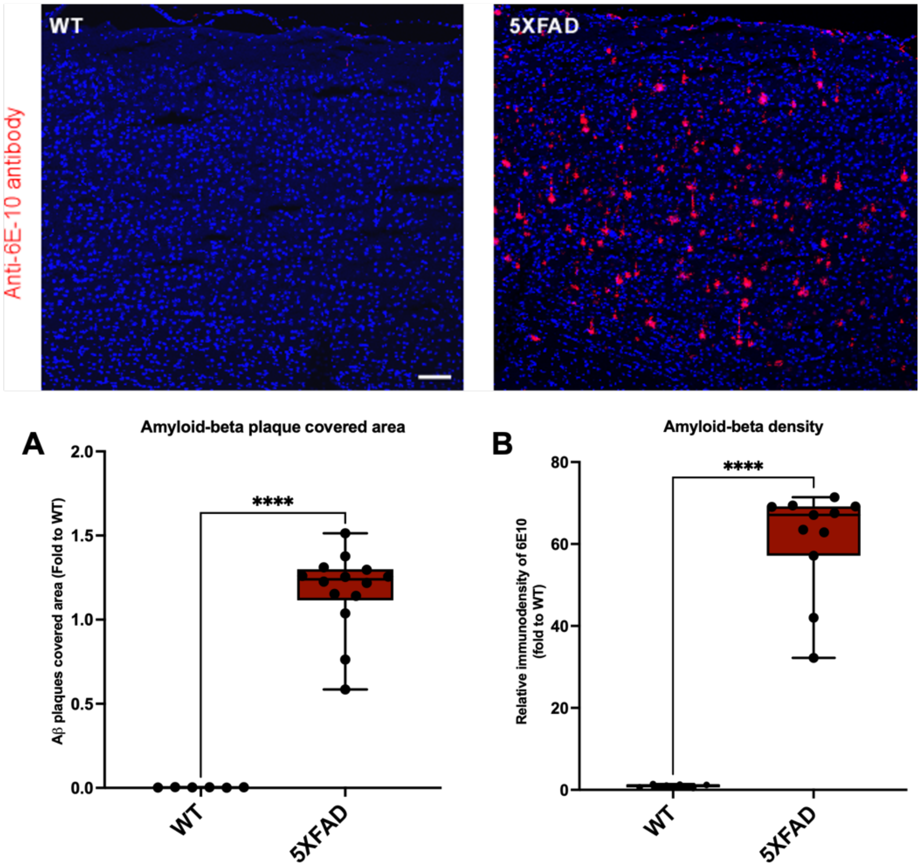

Supplementary Figure 5

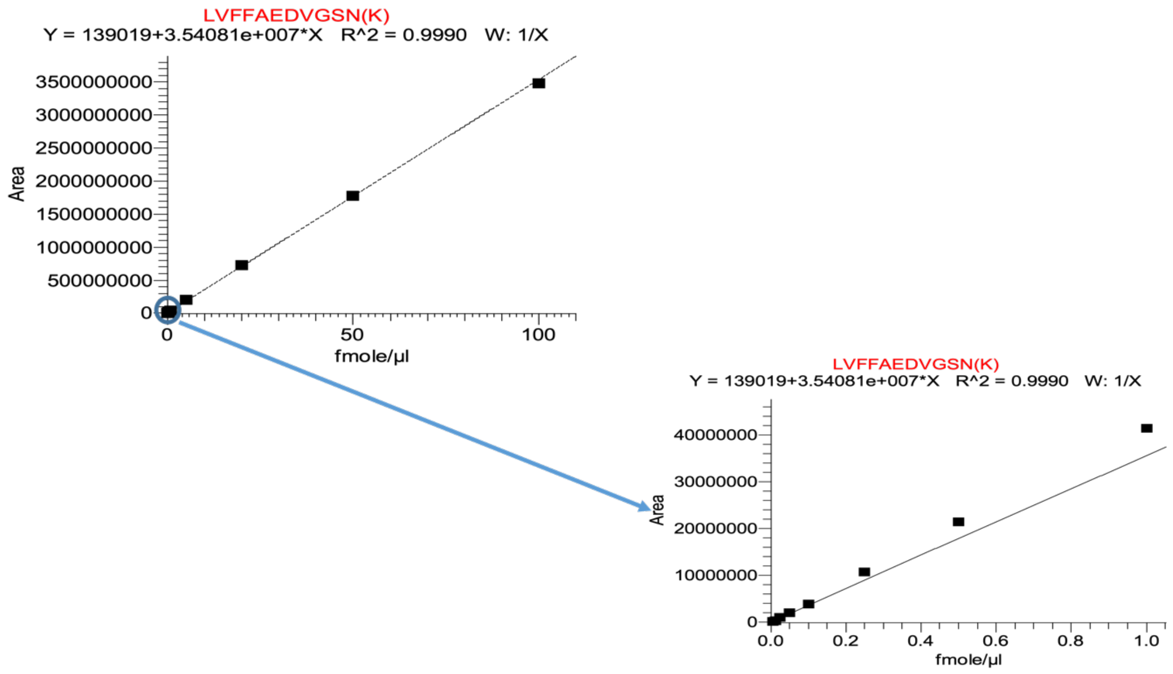

Supplementary Figure 6

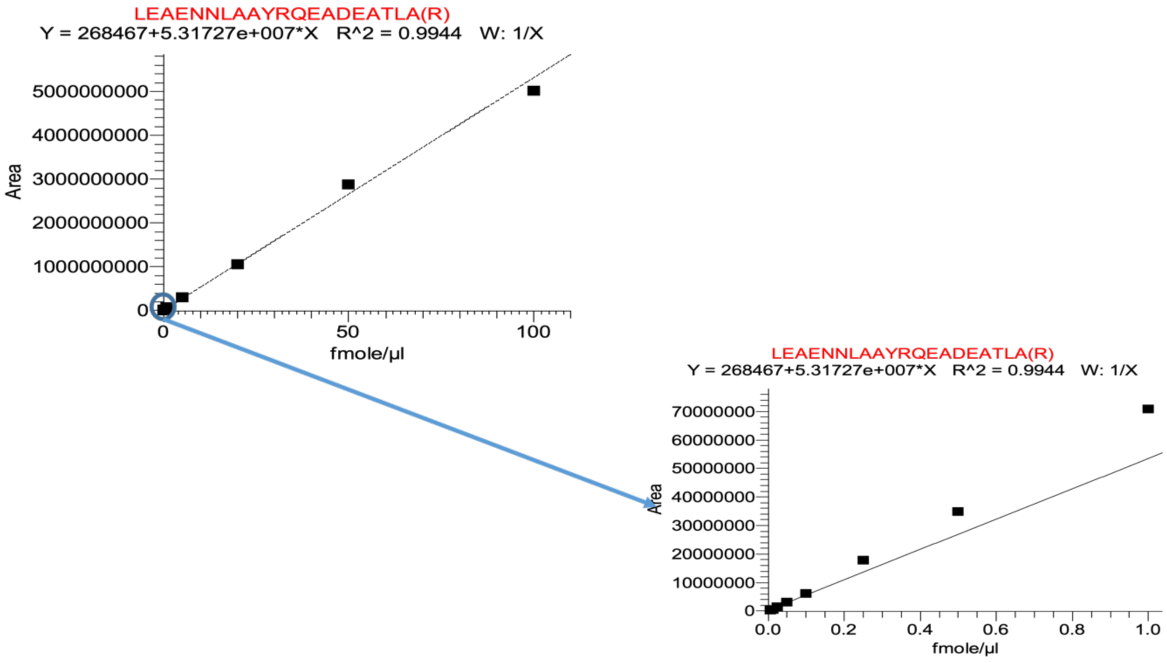

Supplementary Figure 7

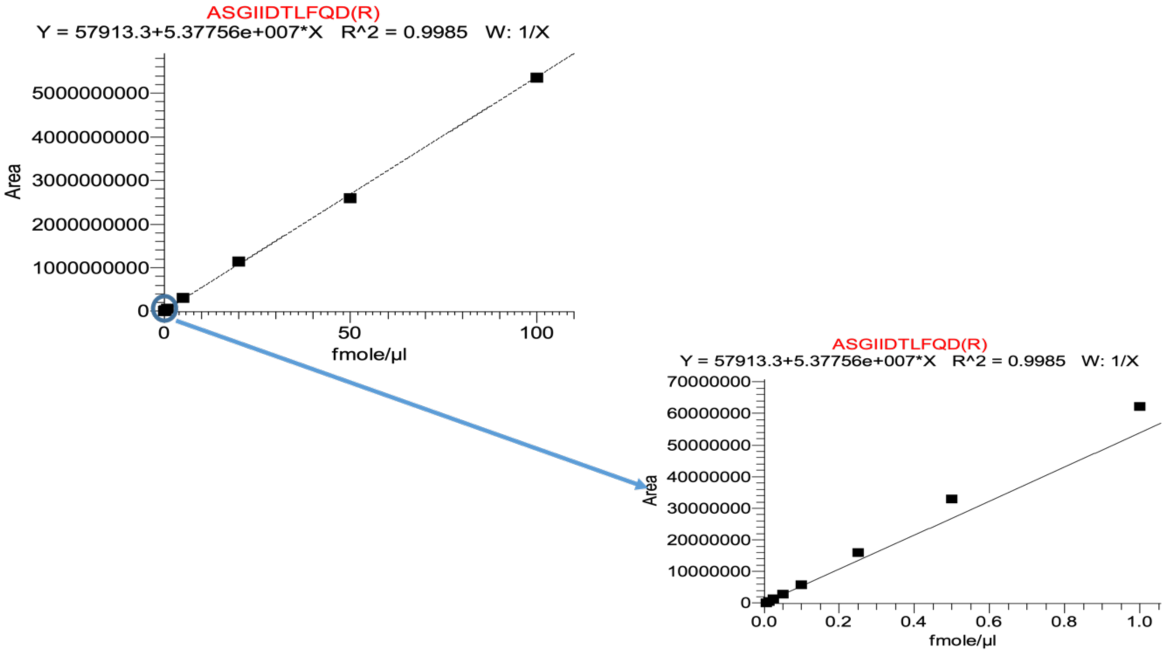

SUPPLEMENTARY MATERIALS (provided as separate files)

Supplementary Table 1. Selected proteins and peptides for PRM verification.

Supplementary Table 2. Targeted precursors and collision energies and selected quantifiers.

Supplementary Table 3. Linearity, limit of detection (LOD) and limit of quantification (LOQ) for the five authentic peptides.

Supplementary Table 4. List of sex-specific up-regulated peptides in AD mice (5XFAD/WT 5XFAD/WT $0.5 \geq FC \geq 2$ and $p \leq 0.1$, unpaired t-test and subsequent 5XFAD male vs 5XFAD female $p \leq 0.05$, unpaired t-test) identified by Label-free proteomics analysis of mice hippocampus. Table includes Uniprot accession number, name of annotated protein, timepoint, sex group in which the peptide is up-regulated, and peptide sequence.

Supplementary Table 5. List of highly up-regulated peptides in AD mice (5XFAD/WT Log2FC≥4 and $p \leq 0.1$, unpaired t-test) identified by label-free proteomics analysis of mice hippocampus. Table includes Uniprot accession number, name of annotated protein, Log10p-value(5XFAD/WT), FC(5XFAD/WT), and peptide sequence.

Supplementary Table 6. List of novel AD-associated proteins that have a causal link with other diseases. The table includes timepoint, up/down-regulation in AD vs WT, protein name, Uniprot accession identifier, gene name, and disease. (* denotes proteins with differential protein expression between male and female 5XFAD mice)

Supplementary Material. Single peptide identification for label-free quantitative proteomic experiment. Tables include peptide sequence, PEAKS quality score, PEAKS significance score, average ppm, average intensity, peptide abundance per sample, average peptide abundance per genetic background group, group profile, maximum ratio, number of vectors, Uniprot accession identifier, and post-translational modification.

Supplementary Material. Additional peptide features for label-free quantitative proteomic experiment. Tables include peptide sequence, peptide mass, peptide length, m/z, precursor charge, RT, source file, Uniprot accession identifier, and post-translational modification.

Supplementary Material. Chromatograms for PRM quantification of the selected analytes. Single chromatograms for each of the three selected analytes and respective isotopically labeled standard are shown in a single-view page organized per sample.

Supplementary Material. Annotated sample ID table.
