# Supplementary material for: Temporal and Sex-Linked Protein Expression Dynamics in a Familial Model of Alzheimer’s Disease
Source: Mol Cell Proteomics. 2022 Aug 6;21(9):100280. doi: 10.1016/j.mcpro.2022.100280 (PMC9483563; doi:10.1016/j.mcpro.2022.100280)
Supplement: Supplemental Data 3 [file mmc5.pdf]

Hippocampus Samples

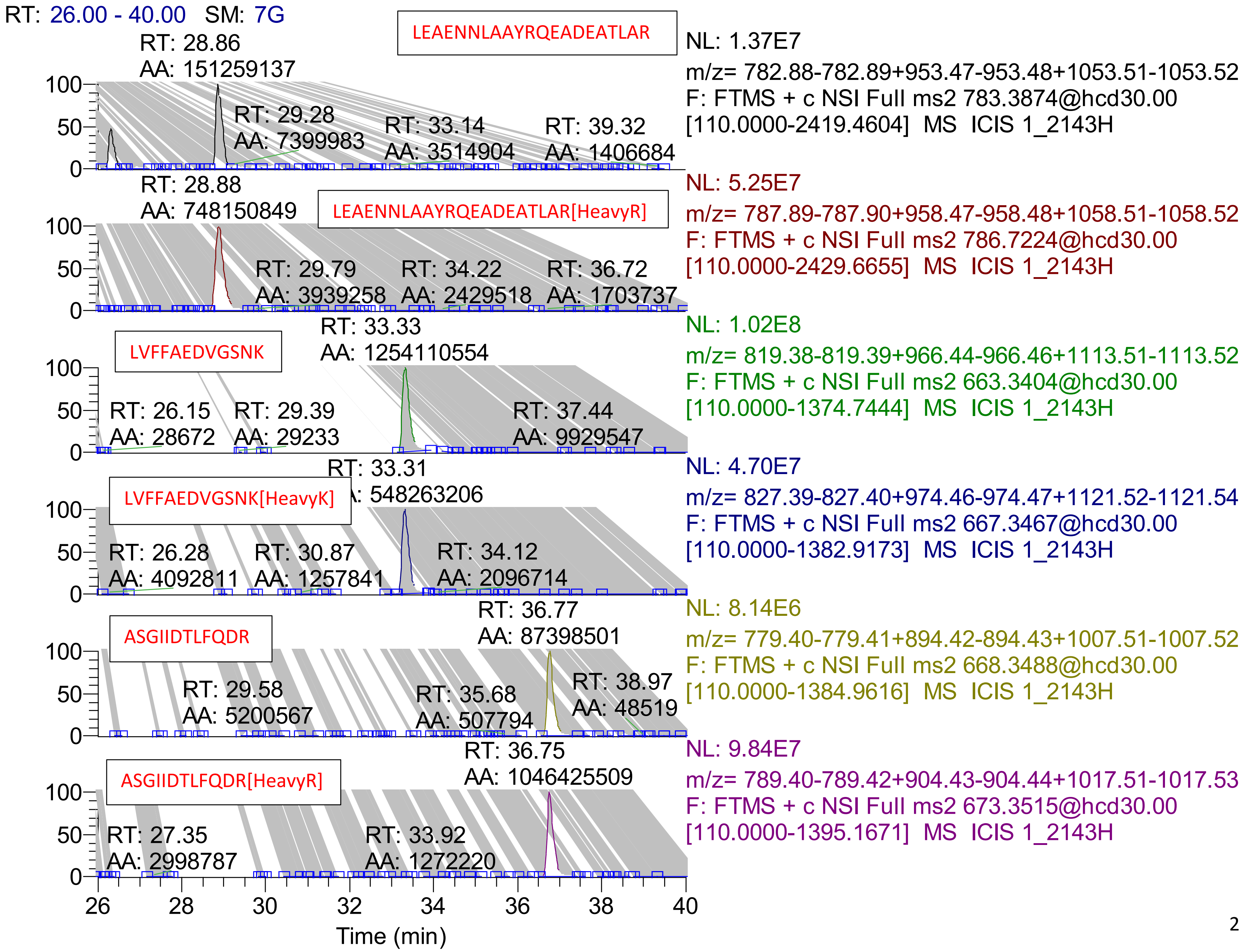

RT: 26.00 - 40.00 SM: 7G
LEAENNLAAYRQEADEATLAR
RT: 28.86 AA: 151259137
RT: 29.28 AA: 7399983
RT: 33.14 AA: 3514904
RT: 39.32 AA: 1406684
NL: 1.37E7
m/z= 782.88-782.89+953.47-953.48+1053.51-1053.52
F: FTMS + c NSI Full ms2 783.3874@hcd30.00
[110.0000-2419.4604] MS ICIS 1_2143H
LEAENNLAAYRQEADEATLAR[HeavyR]
RT: 28.88 AA: 748150849
RT: 29.79 AA: 3939258
RT: 34.22 AA: 2429518
RT: 36.72 AA: 1703737
NL: 5.25E7
m/z= 787.89-787.90+958.47-958.48+1058.51-1058.52
F: FTMS + c NSI Full ms2 786.7224@hcd30.00
[110.0000-2429.6655] MS ICIS 1_2143H
LVFFAEDVGSNK
RT: 33.33 AA: 1254110554
RT: 26.15 AA: 28672
RT: 29.39 AA: 29233
RT: 37.44 AA: 9929547
NL: 1.02E8
m/z= 819.38-819.39+966.44-966.46+1113.51-1113.52
F: FTMS + c NSI Full ms2 663.3404@hcd30.00
[110.0000-1374.7444] MS ICIS 1_2143H
LVFFAEDVGSNK[HeavyK]
RT: 33.31 548263206
RT: 26.28 AA: 4092811
RT: 30.87 AA: 1257841
RT: 34.12 AA: 2096714
NL: 4.70E7
m/z= 827.39-827.40+974.46-974.47+1121.52-1121.54
F: FTMS + c NSI Full ms2 667.3467@hcd30.00
[110.0000-1382.9173] MS ICIS 1_2143H
ASGIIDTLFQDR
RT: 36.77 AA: 87398501
RT: 29.58 AA: 5200567
RT: 35.68 AA: 507794
RT: 38.97 AA: 48519
NL: 8.14E6
m/z= 779.40-779.41+894.42-894.43+1007.51-1007.52
F: FTMS + c NSI Full ms2 668.3488@hcd30.00
[110.0000-1384.9616] MS ICIS 1_2143H
ASGIIDTLFQDR[HeavyR]
RT: 36.75 AA: 1046425509
RT: 27.35 AA: 2998787
RT: 33.92 AA: 1272220
NL: 9.84E7
m/z= 789.40-789.42+904.43-904.44+1017.51-1017.53
F: FTMS + c NSI Full ms2 673.3515@hcd30.00
[110.0000-1395.1671] MS ICIS 1_2143H
100
50
0
26 28 30 32 34 36 38 40
Time (min)

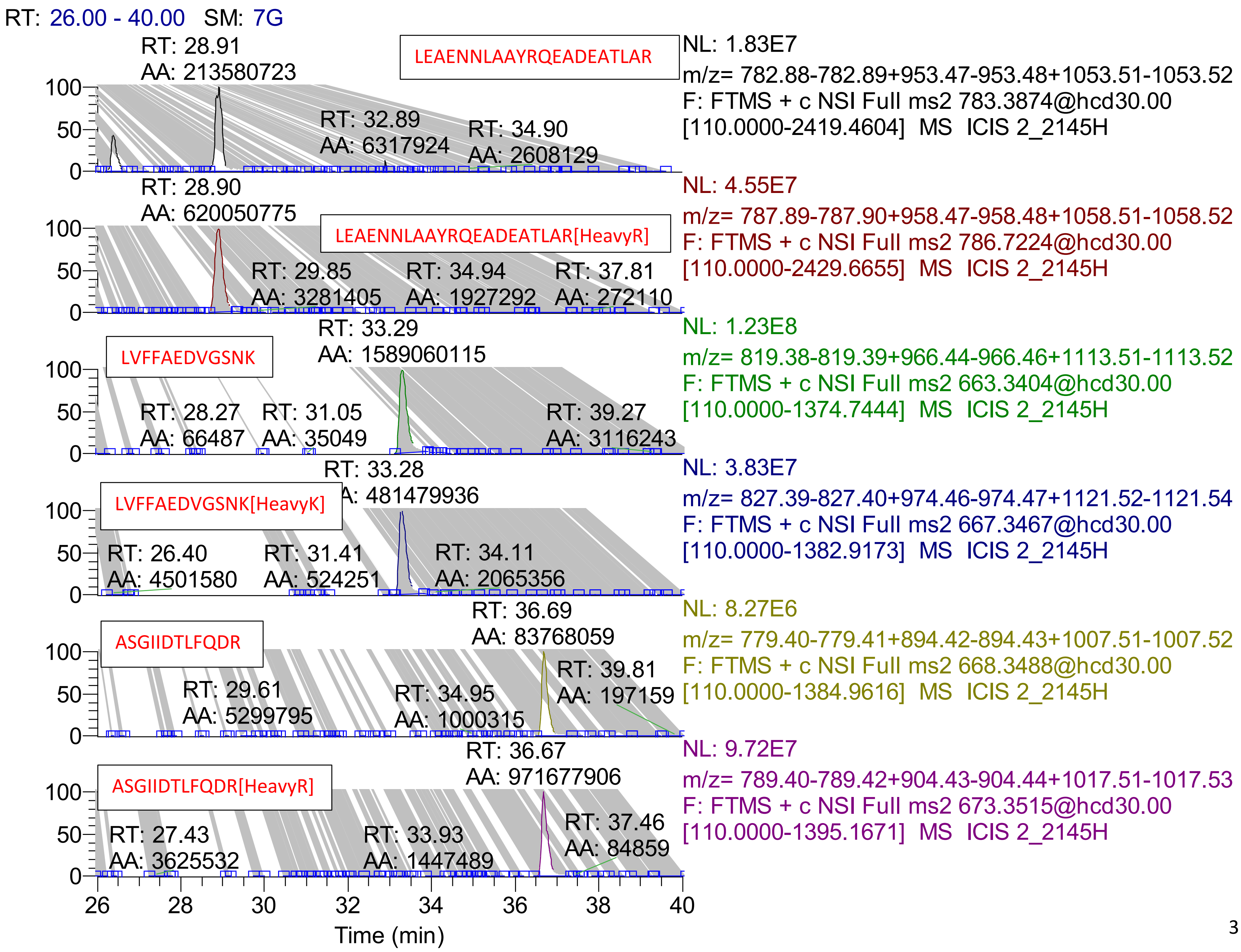
RT: 26.00 - 40.00 SM: 7G
RT: 28.91
AA: 213580723
LEAENNLAAYRQEADEATLAR
NL: 1.83E7
m/z= 782.88-782.89+953.47-953.48+1053.51-1053.52
F: FTMS + c NSI Full ms2 783.3874@hcd30.00
[110.0000-2419.4604] MS ICIS 2_2145H
RT: 32.89
AA: 6317924
RT: 34.90
AA: 2608129
RT: 28.90
AA: 620050775
LEAENNLAAYRQEADEATLAR[HeavyR]
NL: 4.55E7
m/z= 787.89-787.90+958.47-958.48+1058.51-1058.52
F: FTMS + c NSI Full ms2 786.7224@hcd30.00
[110.0000-2429.6655] MS ICIS 2_2145H
RT: 29.85
AA: 3281405
RT: 34.94
AA: 1927292
RT: 37.81
AA: 272110
RT: 33.29
AA: 1589060115
LVFFAEDVGSNK
NL: 1.23E8
m/z= 819.38-819.39+966.44-966.46+1113.51-1113.52
F: FTMS + c NSI Full ms2 663.3404@hcd30.00
[110.0000-1374.7444] MS ICIS 2_2145H
RT: 28.27
AA: 66487
RT: 31.05
AA: 35049
RT: 39.27
AA: 3116243
RT: 33.28
A: 481479936
LVFFAEDVGSNK[HeavyK]
NL: 3.83E7
m/z= 827.39-827.40+974.46-974.47+1121.52-1121.54
F: FTMS + c NSI Full ms2 667.3467@hcd30.00
[110.0000-1382.9173] MS ICIS 2_2145H
RT: 26.40
AA: 4501580
RT: 31.41
AA: 524251
RT: 34.11
AA: 2065356
RT: 36.69
AA: 83768059
ASGIIDTLFQDR
NL: 8.27E6
m/z= 779.40-779.41+894.42-894.43+1007.51-1007.52
F: FTMS + c NSI Full ms2 668.3488@hcd30.00
[110.0000-1384.9616] MS ICIS 2_2145H
RT: 29.61
AA: 5299795
RT: 34.95
AA: 1000315
RT: 39.81
AA: 197159
RT: 36.67
AA: 971677906
ASGIIDTLFQDR[HeavyR]
NL: 9.72E7
m/z= 789.40-789.42+904.43-904.44+1017.51-1017.53
F: FTMS + c NSI Full ms2 673.3515@hcd30.00
[110.0000-1395.1671] MS ICIS 2_2145H
RT: 27.43
AA: 3625532
RT: 33.93
AA: 1447489
RT: 37.46
AA: 84859
100
50
0
26
28
30
32
34
36
38
40
Time (min)

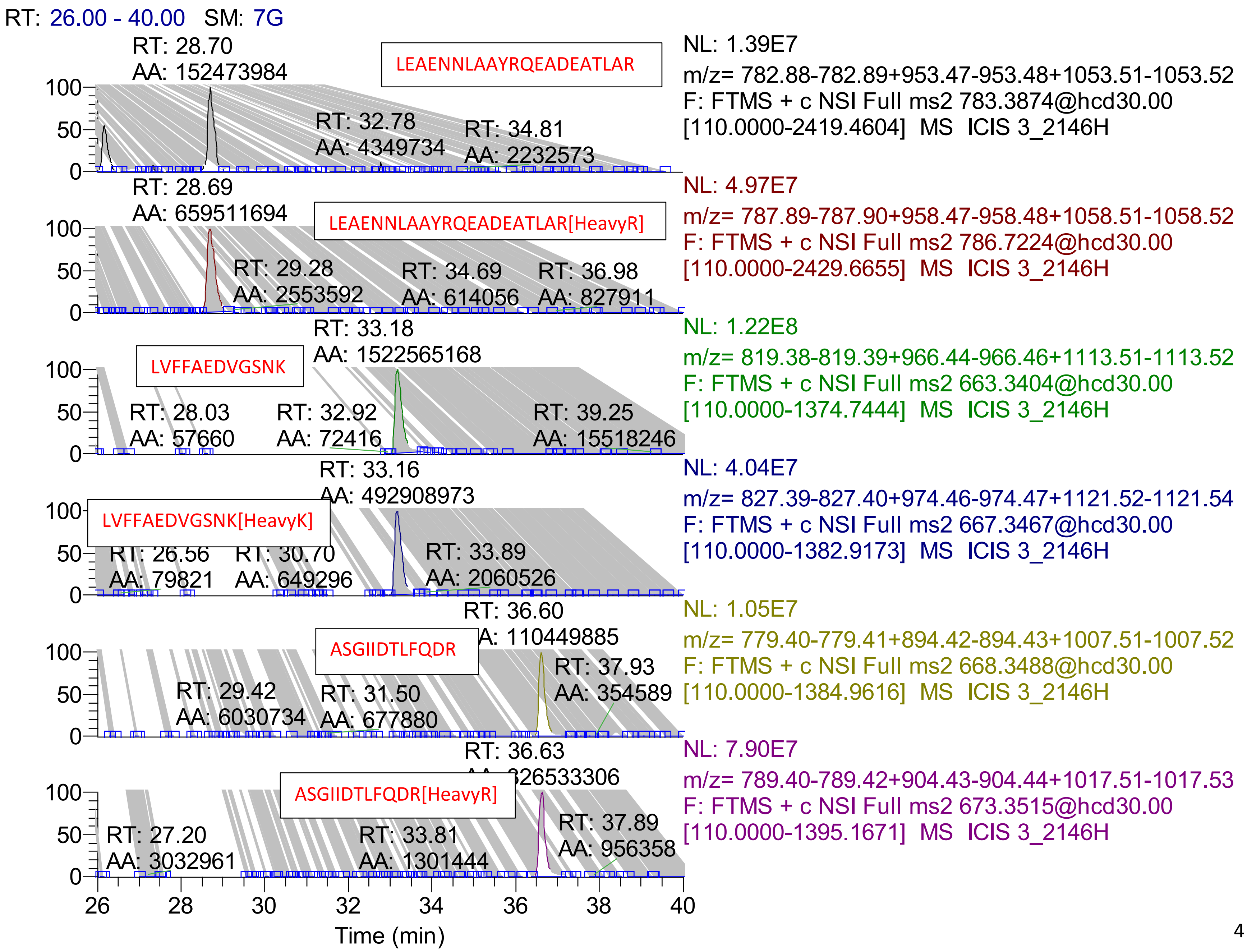
RT: 26.00 - 40.00 SM: 7G
LEAENNLAAYRQEADEATLAR
RT: 28.70
AA: 152473984
RT: 32.78
AA: 4349734
RT: 34.81
AA: 2232573
NL: 1.39E7
m/z= 782.88-782.89+953.47-953.48+1053.51-1053.52
F: FTMS + c NSI Full ms2 783.3874@hcd30.00
[110.0000-2419.4604] MS ICIS 3_2146H
LEAENNLAAYRQEADEATLAR[HeavyR]
RT: 28.69
AA: 659511694
RT: 29.28
AA: 2553592
RT: 34.69
AA: 614056
RT: 36.98
AA: 827911
NL: 4.97E7
m/z= 787.89-787.90+958.47-958.48+1058.51-1058.52
F: FTMS + c NSI Full ms2 786.7224@hcd30.00
[110.0000-2429.6655] MS ICIS 3_2146H
LVFFAEDVGSNK
RT: 33.18
AA: 1522565168
RT: 28.03
AA: 57660
RT: 32.92
AA: 72416
RT: 39.25
AA: 15518246
NL: 1.22E8
m/z= 819.38-819.39+966.44-966.46+1113.51-1113.52
F: FTMS + c NSI Full ms2 663.3404@hcd30.00
[110.0000-1374.7444] MS ICIS 3_2146H
LVFFAEDVGSNK[HeavyK]
RT: 33.16
AA: 492908973
RT: 26.56
AA: 79821
RT: 30.70
AA: 649296
RT: 33.89
AA: 2060526
NL: 4.04E7
m/z= 827.39-827.40+974.46-974.47+1121.52-1121.54
F: FTMS + c NSI Full ms2 667.3467@hcd30.00
[110.0000-1382.9173] MS ICIS 3_2146H
ASGIIDTLFQDR
RT: 36.60
A: 110449885
RT: 29.42
AA: 6030734
RT: 31.50
AA: 677880
RT: 37.93
AA: 354589
NL: 1.05E7
m/z= 779.40-779.41+894.42-894.43+1007.51-1007.52
F: FTMS + c NSI Full ms2 668.3488@hcd30.00
[110.0000-1384.9616] MS ICIS 3_2146H
ASGIIDTLFQDR[HeavyR]
RT: 36.63
326533306
RT: 27.20
AA: 3032961
RT: 33.81
AA: 1301444
RT: 37.89
AA: 956358
NL: 7.90E7
m/z= 789.40-789.42+904.43-904.44+1017.51-1017.53
F: FTMS + c NSI Full ms2 673.3515@hcd30.00
[110.0000-1395.1671] MS ICIS 3_2146H
100
50
0
26
28
30
32
34
36
38
40
Time (min)

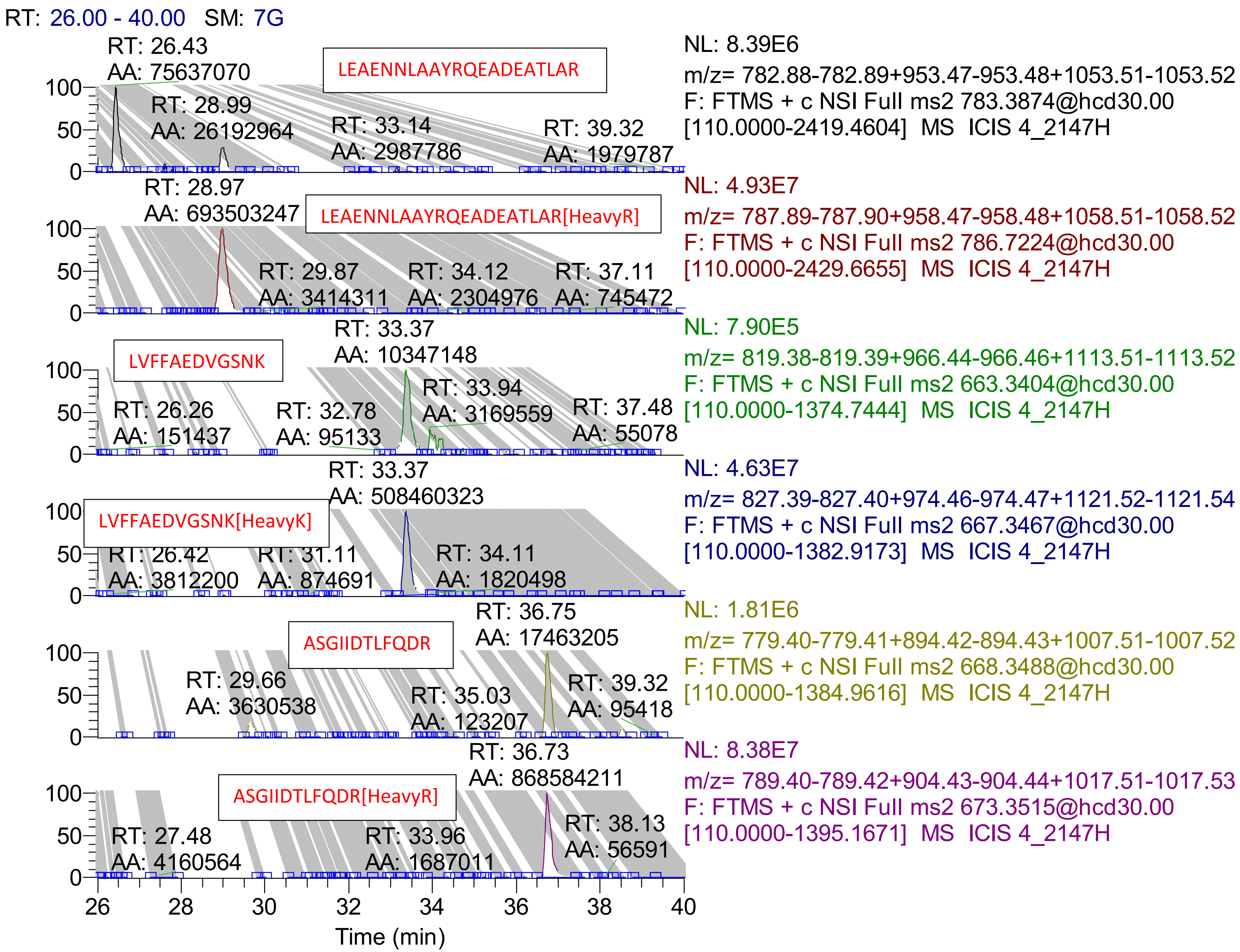
RT: 26.00 - 40.00 SM: 7G
LEAENNLAAYRQEADEATLAR
RT: 26.43 AA: 75637070
RT: 28.99 AA: 26192964
RT: 33.14 AA: 2987786
RT: 39.32 AA: 1979787
NL: 8.39E6
m/z= 782.88-782.89+953.47-953.48+1053.51-1053.52
F: FTMS + c NSI Full ms2 783.3874@hcd30.00
[110.0000-2419.4604] MS ICIS 4_2147H
LEAENNLAAYRQEADEATLAR[HeavyR]
RT: 28.97 AA: 693503247
RT: 29.87 AA: 3414311
RT: 34.12 AA: 2304976
RT: 37.11 AA: 745472
NL: 4.93E7
m/z= 787.89-787.90+958.47-958.48+1058.51-1058.52
F: FTMS + c NSI Full ms2 786.7224@hcd30.00
[110.0000-2429.6655] MS ICIS 4_2147H
LVFFAEDVGSNK
RT: 33.37 AA: 10347148
RT: 26.26 AA: 151437
RT: 32.78 AA: 95133
RT: 33.94 AA: 3169559
RT: 37.48 AA: 55078
NL: 7.90E5
m/z= 819.38-819.39+966.44-966.46+1113.51-1113.52
F: FTMS + c NSI Full ms2 663.3404@hcd30.00
[110.0000-1374.7444] MS ICIS 4_2147H
LVFFAEDVGSNK[HeavyK]
RT: 33.37 AA: 508460323
RT: 26.42 AA: 3812200
RT: 31.11 AA: 874691
RT: 34.11 AA: 1820498
NL: 4.63E7
m/z= 827.39-827.40+974.46-974.47+1121.52-1121.54
F: FTMS + c NSI Full ms2 667.3467@hcd30.00
[110.0000-1382.9173] MS ICIS 4_2147H
ASGIIDTLFQDR
RT: 36.75 AA: 17463205
RT: 29.66 AA: 3630538
RT: 35.03 AA: 123207
RT: 39.32 AA: 95418
NL: 1.81E6
m/z= 779.40-779.41+894.42-894.43+1007.51-1007.52
F: FTMS + c NSI Full ms2 668.3488@hcd30.00
[110.0000-1384.9616] MS ICIS 4_2147H
ASGIIDTLFQDR[HeavyR]
RT: 36.73 AA: 868584211
RT: 27.48 AA: 4160564
RT: 33.96 AA: 1687011
RT: 38.13 AA: 56591
NL: 8.38E7
m/z= 789.40-789.42+904.43-904.44+1017.51-1017.53
F: FTMS + c NSI Full ms2 673.3515@hcd30.00
[110.0000-1395.1671] MS ICIS 4_2147H
100
50
0
26 28 30 32 34 36 38 40
Time (min)

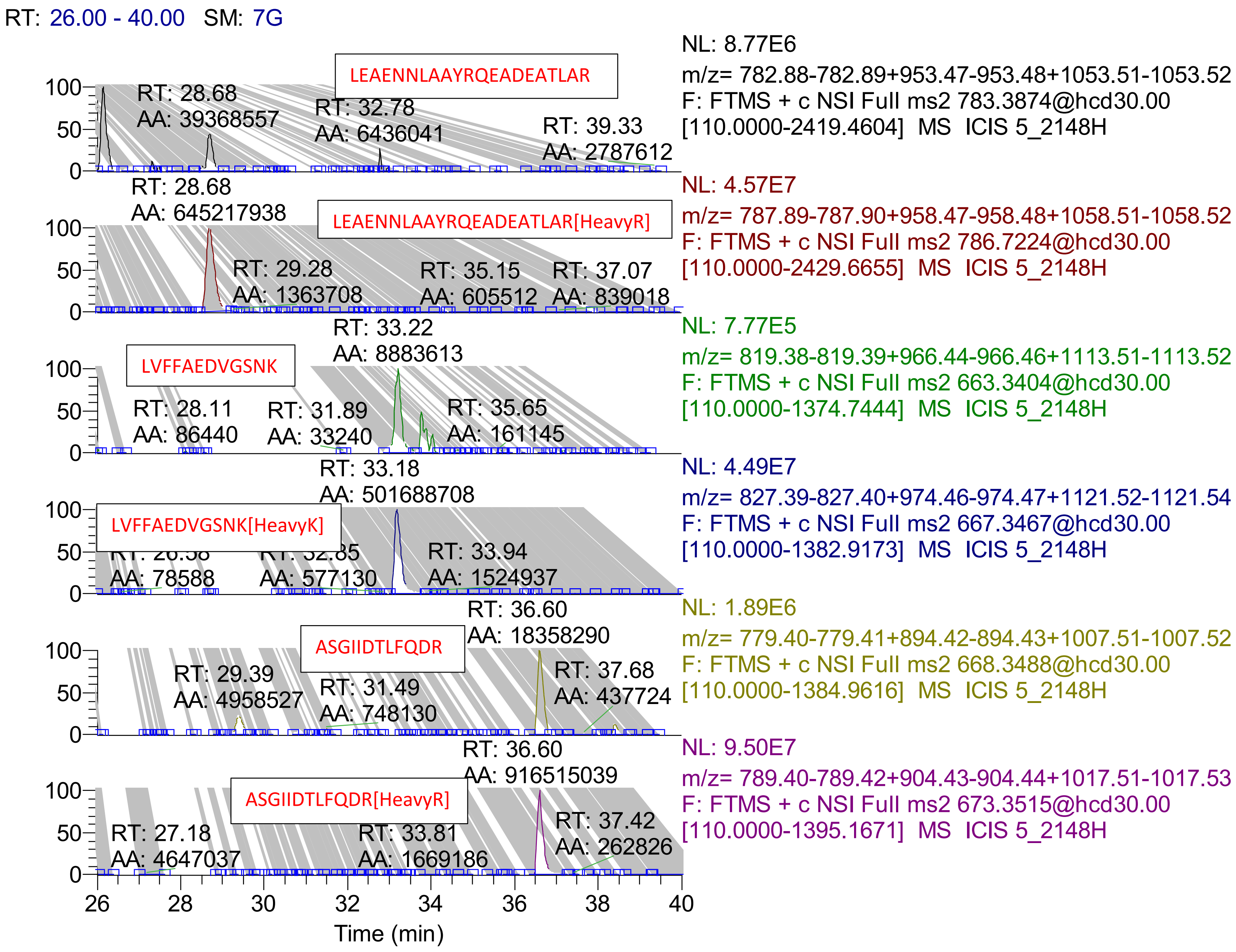

RT: 26.00 - 40.00 SM: 7G
LEAENNLAAYRQEADEATLAR
RT: 28.68
AA: 39368557
RT: 32.78
AA: 6436041
RT: 39.33
AA: 2787612
NL: 8.77E6
m/z= 782.88-782.89+953.47-953.48+1053.51-1053.52
F: FTMS + c NSI Full ms2 783.3874@hcd30.00
[110.0000-2419.4604] MS ICIS 5_2148H
RT: 28.68
AA: 645217938
LEAENNLAAYRQEADEATLAR[HeavyR]
RT: 29.28
AA: 1363708
RT: 35.15
AA: 605512
RT: 37.07
AA: 839018
NL: 4.57E7
m/z= 787.89-787.90+958.47-958.48+1058.51-1058.52
F: FTMS + c NSI Full ms2 786.7224@hcd30.00
[110.0000-2429.6655] MS ICIS 5_2148H
RT: 33.22
AA: 8883613
LVFFAEDVGSNK
RT: 28.11
AA: 86440
RT: 31.89
AA: 33240
RT: 35.65
AA: 161145
NL: 7.77E5
m/z= 819.38-819.39+966.44-966.46+1113.51-1113.52
F: FTMS + c NSI Full ms2 663.3404@hcd30.00
[110.0000-1374.7444] MS ICIS 5_2148H
RT: 33.18
AA: 501688708
LVFFAEDVGSNK[HeavyK]
AA: 78588
AA: 577130
RT: 33.94
AA: 1524937
NL: 4.49E7
m/z= 827.39-827.40+974.46-974.47+1121.52-1121.54
F: FTMS + c NSI Full ms2 667.3467@hcd30.00
[110.0000-1382.9173] MS ICIS 5_2148H
RT: 36.60
AA: 18358290
ASGIIDTLFQDR
RT: 29.39
AA: 4958527
RT: 31.49
AA: 748130
RT: 37.68
AA: 437724
NL: 1.89E6
m/z= 779.40-779.41+894.42-894.43+1007.51-1007.52
F: FTMS + c NSI Full ms2 668.3488@hcd30.00
[110.0000-1384.9616] MS ICIS 5_2148H
RT: 36.60
AA: 916515039
ASGIIDTLFQDR[HeavyR]
RT: 27.18
AA: 4647037
RT: 33.81
AA: 1669186
RT: 37.42
AA: 262826
NL: 9.50E7
m/z= 789.40-789.42+904.43-904.44+1017.51-1017.53
F: FTMS + c NSI Full ms2 673.3515@hcd30.00
[110.0000-1395.1671] MS ICIS 5_2148H
100
50
0
26
28
30
32
34
36
38
40
Time (min)

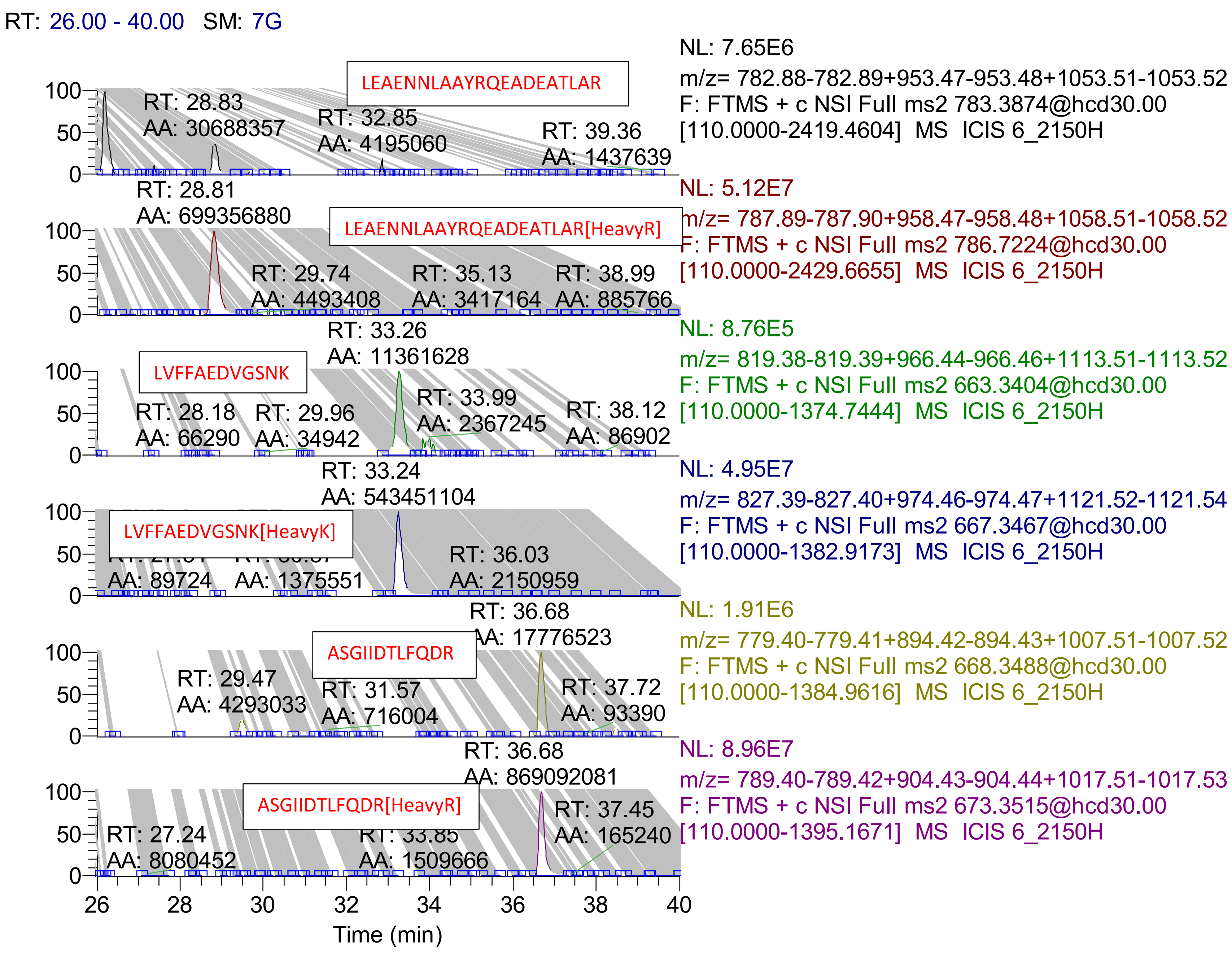
RT: 26.00 - 40.00 SM: 7G
LEAENNLAAYRQEADEATLAR
RT: 28.83
AA: 30688357
RT: 32.85
AA: 4195060
RT: 39.36
AA: 1437639
NL: 7.65E6
m/z= 782.88-782.89+953.47-953.48+1053.51-1053.52
F: FTMS + c NSI Full ms2 783.3874@hcd30.00
[110.0000-2419.4604] MS ICIS 6_2150H
LEAENNLAAYRQEADEATLAR[HeavyR]
RT: 28.81
AA: 699356880
RT: 29.74
AA: 4493408
RT: 35.13
AA: 3417164
RT: 38.99
AA: 885766
NL: 5.12E7
m/z= 787.89-787.90+958.47-958.48+1058.51-1058.52
F: FTMS + c NSI Full ms2 786.7224@hcd30.00
[110.0000-2429.6655] MS ICIS 6_2150H
LVFFAEDVGSNK
RT: 33.26
AA: 11361628
RT: 28.18
AA: 66290
RT: 29.96
AA: 34942
RT: 33.99
AA: 2367245
RT: 38.12
AA: 86902
NL: 8.76E5
m/z= 819.38-819.39+966.44-966.46+1113.51-1113.52
F: FTMS + c NSI Full ms2 663.3404@hcd30.00
[110.0000-1374.7444] MS ICIS 6_2150H
LVFFAEDVGSNK[HeavyK]
RT: 33.24
AA: 543451104
AA: 89724
AA: 1375551
RT: 36.03
AA: 2150959
NL: 4.95E7
m/z= 827.39-827.40+974.46-974.47+1121.52-1121.54
F: FTMS + c NSI Full ms2 667.3467@hcd30.00
[110.0000-1382.9173] MS ICIS 6_2150H
ASGIIDTLFQDR
RT: 36.68
RT: 29.47
AA: 4293033
RT: 31.57
AA: 716004
RT: 37.72
AA: 93390
NL: 1.91E6
m/z= 779.40-779.41+894.42-894.43+1007.51-1007.52
F: FTMS + c NSI Full ms2 668.3488@hcd30.00
[110.0000-1384.9616] MS ICIS 6_2150H
ASGIIDTLFQDR[HeavyR]
RT: 36.68
AA: 869092081
RT: 27.24
AA: 8080452
AA: 1509666
RT: 37.45
AA: 165240
NL: 8.96E7
m/z= 789.40-789.42+904.43-904.44+1017.51-1017.53
F: FTMS + c NSI Full ms2 673.3515@hcd30.00
[110.0000-1395.1671] MS ICIS 6_2150H
100
50
0
26
28
30
32
34
36
38
40
Time (min)

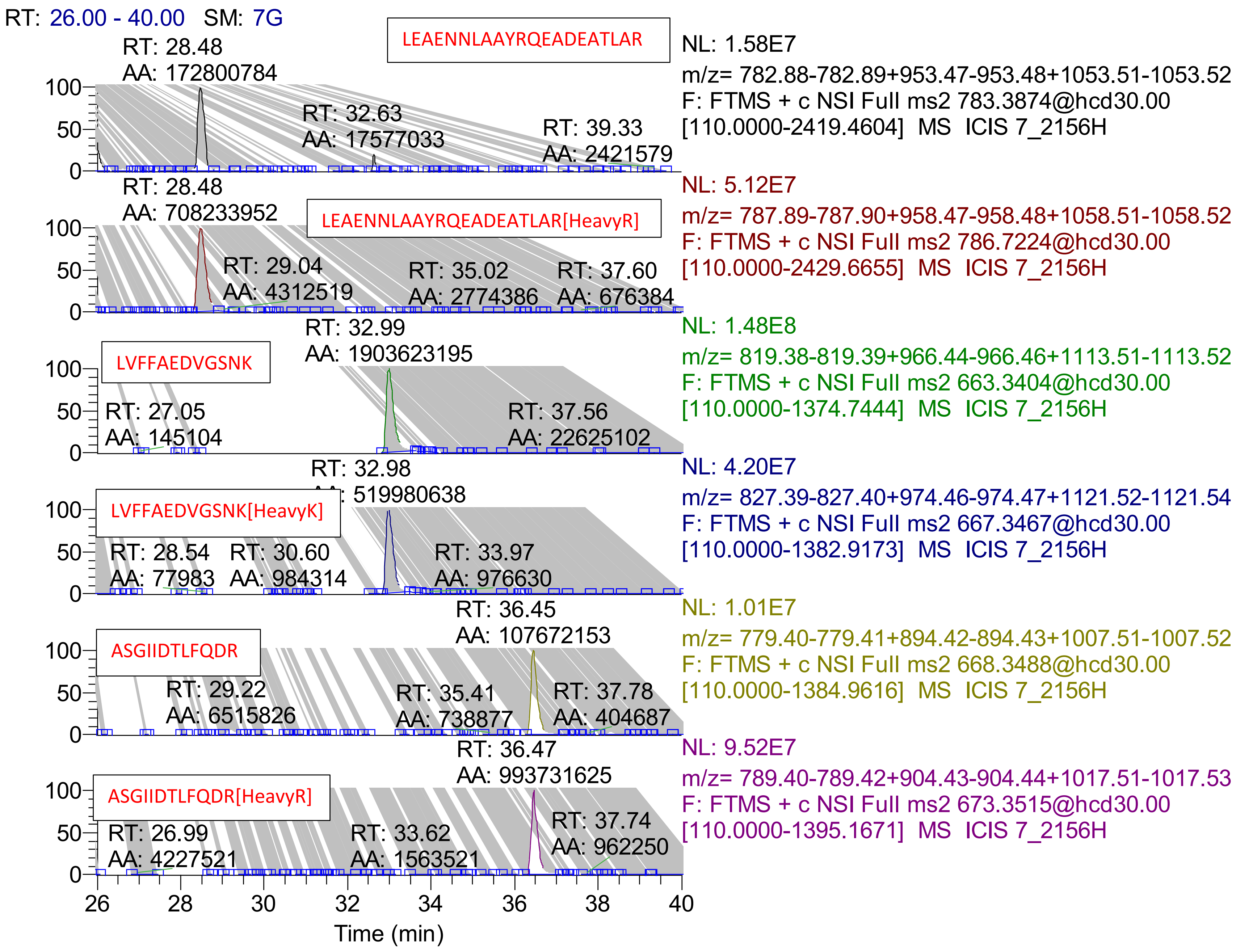
RT: 26.00 - 40.00 SM: 7G
LEAENNLAAYRQEADEATLAR
RT: 28.48
AA: 172800784
RT: 32.63
AA: 17577033
RT: 39.33
AA: 2421579
NL: 1.58E7
m/z= 782.88-782.89+953.47-953.48+1053.51-1053.52
F: FTMS + c NSI Full ms2 783.3874@hcd30.00
[110.0000-2419.4604] MS ICIS 7_2156H
LEAENNLAAYRQEADEATLAR[HeavyR]
RT: 28.48
AA: 708233952
RT: 29.04
AA: 4312519
RT: 35.02
AA: 2774386
RT: 37.60
AA: 676384
NL: 5.12E7
m/z= 787.89-787.90+958.47-958.48+1058.51-1058.52
F: FTMS + c NSI Full ms2 786.7224@hcd30.00
[110.0000-2429.6655] MS ICIS 7_2156H
LVFFAEDVGSNK
RT: 32.99
AA: 1903623195
RT: 27.05
AA: 145104
RT: 37.56
AA: 22625102
NL: 1.48E8
m/z= 819.38-819.39+966.44-966.46+1113.51-1113.52
F: FTMS + c NSI Full ms2 663.3404@hcd30.00
[110.0000-1374.7444] MS ICIS 7_2156H
LVFFAEDVGSNK[HeavyK]
RT: 32.98
: 519980638
RT: 28.54
AA: 77983
RT: 30.60
AA: 984314
RT: 33.97
AA: 976630
NL: 4.20E7
m/z= 827.39-827.40+974.46-974.47+1121.52-1121.54
F: FTMS + c NSI Full ms2 667.3467@hcd30.00
[110.0000-1382.9173] MS ICIS 7_2156H
ASGIIDTLFQDR
RT: 36.45
AA: 107672153
RT: 29.22
AA: 6515826
RT: 35.41
AA: 738877
RT: 37.78
AA: 404687
NL: 1.01E7
m/z= 779.40-779.41+894.42-894.43+1007.51-1007.52
F: FTMS + c NSI Full ms2 668.3488@hcd30.00
[110.0000-1384.9616] MS ICIS 7_2156H
ASGIIDTLFQDR[HeavyR]
RT: 36.47
AA: 993731625
RT: 26.99
AA: 4227521
RT: 33.62
AA: 1563521
RT: 37.74
AA: 962250
NL: 9.52E7
m/z= 789.40-789.42+904.43-904.44+1017.51-1017.53
F: FTMS + c NSI Full ms2 673.3515@hcd30.00
[110.0000-1395.1671] MS ICIS 7_2156H
100
50
0
26
28
30
32
34
36
38
40
Time (min)

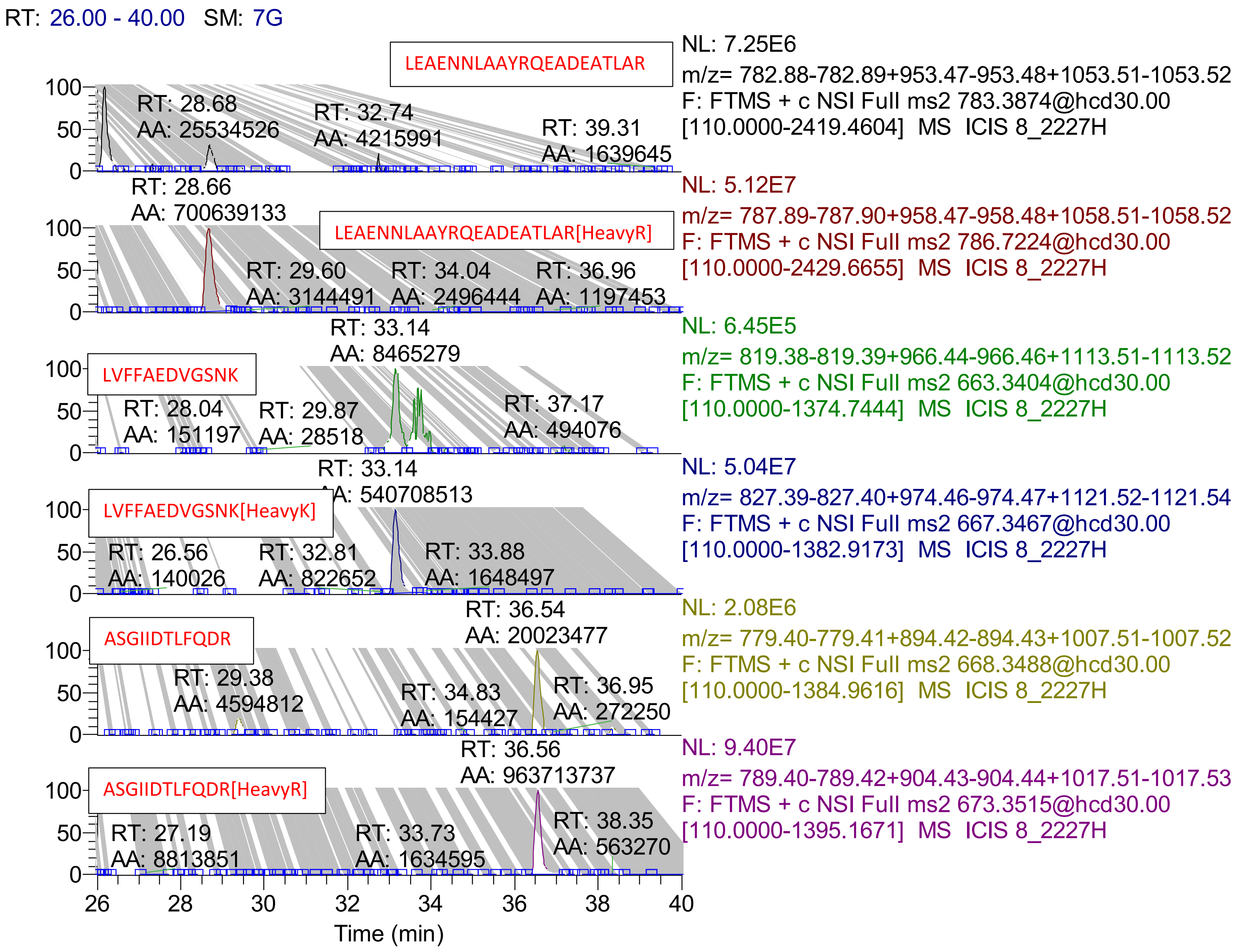

RT: 26.00 - 40.00 SM: 7G
LEAENNLAAYRQEADEATLAR
NL: 7.25E6
m/z= 782.88-782.89+953.47-953.48+1053.51-1053.52
F: FTMS + c NSI Full ms2 783.3874@hcd30.00
[110.0000-2419.4604] MS ICIS 8_2227H
RT: 28.68
AA: 25534526
RT: 32.74
AA: 4215991
RT: 39.31
AA: 1639645
RT: 28.66
AA: 700639133
LEAENNLAAYRQEADEATLAR[HeavyR]
NL: 5.12E7
m/z= 787.89-787.90+958.47-958.48+1058.51-1058.52
F: FTMS + c NSI Full ms2 786.7224@hcd30.00
[110.0000-2429.6655] MS ICIS 8_2227H
RT: 29.60
AA: 3144491
RT: 34.04
AA: 2496444
RT: 36.96
AA: 1197453
LVFFAEDVGSNK
NL: 6.45E5
m/z= 819.38-819.39+966.44-966.46+1113.51-1113.52
F: FTMS + c NSI Full ms2 663.3404@hcd30.00
[110.0000-1374.7444] MS ICIS 8_2227H
RT: 33.14
AA: 8465279
RT: 28.04
AA: 151197
RT: 29.87
AA: 28518
RT: 37.17
AA: 494076
LVFFAEDVGSNK[HeavyK]
NL: 5.04E7
m/z= 827.39-827.40+974.46-974.47+1121.52-1121.54
F: FTMS + c NSI Full ms2 667.3467@hcd30.00
[110.0000-1382.9173] MS ICIS 8_2227H
RT: 33.14
AA: 540708513
RT: 26.56
AA: 140026
RT: 32.81
AA: 822652
RT: 33.88
AA: 1648497
ASGIIDTLFQDR
NL: 2.08E6
m/z= 779.40-779.41+894.42-894.43+1007.51-1007.52
F: FTMS + c NSI Full ms2 668.3488@hcd30.00
[110.0000-1384.9616] MS ICIS 8_2227H
RT: 36.54
AA: 20023477
RT: 29.38
AA: 4594812
RT: 34.83
AA: 154427
RT: 36.95
AA: 272250
ASGIIDTLFQDR[HeavyR]
NL: 9.40E7
m/z= 789.40-789.42+904.43-904.44+1017.51-1017.53
F: FTMS + c NSI Full ms2 673.3515@hcd30.00
[110.0000-1395.1671] MS ICIS 8_2227H
RT: 36.56
AA: 963713737
RT: 27.19
AA: 8813851
RT: 33.73
AA: 1634595
RT: 38.35
AA: 563270
100
50
0
26
28
30
32
34
36
38
40
Time (min)

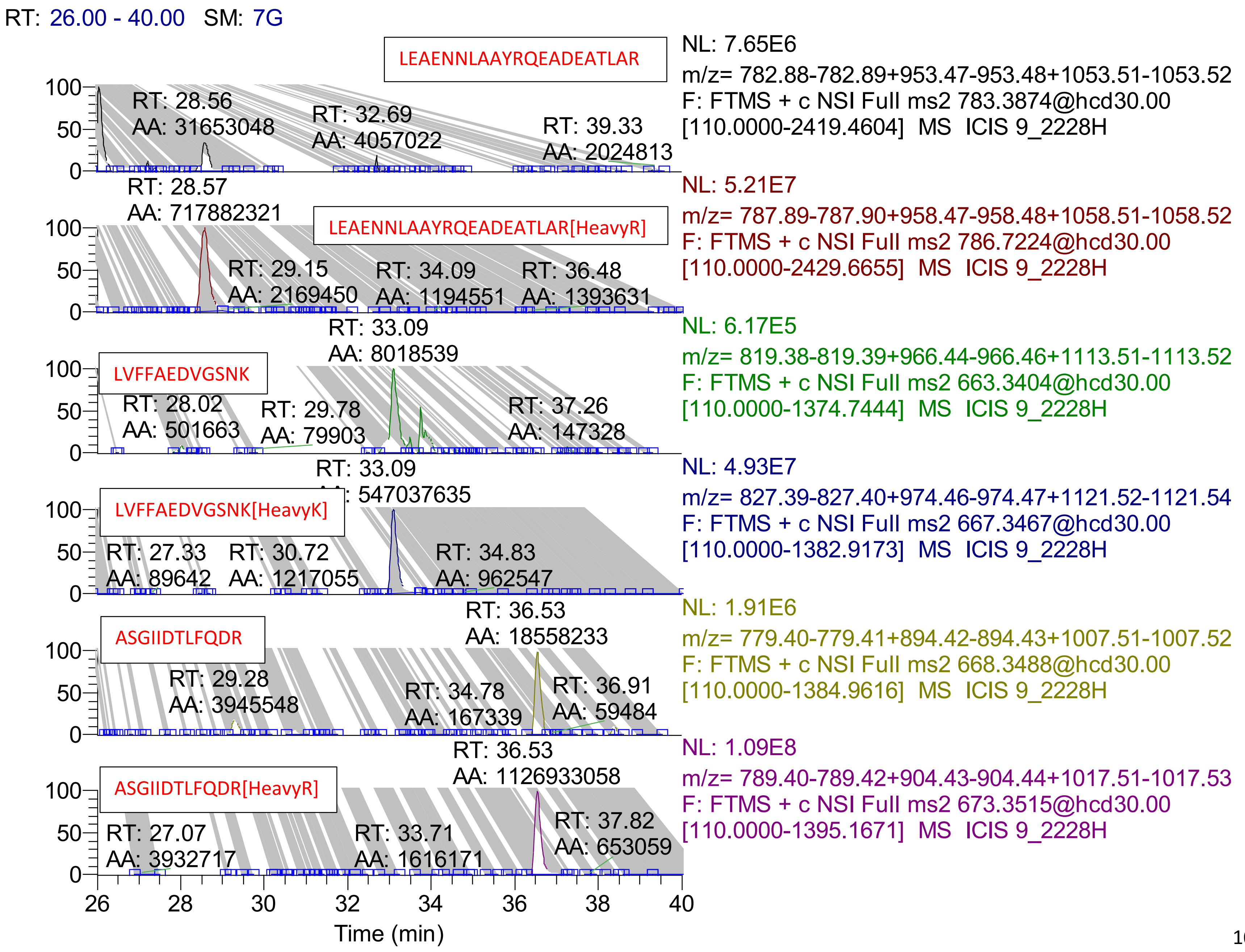
RT: 26.00 - 40.00 SM: 7G
LEAENNLAAYRQEADEATLAR
RT: 28.56 AA: 31653048
RT: 32.69 AA: 4057022
RT: 39.33 AA: 2024813
NL: 7.65E6
m/z= 782.88-782.89+953.47-953.48+1053.51-1053.52
F: FTMS + c NSI Full ms2 783.3874@hcd30.00
[110.0000-2419.4604] MS ICIS 9_2228H
RT: 28.57 AA: 717882321
LEAENNLAAYRQEADEATLAR[HeavyR]
RT: 29.15 AA: 2169450
RT: 34.09 AA: 1194551
RT: 36.48 AA: 1393631
NL: 5.21E7
m/z= 787.89-787.90+958.47-958.48+1058.51-1058.52
F: FTMS + c NSI Full ms2 786.7224@hcd30.00
[110.0000-2429.6655] MS ICIS 9_2228H
RT: 33.09 AA: 8018539
LVFFAEDVGSNK
RT: 28.02 AA: 501663
RT: 29.78 AA: 79903
RT: 37.26 AA: 147328
NL: 6.17E5
m/z= 819.38-819.39+966.44-966.46+1113.51-1113.52
F: FTMS + c NSI Full ms2 663.3404@hcd30.00
[110.0000-1374.7444] MS ICIS 9_2228H
RT: 33.09 AA: 547037635
LVFFAEDVGSNK[HeavyK]
RT: 27.33 AA: 89642
RT: 30.72 AA: 1217055
RT: 34.83 AA: 962547
NL: 4.93E7
m/z= 827.39-827.40+974.46-974.47+1121.52-1121.54
F: FTMS + c NSI Full ms2 667.3467@hcd30.00
[110.0000-1382.9173] MS ICIS 9_2228H
RT: 36.53 AA: 18558233
ASGIIDTLFQDR
RT: 29.28 AA: 3945548
RT: 34.78 AA: 167339
RT: 36.91 AA: 59484
NL: 1.91E6
m/z= 779.40-779.41+894.42-894.43+1007.51-1007.52
F: FTMS + c NSI Full ms2 668.3488@hcd30.00
[110.0000-1384.9616] MS ICIS 9_2228H
RT: 36.53 AA: 1126933058
ASGIIDTLFQDR[HeavyR]
RT: 27.07 AA: 3932717
RT: 33.71 AA: 1616171
RT: 37.82 AA: 653059
NL: 1.09E8
m/z= 789.40-789.42+904.43-904.44+1017.51-1017.53
F: FTMS + c NSI Full ms2 673.3515@hcd30.00
[110.0000-1395.1671] MS ICIS 9_2228H
0
50
100
26
28
30
32
34
36
38
40
Time (min)

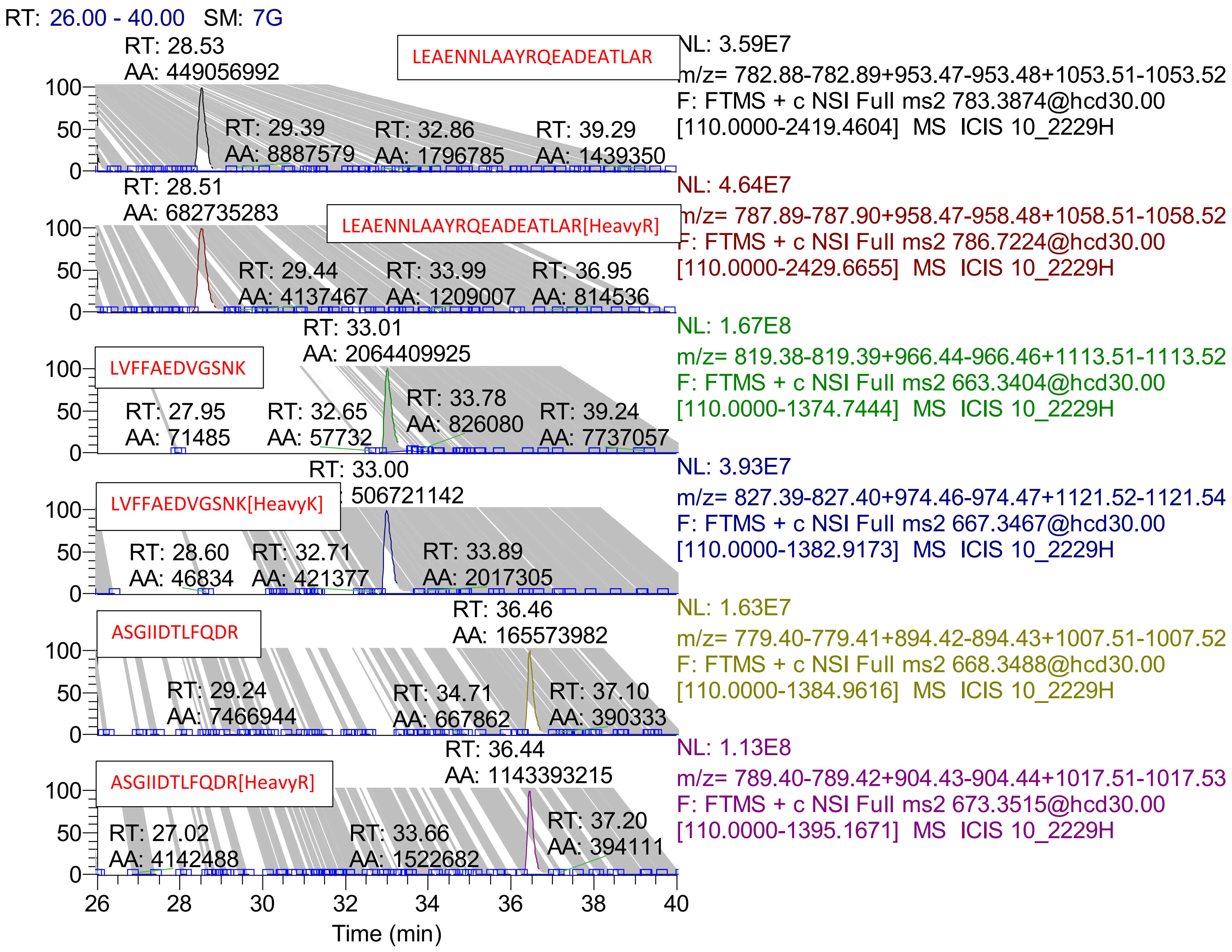
RT: 26.00 - 40.00 SM: 7G
LEAENNLAAYRQEADEATLAR
RT: 28.53
AA: 449056992
RT: 29.39
AA: 8887579
RT: 32.86
AA: 1796785
RT: 39.29
AA: 1439350
NL: 3.59E7
m/z= 782.88-782.89+953.47-953.48+1053.51-1053.52
F: FTMS + c NSI Full ms2 783.3874@hcd30.00
[110.0000-2419.4604] MS ICIS 10_2229H
LEAENNLAAYRQEADEATLAR[HeavyR]
RT: 28.51
AA: 682735283
RT: 29.44
AA: 4137467
RT: 33.99
AA: 1209007
RT: 36.95
AA: 814536
NL: 4.64E7
m/z= 787.89-787.90+958.47-958.48+1058.51-1058.52
F: FTMS + c NSI Full ms2 786.7224@hcd30.00
[110.0000-2429.6655] MS ICIS 10_2229H
LVFFAEDVGSNK
RT: 33.01
AA: 2064409925
RT: 27.95
AA: 71485
RT: 32.65
AA: 57732
RT: 33.78
AA: 826080
RT: 39.24
AA: 7737057
NL: 1.67E8
m/z= 819.38-819.39+966.44-966.46+1113.51-1113.52
F: FTMS + c NSI Full ms2 663.3404@hcd30.00
[110.0000-1374.7444] MS ICIS 10_2229H
LVFFAEDVGSNK[HeavyK]
RT: 33.00
506721142
RT: 28.60
AA: 46834
RT: 32.71
AA: 421377
RT: 33.89
AA: 2017305
NL: 3.93E7
m/z= 827.39-827.40+974.46-974.47+1121.52-1121.54
F: FTMS + c NSI Full ms2 667.3467@hcd30.00
[110.0000-1382.9173] MS ICIS 10_2229H
ASGIIDTLFQDR
RT: 36.46
AA: 165573982
RT: 29.24
AA: 7466944
RT: 34.71
AA: 667862
RT: 37.10
AA: 390333
NL: 1.63E7
m/z= 779.40-779.41+894.42-894.43+1007.51-1007.52
F: FTMS + c NSI Full ms2 668.3488@hcd30.00
[110.0000-1384.9616] MS ICIS 10_2229H
ASGIIDTLFQDR[HeavyR]
RT: 36.44
AA: 1143393215
RT: 27.02
AA: 4142488
RT: 33.66
AA: 1522682
RT: 37.20
AA: 394111
NL: 1.13E8
m/z= 789.40-789.42+904.43-904.44+1017.51-1017.53
F: FTMS + c NSI Full ms2 673.3515@hcd30.00
[110.0000-1395.1671] MS ICIS 10_2229H
100
50
0
26
28
30
32
34
36
38
40
Time (min)

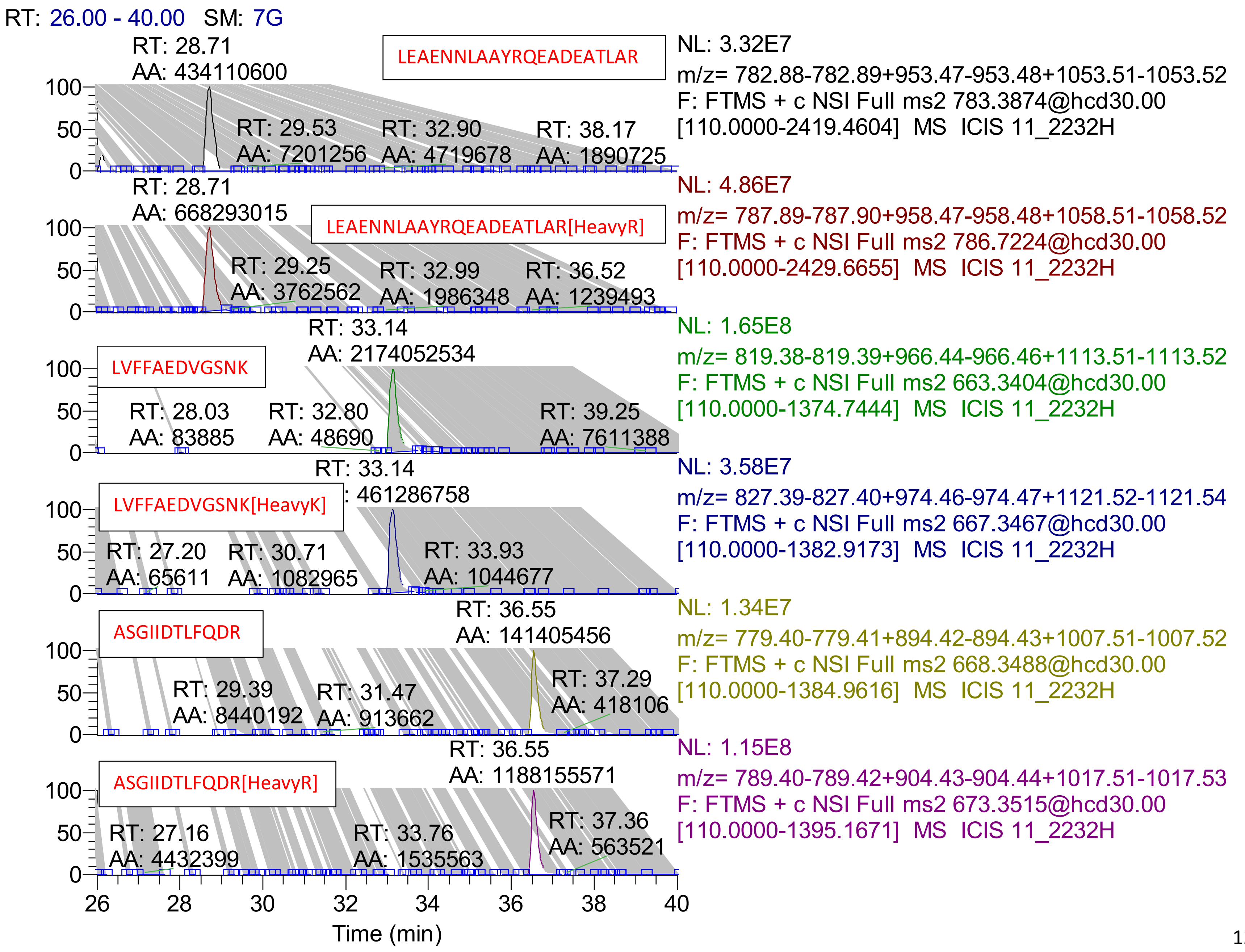
RT: 26.00 - 40.00 SM: 7G
LEAENNLAAYRQEADEATLAR
RT: 28.71
AA: 434110600
RT: 29.53
AA: 7201256
RT: 32.90
AA: 4719678
RT: 38.17
AA: 1890725
NL: 3.32E7
m/z= 782.88-782.89+953.47-953.48+1053.51-1053.52
F: FTMS + c NSI Full ms2 783.3874@hcd30.00
[110.0000-2419.4604] MS ICIS 11_2232H
LEAENNLAAYRQEADEATLAR[HeavyR]
RT: 28.71
AA: 668293015
RT: 29.25
AA: 3762562
RT: 32.99
AA: 1986348
RT: 36.52
AA: 1239493
NL: 4.86E7
m/z= 787.89-787.90+958.47-958.48+1058.51-1058.52
F: FTMS + c NSI Full ms2 786.7224@hcd30.00
[110.0000-2429.6655] MS ICIS 11_2232H
LVFFAEDVGSNK
RT: 33.14
AA: 2174052534
RT: 28.03
AA: 83885
RT: 32.80
AA: 48690
RT: 39.25
AA: 7611388
NL: 1.65E8
m/z= 819.38-819.39+966.44-966.46+1113.51-1113.52
F: FTMS + c NSI Full ms2 663.3404@hcd30.00
[110.0000-1374.7444] MS ICIS 11_2232H
LVFFAEDVGSNK[HeavyK]
RT: 33.14
: 461286758
RT: 27.20
AA: 65611
RT: 30.71
AA: 1082965
RT: 33.93
AA: 1044677
NL: 3.58E7
m/z= 827.39-827.40+974.46-974.47+1121.52-1121.54
F: FTMS + c NSI Full ms2 667.3467@hcd30.00
[110.0000-1382.9173] MS ICIS 11_2232H
ASGIIDTLFQDR
RT: 36.55
AA: 141405456
RT: 29.39
AA: 8440192
RT: 31.47
AA: 913662
RT: 37.29
AA: 418106
NL: 1.34E7
m/z= 779.40-779.41+894.42-894.43+1007.51-1007.52
F: FTMS + c NSI Full ms2 668.3488@hcd30.00
[110.0000-1384.9616] MS ICIS 11_2232H
ASGIIDTLFQDR[HeavyR]
RT: 36.55
AA: 1188155571
RT: 27.16
AA: 4432399
RT: 33.76
AA: 1535563
RT: 37.36
AA: 563521
NL: 1.15E8
m/z= 789.40-789.42+904.43-904.44+1017.51-1017.53
F: FTMS + c NSI Full ms2 673.3515@hcd30.00
[110.0000-1395.1671] MS ICIS 11_2232H
100
50
0
26
28
30
32
34
36
38
40
Time (min)

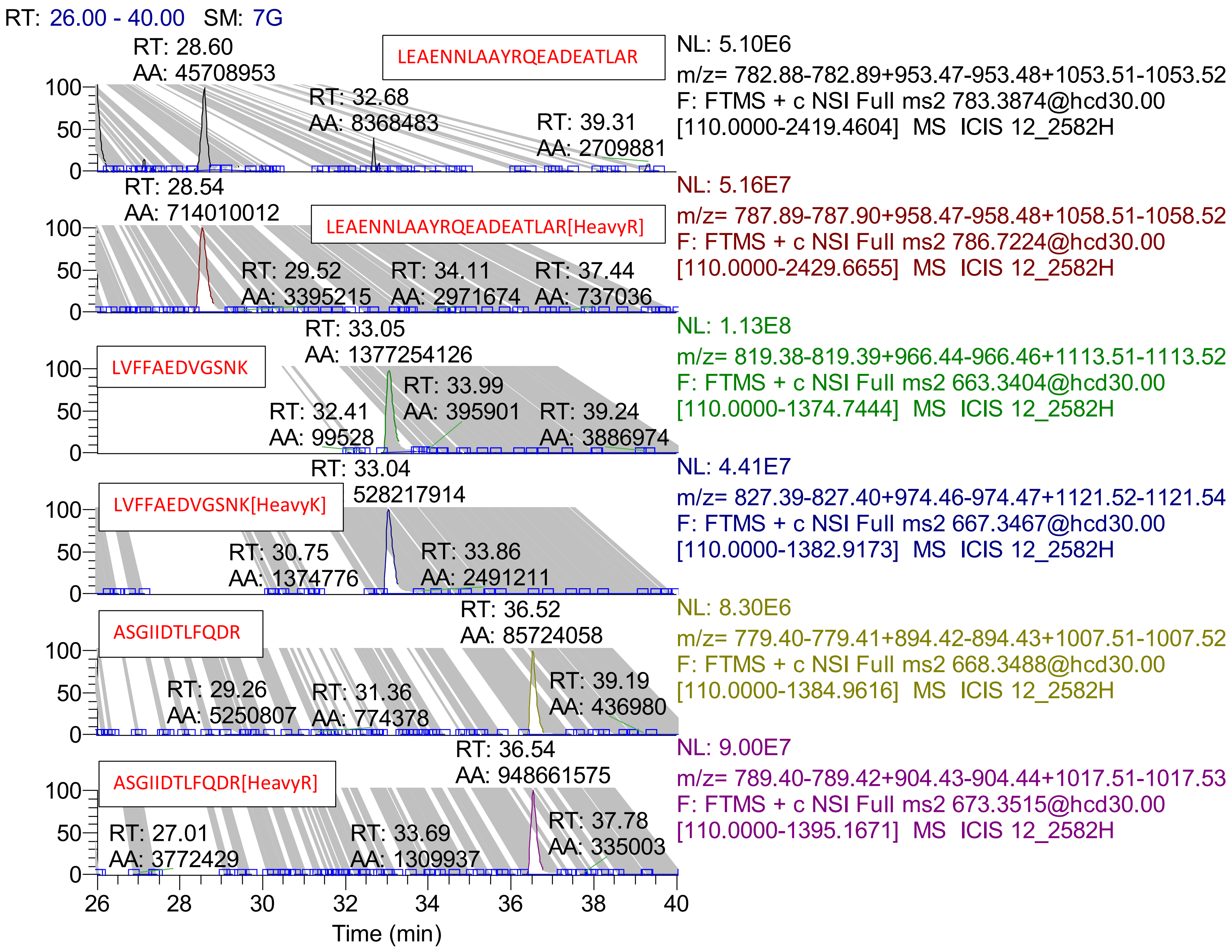
RT: 26.00 - 40.00 SM: 7G
LEAENNLAAYRQEADEATLAR
RT: 28.60
AA: 45708953
RT: 32.68
AA: 8368483
RT: 39.31
AA: 2709881
NL: 5.10E6
m/z= 782.88-782.89+953.47-953.48+1053.51-1053.52
F: FTMS + c NSI Full ms2 783.3874@hcd30.00
[110.0000-2419.4604] MS ICIS 12_2582H
LEAENNLAAYRQEADEATLAR[HeavyR]
RT: 28.54
AA: 714010012
RT: 29.52
AA: 3395215
RT: 34.11
AA: 2971674
RT: 37.44
AA: 737036
NL: 5.16E7
m/z= 787.89-787.90+958.47-958.48+1058.51-1058.52
F: FTMS + c NSI Full ms2 786.7224@hcd30.00
[110.0000-2429.6655] MS ICIS 12_2582H
LVFFAEDVGSNK
RT: 33.05
AA: 1377254126
RT: 32.41
AA: 99528
RT: 33.99
AA: 395901
RT: 39.24
AA: 3886974
NL: 1.13E8
m/z= 819.38-819.39+966.44-966.46+1113.51-1113.52
F: FTMS + c NSI Full ms2 663.3404@hcd30.00
[110.0000-1374.7444] MS ICIS 12_2582H
LVFFAEDVGSNK[HeavyK]
RT: 33.04
528217914
RT: 30.75
AA: 1374776
RT: 33.86
AA: 2491211
NL: 4.41E7
m/z= 827.39-827.40+974.46-974.47+1121.52-1121.54
F: FTMS + c NSI Full ms2 667.3467@hcd30.00
[110.0000-1382.9173] MS ICIS 12_2582H
ASGIIDTLFQDR
RT: 36.52
AA: 85724058
RT: 29.26
AA: 5250807
RT: 31.36
AA: 774378
RT: 39.19
AA: 436980
NL: 8.30E6
m/z= 779.40-779.41+894.42-894.43+1007.51-1007.52
F: FTMS + c NSI Full ms2 668.3488@hcd30.00
[110.0000-1384.9616] MS ICIS 12_2582H
ASGIIDTLFQDR[HeavyR]
RT: 36.54
AA: 948661575
RT: 27.01
AA: 3772429
RT: 33.69
AA: 1309937
RT: 37.78
AA: 335003
NL: 9.00E7
m/z= 789.40-789.42+904.43-904.44+1017.51-1017.53
F: FTMS + c NSI Full ms2 673.3515@hcd30.00
[110.0000-1395.1671] MS ICIS 12_2582H
100
50
0
26
28
30
32
34
36
38
40
Time (min)

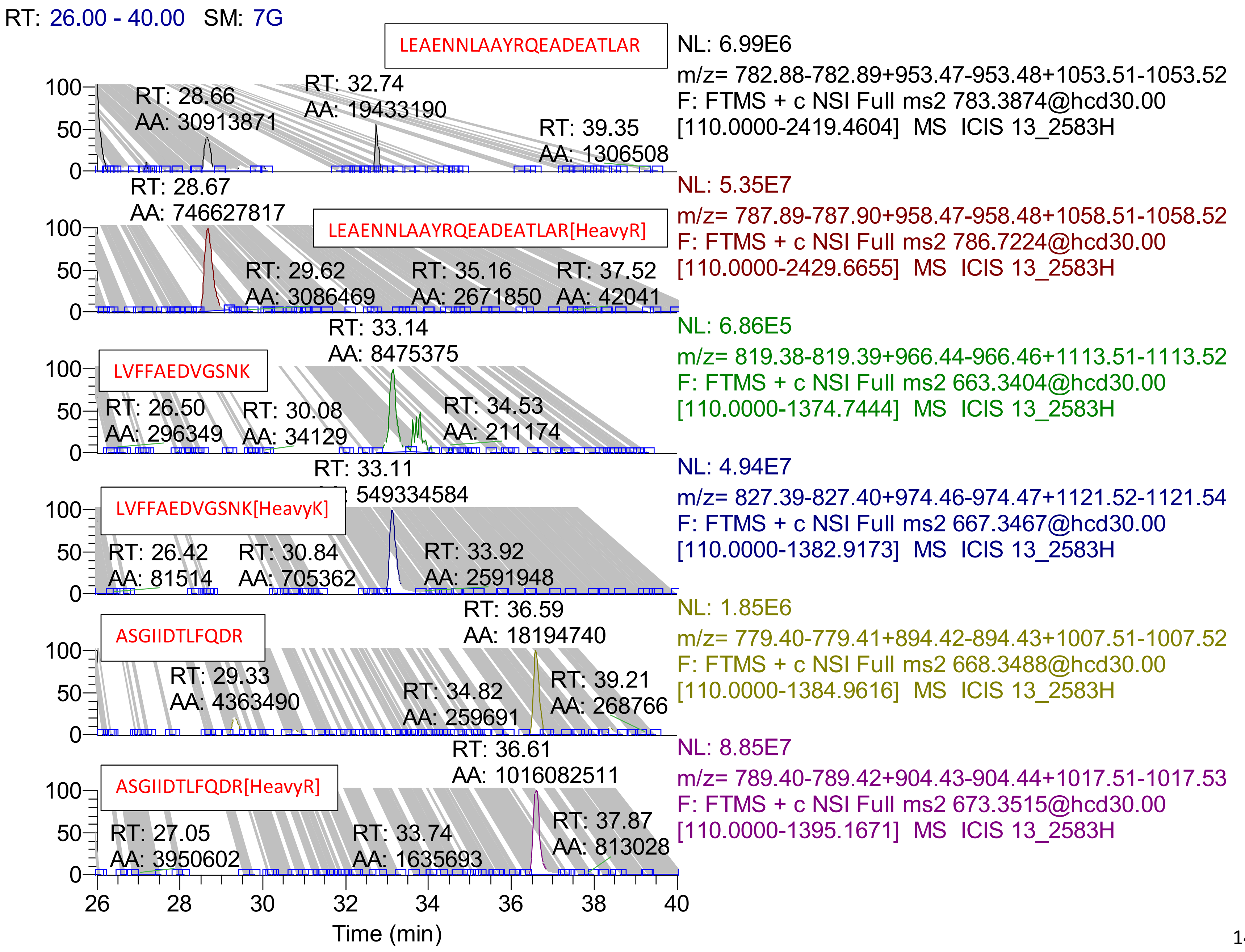
RT: 26.00 - 40.00 SM: 7G
LEAENNLAAYRQEADEATLAR
NL: 6.99E6
m/z= 782.88-782.89+953.47-953.48+1053.51-1053.52
F: FTMS + c NSI Full ms2 783.3874@hcd30.00
[110.0000-2419.4604] MS ICIS 13_2583H
RT: 28.66
AA: 30913871
RT: 32.74
AA: 19433190
RT: 39.35
AA: 1306508
LEAENNLAAYRQEADEATLAR[HeavyR]
NL: 5.35E7
m/z= 787.89-787.90+958.47-958.48+1058.51-1058.52
F: FTMS + c NSI Full ms2 786.7224@hcd30.00
[110.0000-2429.6655] MS ICIS 13_2583H
RT: 28.67
AA: 746627817
RT: 29.62
AA: 3086469
RT: 35.16
AA: 2671850
RT: 37.52
AA: 42041
LVFFAEDVGSNK
NL: 6.86E5
m/z= 819.38-819.39+966.44-966.46+1113.51-1113.52
F: FTMS + c NSI Full ms2 663.3404@hcd30.00
[110.0000-1374.7444] MS ICIS 13_2583H
RT: 33.14
AA: 8475375
RT: 26.50
AA: 296349
RT: 30.08
AA: 34129
RT: 34.53
AA: 211174
LVFFAEDVGSNK[HeavyK]
NL: 4.94E7
m/z= 827.39-827.40+974.46-974.47+1121.52-1121.54
F: FTMS + c NSI Full ms2 667.3467@hcd30.00
[110.0000-1382.9173] MS ICIS 13_2583H
RT: 33.11
549334584
RT: 26.42
AA: 81514
RT: 30.84
AA: 705362
RT: 33.92
AA: 2591948
ASGIIDTLFQDR
NL: 1.85E6
m/z= 779.40-779.41+894.42-894.43+1007.51-1007.52
F: FTMS + c NSI Full ms2 668.3488@hcd30.00
[110.0000-1384.9616] MS ICIS 13_2583H
RT: 36.59
AA: 18194740
RT: 29.33
AA: 4363490
RT: 34.82
AA: 259691
RT: 39.21
AA: 268766
ASGIIDTLFQDR[HeavyR]
NL: 8.85E7
m/z= 789.40-789.42+904.43-904.44+1017.51-1017.53
F: FTMS + c NSI Full ms2 673.3515@hcd30.00
[110.0000-1395.1671] MS ICIS 13_2583H
RT: 36.61
AA: 1016082511
RT: 27.05
AA: 3950602
RT: 33.74
AA: 1635693
RT: 37.87
AA: 813028
100
50
0
26
28
30
32
34
36
38
40
Time (min)

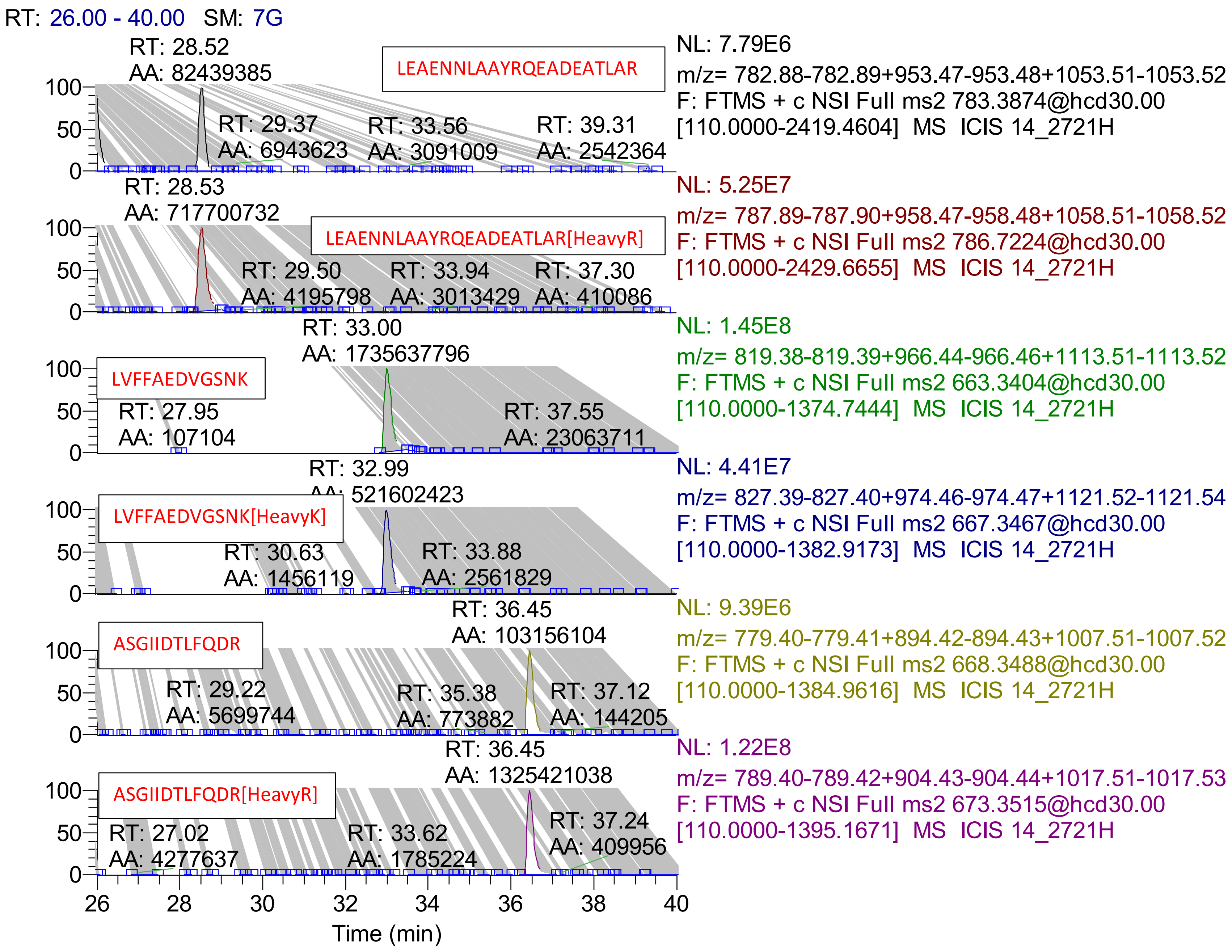

RT: 26.00 - 40.00 SM: 7G
RT: 28.52
AA: 82439385
LEAENNLAAYRQEADEATLAR
RT: 29.37
AA: 6943623
RT: 33.56
AA: 3091009
RT: 39.31
AA: 2542364
NL: 7.79E6
m/z= 782.88-782.89+953.47-953.48+1053.51-1053.52
F: FTMS + c NSI Full ms2 783.3874@hcd30.00
[110.0000-2419.4604] MS ICIS 14_2721H
RT: 28.53
AA: 717700732
LEAENNLAAYRQEADEATLAR[HeavyR]
RT: 29.50
AA: 4195798
RT: 33.94
AA: 3013429
RT: 37.30
AA: 410086
NL: 5.25E7
m/z= 787.89-787.90+958.47-958.48+1058.51-1058.52
F: FTMS + c NSI Full ms2 786.7224@hcd30.00
[110.0000-2429.6655] MS ICIS 14_2721H
RT: 33.00
AA: 1735637796
LVFFAEDVGSNK
RT: 27.95
AA: 107104
RT: 37.55
AA: 23063711
NL: 1.45E8
m/z= 819.38-819.39+966.44-966.46+1113.51-1113.52
F: FTMS + c NSI Full ms2 663.3404@hcd30.00
[110.0000-1374.7444] MS ICIS 14_2721H
RT: 32.99
AA: 521602423
LVFFAEDVGSNK[HeavyK]
RT: 30.63
AA: 1456119
RT: 33.88
AA: 2561829
NL: 4.41E7
m/z= 827.39-827.40+974.46-974.47+1121.52-1121.54
F: FTMS + c NSI Full ms2 667.3467@hcd30.00
[110.0000-1382.9173] MS ICIS 14_2721H
RT: 36.45
AA: 103156104
ASGIIDTLFQDR
RT: 29.22
AA: 5699744
RT: 35.38
AA: 773882
RT: 37.12
AA: 144205
NL: 9.39E6
m/z= 779.40-779.41+894.42-894.43+1007.51-1007.52
F: FTMS + c NSI Full ms2 668.3488@hcd30.00
[110.0000-1384.9616] MS ICIS 14_2721H
RT: 36.45
AA: 1325421038
ASGIIDTLFQDR[HeavyR]
RT: 27.02
AA: 4277637
RT: 33.62
AA: 1785224
RT: 37.24
AA: 409956
NL: 1.22E8
m/z= 789.40-789.42+904.43-904.44+1017.51-1017.53
F: FTMS + c NSI Full ms2 673.3515@hcd30.00
[110.0000-1395.1671] MS ICIS 14_2721H
100
50
0
26 28 30 32 34 36 38 40
Time (min)

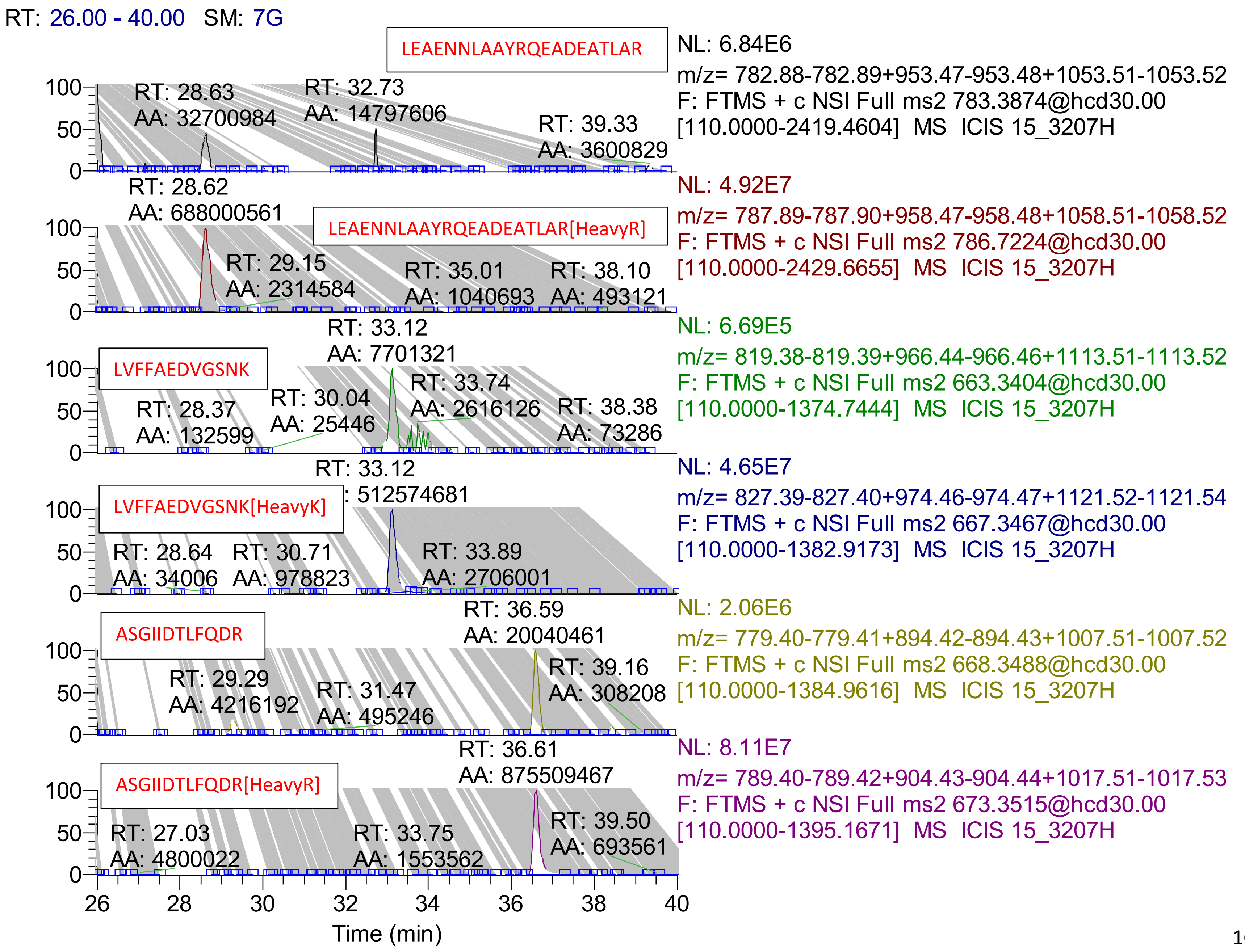

RT: 26.00 - 40.00 SM: 7G
LEAENNLAAYRQEADEATLAR
RT: 28.63 AA: 32700984
RT: 32.73 AA: 14797606
RT: 39.33 AA: 3600829
NL: 6.84E6
m/z= 782.88-782.89+953.47-953.48+1053.51-1053.52
F: FTMS + c NSI Full ms2 783.3874@hcd30.00
[110.0000-2419.4604] MS ICIS 15_3207H
LEAENNLAAYRQEADEATLAR[HeavyR]
RT: 28.62 AA: 688000561
RT: 29.15 AA: 2314584
RT: 35.01 AA: 1040693
RT: 38.10 AA: 493121
NL: 4.92E7
m/z= 787.89-787.90+958.47-958.48+1058.51-1058.52
F: FTMS + c NSI Full ms2 786.7224@hcd30.00
[110.0000-2429.6655] MS ICIS 15_3207H
LVFFAEDVGSNK
RT: 33.12 AA: 7701321
RT: 28.37 AA: 132599
RT: 30.04 AA: 25446
RT: 33.74 AA: 2616126
RT: 38.38 AA: 73286
NL: 6.69E5
m/z= 819.38-819.39+966.44-966.46+1113.51-1113.52
F: FTMS + c NSI Full ms2 663.3404@hcd30.00
[110.0000-1374.7444] MS ICIS 15_3207H
LVFFAEDVGSNK[HeavyK]
RT: 33.12 : 512574681
RT: 28.64 AA: 34006
RT: 30.71 AA: 978823
RT: 33.89 AA: 2706001
NL: 4.65E7
m/z= 827.39-827.40+974.46-974.47+1121.52-1121.54
F: FTMS + c NSI Full ms2 667.3467@hcd30.00
[110.0000-1382.9173] MS ICIS 15_3207H
ASGIIDTLFQDR
RT: 36.59 AA: 20040461
RT: 29.29 AA: 4216192
RT: 31.47 AA: 495246
RT: 39.16 AA: 308208
NL: 2.06E6
m/z= 779.40-779.41+894.42-894.43+1007.51-1007.52
F: FTMS + c NSI Full ms2 668.3488@hcd30.00
[110.0000-1384.9616] MS ICIS 15_3207H
ASGIIDTLFQDR[HeavyR]
RT: 36.61 AA: 875509467
RT: 27.03 AA: 4800022
RT: 33.75 AA: 1553562
RT: 39.50 AA: 693561
NL: 8.11E7
m/z= 789.40-789.42+904.43-904.44+1017.51-1017.53
F: FTMS + c NSI Full ms2 673.3515@hcd30.00
[110.0000-1395.1671] MS ICIS 15_3207H
100
50
0
26 28 30 32 34 36 38 40
Time (min)

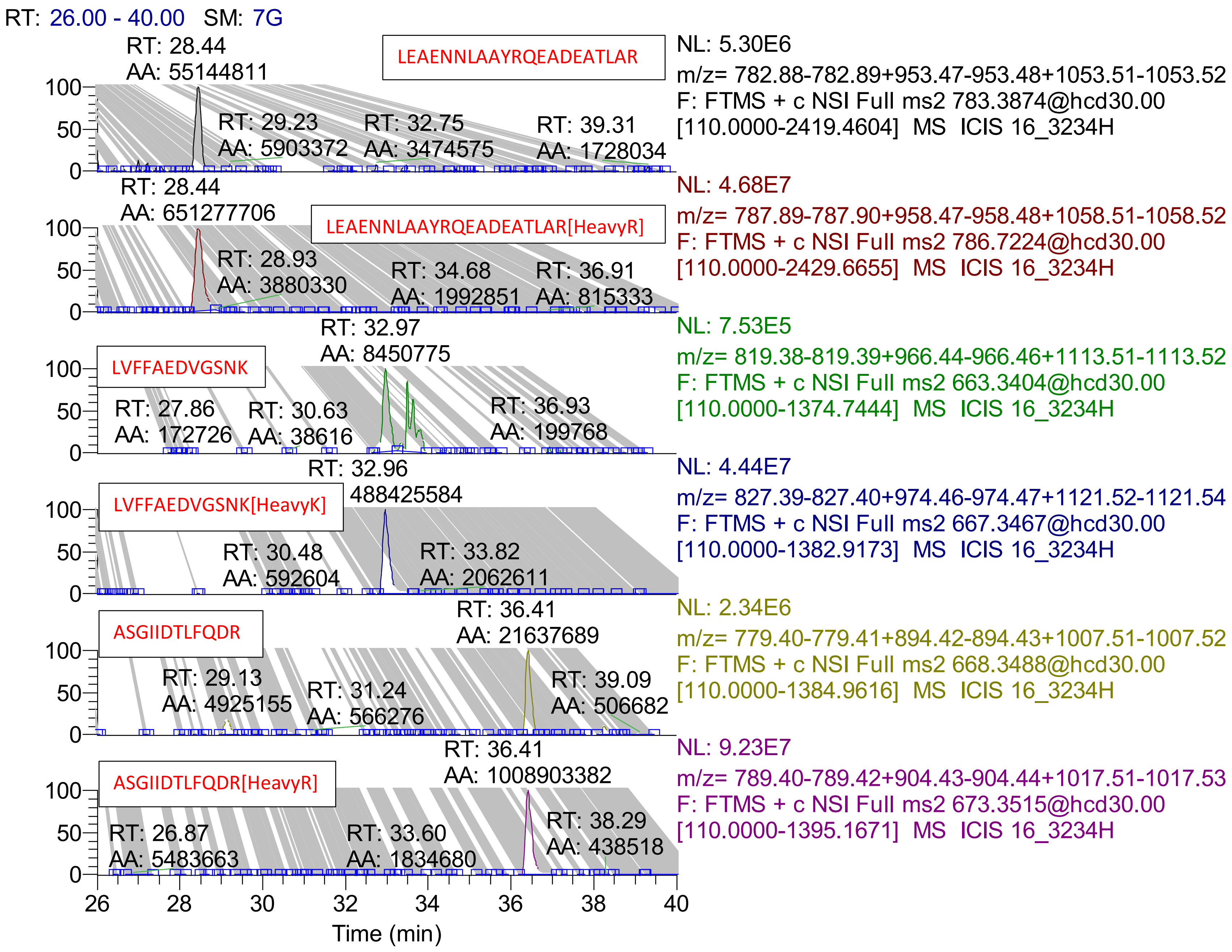

RT: 26.00 - 40.00 SM: 7G
LEAENNLAAYRQEADEATLAR
RT: 28.44
AA: 55144811
RT: 29.23
AA: 5903372
RT: 32.75
AA: 3474575
RT: 39.31
AA: 1728034
NL: 5.30E6
m/z= 782.88-782.89+953.47-953.48+1053.51-1053.52
F: FTMS + c NSI Full ms2 783.3874@hcd30.00
[110.0000-2419.4604] MS ICIS 16_3234H
LEAENNLAAYRQEADEATLAR[HeavyR]
RT: 28.44
AA: 651277706
RT: 28.93
AA: 3880330
RT: 34.68
AA: 1992851
RT: 36.91
AA: 815333
NL: 4.68E7
m/z= 787.89-787.90+958.47-958.48+1058.51-1058.52
F: FTMS + c NSI Full ms2 786.7224@hcd30.00
[110.0000-2429.6655] MS ICIS 16_3234H
LVFFAEDVGSNK
RT: 32.97
AA: 8450775
RT: 27.86
AA: 172726
RT: 30.63
AA: 38616
RT: 36.93
AA: 199768
NL: 7.53E5
m/z= 819.38-819.39+966.44-966.46+1113.51-1113.52
F: FTMS + c NSI Full ms2 663.3404@hcd30.00
[110.0000-1374.7444] MS ICIS 16_3234H
LVFFAEDVGSNK[HeavyK]
RT: 32.96
488425584
RT: 30.48
AA: 592604
RT: 33.82
AA: 2062611
NL: 4.44E7
m/z= 827.39-827.40+974.46-974.47+1121.52-1121.54
F: FTMS + c NSI Full ms2 667.3467@hcd30.00
[110.0000-1382.9173] MS ICIS 16_3234H
ASGIIDTLFQDR
RT: 36.41
AA: 21637689
RT: 29.13
AA: 4925155
RT: 31.24
AA: 566276
RT: 39.09
AA: 506682
NL: 2.34E6
m/z= 779.40-779.41+894.42-894.43+1007.51-1007.52
F: FTMS + c NSI Full ms2 668.3488@hcd30.00
[110.0000-1384.9616] MS ICIS 16_3234H
ASGIIDTLFQDR[HeavyR]
RT: 36.41
AA: 1008903382
RT: 26.87
AA: 5483663
RT: 33.60
AA: 1834680
RT: 38.29
AA: 438518
NL: 9.23E7
m/z= 789.40-789.42+904.43-904.44+1017.51-1017.53
F: FTMS + c NSI Full ms2 673.3515@hcd30.00
[110.0000-1395.1671] MS ICIS 16_3234H
100
50
0
26 28 30 32 34 36 38 40
Time (min)

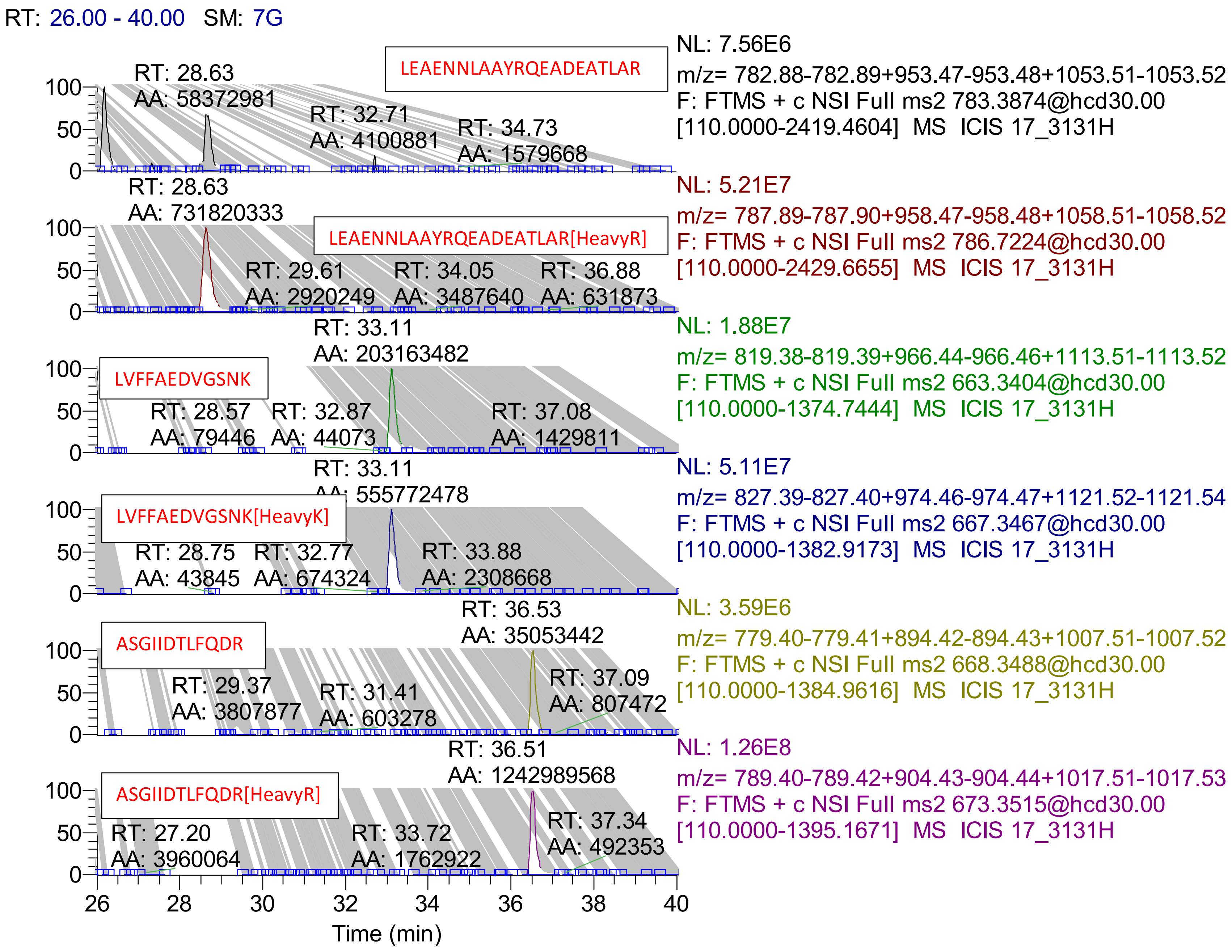

RT: 26.00 - 40.00 SM: 7G
LEAENNLAAYRQEADEATLAR
NL: 7.56E6
m/z= 782.88-782.89+953.47-953.48+1053.51-1053.52
F: FTMS + c NSI Full ms2 783.3874@hcd30.00
[110.0000-2419.4604] MS ICIS 17_3131H
RT: 28.63
AA: 58372981
RT: 32.71
AA: 4100881
RT: 34.73
AA: 1579668
LEAENNLAAYRQEADEATLAR[HeavyR]
NL: 5.21E7
m/z= 787.89-787.90+958.47-958.48+1058.51-1058.52
F: FTMS + c NSI Full ms2 786.7224@hcd30.00
[110.0000-2429.6655] MS ICIS 17_3131H
RT: 28.63
AA: 731820333
RT: 29.61
AA: 2920249
RT: 34.05
AA: 3487640
RT: 36.88
AA: 631873
LVFFAEDVGSNK
NL: 1.88E7
m/z= 819.38-819.39+966.44-966.46+1113.51-1113.52
F: FTMS + c NSI Full ms2 663.3404@hcd30.00
[110.0000-1374.7444] MS ICIS 17_3131H
RT: 33.11
AA: 203163482
RT: 28.57
AA: 79446
RT: 32.87
AA: 44073
RT: 37.08
AA: 1429811
LVFFAEDVGSNK[HeavyK]
NL: 5.11E7
m/z= 827.39-827.40+974.46-974.47+1121.52-1121.54
F: FTMS + c NSI Full ms2 667.3467@hcd30.00
[110.0000-1382.9173] MS ICIS 17_3131H
RT: 33.11
AA: 555772478
RT: 28.75
AA: 43845
RT: 32.77
AA: 674324
RT: 33.88
AA: 2308668
ASGIIDTLFQDR
NL: 3.59E6
m/z= 779.40-779.41+894.42-894.43+1007.51-1007.52
F: FTMS + c NSI Full ms2 668.3488@hcd30.00
[110.0000-1384.9616] MS ICIS 17_3131H
RT: 36.53
AA: 35053442
RT: 29.37
AA: 3807877
RT: 31.41
AA: 603278
RT: 37.09
AA: 807472
ASGIIDTLFQDR[HeavyR]
NL: 1.26E8
m/z= 789.40-789.42+904.43-904.44+1017.51-1017.53
F: FTMS + c NSI Full ms2 673.3515@hcd30.00
[110.0000-1395.1671] MS ICIS 17_3131H
RT: 36.51
AA: 1242989568
RT: 27.20
AA: 3960064
RT: 33.72
AA: 1762922
RT: 37.34
AA: 492353
100
50
0
26
28
30
32
34
36
38
40
Time (min)

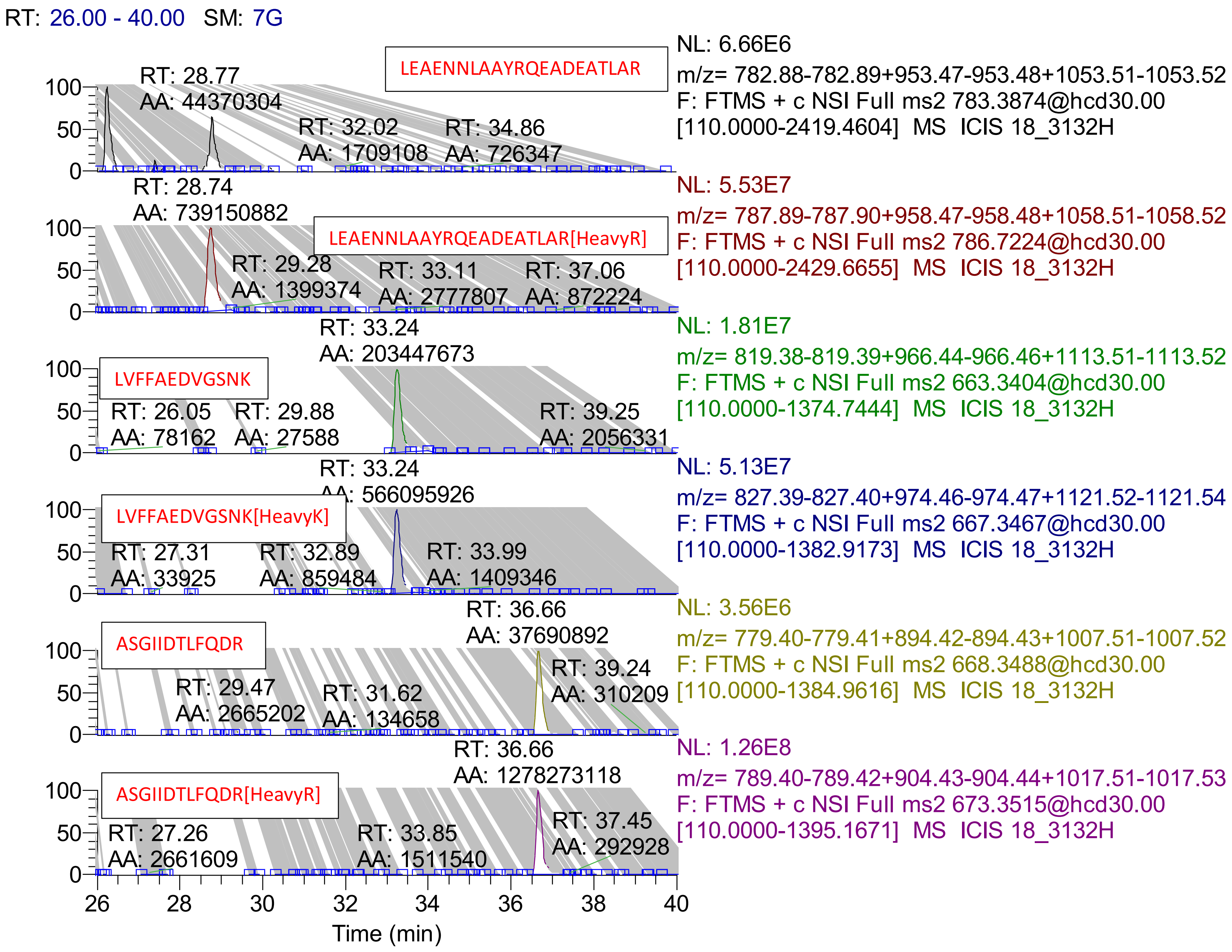

RT: 26.00 - 40.00 SM: 7G
LEAENNLAAYRQEADEATLAR
NL: 6.66E6
m/z= 782.88-782.89+953.47-953.48+1053.51-1053.52
F: FTMS + c NSI Full ms2 783.3874@hcd30.00
[110.0000-2419.4604] MS ICIS 18_3132H
RT: 28.77
AA: 44370304
RT: 32.02
AA: 1709108
RT: 34.86
AA: 726347
RT: 28.74
AA: 739150882
LEAENNLAAYRQEADEATLAR[HeavyR]
NL: 5.53E7
m/z= 787.89-787.90+958.47-958.48+1058.51-1058.52
F: FTMS + c NSI Full ms2 786.7224@hcd30.00
[110.0000-2429.6655] MS ICIS 18_3132H
RT: 29.28
AA: 1399374
RT: 33.11
AA: 2777807
RT: 37.06
AA: 872224
RT: 33.24
AA: 203447673
LVFFAEDVGSNK
NL: 1.81E7
m/z= 819.38-819.39+966.44-966.46+1113.51-1113.52
F: FTMS + c NSI Full ms2 663.3404@hcd30.00
[110.0000-1374.7444] MS ICIS 18_3132H
RT: 26.05
AA: 78162
RT: 29.88
AA: 27588
RT: 39.25
AA: 2056331
RT: 33.24
AA: 566095926
LVFFAEDVGSNK[HeavyK]
NL: 5.13E7
m/z= 827.39-827.40+974.46-974.47+1121.52-1121.54
F: FTMS + c NSI Full ms2 667.3467@hcd30.00
[110.0000-1382.9173] MS ICIS 18_3132H
RT: 27.31
AA: 33925
RT: 32.89
AA: 859484
RT: 33.99
AA: 1409346
RT: 36.66
AA: 37690892
ASGIIDTLFQDR
NL: 3.56E6
m/z= 779.40-779.41+894.42-894.43+1007.51-1007.52
F: FTMS + c NSI Full ms2 668.3488@hcd30.00
[110.0000-1384.9616] MS ICIS 18_3132H
RT: 29.47
AA: 2665202
RT: 31.62
AA: 134658
RT: 39.24
AA: 310209
RT: 36.66
AA: 1278273118
ASGIIDTLFQDR[HeavyR]
NL: 1.26E8
m/z= 789.40-789.42+904.43-904.44+1017.51-1017.53
F: FTMS + c NSI Full ms2 673.3515@hcd30.00
[110.0000-1395.1671] MS ICIS 18_3132H
RT: 27.26
AA: 2661609
RT: 33.85
AA: 1511540
RT: 37.45
AA: 292928
100
50
0
26
28
30
32
34
36
38
40
Time (min)

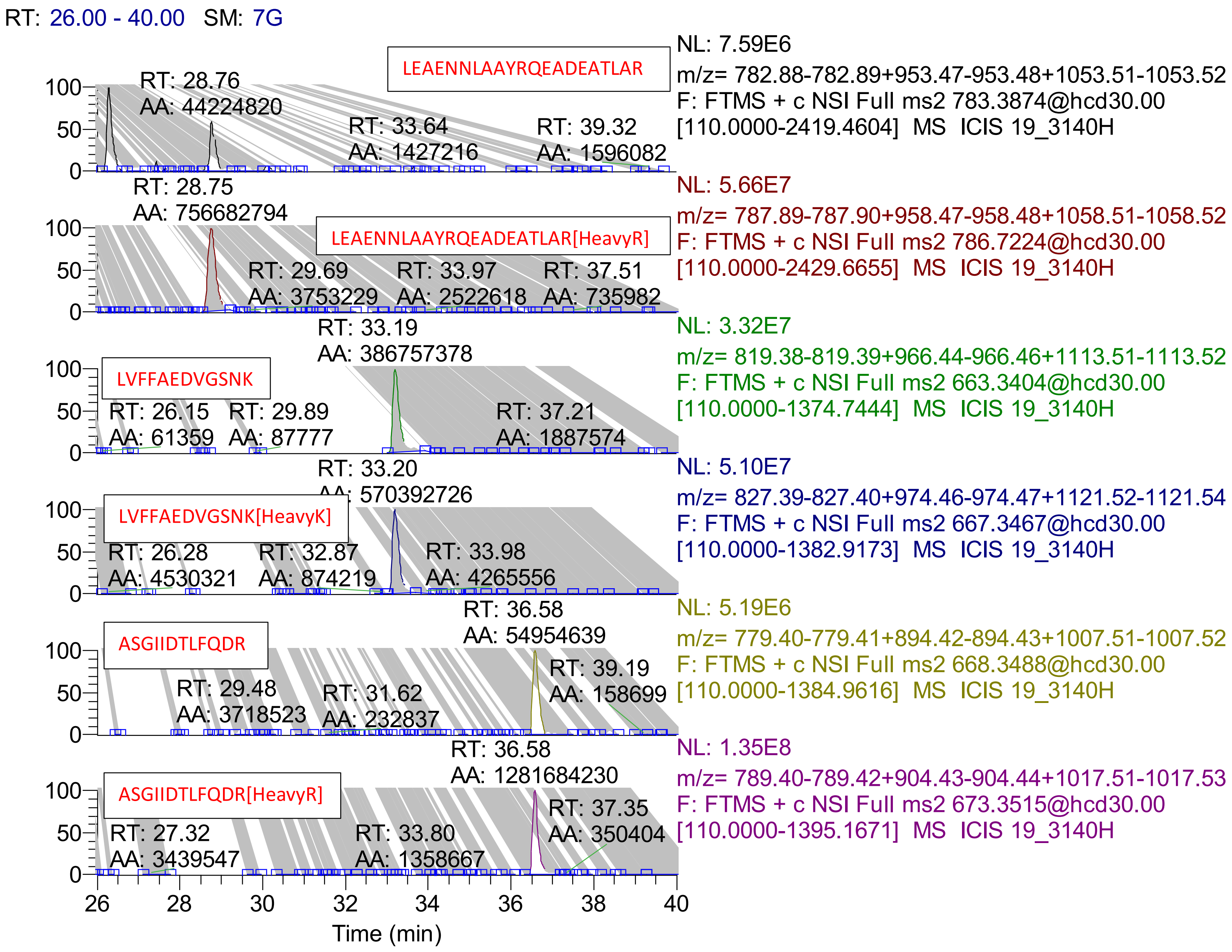
RT: 26.00 - 40.00 SM: 7G
LEAENNLAAYRQEADEATLAR
NL: 7.59E6
m/z= 782.88-782.89+953.47-953.48+1053.51-1053.52
F: FTMS + c NSI Full ms2 783.3874@hcd30.00
[110.0000-2419.4604] MS ICIS 19_3140H
RT: 28.76
AA: 44224820
RT: 33.64
AA: 1427216
RT: 39.32
AA: 1596082
RT: 28.75
AA: 756682794
LEAENNLAAYRQEADEATLAR[HeavyR]
NL: 5.66E7
m/z= 787.89-787.90+958.47-958.48+1058.51-1058.52
F: FTMS + c NSI Full ms2 786.7224@hcd30.00
[110.0000-2429.6655] MS ICIS 19_3140H
RT: 29.69
AA: 3753229
RT: 33.97
AA: 2522618
RT: 37.51
AA: 735982
RT: 33.19
AA: 386757378
LVFFAEDVGSNK
NL: 3.32E7
m/z= 819.38-819.39+966.44-966.46+1113.51-1113.52
F: FTMS + c NSI Full ms2 663.3404@hcd30.00
[110.0000-1374.7444] MS ICIS 19_3140H
RT: 26.15
AA: 61359
RT: 29.89
AA: 87777
RT: 37.21
AA: 1887574
RT: 33.20
AA: 570392726
LVFFAEDVGSNK[HeavyK]
NL: 5.10E7
m/z= 827.39-827.40+974.46-974.47+1121.52-1121.54
F: FTMS + c NSI Full ms2 667.3467@hcd30.00
[110.0000-1382.9173] MS ICIS 19_3140H
RT: 26.28
AA: 4530321
RT: 32.87
AA: 874219
RT: 33.98
AA: 4265556
RT: 36.58
AA: 54954639
ASGIIDTLFQDR
NL: 5.19E6
m/z= 779.40-779.41+894.42-894.43+1007.51-1007.52
F: FTMS + c NSI Full ms2 668.3488@hcd30.00
[110.0000-1384.9616] MS ICIS 19_3140H
RT: 29.48
AA: 3718523
RT: 31.62
AA: 232837
RT: 39.19
AA: 158699
RT: 36.58
AA: 1281684230
ASGIIDTLFQDR[HeavyR]
NL: 1.35E8
m/z= 789.40-789.42+904.43-904.44+1017.51-1017.53
F: FTMS + c NSI Full ms2 673.3515@hcd30.00
[110.0000-1395.1671] MS ICIS 19_3140H
RT: 27.32
AA: 3439547
RT: 33.80
AA: 1358667
RT: 37.35
AA: 350404
0
50
100
26
28
30
32
34
36
38
40
Time (min)

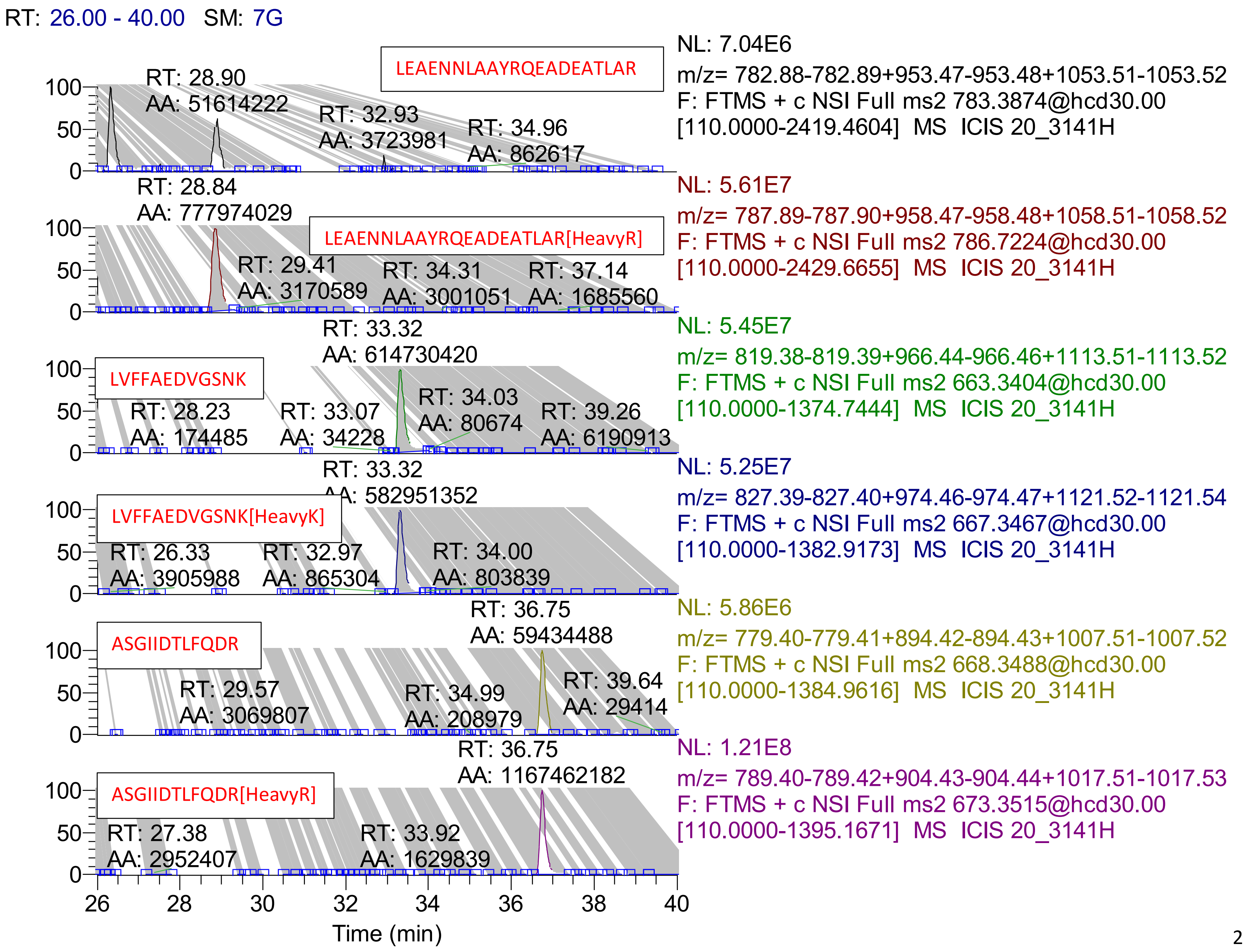

RT: 26.00 - 40.00 SM: 7G
LEAENNLAAYRQEADEATLAR
RT: 28.90
AA: 51614222
RT: 32.93
AA: 3723981
RT: 34.96
AA: 862617
NL: 7.04E6
m/z= 782.88-782.89+953.47-953.48+1053.51-1053.52
F: FTMS + c NSI Full ms2 783.3874@hcd30.00
[110.0000-2419.4604] MS ICIS 20_3141H
RT: 28.84
AA: 777974029
LEAENNLAAYRQEADEATLAR[HeavyR]
RT: 29.41
AA: 3170589
RT: 34.31
AA: 3001051
RT: 37.14
AA: 1685560
NL: 5.61E7
m/z= 787.89-787.90+958.47-958.48+1058.51-1058.52
F: FTMS + c NSI Full ms2 786.7224@hcd30.00
[110.0000-2429.6655] MS ICIS 20_3141H
RT: 33.32
AA: 614730420
LVFFAEDVGSNK
RT: 28.23
AA: 174485
RT: 33.07
AA: 34228
RT: 34.03
AA: 80674
RT: 39.26
AA: 6190913
NL: 5.45E7
m/z= 819.38-819.39+966.44-966.46+1113.51-1113.52
F: FTMS + c NSI Full ms2 663.3404@hcd30.00
[110.0000-1374.7444] MS ICIS 20_3141H
RT: 33.32
AA: 582951352
LVFFAEDVGSNK[HeavyK]
RT: 26.33
AA: 3905988
RT: 32.97
AA: 865304
RT: 34.00
AA: 803839
NL: 5.25E7
m/z= 827.39-827.40+974.46-974.47+1121.52-1121.54
F: FTMS + c NSI Full ms2 667.3467@hcd30.00
[110.0000-1382.9173] MS ICIS 20_3141H
RT: 36.75
AA: 59434488
ASGIIDTLFQDR
RT: 29.57
AA: 3069807
RT: 34.99
AA: 208979
RT: 39.64
AA: 29414
NL: 5.86E6
m/z= 779.40-779.41+894.42-894.43+1007.51-1007.52
F: FTMS + c NSI Full ms2 668.3488@hcd30.00
[110.0000-1384.9616] MS ICIS 20_3141H
RT: 36.75
AA: 1167462182
ASGIIDTLFQDR[HeavyR]
RT: 27.38
AA: 2952407
RT: 33.92
AA: 1629839
NL: 1.21E8
m/z= 789.40-789.42+904.43-904.44+1017.51-1017.53
F: FTMS + c NSI Full ms2 673.3515@hcd30.00
[110.0000-1395.1671] MS ICIS 20_3141H
100
50
0
26
28
30
32
34
36
38
40
Time (min)

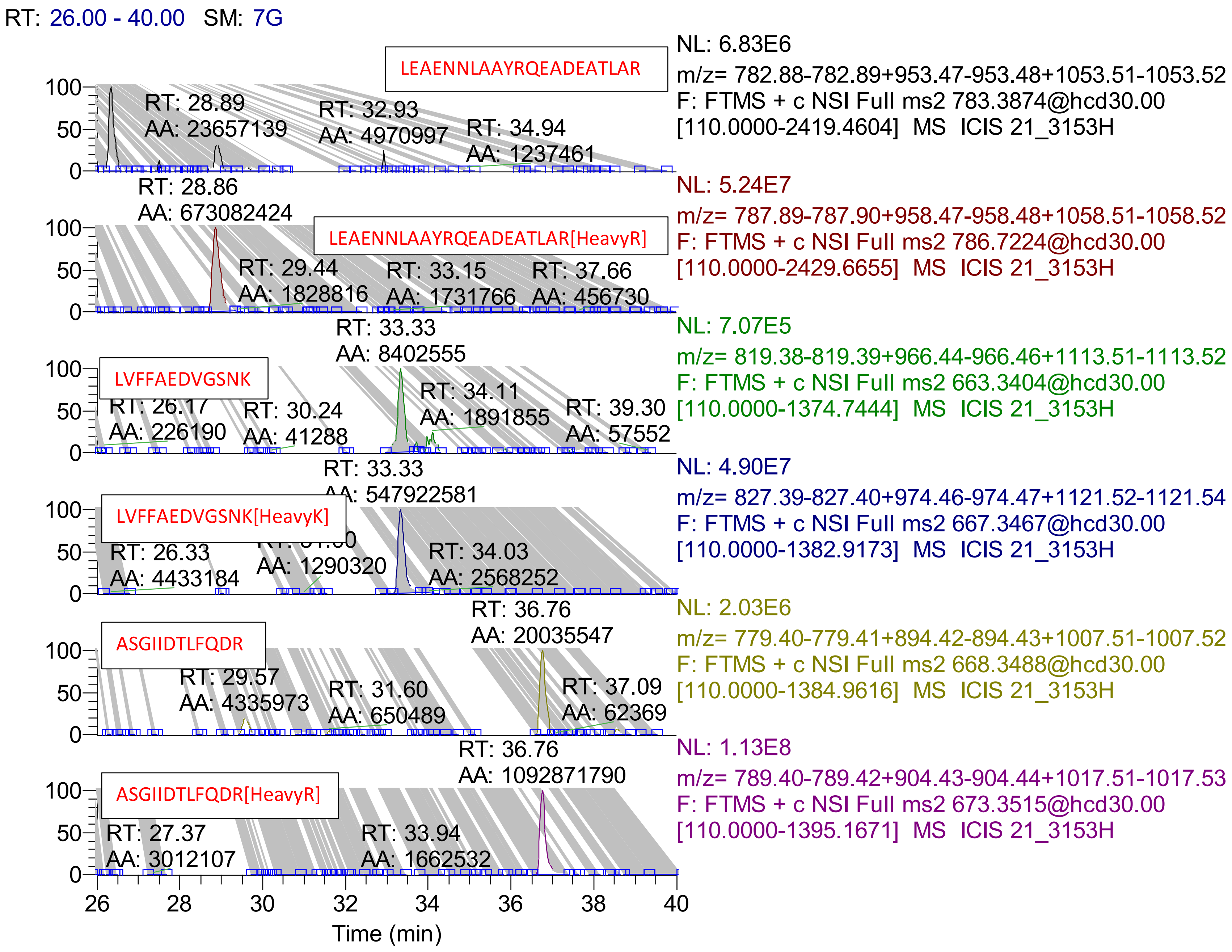

RT: 26.00 - 40.00 SM: 7G
LEAENNLAAYRQEADEATLAR
RT: 28.89
AA: 23657139
RT: 32.93
AA: 4970997
RT: 34.94
AA: 1237461
NL: 6.83E6
m/z= 782.88-782.89+953.47-953.48+1053.51-1053.52
F: FTMS + c NSI Full ms2 783.3874@hcd30.00
[110.0000-2419.4604] MS ICIS 21_3153H
RT: 28.86
AA: 673082424
LEAENNLAAYRQEADEATLAR[HeavyR]
RT: 29.44
AA: 1828816
RT: 33.15
AA: 1731766
RT: 37.66
AA: 456730
NL: 5.24E7
m/z= 787.89-787.90+958.47-958.48+1058.51-1058.52
F: FTMS + c NSI Full ms2 786.7224@hcd30.00
[110.0000-2429.6655] MS ICIS 21_3153H
RT: 33.33
AA: 8402555
LVFFAEDVGSNK
RT: 26.17
AA: 226190
RT: 30.24
AA: 41288
RT: 34.11
AA: 1891855
RT: 39.30
AA: 57552
NL: 7.07E5
m/z= 819.38-819.39+966.44-966.46+1113.51-1113.52
F: FTMS + c NSI Full ms2 663.3404@hcd30.00
[110.0000-1374.7444] MS ICIS 21_3153H
RT: 33.33
A: 547922581
LVFFAEDVGSNK[HeavyK]
RT: 26.33
AA: 4433184
AA: 1290320
RT: 34.03
AA: 2568252
NL: 4.90E7
m/z= 827.39-827.40+974.46-974.47+1121.52-1121.54
F: FTMS + c NSI Full ms2 667.3467@hcd30.00
[110.0000-1382.9173] MS ICIS 21_3153H
RT: 36.76
AA: 20035547
ASGIIDTLFQDR
RT: 29.57
AA: 4335973
RT: 31.60
AA: 650489
RT: 37.09
AA: 62369
NL: 2.03E6
m/z= 779.40-779.41+894.42-894.43+1007.51-1007.52
F: FTMS + c NSI Full ms2 668.3488@hcd30.00
[110.0000-1384.9616] MS ICIS 21_3153H
RT: 36.76
AA: 1092871790
ASGIIDTLFQDR[HeavyR]
RT: 27.37
AA: 3012107
RT: 33.94
AA: 1662532
NL: 1.13E8
m/z= 789.40-789.42+904.43-904.44+1017.51-1017.53
F: FTMS + c NSI Full ms2 673.3515@hcd30.00
[110.0000-1395.1671] MS ICIS 21_3153H
100
50
0
26
28
30
32
34
36
38
40
Time (min)

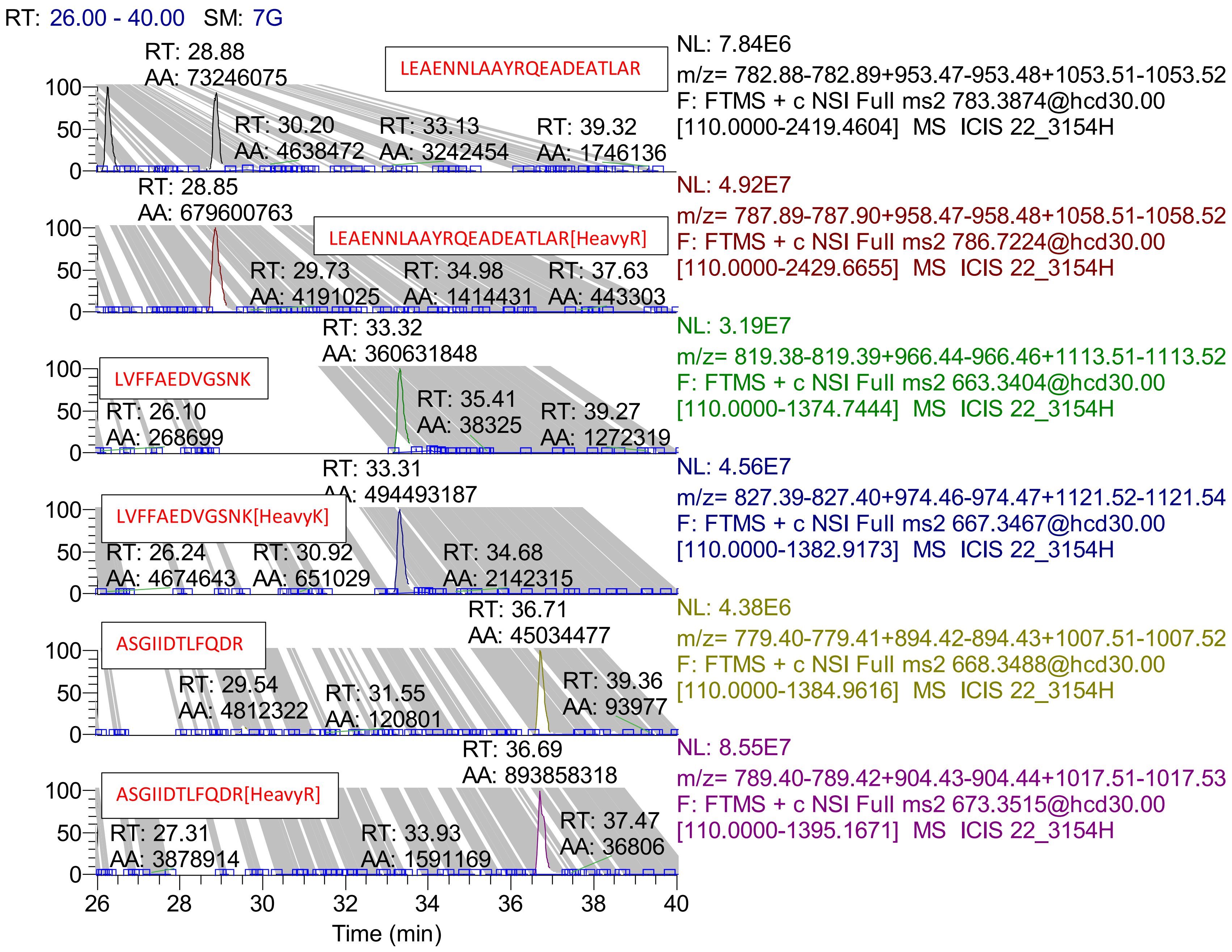
RT: 26.00 - 40.00 SM: 7G
RT: 28.88
AA: 73246075
LEAEENNLAAYRQEADEATLAR
RT: 30.20
AA: 4638472
RT: 33.13
AA: 3242454
RT: 39.32
AA: 1746136
NL: 7.84E6
m/z= 782.88-782.89+953.47-953.48+1053.51-1053.52
F: FTMS + c NSI Full ms2 783.3874@hcd30.00
[110.0000-2419.4604] MS ICIS 22_3154H
RT: 28.85
AA: 679600763
LEAEENNLAAYRQEADEATLAR[HeavyR]
RT: 29.73
AA: 4191025
RT: 34.98
AA: 1414431
RT: 37.63
AA: 443303
NL: 4.92E7
m/z= 787.89-787.90+958.47-958.48+1058.51-1058.52
F: FTMS + c NSI Full ms2 786.7224@hcd30.00
[110.0000-2429.6655] MS ICIS 22_3154H
RT: 33.32
AA: 360631848
LVFFAEDVGSNK
RT: 26.10
AA: 268699
RT: 35.41
AA: 38325
RT: 39.27
AA: 1272319
NL: 3.19E7
m/z= 819.38-819.39+966.44-966.46+1113.51-1113.52
F: FTMS + c NSI Full ms2 663.3404@hcd30.00
[110.0000-1374.7444] MS ICIS 22_3154H
RT: 33.31
AA: 494493187
LVFFAEDVGSNK[HeavyK]
RT: 26.24
AA: 4674643
RT: 30.92
AA: 651029
RT: 34.68
AA: 2142315
NL: 4.56E7
m/z= 827.39-827.40+974.46-974.47+1121.52-1121.54
F: FTMS + c NSI Full ms2 667.3467@hcd30.00
[110.0000-1382.9173] MS ICIS 22_3154H
RT: 36.71
AA: 45034477
ASGIIDTLFQDR
RT: 29.54
AA: 4812322
RT: 31.55
AA: 120801
RT: 39.36
AA: 93977
NL: 4.38E6
m/z= 779.40-779.41+894.42-894.43+1007.51-1007.52
F: FTMS + c NSI Full ms2 668.3488@hcd30.00
[110.0000-1384.9616] MS ICIS 22_3154H
RT: 36.69
AA: 893858318
ASGIIDTLFQDR[HeavyR]
RT: 27.31
AA: 3878914
RT: 33.93
AA: 1591169
RT: 37.47
AA: 36806
NL: 8.55E7
m/z= 789.40-789.42+904.43-904.44+1017.51-1017.53
F: FTMS + c NSI Full ms2 673.3515@hcd30.00
[110.0000-1395.1671] MS ICIS 22_3154H
100
50
0
26
28
30
32
34
36
38
40
Time (min)

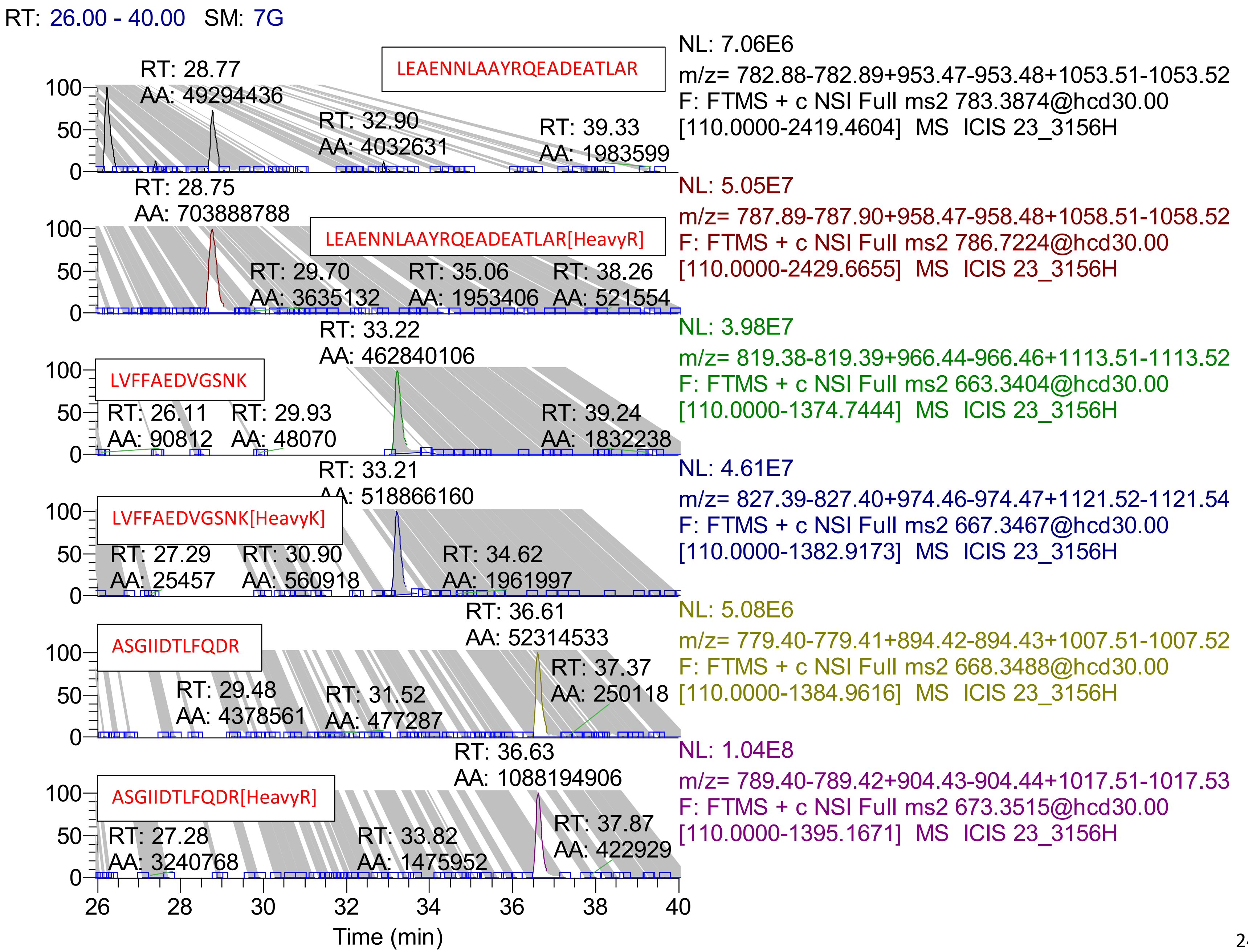
RT: 26.00 - 40.00 SM: 7G
RT: 28.77
AA: 49294436
LEAENNLAAYRQEADEATLAR
RT: 32.90
AA: 4032631
RT: 39.33
AA: 1983599
NL: 7.06E6
m/z= 782.88-782.89+953.47-953.48+1053.51-1053.52
F: FTMS + c NSI Full ms2 783.3874@hcd30.00
[110.0000-2419.4604] MS ICIS 23_3156H
RT: 28.75
AA: 703888788
LEAENNLAAYRQEADEATLAR[HeavyR]
RT: 29.70
AA: 3635132
RT: 35.06
AA: 1953406
RT: 38.26
AA: 521554
NL: 5.05E7
m/z= 787.89-787.90+958.47-958.48+1058.51-1058.52
F: FTMS + c NSI Full ms2 786.7224@hcd30.00
[110.0000-2429.6655] MS ICIS 23_3156H
RT: 33.22
AA: 462840106
LVFFAEDVGSNK
RT: 26.11
AA: 90812
RT: 29.93
AA: 48070
RT: 39.24
AA: 1832238
NL: 3.98E7
m/z= 819.38-819.39+966.44-966.46+1113.51-1113.52
F: FTMS + c NSI Full ms2 663.3404@hcd30.00
[110.0000-1374.7444] MS ICIS 23_3156H
RT: 33.21
AA: 518866160
LVFFAEDVGSNK[HeavyK]
RT: 27.29
AA: 25457
RT: 30.90
AA: 560918
RT: 34.62
AA: 1961997
NL: 4.61E7
m/z= 827.39-827.40+974.46-974.47+1121.52-1121.54
F: FTMS + c NSI Full ms2 667.3467@hcd30.00
[110.0000-1382.9173] MS ICIS 23_3156H
RT: 36.61
AA: 52314533
ASGIIDTLFQDR
RT: 29.48
AA: 4378561
RT: 31.52
AA: 477287
RT: 37.37
AA: 250118
NL: 5.08E6
m/z= 779.40-779.41+894.42-894.43+1007.51-1007.52
F: FTMS + c NSI Full ms2 668.3488@hcd30.00
[110.0000-1384.9616] MS ICIS 23_3156H
RT: 36.63
AA: 1088194906
ASGIIDTLFQDR[HeavyR]
RT: 27.28
AA: 3240768
RT: 33.82
AA: 1475952
RT: 37.87
AA: 422929
NL: 1.04E8
m/z= 789.40-789.42+904.43-904.44+1017.51-1017.53
F: FTMS + c NSI Full ms2 673.3515@hcd30.00
[110.0000-1395.1671] MS ICIS 23_3156H
100
50
0
26 28 30 32 34 36 38 40
Time (min)

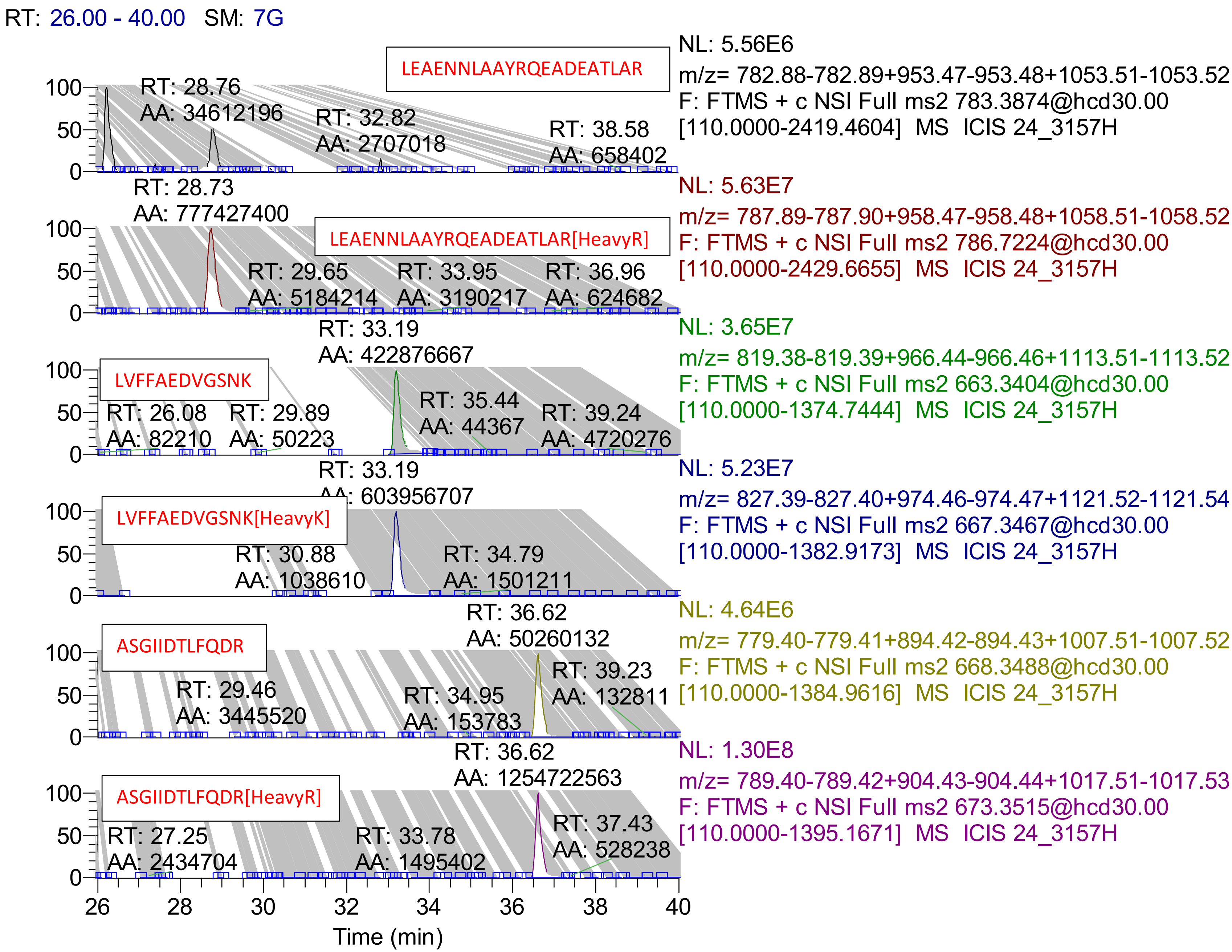
RT: 26.00 - 40.00 SM: 7G
LEAENNLAAYRQEADEATLAR
RT: 28.76
AA: 34612196
RT: 32.82
AA: 2707018
RT: 38.58
AA: 658402
NL: 5.56E6
m/z= 782.88-782.89+953.47-953.48+1053.51-1053.52
F: FTMS + c NSI Full ms2 783.3874@hcd30.00
[110.0000-2419.4604] MS ICIS 24_3157H
RT: 28.73
AA: 777427400
LEAENNLAAYRQEADEATLAR[HeavyR]
RT: 29.65
AA: 5184214
RT: 33.95
AA: 3190217
RT: 36.96
AA: 624682
NL: 5.63E7
m/z= 787.89-787.90+958.47-958.48+1058.51-1058.52
F: FTMS + c NSI Full ms2 786.7224@hcd30.00
[110.0000-2429.6655] MS ICIS 24_3157H
LVFFAEDVGSNK
RT: 26.08
AA: 82210
RT: 29.89
AA: 50223
RT: 33.19
AA: 422876667
RT: 35.44
AA: 44367
RT: 39.24
AA: 4720276
NL: 3.65E7
m/z= 819.38-819.39+966.44-966.46+1113.51-1113.52
F: FTMS + c NSI Full ms2 663.3404@hcd30.00
[110.0000-1374.7444] MS ICIS 24_3157H
LVFFAEDVGSNK[HeavyK]
RT: 33.19
AA: 603956707
RT: 30.88
AA: 1038610
RT: 34.79
AA: 1501211
NL: 5.23E7
m/z= 827.39-827.40+974.46-974.47+1121.52-1121.54
F: FTMS + c NSI Full ms2 667.3467@hcd30.00
[110.0000-1382.9173] MS ICIS 24_3157H
ASGIIDTLFQDR
RT: 36.62
AA: 50260132
RT: 29.46
AA: 3445520
RT: 34.95
AA: 153783
RT: 39.23
AA: 132811
NL: 4.64E6
m/z= 779.40-779.41+894.42-894.43+1007.51-1007.52
F: FTMS + c NSI Full ms2 668.3488@hcd30.00
[110.0000-1384.9616] MS ICIS 24_3157H
ASGIIDTLFQDR[HeavyR]
RT: 36.62
AA: 1254722563
RT: 27.25
AA: 2434704
RT: 33.78
AA: 1495402
RT: 37.43
AA: 528238
NL: 1.30E8
m/z= 789.40-789.42+904.43-904.44+1017.51-1017.53
F: FTMS + c NSI Full ms2 673.3515@hcd30.00
[110.0000-1395.1671] MS ICIS 24_3157H
100
50
0
26
28
30
32
34
36
38
40
Time (min)

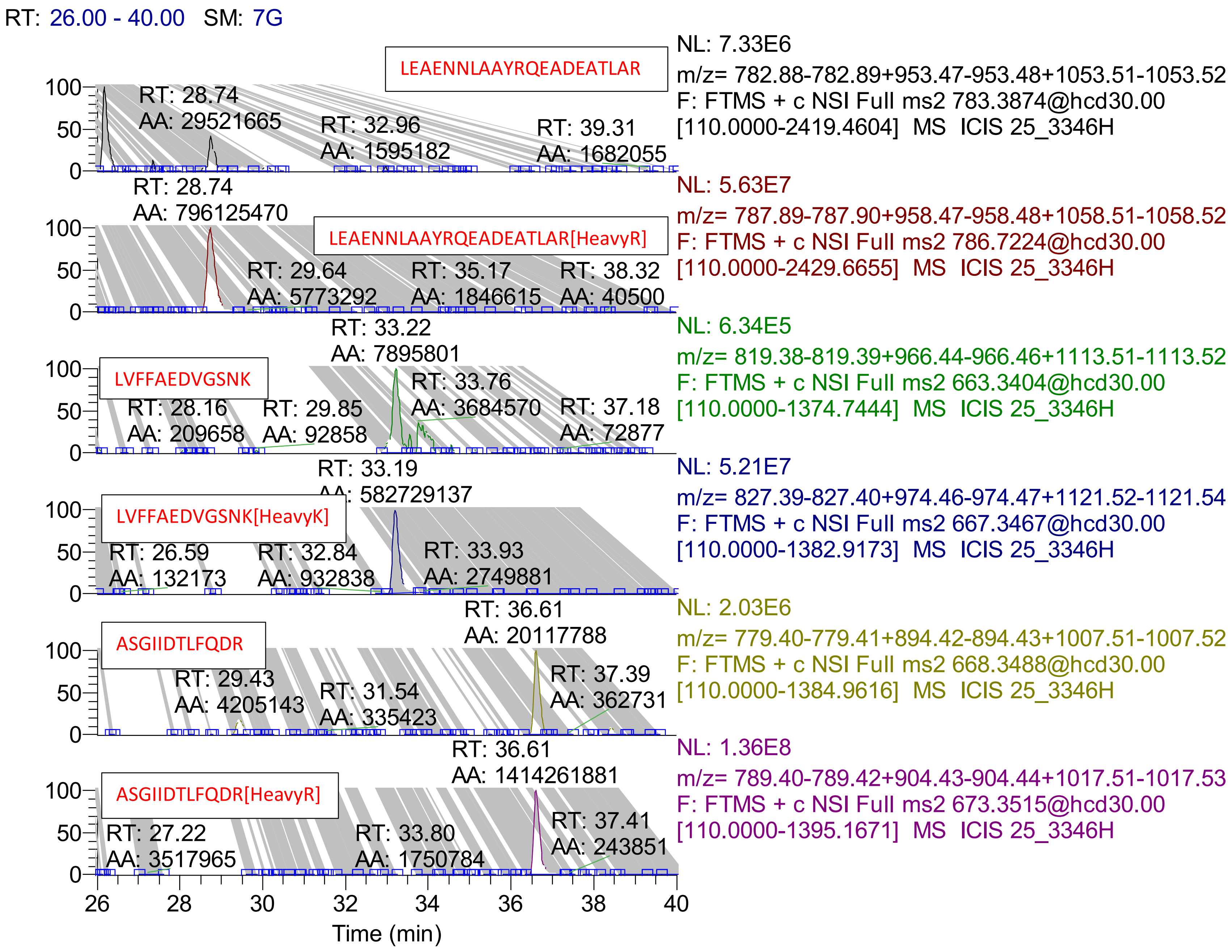

RT: 26.00 - 40.00 SM: 7G
LEAENNLAAYRQEADEATLAR
RT: 28.74
AA: 29521665
RT: 32.96
AA: 1595182
RT: 39.31
AA: 1682055
NL: 7.33E6
m/z= 782.88-782.89+953.47-953.48+1053.51-1053.52
F: FTMS + c NSI Full ms2 783.3874@hcd30.00
[110.0000-2419.4604] MS ICIS 25_3346H
RT: 28.74
AA: 796125470
LEAENNLAAYRQEADEATLAR[HeavyR]
RT: 29.64
AA: 5773292
RT: 35.17
AA: 1846615
RT: 38.32
AA: 40500
NL: 5.63E7
m/z= 787.89-787.90+958.47-958.48+1058.51-1058.52
F: FTMS + c NSI Full ms2 786.7224@hcd30.00
[110.0000-2429.6655] MS ICIS 25_3346H
RT: 33.22
AA: 7895801
LVFFAEDVGSNK
RT: 28.16
AA: 209658
RT: 29.85
AA: 92858
RT: 33.76
AA: 3684570
RT: 37.18
AA: 72877
NL: 6.34E5
m/z= 819.38-819.39+966.44-966.46+1113.51-1113.52
F: FTMS + c NSI Full ms2 663.3404@hcd30.00
[110.0000-1374.7444] MS ICIS 25_3346H
RT: 33.19
AA: 582729137
LVFFAEDVGSNK[HeavyK]
RT: 26.59
AA: 132173
RT: 32.84
AA: 932838
RT: 33.93
AA: 2749881
NL: 5.21E7
m/z= 827.39-827.40+974.46-974.47+1121.52-1121.54
F: FTMS + c NSI Full ms2 667.3467@hcd30.00
[110.0000-1382.9173] MS ICIS 25_3346H
RT: 36.61
AA: 20117788
ASGIIDTLFQDR
RT: 29.43
AA: 4205143
RT: 31.54
AA: 335423
RT: 37.39
AA: 362731
NL: 2.03E6
m/z= 779.40-779.41+894.42-894.43+1007.51-1007.52
F: FTMS + c NSI Full ms2 668.3488@hcd30.00
[110.0000-1384.9616] MS ICIS 25_3346H
RT: 36.61
AA: 1414261881
ASGIIDTLFQDR[HeavyR]
RT: 27.22
AA: 3517965
RT: 33.80
AA: 1750784
RT: 37.41
AA: 243851
NL: 1.36E8
m/z= 789.40-789.42+904.43-904.44+1017.51-1017.53
F: FTMS + c NSI Full ms2 673.3515@hcd30.00
[110.0000-1395.1671] MS ICIS 25_3346H
100
50
0
26 28 30 32 34 36 38 40
Time (min)

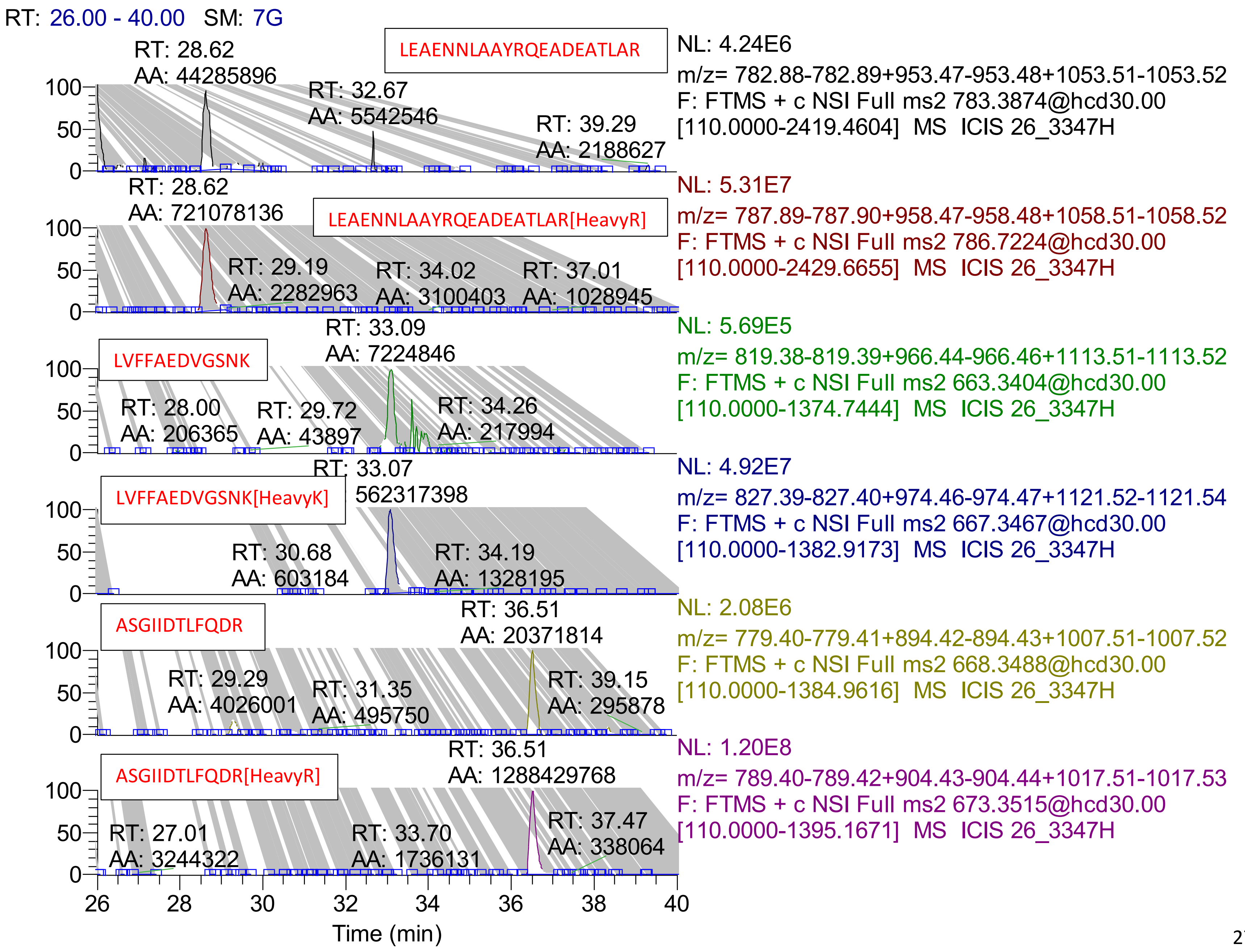

RT: 26.00 - 40.00 SM: 7G
LEAENNLAAYRQEADEATLAR
RT: 28.62
AA: 44285896
RT: 32.67
AA: 5542546
RT: 39.29
AA: 2188627
NL: 4.24E6
m/z= 782.88-782.89+953.47-953.48+1053.51-1053.52
F: FTMS + c NSI Full ms2 783.3874@hcd30.00
[110.0000-2419.4604] MS ICIS 26_3347H
LEAENNLAAYRQEADEATLAR[HeavyR]
RT: 28.62
AA: 721078136
RT: 29.19
AA: 2282963
RT: 34.02
AA: 3100403
RT: 37.01
AA: 1028945
NL: 5.31E7
m/z= 787.89-787.90+958.47-958.48+1058.51-1058.52
F: FTMS + c NSI Full ms2 786.7224@hcd30.00
[110.0000-2429.6655] MS ICIS 26_3347H
LVFFAEDVGSNK
RT: 33.09
AA: 7224846
RT: 28.00
AA: 206365
RT: 29.72
AA: 43897
RT: 34.26
AA: 217994
NL: 5.69E5
m/z= 819.38-819.39+966.44-966.46+1113.51-1113.52
F: FTMS + c NSI Full ms2 663.3404@hcd30.00
[110.0000-1374.7444] MS ICIS 26_3347H
LVFFAEDVGSNK[HeavyK]
RT: 33.07
562317398
RT: 30.68
AA: 603184
RT: 34.19
AA: 1328195
NL: 4.92E7
m/z= 827.39-827.40+974.46-974.47+1121.52-1121.54
F: FTMS + c NSI Full ms2 667.3467@hcd30.00
[110.0000-1382.9173] MS ICIS 26_3347H
ASGIIDTLFQDR
RT: 36.51
AA: 20371814
RT: 29.29
AA: 4026001
RT: 31.35
AA: 495750
RT: 39.15
AA: 295878
NL: 2.08E6
m/z= 779.40-779.41+894.42-894.43+1007.51-1007.52
F: FTMS + c NSI Full ms2 668.3488@hcd30.00
[110.0000-1384.9616] MS ICIS 26_3347H
ASGIIDTLFQDR[HeavyR]
RT: 36.51
AA: 1288429768
RT: 27.01
AA: 3244322
RT: 33.70
AA: 1736131
RT: 37.47
AA: 338064
NL: 1.20E8
m/z= 789.40-789.42+904.43-904.44+1017.51-1017.53
F: FTMS + c NSI Full ms2 673.3515@hcd30.00
[110.0000-1395.1671] MS ICIS 26_3347H
100
50
0
26
28
30
32
34
36
38
40
Time (min)

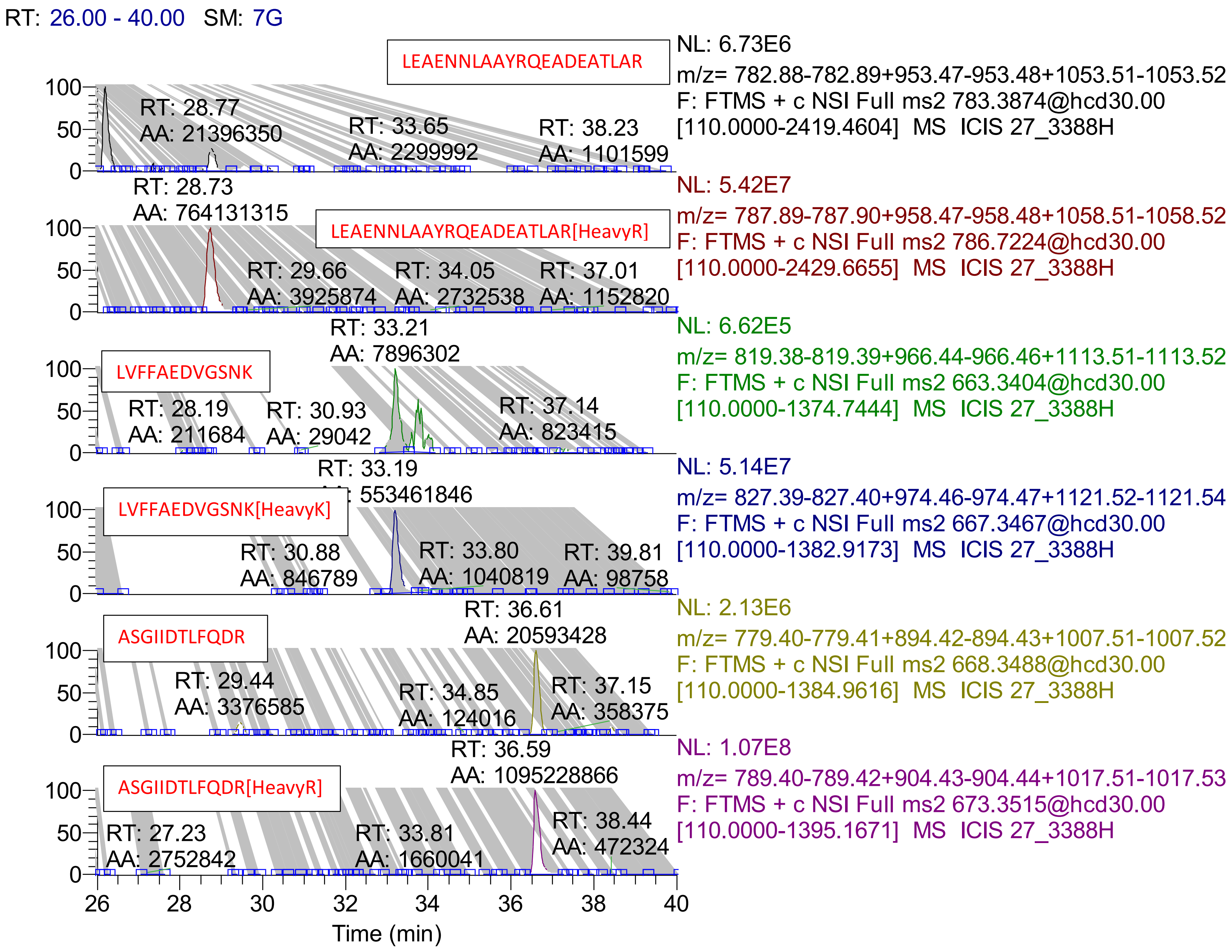
RT: 26.00 - 40.00 SM: 7G
LEAENNLAAYRQEADEATLAR
NL: 6.73E6
m/z= 782.88-782.89+953.47-953.48+1053.51-1053.52
F: FTMS + c NSI Full ms2 783.3874@hcd30.00
[110.0000-2419.4604] MS ICIS 27_3388H
RT: 28.77
AA: 21396350
RT: 33.65
AA: 2299992
RT: 38.23
AA: 1101599
RT: 28.73
AA: 764131315
LEAENNLAAYRQEADEATLAR[HeavyR]
NL: 5.42E7
m/z= 787.89-787.90+958.47-958.48+1058.51-1058.52
F: FTMS + c NSI Full ms2 786.7224@hcd30.00
[110.0000-2429.6655] MS ICIS 27_3388H
RT: 29.66
AA: 3925874
RT: 34.05
AA: 2732538
RT: 37.01
AA: 1152820
LVFFAEDVGSNK
RT: 33.21
AA: 7896302
NL: 6.62E5
m/z= 819.38-819.39+966.44-966.46+1113.51-1113.52
F: FTMS + c NSI Full ms2 663.3404@hcd30.00
[110.0000-1374.7444] MS ICIS 27_3388H
RT: 28.19
AA: 211684
RT: 30.93
AA: 29042
RT: 37.14
AA: 823415
LVFFAEDVGSNK[HeavyK]
RT: 33.19
: 553461846
NL: 5.14E7
m/z= 827.39-827.40+974.46-974.47+1121.52-1121.54
F: FTMS + c NSI Full ms2 667.3467@hcd30.00
[110.0000-1382.9173] MS ICIS 27_3388H
RT: 30.88
AA: 846789
RT: 33.80
AA: 1040819
RT: 39.81
AA: 98758
ASGIIDTLFQDR
RT: 36.61
AA: 20593428
NL: 2.13E6
m/z= 779.40-779.41+894.42-894.43+1007.51-1007.52
F: FTMS + c NSI Full ms2 668.3488@hcd30.00
[110.0000-1384.9616] MS ICIS 27_3388H
RT: 29.44
AA: 3376585
RT: 34.85
AA: 124016
RT: 37.15
AA: 358375
ASGIIDTLFQDR[HeavyR]
RT: 36.59
AA: 1095228866
NL: 1.07E8
m/z= 789.40-789.42+904.43-904.44+1017.51-1017.53
F: FTMS + c NSI Full ms2 673.3515@hcd30.00
[110.0000-1395.1671] MS ICIS 27_3388H
RT: 27.23
AA: 2752842
RT: 33.81
AA: 1660041
RT: 38.44
AA: 472324
100
50
0
26
28
30
32
34
36
38
40
Time (min)

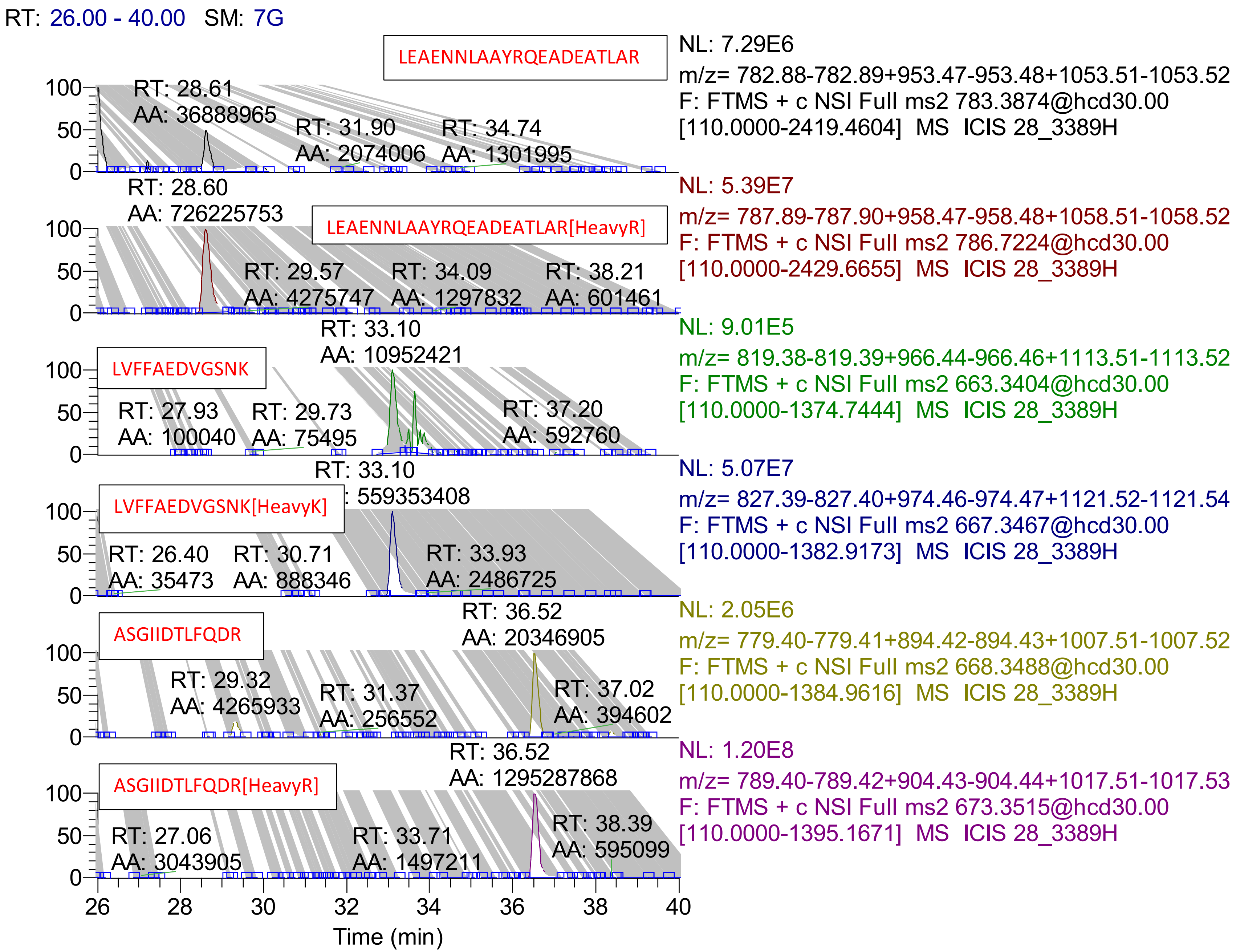

RT: 26.00 - 40.00 SM: 7G
LEAENNLAAYRQEADEATLAR
NL: 7.29E6
m/z= 782.88-782.89+953.47-953.48+1053.51-1053.52
F: FTMS + c NSI Full ms2 783.3874@hcd30.00
[110.0000-2419.4604] MS ICIS 28_3389H
RT: 28.61
AA: 36888965
RT: 31.90
AA: 2074006
RT: 34.74
AA: 1301995
RT: 28.60
AA: 726225753
LEAENNLAAYRQEADEATLAR[HeavyR]
NL: 5.39E7
m/z= 787.89-787.90+958.47-958.48+1058.51-1058.52
F: FTMS + c NSI Full ms2 786.7224@hcd30.00
[110.0000-2429.6655] MS ICIS 28_3389H
RT: 29.57
AA: 4275747
RT: 34.09
AA: 1297832
RT: 38.21
AA: 601461
RT: 33.10
AA: 10952421
LVFFAEDVGSNK
NL: 9.01E5
m/z= 819.38-819.39+966.44-966.46+1113.51-1113.52
F: FTMS + c NSI Full ms2 663.3404@hcd30.00
[110.0000-1374.7444] MS ICIS 28_3389H
RT: 27.93
AA: 100040
RT: 29.73
AA: 75495
RT: 37.20
AA: 592760
RT: 33.10
: 559353408
LVFFAEDVGSNK[HeavyK]
NL: 5.07E7
m/z= 827.39-827.40+974.46-974.47+1121.52-1121.54
F: FTMS + c NSI Full ms2 667.3467@hcd30.00
[110.0000-1382.9173] MS ICIS 28_3389H
RT: 26.40
AA: 35473
RT: 30.71
AA: 888346
RT: 33.93
AA: 2486725
RT: 36.52
AA: 20346905
ASGIIDTLFQDR
NL: 2.05E6
m/z= 779.40-779.41+894.42-894.43+1007.51-1007.52
F: FTMS + c NSI Full ms2 668.3488@hcd30.00
[110.0000-1384.9616] MS ICIS 28_3389H
RT: 29.32
AA: 4265933
RT: 31.37
AA: 256552
RT: 37.02
AA: 394602
RT: 36.52
AA: 1295287868
ASGIIDTLFQDR[HeavyR]
NL: 1.20E8
m/z= 789.40-789.42+904.43-904.44+1017.51-1017.53
F: FTMS + c NSI Full ms2 673.3515@hcd30.00
[110.0000-1395.1671] MS ICIS 28_3389H
RT: 27.06
AA: 3043905
RT: 33.71
AA: 1497211
RT: 38.39
AA: 595099
100
50
0
26
28
30
32
34
36
38
40
Time (min)

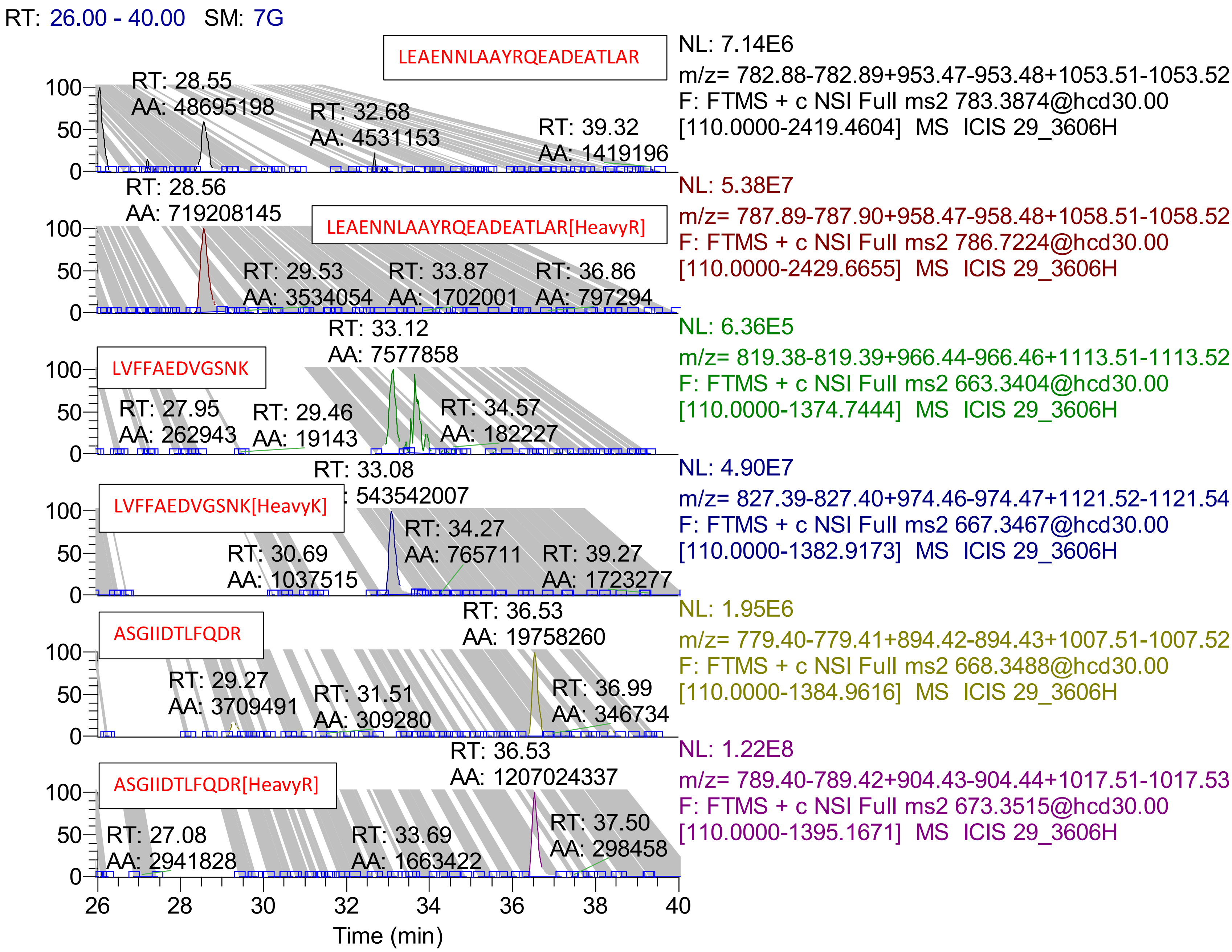
RT: 26.00 - 40.00 SM: 7G
LEAENNLAAYRQEADEATLAR
NL: 7.14E6
m/z= 782.88-782.89+953.47-953.48+1053.51-1053.52
F: FTMS + c NSI Full ms2 783.3874@hcd30.00
[110.0000-2419.4604] MS ICIS 29_3606H
RT: 28.55
AA: 48695198
RT: 32.68
AA: 4531153
RT: 39.32
AA: 1419196
RT: 28.56
AA: 719208145
LEAENNLAAYRQEADEATLAR[HeavyR]
NL: 5.38E7
m/z= 787.89-787.90+958.47-958.48+1058.51-1058.52
F: FTMS + c NSI Full ms2 786.7224@hcd30.00
[110.0000-2429.6655] MS ICIS 29_3606H
RT: 29.53
AA: 3534054
RT: 33.87
AA: 1702001
RT: 36.86
AA: 797294
LVFFAEDVGSNK
RT: 33.12
AA: 7577858
NL: 6.36E5
m/z= 819.38-819.39+966.44-966.46+1113.51-1113.52
F: FTMS + c NSI Full ms2 663.3404@hcd30.00
[110.0000-1374.7444] MS ICIS 29_3606H
RT: 27.95
AA: 262943
RT: 29.46
AA: 19143
RT: 34.57
AA: 182227
LVFFAEDVGSNK[HeavyK]
RT: 33.08
: 543542007
NL: 4.90E7
m/z= 827.39-827.40+974.46-974.47+1121.52-1121.54
F: FTMS + c NSI Full ms2 667.3467@hcd30.00
[110.0000-1382.9173] MS ICIS 29_3606H
RT: 30.69
AA: 1037515
RT: 34.27
AA: 765711
RT: 39.27
AA: 1723277
ASGIIDTLFQDR
RT: 36.53
AA: 19758260
NL: 1.95E6
m/z= 779.40-779.41+894.42-894.43+1007.51-1007.52
F: FTMS + c NSI Full ms2 668.3488@hcd30.00
[110.0000-1384.9616] MS ICIS 29_3606H
RT: 29.27
AA: 3709491
RT: 31.51
AA: 309280
RT: 36.99
AA: 346734
ASGIIDTLFQDR[HeavyR]
RT: 36.53
AA: 1207024337
NL: 1.22E8
m/z= 789.40-789.42+904.43-904.44+1017.51-1017.53
F: FTMS + c NSI Full ms2 673.3515@hcd30.00
[110.0000-1395.1671] MS ICIS 29_3606H
RT: 27.08
AA: 2941828
RT: 33.69
AA: 1663422
RT: 37.50
AA: 298458
100
50
0
26
28
30
32
34
36
38
40
Time (min)

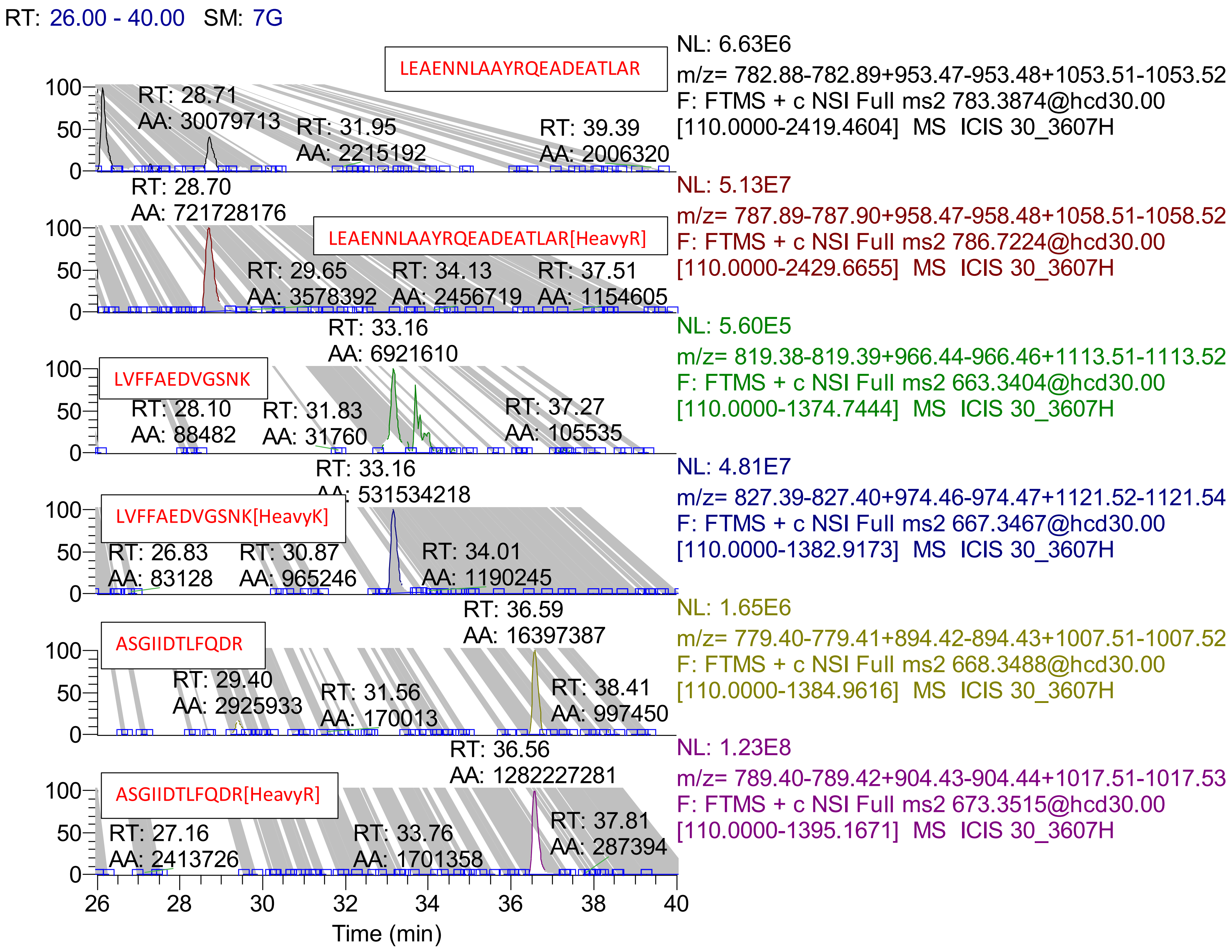

RT: 26.00 - 40.00 SM: 7G
LEAEENNLAAYRQEADEATLAR
RT: 28.71
AA: 30079713
RT: 31.95
AA: 2215192
RT: 39.39
AA: 2006320
NL: 6.63E6
m/z= 782.88-782.89+953.47-953.48+1053.51-1053.52
F: FTMS + c NSI Full ms2 783.3874@hcd30.00
[110.0000-2419.4604] MS ICIS 30_3607H
RT: 28.70
AA: 721728176
LEAEENNLAAYRQEADEATLAR[HeavyR]
RT: 29.65
AA: 3578392
RT: 34.13
AA: 2456719
RT: 37.51
AA: 1154605
NL: 5.13E7
m/z= 787.89-787.90+958.47-958.48+1058.51-1058.52
F: FTMS + c NSI Full ms2 786.7224@hcd30.00
[110.0000-2429.6655] MS ICIS 30_3607H
RT: 33.16
AA: 6921610
LVFFAEDVGSNK
RT: 28.10
AA: 88482
RT: 31.83
AA: 31760
RT: 37.27
AA: 105535
NL: 5.60E5
m/z= 819.38-819.39+966.44-966.46+1113.51-1113.52
F: FTMS + c NSI Full ms2 663.3404@hcd30.00
[110.0000-1374.7444] MS ICIS 30_3607H
RT: 33.16
AA: 531534218
LVFFAEDVGSNK[HeavyK]
RT: 26.83
AA: 83128
RT: 30.87
AA: 965246
RT: 34.01
AA: 1190245
NL: 4.81E7
m/z= 827.39-827.40+974.46-974.47+1121.52-1121.54
F: FTMS + c NSI Full ms2 667.3467@hcd30.00
[110.0000-1382.9173] MS ICIS 30_3607H
RT: 36.59
AA: 16397387
ASGIIDTLFQDR
RT: 29.40
AA: 2925933
RT: 31.56
AA: 170013
RT: 38.41
AA: 997450
NL: 1.65E6
m/z= 779.40-779.41+894.42-894.43+1007.51-1007.52
F: FTMS + c NSI Full ms2 668.3488@hcd30.00
[110.0000-1384.9616] MS ICIS 30_3607H
RT: 36.56
AA: 1282227281
ASGIIDTLFQDR[HeavyR]
RT: 27.16
AA: 2413726
RT: 33.76
AA: 1701358
RT: 37.81
AA: 287394
NL: 1.23E8
m/z= 789.40-789.42+904.43-904.44+1017.51-1017.53
F: FTMS + c NSI Full ms2 673.3515@hcd30.00
[110.0000-1395.1671] MS ICIS 30_3607H
100
50
0
26
28
30
32
34
36
38
40
Time (min)

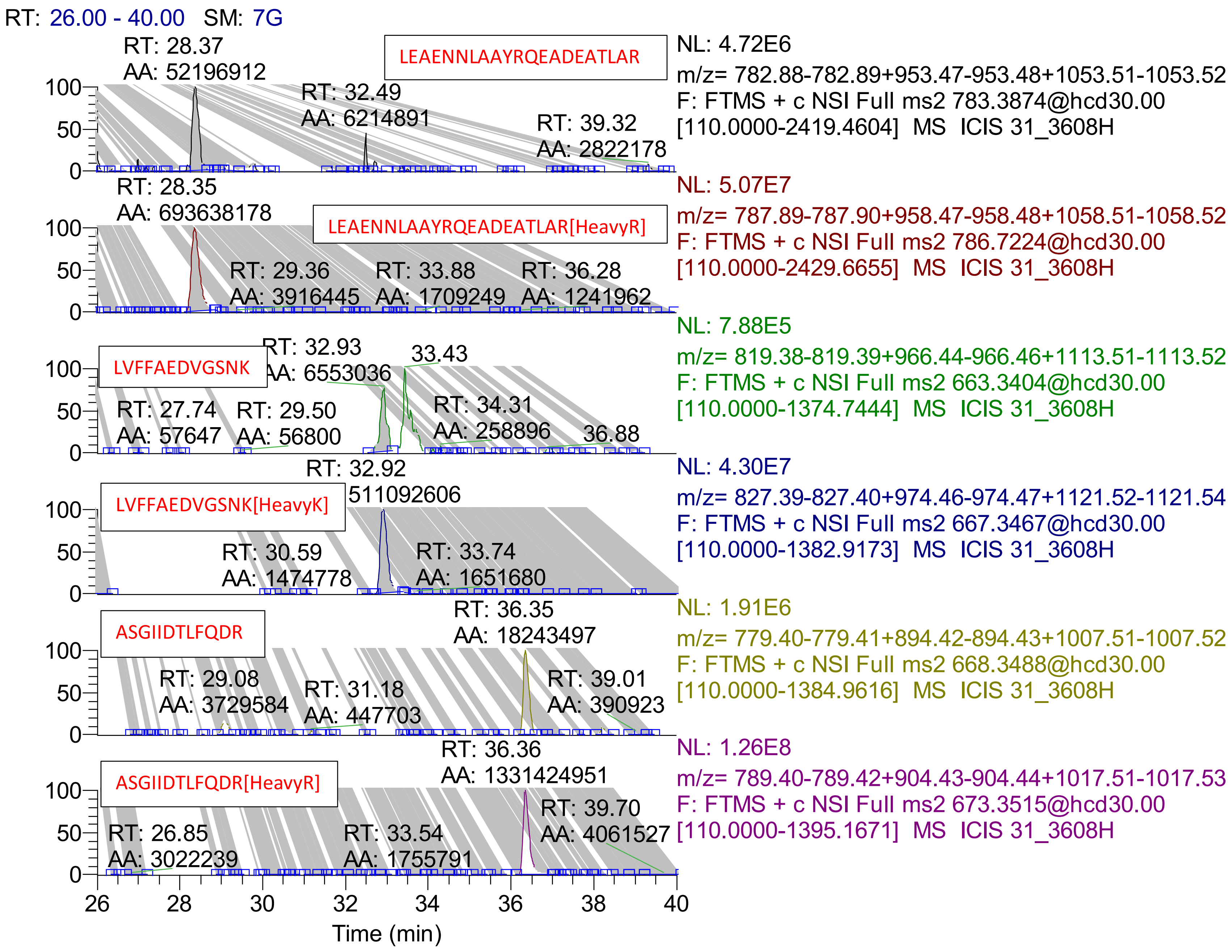

RT: 26.00 - 40.00 SM: 7G
LEAENNLAAYRQEADEATLAR
RT: 28.37
AA: 52196912
RT: 32.49
AA: 6214891
RT: 39.32
AA: 2822178
NL: 4.72E6
m/z= 782.88-782.89+953.47-953.48+1053.51-1053.52
F: FTMS + c NSI Full ms2 783.3874@hcd30.00
[110.0000-2419.4604] MS ICIS 31_3608H
LEAENNLAAYRQEADEATLAR[HeavyR]
RT: 28.35
AA: 693638178
RT: 29.36
AA: 3916445
RT: 33.88
AA: 1709249
RT: 36.28
AA: 1241962
NL: 5.07E7
m/z= 787.89-787.90+958.47-958.48+1058.51-1058.52
F: FTMS + c NSI Full ms2 786.7224@hcd30.00
[110.0000-2429.6655] MS ICIS 31_3608H
LVFFAEDVGSNK
RT: 32.93
AA: 6553036
33.43
RT: 27.74
AA: 57647
RT: 29.50
AA: 56800
RT: 34.31
AA: 258896
36.88
NL: 7.88E5
m/z= 819.38-819.39+966.44-966.46+1113.51-1113.52
F: FTMS + c NSI Full ms2 663.3404@hcd30.00
[110.0000-1374.7444] MS ICIS 31_3608H
LVFFAEDVGSNK[HeavyK]
RT: 32.92
511092606
RT: 30.59
AA: 1474778
RT: 33.74
AA: 1651680
NL: 4.30E7
m/z= 827.39-827.40+974.46-974.47+1121.52-1121.54
F: FTMS + c NSI Full ms2 667.3467@hcd30.00
[110.0000-1382.9173] MS ICIS 31_3608H
ASGIIDTLFQDR
RT: 36.35
AA: 18243497
RT: 29.08
AA: 3729584
RT: 31.18
AA: 447703
RT: 39.01
AA: 390923
NL: 1.91E6
m/z= 779.40-779.41+894.42-894.43+1007.51-1007.52
F: FTMS + c NSI Full ms2 668.3488@hcd30.00
[110.0000-1384.9616] MS ICIS 31_3608H
ASGIIDTLFQDR[HeavyR]
RT: 36.36
AA: 1331424951
RT: 26.85
AA: 3022239
RT: 33.54
AA: 1755791
RT: 39.70
AA: 4061527
NL: 1.26E8
m/z= 789.40-789.42+904.43-904.44+1017.51-1017.53
F: FTMS + c NSI Full ms2 673.3515@hcd30.00
[110.0000-1395.1671] MS ICIS 31_3608H
100
50
0
26
28
30
32
34
36
38
40
Time (min)

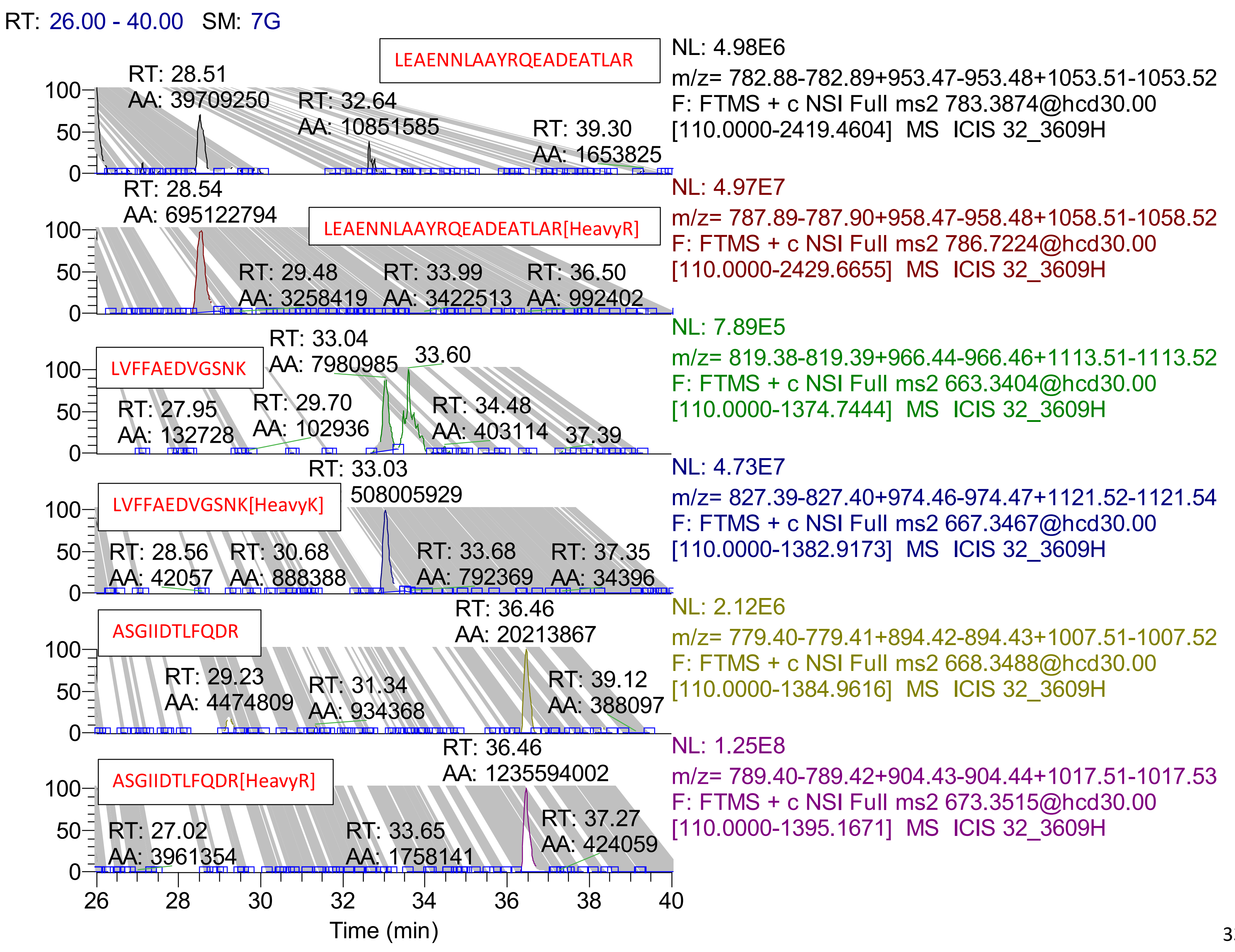

RT: 26.00 - 40.00 SM: 7G
LEAENNLAAYRQEADEATLAR
RT: 28.51
AA: 39709250
RT: 32.64
AA: 10851585
RT: 39.30
AA: 1653825
NL: 4.98E6
m/z= 782.88-782.89+953.47-953.48+1053.51-1053.52
F: FTMS + c NSI Full ms2 783.3874@hcd30.00
[110.0000-2419.4604] MS ICIS 32_3609H
RT: 28.54
AA: 695122794
LEAENNLAAYRQEADEATLAR[HeavyR]
RT: 29.48
AA: 3258419
RT: 33.99
AA: 3422513
RT: 36.50
AA: 992402
NL: 4.97E7
m/z= 787.89-787.90+958.47-958.48+1058.51-1058.52
F: FTMS + c NSI Full ms2 786.7224@hcd30.00
[110.0000-2429.6655] MS ICIS 32_3609H
LVFFAEDVGSNK
RT: 33.04
AA: 7980985
33.60
RT: 27.95
AA: 132728
RT: 29.70
AA: 102936
RT: 34.48
AA: 403114
37.39
NL: 7.89E5
m/z= 819.38-819.39+966.44-966.46+1113.51-1113.52
F: FTMS + c NSI Full ms2 663.3404@hcd30.00
[110.0000-1374.7444] MS ICIS 32_3609H
RT: 33.03
508005929
LVFFAEDVGSNK[HeavyK]
RT: 28.56
AA: 42057
RT: 30.68
AA: 888388
RT: 33.68
AA: 792369
RT: 37.35
AA: 34396
NL: 4.73E7
m/z= 827.39-827.40+974.46-974.47+1121.52-1121.54
F: FTMS + c NSI Full ms2 667.3467@hcd30.00
[110.0000-1382.9173] MS ICIS 32_3609H
ASGIIDTLFQDR
RT: 36.46
AA: 20213867
RT: 29.23
AA: 4474809
RT: 31.34
AA: 934368
RT: 39.12
AA: 388097
NL: 2.12E6
m/z= 779.40-779.41+894.42-894.43+1007.51-1007.52
F: FTMS + c NSI Full ms2 668.3488@hcd30.00
[110.0000-1384.9616] MS ICIS 32_3609H
ASGIIDTLFQDR[HeavyR]
RT: 36.46
AA: 1235594002
RT: 27.02
AA: 3961354
RT: 33.65
AA: 1758141
RT: 37.27
AA: 424059
NL: 1.25E8
m/z= 789.40-789.42+904.43-904.44+1017.51-1017.53
F: FTMS + c NSI Full ms2 673.3515@hcd30.00
[110.0000-1395.1671] MS ICIS 32_3609H
100
50
0
26 28 30 32 34 36 38 40
Time (min)

Cortex Samples

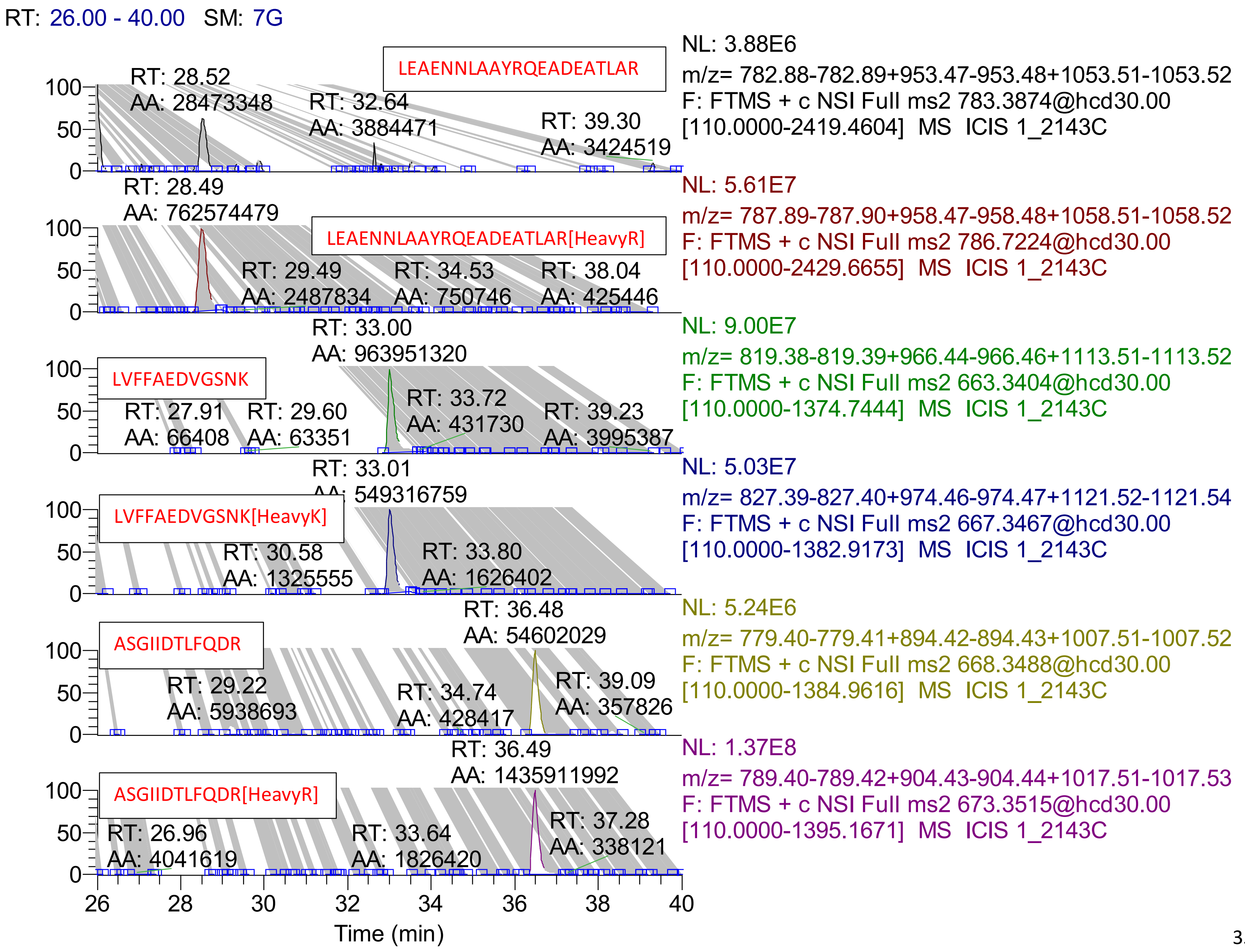

RT: 26.00 - 40.00 SM: 7G
LEAENNLAAYRQEADEATLAR
RT: 28.52
AA: 28473348
RT: 32.64
AA: 3884471
RT: 39.30
AA: 3424519
NL: 3.88E6
m/z= 782.88-782.89+953.47-953.48+1053.51-1053.52
F: FTMS + c NSI Full ms2 783.3874@hcd30.00
[110.0000-2419.4604] MS ICIS 1_2143C
RT: 28.49
AA: 762574479
LEAENNLAAYRQEADEATLAR[HeavyR]
RT: 29.49
AA: 2487834
RT: 34.53
AA: 750746
RT: 38.04
AA: 425446
NL: 5.61E7
m/z= 787.89-787.90+958.47-958.48+1058.51-1058.52
F: FTMS + c NSI Full ms2 786.7224@hcd30.00
[110.0000-2429.6655] MS ICIS 1_2143C
RT: 33.00
AA: 963951320
LVFFAEDVGSNK
RT: 27.91
AA: 66408
RT: 29.60
AA: 63351
RT: 33.72
AA: 431730
RT: 39.23
AA: 3995387
NL: 9.00E7
m/z= 819.38-819.39+966.44-966.46+1113.51-1113.52
F: FTMS + c NSI Full ms2 663.3404@hcd30.00
[110.0000-1374.7444] MS ICIS 1_2143C
RT: 33.01
AA: 549316759
LVFFAEDVGSNK[HeavyK]
RT: 30.58
AA: 1325555
RT: 33.80
AA: 1626402
NL: 5.03E7
m/z= 827.39-827.40+974.46-974.47+1121.52-1121.54
F: FTMS + c NSI Full ms2 667.3467@hcd30.00
[110.0000-1382.9173] MS ICIS 1_2143C
RT: 36.48
AA: 54602029
ASGIIDTLFQDR
RT: 29.22
AA: 5938693
RT: 34.74
AA: 428417
RT: 39.09
AA: 357826
NL: 5.24E6
m/z= 779.40-779.41+894.42-894.43+1007.51-1007.52
F: FTMS + c NSI Full ms2 668.3488@hcd30.00
[110.0000-1384.9616] MS ICIS 1_2143C
RT: 36.49
AA: 1435911992
ASGIIDTLFQDR[HeavyR]
RT: 26.96
AA: 4041619
RT: 33.64
AA: 1826420
RT: 37.28
AA: 338121
NL: 1.37E8
m/z= 789.40-789.42+904.43-904.44+1017.51-1017.53
F: FTMS + c NSI Full ms2 673.3515@hcd30.00
[110.0000-1395.1671] MS ICIS 1_2143C
100
50
0
26 28 30 32 34 36 38 40
Time (min)

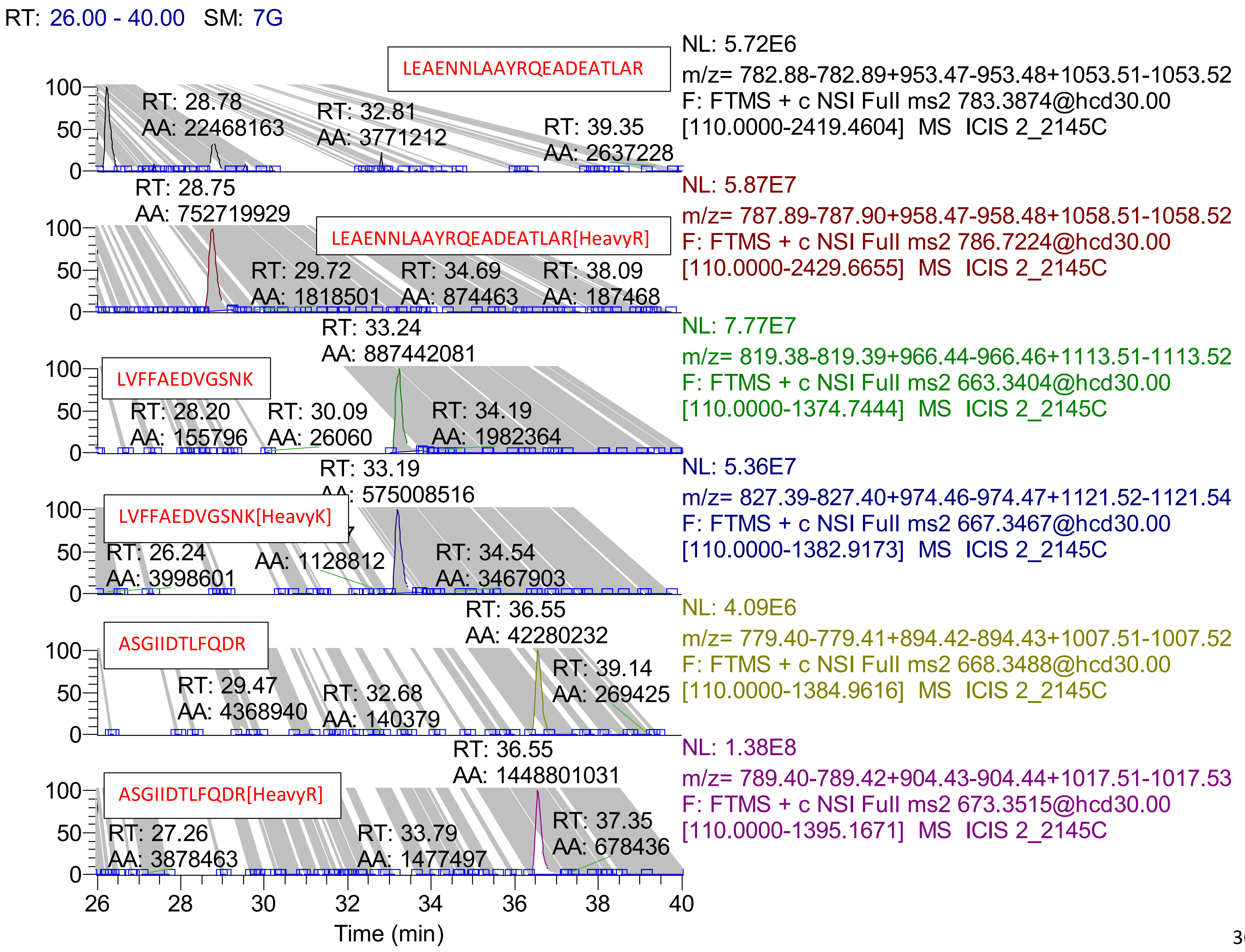

RT: 26.00 - 40.00 SM: 7G
LEAENNLAAYRQEADEATLAR
NL: 5.72E6
m/z= 782.88-782.89+953.47-953.48+1053.51-1053.52
F: FTMS + c NSI Full ms2 783.3874@hcd30.00
[110.0000-2419.4604] MS ICIS 2_2145C
RT: 28.78
AA: 22468163
RT: 32.81
AA: 3771212
RT: 39.35
AA: 2637228
RT: 28.75
AA: 752719929
LEAENNLAAYRQEADEATLAR[HeavyR]
NL: 5.87E7
m/z= 787.89-787.90+958.47-958.48+1058.51-1058.52
F: FTMS + c NSI Full ms2 786.7224@hcd30.00
[110.0000-2429.6655] MS ICIS 2_2145C
RT: 29.72
AA: 1818501
RT: 34.69
AA: 874463
RT: 38.09
AA: 187468
RT: 33.24
AA: 887442081
LVFFAEDVGSNK
NL: 7.77E7
m/z= 819.38-819.39+966.44-966.46+1113.51-1113.52
F: FTMS + c NSI Full ms2 663.3404@hcd30.00
[110.0000-1374.7444] MS ICIS 2_2145C
RT: 28.20
AA: 155796
RT: 30.09
AA: 26060
RT: 34.19
AA: 1982364
RT: 33.19
AA: 575008516
LVFFAEDVGSNK[HeavyK]
NL: 5.36E7
m/z= 827.39-827.40+974.46-974.47+1121.52-1121.54
F: FTMS + c NSI Full ms2 667.3467@hcd30.00
[110.0000-1382.9173] MS ICIS 2_2145C
RT: 26.24
AA: 3998601
AA: 1128812
RT: 34.54
AA: 3467903
RT: 36.55
AA: 42280232
ASGIIDTLFQDR
NL: 4.09E6
m/z= 779.40-779.41+894.42-894.43+1007.51-1007.52
F: FTMS + c NSI Full ms2 668.3488@hcd30.00
[110.0000-1384.9616] MS ICIS 2_2145C
RT: 29.47
AA: 4368940
RT: 32.68
AA: 140379
RT: 39.14
AA: 269425
RT: 36.55
AA: 1448801031
ASGIIDTLFQDR[HeavyR]
NL: 1.38E8
m/z= 789.40-789.42+904.43-904.44+1017.51-1017.53
F: FTMS + c NSI Full ms2 673.3515@hcd30.00
[110.0000-1395.1671] MS ICIS 2_2145C
RT: 27.26
AA: 3878463
RT: 33.79
AA: 1477497
RT: 37.35
AA: 678436
100
50
0
26
28
30
32
34
36
38
40
Time (min)

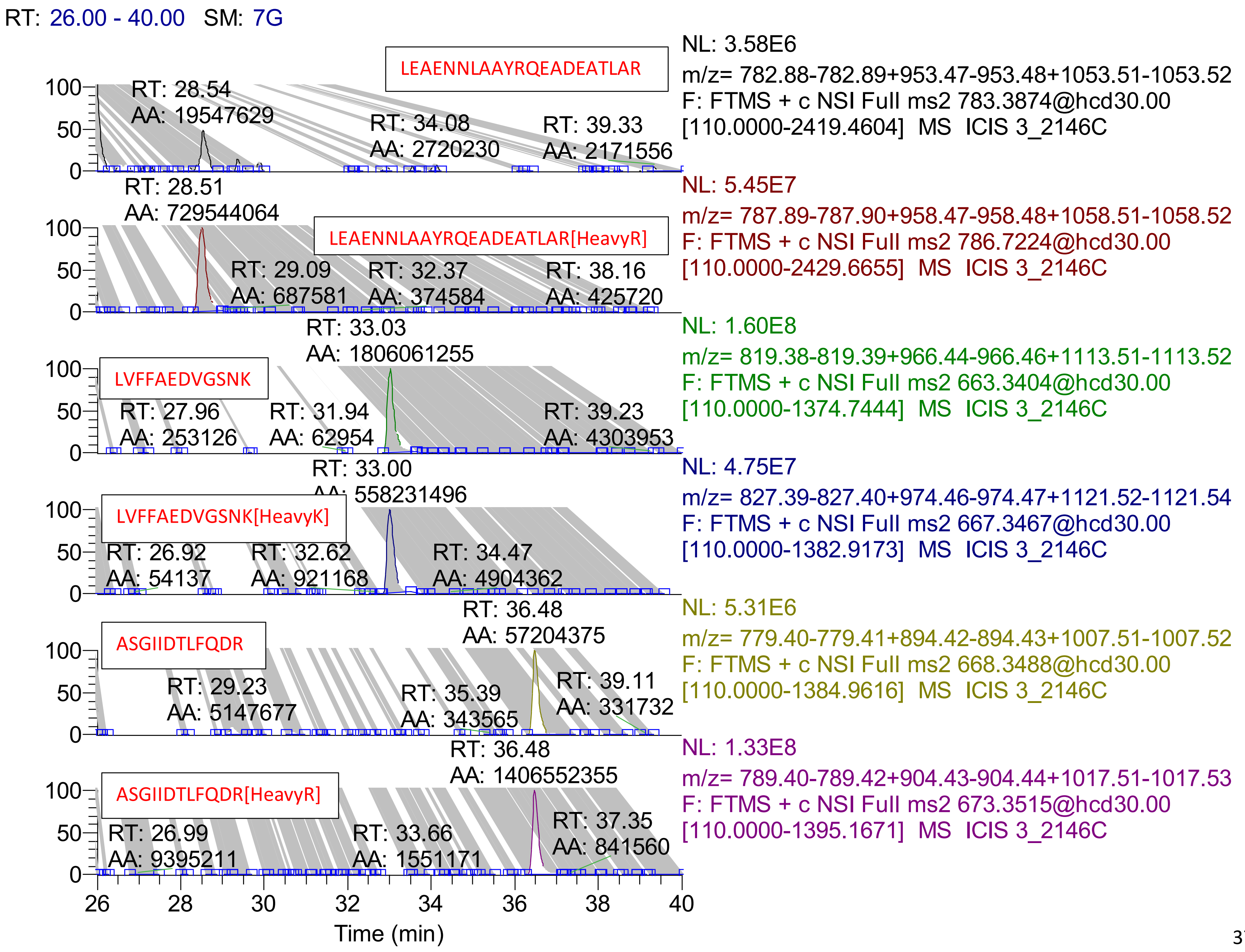
RT: 26.00 - 40.00 SM: 7G
LEAENNLAAYRQEADEATLAR
RT: 28.54
AA: 19547629
RT: 34.08
AA: 2720230
RT: 39.33
AA: 2171556
NL: 3.58E6
m/z= 782.88-782.89+953.47-953.48+1053.51-1053.52
F: FTMS + c NSI Full ms2 783.3874@hcd30.00
[110.0000-2419.4604] MS ICIS 3_2146C
RT: 28.51
AA: 729544064
LEAENNLAAYRQEADEATLAR[HeavyR]
RT: 29.09
AA: 687581
RT: 32.37
AA: 374584
RT: 38.16
AA: 425720
NL: 5.45E7
m/z= 787.89-787.90+958.47-958.48+1058.51-1058.52
F: FTMS + c NSI Full ms2 786.7224@hcd30.00
[110.0000-2429.6655] MS ICIS 3_2146C
RT: 33.03
AA: 1806061255
LVFFAEDVGSNK
RT: 27.96
AA: 253126
RT: 31.94
AA: 62954
RT: 39.23
AA: 4303953
NL: 1.60E8
m/z= 819.38-819.39+966.44-966.46+1113.51-1113.52
F: FTMS + c NSI Full ms2 663.3404@hcd30.00
[110.0000-1374.7444] MS ICIS 3_2146C
RT: 33.00
AA: 558231496
LVFFAEDVGSNK[HeavyK]
RT: 26.92
AA: 54137
RT: 32.62
AA: 921168
RT: 34.47
AA: 4904362
NL: 4.75E7
m/z= 827.39-827.40+974.46-974.47+1121.52-1121.54
F: FTMS + c NSI Full ms2 667.3467@hcd30.00
[110.0000-1382.9173] MS ICIS 3_2146C
RT: 36.48
AA: 57204375
ASGIIDTLFQDR
RT: 29.23
AA: 5147677
RT: 35.39
AA: 343565
RT: 39.11
AA: 331732
NL: 5.31E6
m/z= 779.40-779.41+894.42-894.43+1007.51-1007.52
F: FTMS + c NSI Full ms2 668.3488@hcd30.00
[110.0000-1384.9616] MS ICIS 3_2146C
RT: 36.48
AA: 1406552355
ASGIIDTLFQDR[HeavyR]
RT: 26.99
AA: 9395211
RT: 33.66
AA: 1551171
RT: 37.35
AA: 841560
NL: 1.33E8
m/z= 789.40-789.42+904.43-904.44+1017.51-1017.53
F: FTMS + c NSI Full ms2 673.3515@hcd30.00
[110.0000-1395.1671] MS ICIS 3_2146C
100
50
0
26
28
30
32
34
36
38
40
Time (min)

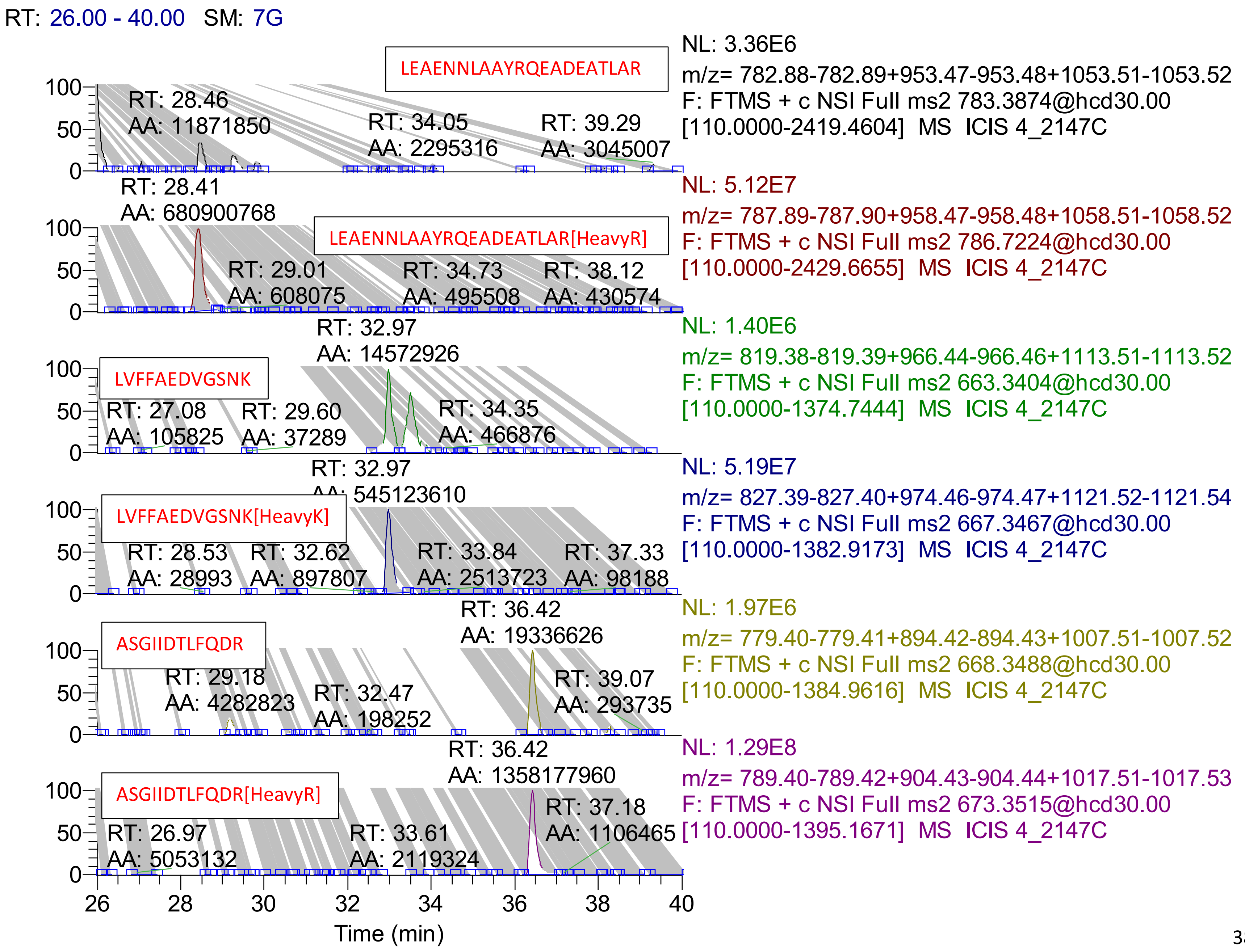
RT: 26.00 - 40.00 SM: 7G
LEAENNLAAYRQEADEATLAR
NL: 3.36E6
m/z= 782.88-782.89+953.47-953.48+1053.51-1053.52
F: FTMS + c NSI Full ms2 783.3874@hcd30.00
[110.0000-2419.4604] MS ICIS 4_2147C
RT: 28.46
AA: 11871850
RT: 34.05
AA: 2295316
RT: 39.29
AA: 3045007
RT: 28.41
AA: 680900768
LEAENNLAAYRQEADEATLAR[HeavyR]
NL: 5.12E7
m/z= 787.89-787.90+958.47-958.48+1058.51-1058.52
F: FTMS + c NSI Full ms2 786.7224@hcd30.00
[110.0000-2429.6655] MS ICIS 4_2147C
RT: 29.01
AA: 608075
RT: 34.73
AA: 495508
RT: 38.12
AA: 430574
RT: 32.97
AA: 14572926
LVFFAEDVGSNK
NL: 1.40E6
m/z= 819.38-819.39+966.44-966.46+1113.51-1113.52
F: FTMS + c NSI Full ms2 663.3404@hcd30.00
[110.0000-1374.7444] MS ICIS 4_2147C
RT: 27.08
AA: 105825
RT: 29.60
AA: 37289
RT: 34.35
AA: 466876
RT: 32.97
AA: 545123610
LVFFAEDVGSNK[HeavyK]
NL: 5.19E7
m/z= 827.39-827.40+974.46-974.47+1121.52-1121.54
F: FTMS + c NSI Full ms2 667.3467@hcd30.00
[110.0000-1382.9173] MS ICIS 4_2147C
RT: 28.53
AA: 28993
RT: 32.62
AA: 897807
RT: 33.84
AA: 2513723
RT: 37.33
AA: 98188
RT: 36.42
AA: 19336626
ASGIIDTLFQDR
NL: 1.97E6
m/z= 779.40-779.41+894.42-894.43+1007.51-1007.52
F: FTMS + c NSI Full ms2 668.3488@hcd30.00
[110.0000-1384.9616] MS ICIS 4_2147C
RT: 29.18
AA: 4282823
RT: 32.47
AA: 198252
RT: 39.07
AA: 293735
RT: 36.42
AA: 1358177960
ASGIIDTLFQDR[HeavyR]
NL: 1.29E8
m/z= 789.40-789.42+904.43-904.44+1017.51-1017.53
F: FTMS + c NSI Full ms2 673.3515@hcd30.00
[110.0000-1395.1671] MS ICIS 4_2147C
RT: 26.97
AA: 5053132
RT: 33.61
AA: 2119324
RT: 37.18
AA: 1106465
100
50
0
26
28
30
32
34
36
38
40
Time (min)

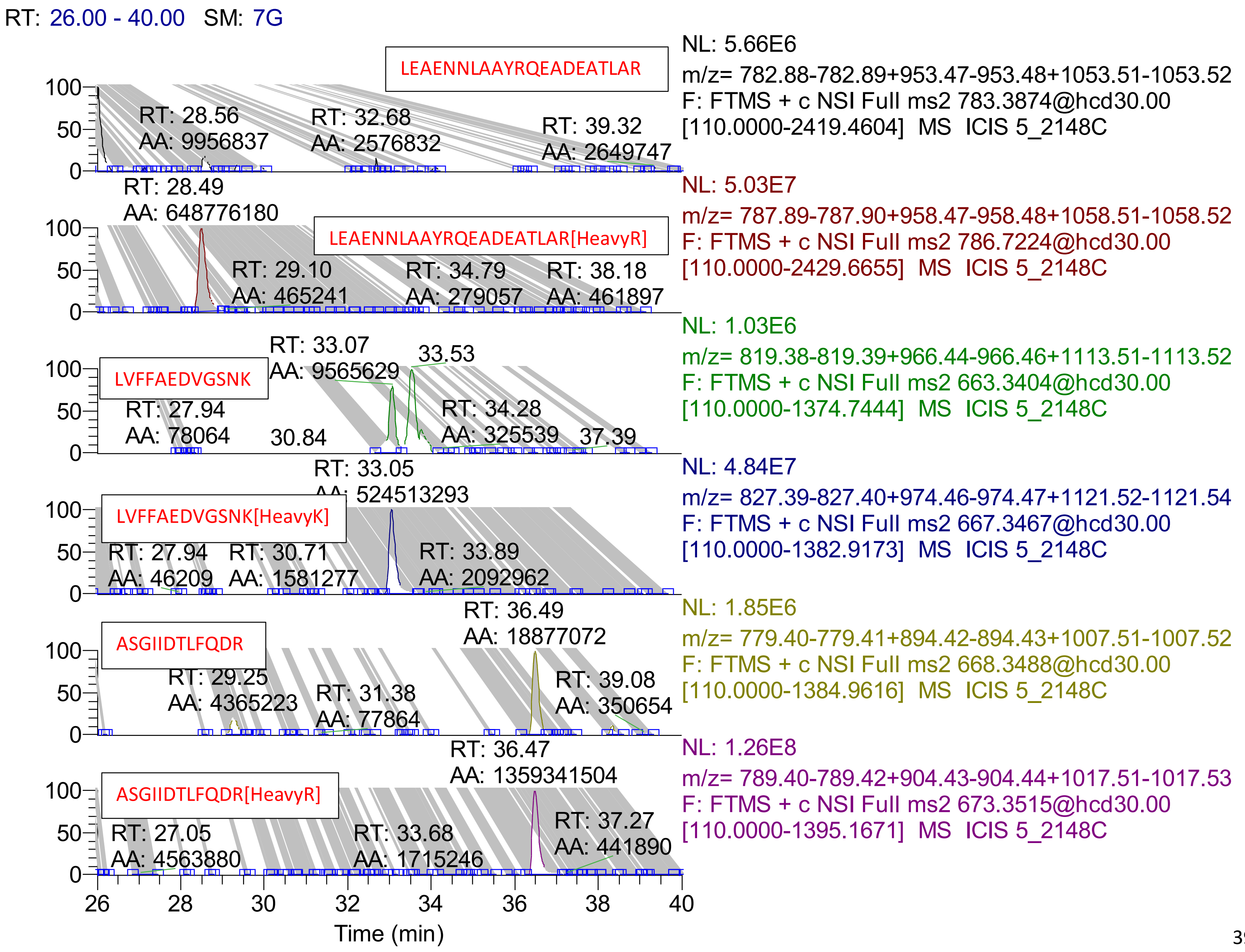
RT: 26.00 - 40.00 SM: 7G
LEAENNLAAYRQEADEATLAR
RT: 28.56
AA: 9956837
RT: 32.68
AA: 2576832
RT: 39.32
AA: 2649747
NL: 5.66E6
m/z= 782.88-782.89+953.47-953.48+1053.51-1053.52
F: FTMS + c NSI Full ms2 783.3874@hcd30.00
[110.0000-2419.4604] MS ICIS 5_2148C
RT: 28.49
AA: 648776180
LEAENNLAAYRQEADEATLAR[HeavyR]
RT: 29.10
AA: 465241
RT: 34.79
AA: 279057
RT: 38.18
AA: 461897
NL: 5.03E7
m/z= 787.89-787.90+958.47-958.48+1058.51-1058.52
F: FTMS + c NSI Full ms2 786.7224@hcd30.00
[110.0000-2429.6655] MS ICIS 5_2148C
LVFFAEDVGSNK
RT: 33.07
AA: 9565629
33.53
RT: 27.94
AA: 78064
30.84
RT: 34.28
AA: 325539
37.39
NL: 1.03E6
m/z= 819.38-819.39+966.44-966.46+1113.51-1113.52
F: FTMS + c NSI Full ms2 663.3404@hcd30.00
[110.0000-1374.7444] MS ICIS 5_2148C
RT: 33.05
AA: 524513293
LVFFAEDVGSNK[HeavyK]
RT: 27.94
AA: 46209
RT: 30.71
AA: 1581277
RT: 33.89
AA: 2092962
NL: 4.84E7
m/z= 827.39-827.40+974.46-974.47+1121.52-1121.54
F: FTMS + c NSI Full ms2 667.3467@hcd30.00
[110.0000-1382.9173] MS ICIS 5_2148C
ASGIIDTLFQDR
RT: 36.49
AA: 18877072
RT: 29.25
AA: 4365223
RT: 31.38
AA: 77864
RT: 39.08
AA: 350654
NL: 1.85E6
m/z= 779.40-779.41+894.42-894.43+1007.51-1007.52
F: FTMS + c NSI Full ms2 668.3488@hcd30.00
[110.0000-1384.9616] MS ICIS 5_2148C
ASGIIDTLFQDR[HeavyR]
RT: 36.47
AA: 1359341504
RT: 27.05
AA: 4563880
RT: 33.68
AA: 1715246
RT: 37.27
AA: 441890
NL: 1.26E8
m/z= 789.40-789.42+904.43-904.44+1017.51-1017.53
F: FTMS + c NSI Full ms2 673.3515@hcd30.00
[110.0000-1395.1671] MS ICIS 5_2148C
100
50
0
26
28
30
32
34
36
38
40
Time (min)

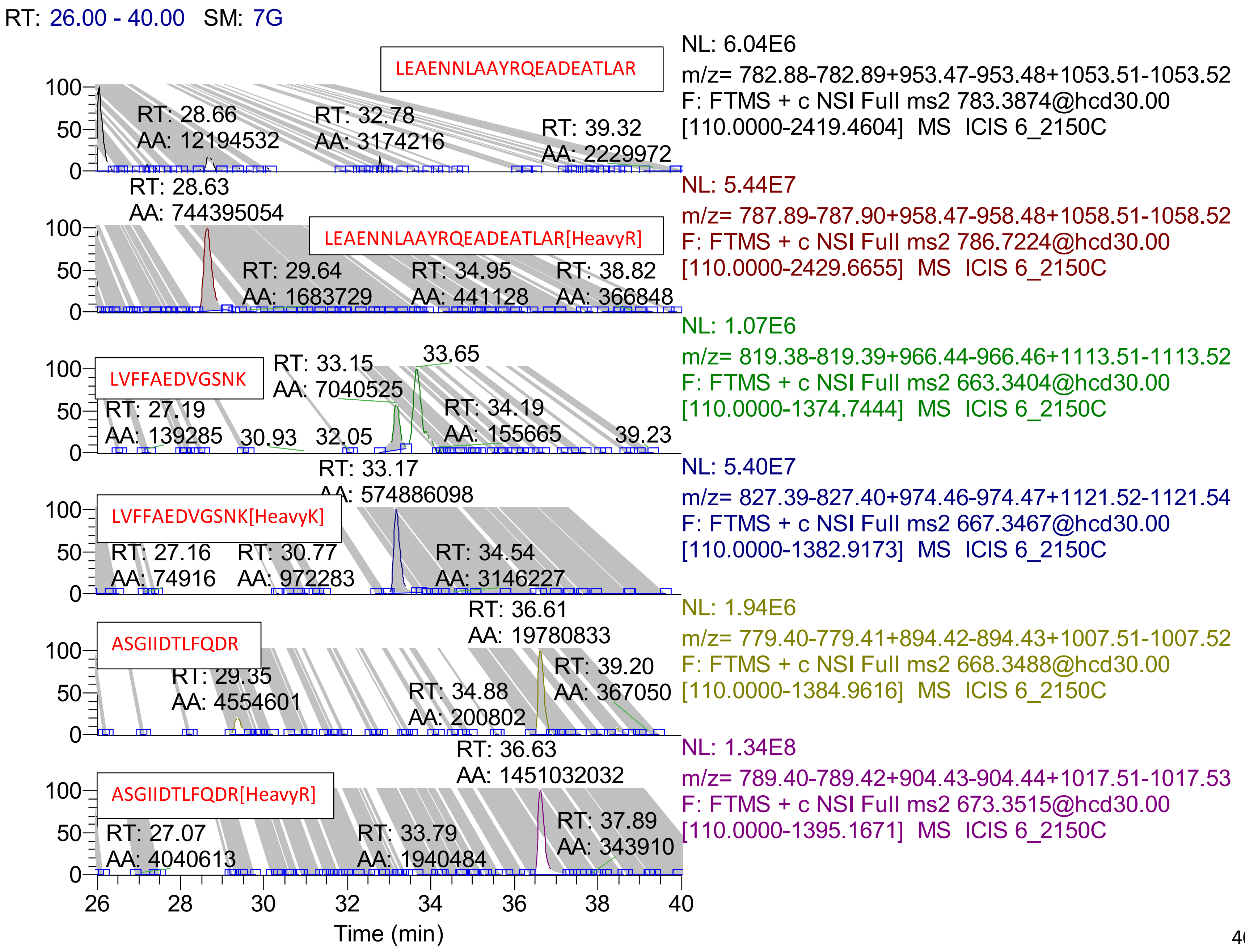

RT: 26.00 - 40.00 SM: 7G
LEAENNLAAYRQEADEATLAR
RT: 28.66
AA: 12194532
RT: 32.78
AA: 3174216
RT: 39.32
AA: 2229972
NL: 6.04E6
m/z= 782.88-782.89+953.47-953.48+1053.51-1053.52
F: FTMS + c NSI Full ms2 783.3874@hcd30.00
[110.0000-2419.4604] MS ICIS 6_2150C
RT: 28.63
AA: 744395054
LEAENNLAAYRQEADEATLAR[HeavyR]
RT: 29.64
AA: 1683729
RT: 34.95
AA: 441128
RT: 38.82
AA: 366848
NL: 5.44E7
m/z= 787.89-787.90+958.47-958.48+1058.51-1058.52
F: FTMS + c NSI Full ms2 786.7224@hcd30.00
[110.0000-2429.6655] MS ICIS 6_2150C
LVFFAEDVGSNK
RT: 33.15
AA: 7040525
33.65
RT: 27.19
AA: 139285
30.93
32.05
RT: 34.19
AA: 155665
39.23
NL: 1.07E6
m/z= 819.38-819.39+966.44-966.46+1113.51-1113.52
F: FTMS + c NSI Full ms2 663.3404@hcd30.00
[110.0000-1374.7444] MS ICIS 6_2150C
RT: 33.17
AA: 574886098
LVFFAEDVGSNK[HeavyK]
RT: 27.16
AA: 74916
RT: 30.77
AA: 972283
RT: 34.54
AA: 3146227
NL: 5.40E7
m/z= 827.39-827.40+974.46-974.47+1121.52-1121.54
F: FTMS + c NSI Full ms2 667.3467@hcd30.00
[110.0000-1382.9173] MS ICIS 6_2150C
RT: 36.61
AA: 19780833
ASGIIDTLFQDR
RT: 29.35
AA: 4554601
RT: 34.88
AA: 200802
RT: 39.20
AA: 367050
NL: 1.94E6
m/z= 779.40-779.41+894.42-894.43+1007.51-1007.52
F: FTMS + c NSI Full ms2 668.3488@hcd30.00
[110.0000-1384.9616] MS ICIS 6_2150C
RT: 36.63
AA: 1451032032
ASGIIDTLFQDR[HeavyR]
RT: 27.07
AA: 4040613
RT: 33.79
AA: 1940484
RT: 37.89
AA: 343910
NL: 1.34E8
m/z= 789.40-789.42+904.43-904.44+1017.51-1017.53
F: FTMS + c NSI Full ms2 673.3515@hcd30.00
[110.0000-1395.1671] MS ICIS 6_2150C
100
50
0
26 28 30 32 34 36 38 40
Time (min)

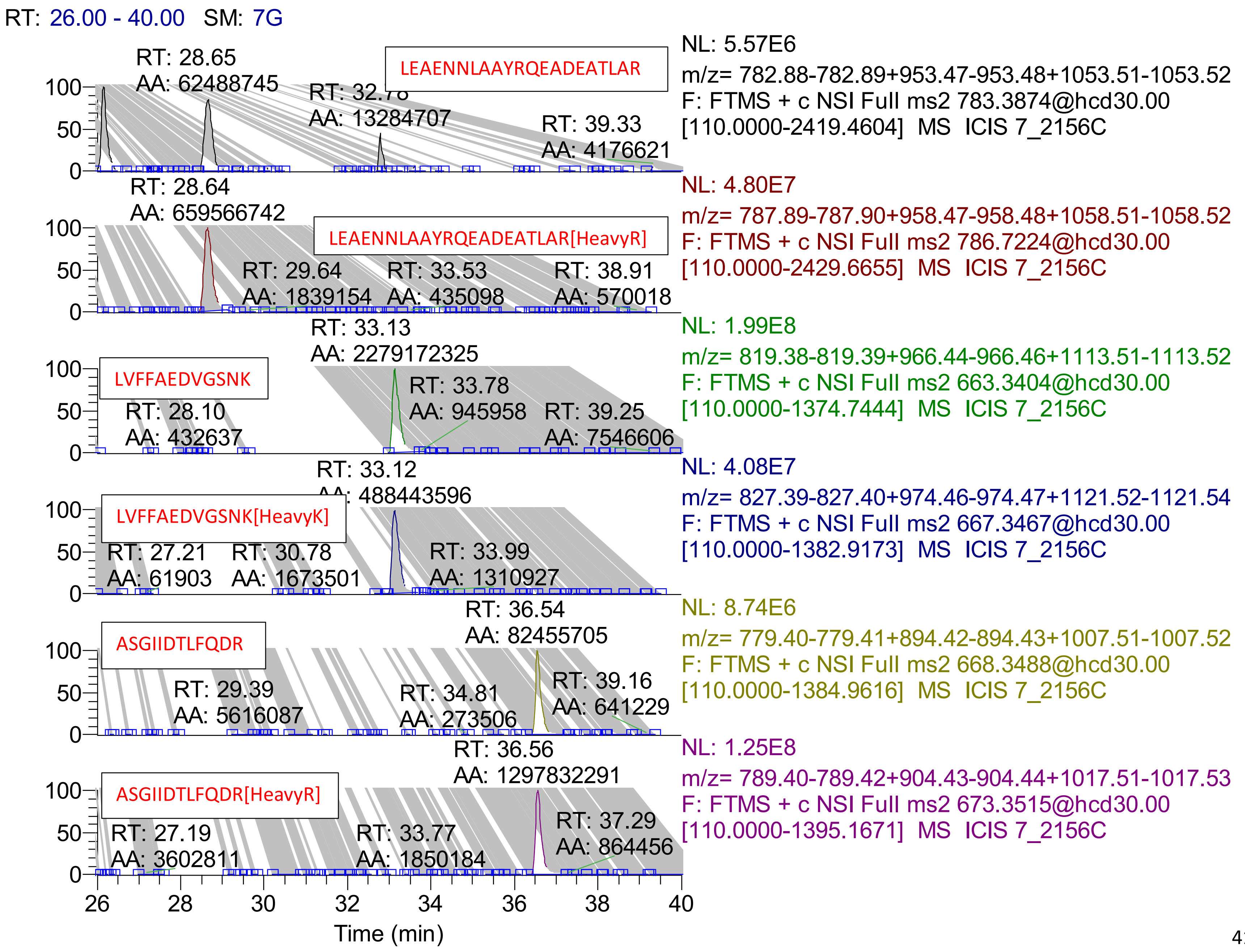
RT: 26.00 - 40.00 SM: 7G
RT: 28.65
AA: 62488745
RT: 32.78
AA: 13284707
RT: 39.33
AA: 4176621
LEAENNLAAYRQEADEATLAR
NL: 5.57E6
m/z= 782.88-782.89+953.47-953.48+1053.51-1053.52
F: FTMS + c NSI Full ms2 783.3874@hcd30.00
[110.0000-2419.4604] MS ICIS 7_2156C
RT: 28.64
AA: 659566742
LEAENNLAAYRQEADEATLAR[HeavyR]
RT: 29.64
AA: 1839154
RT: 33.53
AA: 435098
RT: 38.91
AA: 570018
NL: 4.80E7
m/z= 787.89-787.90+958.47-958.48+1058.51-1058.52
F: FTMS + c NSI Full ms2 786.7224@hcd30.00
[110.0000-2429.6655] MS ICIS 7_2156C
RT: 33.13
AA: 2279172325
LVFFAEDVGSNK
RT: 28.10
AA: 432637
RT: 33.78
AA: 945958
RT: 39.25
AA: 7546606
NL: 1.99E8
m/z= 819.38-819.39+966.44-966.46+1113.51-1113.52
F: FTMS + c NSI Full ms2 663.3404@hcd30.00
[110.0000-1374.7444] MS ICIS 7_2156C
RT: 33.12
AA: 488443596
LVFFAEDVGSNK[HeavyK]
RT: 27.21
AA: 61903
RT: 30.78
AA: 1673501
RT: 33.99
AA: 1310927
NL: 4.08E7
m/z= 827.39-827.40+974.46-974.47+1121.52-1121.54
F: FTMS + c NSI Full ms2 667.3467@hcd30.00
[110.0000-1382.9173] MS ICIS 7_2156C
RT: 36.54
AA: 82455705
ASGIIDTLFQDR
RT: 29.39
AA: 5616087
RT: 34.81
AA: 273506
RT: 39.16
AA: 641229
NL: 8.74E6
m/z= 779.40-779.41+894.42-894.43+1007.51-1007.52
F: FTMS + c NSI Full ms2 668.3488@hcd30.00
[110.0000-1384.9616] MS ICIS 7_2156C
RT: 36.56
AA: 1297832291
ASGIIDTLFQDR[HeavyR]
RT: 27.19
AA: 3602811
RT: 33.77
AA: 1850184
RT: 37.29
AA: 864456
NL: 1.25E8
m/z= 789.40-789.42+904.43-904.44+1017.51-1017.53
F: FTMS + c NSI Full ms2 673.3515@hcd30.00
[110.0000-1395.1671] MS ICIS 7_2156C
100
50
0
26
28
30
32
34
36
38
40
Time (min)

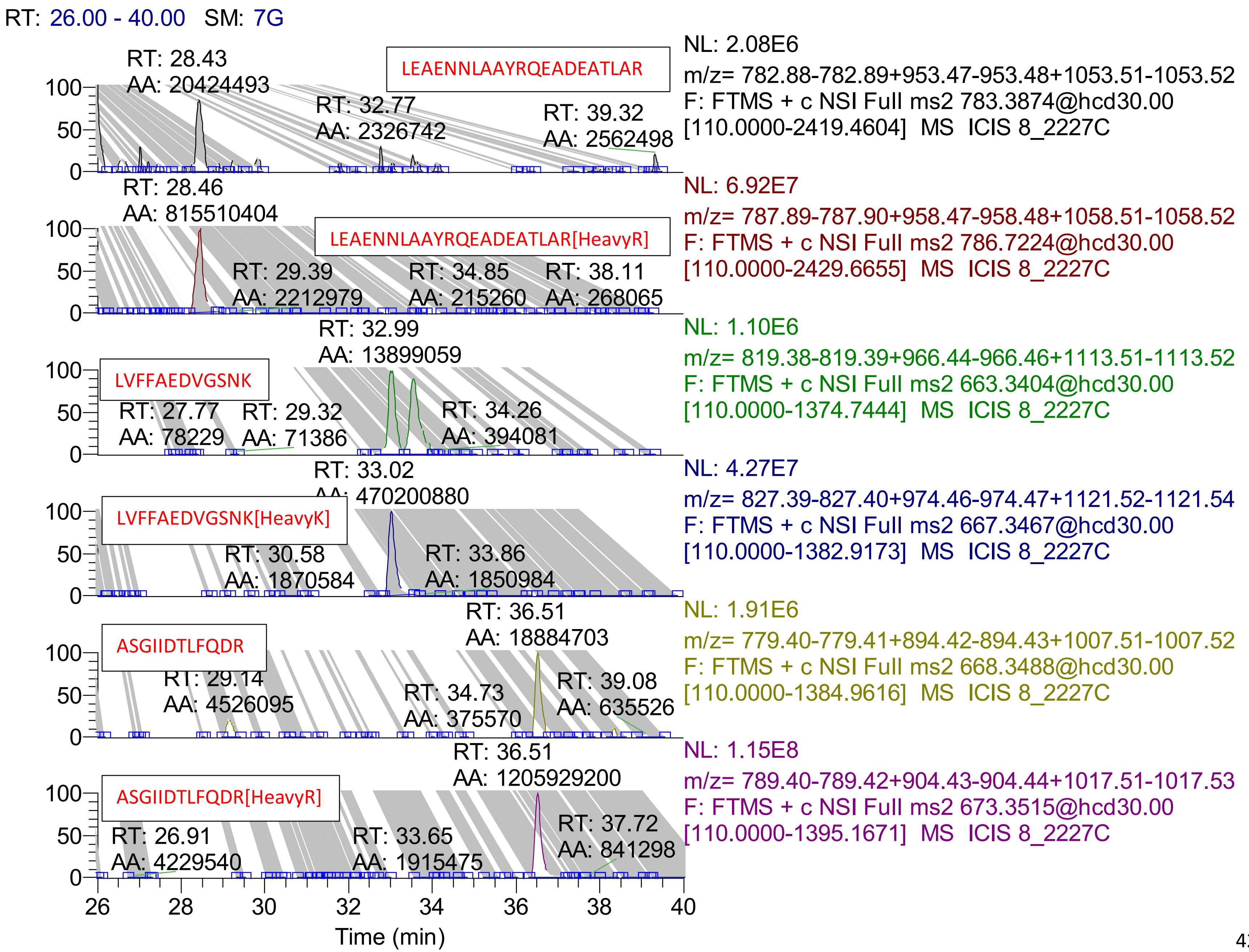
RT: 26.00 - 40.00 SM: 7G
RT: 28.43
AA: 20424493
LEAENNLAAYRQEADEATLAR
RT: 32.77
AA: 2326742
RT: 39.32
AA: 2562498
NL: 2.08E6
m/z= 782.88-782.89+953.47-953.48+1053.51-1053.52
F: FTMS + c NSI Full ms2 783.3874@hcd30.00
[110.0000-2419.4604] MS ICIS 8_2227C
RT: 28.46
AA: 815510404
LEAENNLAAYRQEADEATLAR[HeavyR]
RT: 29.39
AA: 2212979
RT: 34.85
AA: 215260
RT: 38.11
AA: 268065
NL: 6.92E7
m/z= 787.89-787.90+958.47-958.48+1058.51-1058.52
F: FTMS + c NSI Full ms2 786.7224@hcd30.00
[110.0000-2429.6655] MS ICIS 8_2227C
RT: 32.99
AA: 13899059
LVFFAEDVGSNK
RT: 27.77
AA: 78229
RT: 29.32
AA: 71386
RT: 34.26
AA: 394081
NL: 1.10E6
m/z= 819.38-819.39+966.44-966.46+1113.51-1113.52
F: FTMS + c NSI Full ms2 663.3404@hcd30.00
[110.0000-1374.7444] MS ICIS 8_2227C
RT: 33.02
AA: 470200880
LVFFAEDVGSNK[HeavyK]
RT: 30.58
AA: 1870584
RT: 33.86
AA: 1850984
NL: 4.27E7
m/z= 827.39-827.40+974.46-974.47+1121.52-1121.54
F: FTMS + c NSI Full ms2 667.3467@hcd30.00
[110.0000-1382.9173] MS ICIS 8_2227C
RT: 36.51
AA: 18884703
ASGIIDTLFQDR
RT: 29.14
AA: 4526095
RT: 34.73
AA: 375570
RT: 39.08
AA: 635526
NL: 1.91E6
m/z= 779.40-779.41+894.42-894.43+1007.51-1007.52
F: FTMS + c NSI Full ms2 668.3488@hcd30.00
[110.0000-1384.9616] MS ICIS 8_2227C
RT: 36.51
AA: 1205929200
ASGIIDTLFQDR[HeavyR]
RT: 26.91
AA: 4229540
RT: 33.65
AA: 1915475
RT: 37.72
AA: 841298
NL: 1.15E8
m/z= 789.40-789.42+904.43-904.44+1017.51-1017.53
F: FTMS + c NSI Full ms2 673.3515@hcd30.00
[110.0000-1395.1671] MS ICIS 8_2227C
100
50
0
26 28 30 32 34 36 38 40
Time (min)

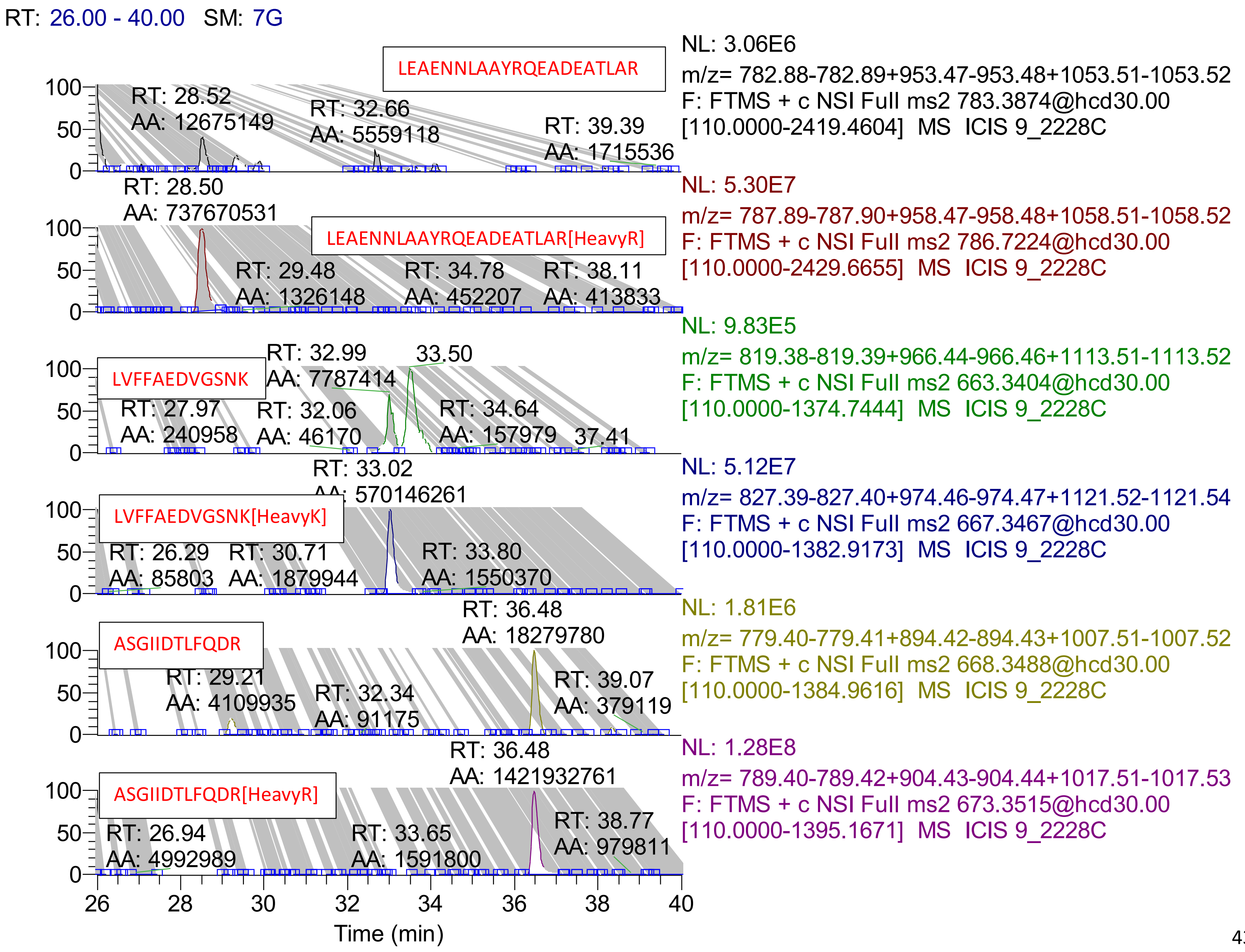

RT: 26.00 - 40.00 SM: 7G
LEAENNLAAYRQEADEATLAR
RT: 28.52 AA: 12675149
RT: 32.66 AA: 5559118
RT: 39.39 AA: 1715536
NL: 3.06E6
m/z= 782.88-782.89+953.47-953.48+1053.51-1053.52
F: FTMS + c NSI Full ms2 783.3874@hcd30.00
[110.0000-2419.4604] MS ICIS 9_2228C
RT: 28.50 AA: 737670531
LEAENNLAAYRQEADEATLAR[HeavyR]
RT: 29.48 AA: 1326148
RT: 34.78 AA: 452207
RT: 38.11 AA: 413833
NL: 5.30E7
m/z= 787.89-787.90+958.47-958.48+1058.51-1058.52
F: FTMS + c NSI Full ms2 786.7224@hcd30.00
[110.0000-2429.6655] MS ICIS 9_2228C
LVFFAEDVGSNK
RT: 32.99 AA: 7787414
33.50
RT: 27.97 AA: 240958
RT: 32.06 AA: 46170
RT: 34.64 AA: 157979
37.41
NL: 9.83E5
m/z= 819.38-819.39+966.44-966.46+1113.51-1113.52
F: FTMS + c NSI Full ms2 663.3404@hcd30.00
[110.0000-1374.7444] MS ICIS 9_2228C
RT: 33.02 AA: 570146261
LVFFAEDVGSNK[HeavyK]
RT: 26.29 AA: 85803
RT: 30.71 AA: 1879944
RT: 33.80 AA: 1550370
NL: 5.12E7
m/z= 827.39-827.40+974.46-974.47+1121.52-1121.54
F: FTMS + c NSI Full ms2 667.3467@hcd30.00
[110.0000-1382.9173] MS ICIS 9_2228C
RT: 36.48 AA: 18279780
ASGIIDTLFQDR
RT: 29.21 AA: 4109935
RT: 32.34 AA: 91175
RT: 39.07 AA: 379119
NL: 1.81E6
m/z= 779.40-779.41+894.42-894.43+1007.51-1007.52
F: FTMS + c NSI Full ms2 668.3488@hcd30.00
[110.0000-1384.9616] MS ICIS 9_2228C
RT: 36.48 AA: 1421932761
ASGIIDTLFQDR[HeavyR]
RT: 26.94 AA: 4992989
RT: 33.65 AA: 1591800
RT: 38.77 AA: 979811
NL: 1.28E8
m/z= 789.40-789.42+904.43-904.44+1017.51-1017.53
F: FTMS + c NSI Full ms2 673.3515@hcd30.00
[110.0000-1395.1671] MS ICIS 9_2228C
100
50
0
26 28 30 32 34 36 38 40
Time (min)

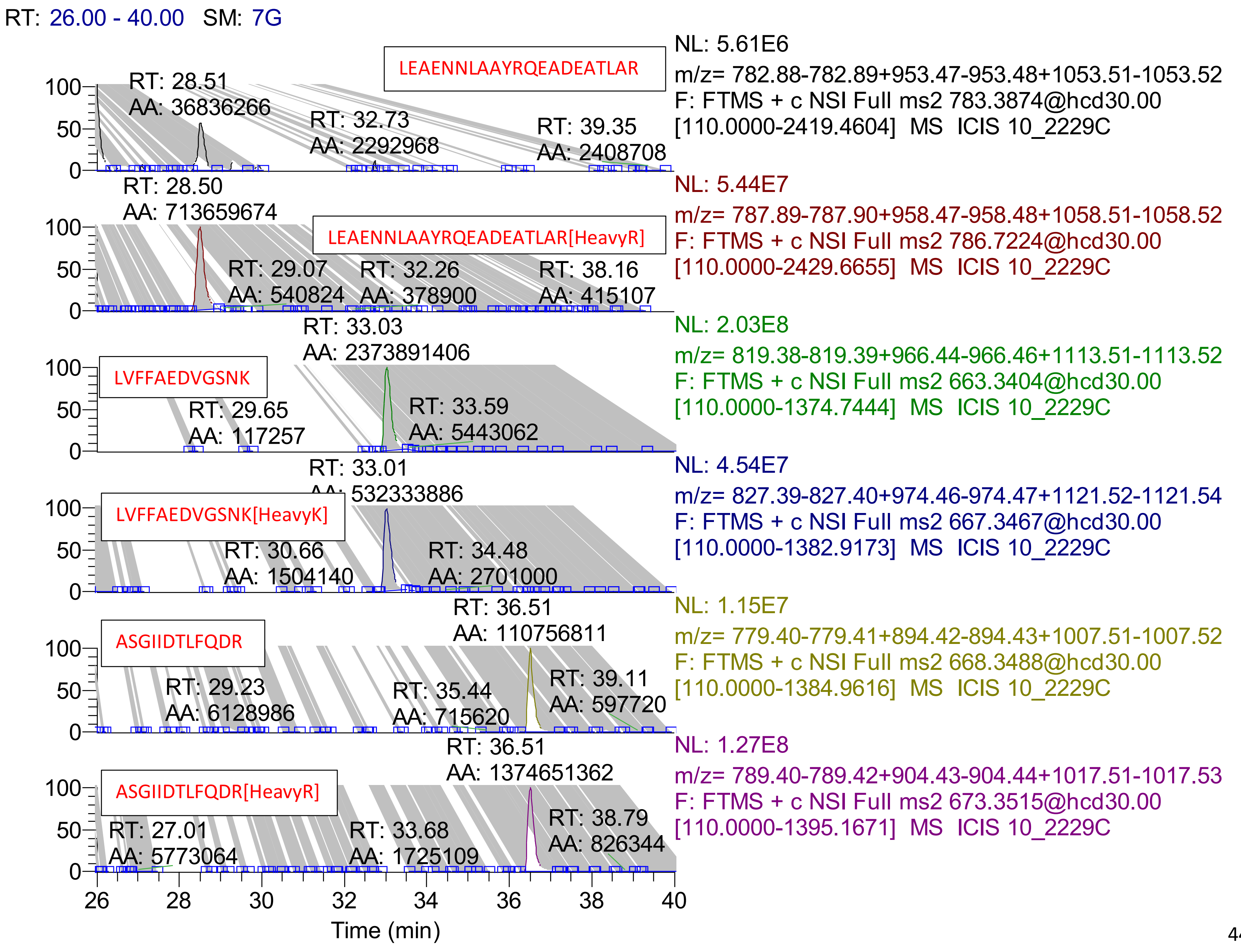
RT: 26.00 - 40.00 SM: 7G
LEAENNLAAYRQEADEATLAR
RT: 28.51
AA: 36836266
RT: 32.73
AA: 2292968
RT: 39.35
AA: 2408708
NL: 5.61E6
m/z= 782.88-782.89+953.47-953.48+1053.51-1053.52
F: FTMS + c NSI Full ms2 783.3874@hcd30.00
[110.0000-2419.4604] MS ICIS 10_2229C
RT: 28.50
AA: 713659674
LEAENNLAAYRQEADEATLAR[HeavyR]
RT: 29.07
AA: 540824
RT: 32.26
AA: 378900
RT: 38.16
AA: 415107
NL: 5.44E7
m/z= 787.89-787.90+958.47-958.48+1058.51-1058.52
F: FTMS + c NSI Full ms2 786.7224@hcd30.00
[110.0000-2429.6655] MS ICIS 10_2229C
RT: 33.03
AA: 2373891406
LVFFAEDVGSNK
RT: 29.65
AA: 117257
RT: 33.59
AA: 5443062
NL: 2.03E8
m/z= 819.38-819.39+966.44-966.46+1113.51-1113.52
F: FTMS + c NSI Full ms2 663.3404@hcd30.00
[110.0000-1374.7444] MS ICIS 10_2229C
RT: 33.01
AA: 532333886
LVFFAEDVGSNK[HeavyK]
RT: 30.66
AA: 1504140
RT: 34.48
AA: 2701000
NL: 4.54E7
m/z= 827.39-827.40+974.46-974.47+1121.52-1121.54
F: FTMS + c NSI Full ms2 667.3467@hcd30.00
[110.0000-1382.9173] MS ICIS 10_2229C
RT: 36.51
AA: 110756811
ASGIIDTLFQDR
RT: 29.23
AA: 6128986
RT: 35.44
AA: 715620
RT: 39.11
AA: 597720
NL: 1.15E7
m/z= 779.40-779.41+894.42-894.43+1007.51-1007.52
F: FTMS + c NSI Full ms2 668.3488@hcd30.00
[110.0000-1384.9616] MS ICIS 10_2229C
RT: 36.51
AA: 1374651362
ASGIIDTLFQDR[HeavyR]
RT: 27.01
AA: 5773064
RT: 33.68
AA: 1725109
RT: 38.79
AA: 826344
NL: 1.27E8
m/z= 789.40-789.42+904.43-904.44+1017.51-1017.53
F: FTMS + c NSI Full ms2 673.3515@hcd30.00
[110.0000-1395.1671] MS ICIS 10_2229C
100
50
0
26 28 30 32 34 36 38 40
Time (min)

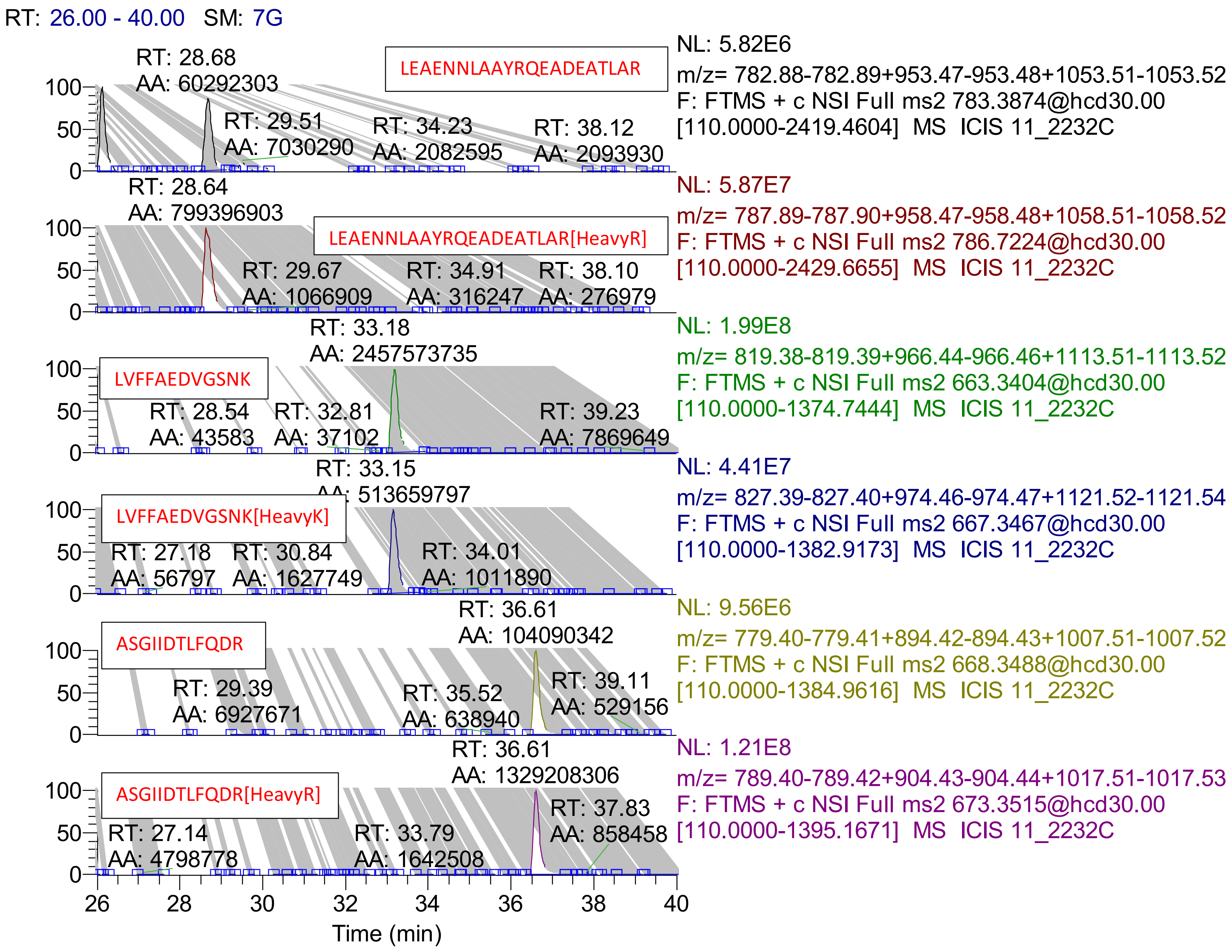

RT: 26.00 - 40.00 SM: 7G
RT: 28.68
AA: 60292303
LEAENNLAAYRQEADEATLAR
RT: 29.51
AA: 7030290
RT: 34.23
AA: 2082595
RT: 38.12
AA: 2093930
NL: 5.82E6
m/z= 782.88-782.89+953.47-953.48+1053.51-1053.52
F: FTMS + c NSI Full ms2 783.3874@hcd30.00
[110.0000-2419.4604] MS ICIS 11_2232C
RT: 28.64
AA: 799396903
LEAENNLAAYRQEADEATLAR[HeavyR]
RT: 29.67
AA: 1066909
RT: 34.91
AA: 316247
RT: 38.10
AA: 276979
NL: 5.87E7
m/z= 787.89-787.90+958.47-958.48+1058.51-1058.52
F: FTMS + c NSI Full ms2 786.7224@hcd30.00
[110.0000-2429.6655] MS ICIS 11_2232C
RT: 33.18
AA: 2457573735
LVFFAEDVGSNK
RT: 28.54
AA: 43583
RT: 32.81
AA: 37102
RT: 39.23
AA: 7869649
NL: 1.99E8
m/z= 819.38-819.39+966.44-966.46+1113.51-1113.52
F: FTMS + c NSI Full ms2 663.3404@hcd30.00
[110.0000-1374.7444] MS ICIS 11_2232C
RT: 33.15
AA: 513659797
LVFFAEDVGSNK[HeavyK]
RT: 27.18
AA: 56797
RT: 30.84
AA: 1627749
RT: 34.01
AA: 1011890
NL: 4.41E7
m/z= 827.39-827.40+974.46-974.47+1121.52-1121.54
F: FTMS + c NSI Full ms2 667.3467@hcd30.00
[110.0000-1382.9173] MS ICIS 11_2232C
RT: 36.61
AA: 104090342
ASGIIDTLFQDR
RT: 29.39
AA: 6927671
RT: 35.52
AA: 638940
RT: 39.11
AA: 529156
NL: 9.56E6
m/z= 779.40-779.41+894.42-894.43+1007.51-1007.52
F: FTMS + c NSI Full ms2 668.3488@hcd30.00
[110.0000-1384.9616] MS ICIS 11_2232C
RT: 36.61
AA: 1329208306
ASGIIDTLFQDR[HeavyR]
RT: 27.14
AA: 4798778
RT: 33.79
AA: 1642508
RT: 37.83
AA: 858458
NL: 1.21E8
m/z= 789.40-789.42+904.43-904.44+1017.51-1017.53
F: FTMS + c NSI Full ms2 673.3515@hcd30.00
[110.0000-1395.1671] MS ICIS 11_2232C
100
50
0
26
28
30
32
34
36
38
40
Time (min)

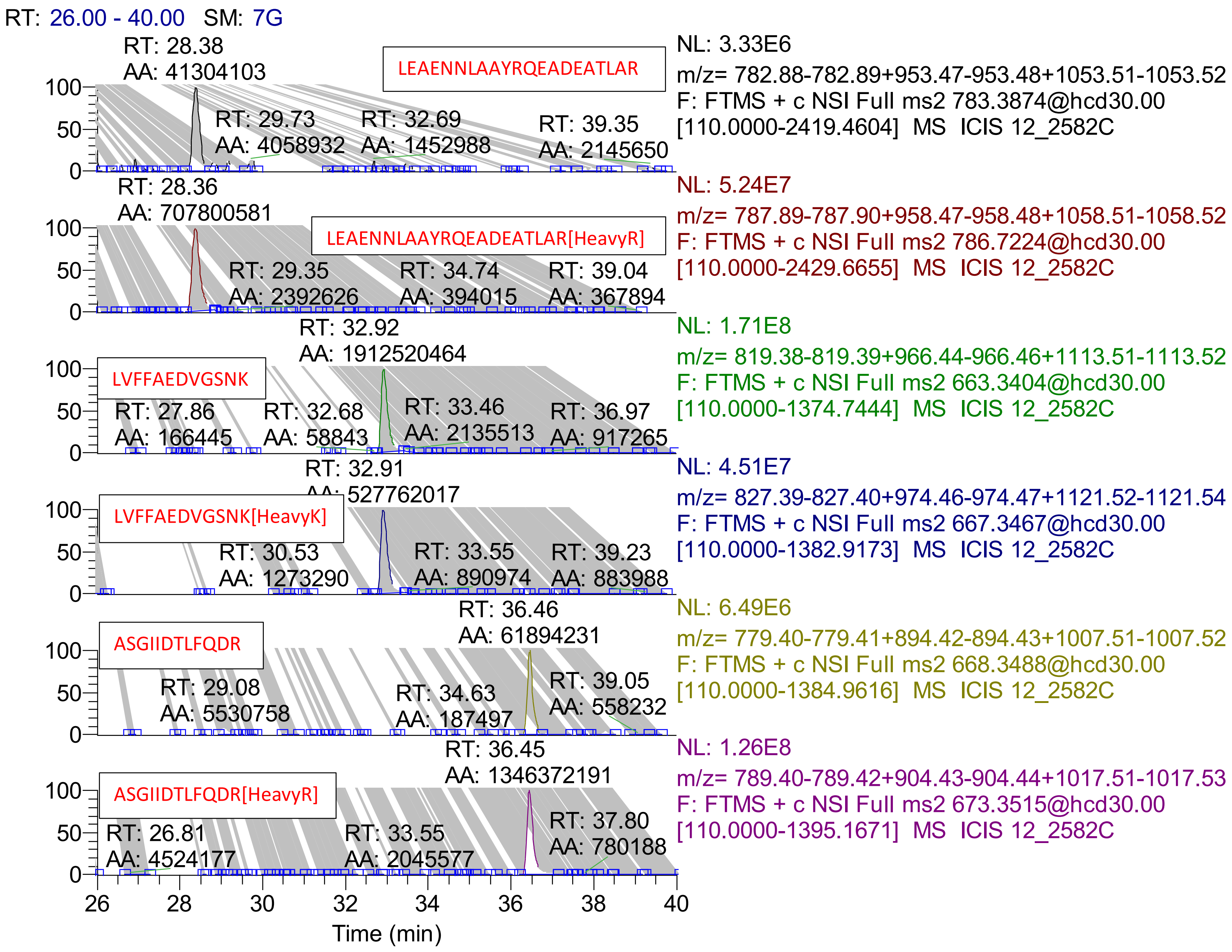
RT: 26.00 - 40.00 SM: 7G
RT: 28.38
AA: 41304103
LEAENNLAAYRQEADEATLAR
RT: 29.73
AA: 4058932
RT: 32.69
AA: 1452988
RT: 39.35
AA: 2145650
NL: 3.33E6
m/z= 782.88-782.89+953.47-953.48+1053.51-1053.52
F: FTMS + c NSI Full ms2 783.3874@hcd30.00
[110.0000-2419.4604] MS ICIS 12_2582C
RT: 28.36
AA: 707800581
LEAENNLAAYRQEADEATLAR[HeavyR]
RT: 29.35
AA: 2392626
RT: 34.74
AA: 394015
RT: 39.04
AA: 367894
NL: 5.24E7
m/z= 787.89-787.90+958.47-958.48+1058.51-1058.52
F: FTMS + c NSI Full ms2 786.7224@hcd30.00
[110.0000-2429.6655] MS ICIS 12_2582C
RT: 32.92
AA: 1912520464
LVFFAEDVGSNK
RT: 27.86
AA: 166445
RT: 32.68
AA: 58843
RT: 33.46
AA: 2135513
RT: 36.97
AA: 917265
NL: 1.71E8
m/z= 819.38-819.39+966.44-966.46+1113.51-1113.52
F: FTMS + c NSI Full ms2 663.3404@hcd30.00
[110.0000-1374.7444] MS ICIS 12_2582C
RT: 32.91
AA: 527762017
LVFFAEDVGSNK[HeavyK]
RT: 30.53
AA: 1273290
RT: 33.55
AA: 890974
RT: 39.23
AA: 883988
NL: 4.51E7
m/z= 827.39-827.40+974.46-974.47+1121.52-1121.54
F: FTMS + c NSI Full ms2 667.3467@hcd30.00
[110.0000-1382.9173] MS ICIS 12_2582C
RT: 36.46
AA: 61894231
ASGIIDTLFQDR
RT: 29.08
AA: 5530758
RT: 34.63
AA: 187497
RT: 39.05
AA: 558232
NL: 6.49E6
m/z= 779.40-779.41+894.42-894.43+1007.51-1007.52
F: FTMS + c NSI Full ms2 668.3488@hcd30.00
[110.0000-1384.9616] MS ICIS 12_2582C
RT: 36.45
AA: 1346372191
ASGIIDTLFQDR[HeavyR]
RT: 26.81
AA: 4524177
RT: 33.55
AA: 2045577
RT: 37.80
AA: 780188
NL: 1.26E8
m/z= 789.40-789.42+904.43-904.44+1017.51-1017.53
F: FTMS + c NSI Full ms2 673.3515@hcd30.00
[110.0000-1395.1671] MS ICIS 12_2582C
100
50
0
26
28
30
32
34
36
38
40
Time (min)

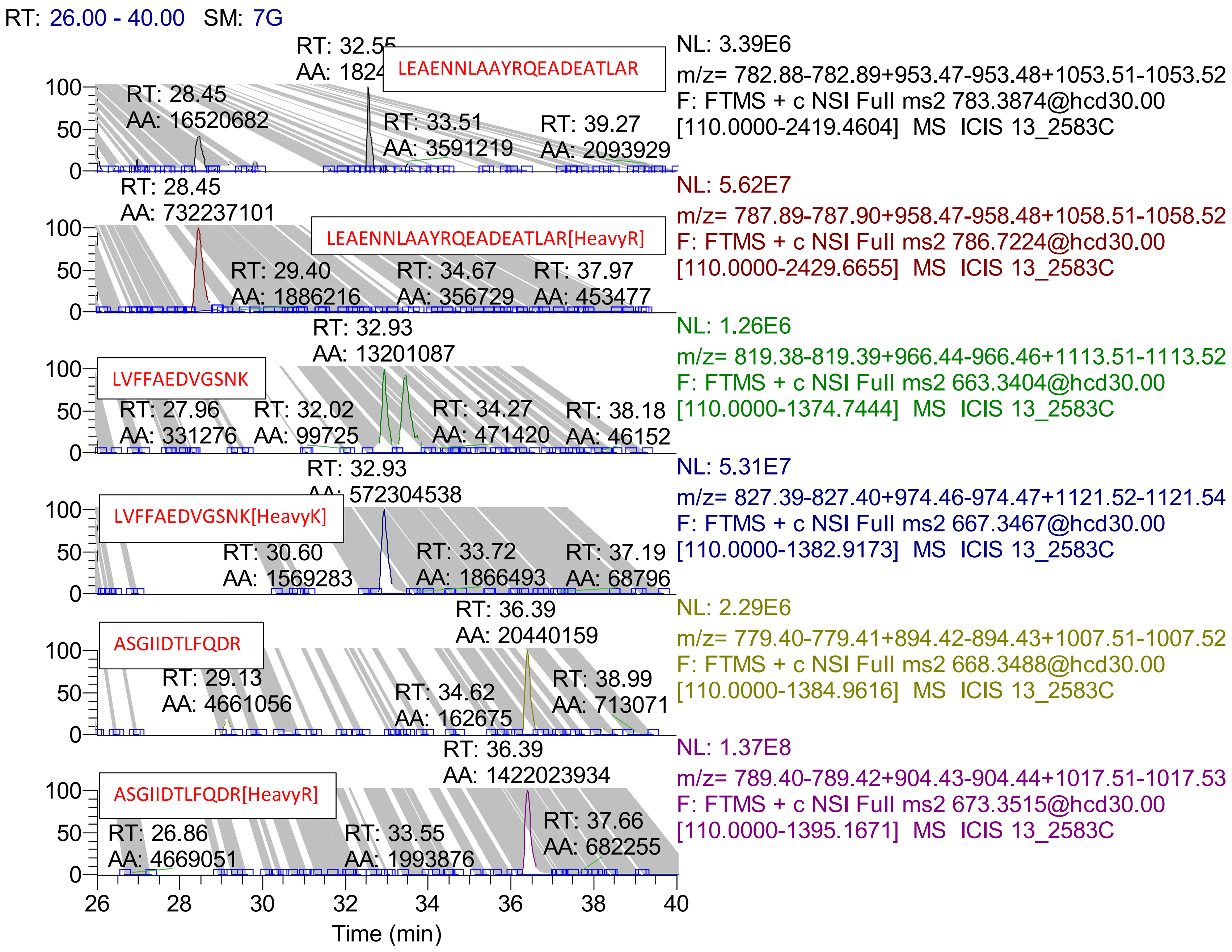
RT: 26.00 - 40.00 SM: 7G
RT: 32.55
AA: 1824
LEAENNLAAYRQEADEATLAR
RT: 28.45
AA: 16520682
RT: 33.51
AA: 3591219
RT: 39.27
AA: 2093929
NL: 3.39E6
m/z= 782.88-782.89+953.47-953.48+1053.51-1053.52
F: FTMS + c NSI Full ms2 783.3874@hcd30.00
[110.0000-2419.4604] MS ICIS 13_2583C
RT: 28.45
AA: 732237101
LEAENNLAAYRQEADEATLAR[HeavyR]
RT: 29.40
AA: 1886216
RT: 34.67
AA: 356729
RT: 37.97
AA: 453477
NL: 5.62E7
m/z= 787.89-787.90+958.47-958.48+1058.51-1058.52
F: FTMS + c NSI Full ms2 786.7224@hcd30.00
[110.0000-2429.6655] MS ICIS 13_2583C
RT: 32.93
AA: 13201087
LVFFAEDVGSNK
RT: 27.96
AA: 331276
RT: 32.02
AA: 99725
RT: 34.27
AA: 471420
RT: 38.18
AA: 46152
NL: 1.26E6
m/z= 819.38-819.39+966.44-966.46+1113.51-1113.52
F: FTMS + c NSI Full ms2 663.3404@hcd30.00
[110.0000-1374.7444] MS ICIS 13_2583C
RT: 32.93
AA: 572304538
LVFFAEDVGSNK[HeavyK]
RT: 30.60
AA: 1569283
RT: 33.72
AA: 1866493
RT: 37.19
AA: 68796
NL: 5.31E7
m/z= 827.39-827.40+974.46-974.47+1121.52-1121.54
F: FTMS + c NSI Full ms2 667.3467@hcd30.00
[110.0000-1382.9173] MS ICIS 13_2583C
RT: 36.39
AA: 20440159
ASGIIDTLFQDR
RT: 29.13
AA: 4661056
RT: 34.62
AA: 162675
RT: 38.99
AA: 713071
NL: 2.29E6
m/z= 779.40-779.41+894.42-894.43+1007.51-1007.52
F: FTMS + c NSI Full ms2 668.3488@hcd30.00
[110.0000-1384.9616] MS ICIS 13_2583C
RT: 36.39
AA: 1422023934
ASGIIDTLFQDR[HeavyR]
RT: 26.86
AA: 4669051
RT: 33.55
AA: 1993876
RT: 37.66
AA: 682255
NL: 1.37E8
m/z= 789.40-789.42+904.43-904.44+1017.51-1017.53
F: FTMS + c NSI Full ms2 673.3515@hcd30.00
[110.0000-1395.1671] MS ICIS 13_2583C
100
50
0
26
28
30
32
34
36
38
40
Time (min)

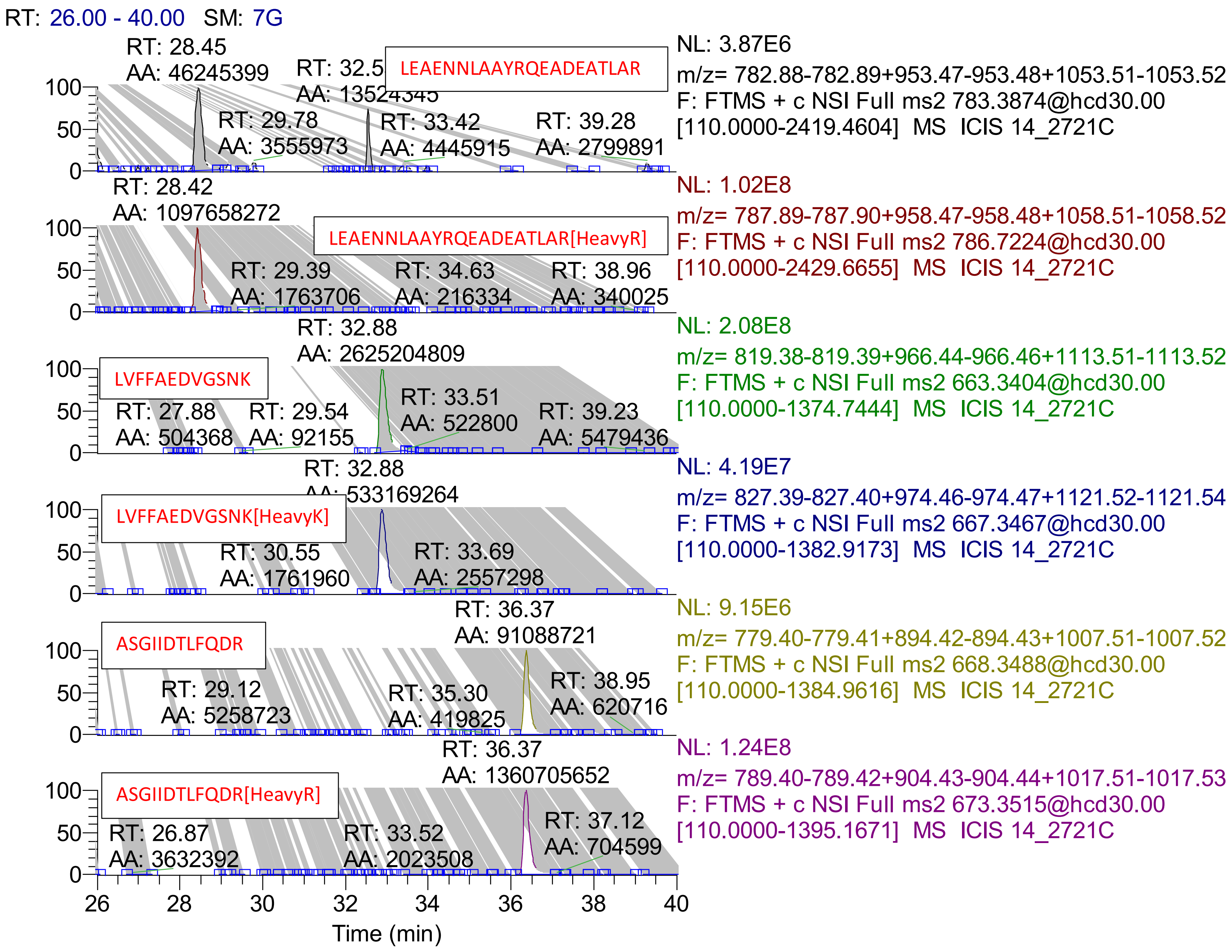
RT: 26.00 - 40.00 SM: 7G
RT: 28.45
AA: 46245399
RT: 32.5
AA: 13524345
LEAENNLAAYRQEADEATLAR
RT: 29.78
AA: 3555973
RT: 33.42
AA: 4445915
RT: 39.28
AA: 2799891
NL: 3.87E6
m/z= 782.88-782.89+953.47-953.48+1053.51-1053.52
F: FTMS + c NSI Full ms2 783.3874@hcd30.00
[110.0000-2419.4604] MS ICIS 14_2721C
RT: 28.42
AA: 1097658272
LEAENNLAAYRQEADEATLAR[HeavyR]
RT: 29.39
AA: 1763706
RT: 34.63
AA: 216334
RT: 38.96
AA: 340025
NL: 1.02E8
m/z= 787.89-787.90+958.47-958.48+1058.51-1058.52
F: FTMS + c NSI Full ms2 786.7224@hcd30.00
[110.0000-2429.6655] MS ICIS 14_2721C
RT: 32.88
AA: 2625204809
LVFFAEDVGSNK
RT: 27.88
AA: 504368
RT: 29.54
AA: 92155
RT: 33.51
AA: 522800
RT: 39.23
AA: 5479436
NL: 2.08E8
m/z= 819.38-819.39+966.44-966.46+1113.51-1113.52
F: FTMS + c NSI Full ms2 663.3404@hcd30.00
[110.0000-1374.7444] MS ICIS 14_2721C
RT: 32.88
AA: 533169264
LVFFAEDVGSNK[HeavyK]
RT: 30.55
AA: 1761960
RT: 33.69
AA: 2557298
NL: 4.19E7
m/z= 827.39-827.40+974.46-974.47+1121.52-1121.54
F: FTMS + c NSI Full ms2 667.3467@hcd30.00
[110.0000-1382.9173] MS ICIS 14_2721C
RT: 36.37
AA: 91088721
ASGIIDTLFQDR
RT: 29.12
AA: 5258723
RT: 35.30
AA: 419825
RT: 38.95
AA: 620716
NL: 9.15E6
m/z= 779.40-779.41+894.42-894.43+1007.51-1007.52
F: FTMS + c NSI Full ms2 668.3488@hcd30.00
[110.0000-1384.9616] MS ICIS 14_2721C
RT: 36.37
AA: 1360705652
ASGIIDTLFQDR[HeavyR]
RT: 26.87
AA: 3632392
RT: 33.52
AA: 2023508
RT: 37.12
AA: 704599
NL: 1.24E8
m/z= 789.40-789.42+904.43-904.44+1017.51-1017.53
F: FTMS + c NSI Full ms2 673.3515@hcd30.00
[110.0000-1395.1671] MS ICIS 14_2721C
100
50
0
26
28
30
32
34
36
38
40
Time (min)

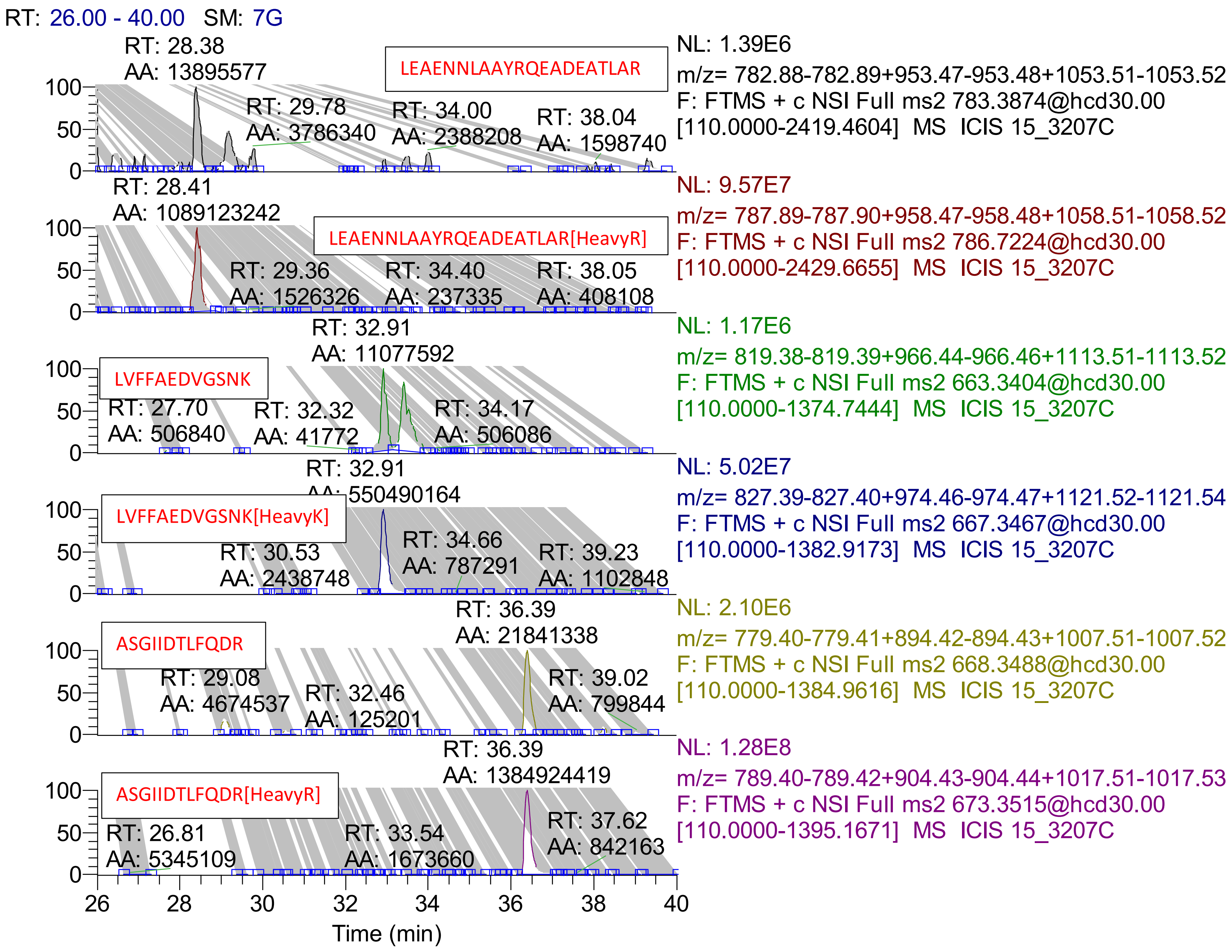
RT: 26.00 - 40.00 SM: 7G
RT: 28.38
AA: 13895577
LEAENNLAAYRQEADEATLAR
RT: 29.78
AA: 3786340
RT: 34.00
AA: 2388208
RT: 38.04
AA: 1598740
NL: 1.39E6
m/z= 782.88-782.89+953.47-953.48+1053.51-1053.52
F: FTMS + c NSI Full ms2 783.3874@hcd30.00
[110.0000-2419.4604] MS ICIS 15_3207C
RT: 28.41
AA: 1089123242
LEAENNLAAYRQEADEATLAR[HeavyR]
RT: 29.36
AA: 1526326
RT: 34.40
AA: 237335
RT: 38.05
AA: 408108
NL: 9.57E7
m/z= 787.89-787.90+958.47-958.48+1058.51-1058.52
F: FTMS + c NSI Full ms2 786.7224@hcd30.00
[110.0000-2429.6655] MS ICIS 15_3207C
RT: 32.91
AA: 11077592
LVFFAEDVGSNK
RT: 27.70
AA: 506840
RT: 32.32
AA: 41772
RT: 34.17
AA: 506086
NL: 1.17E6
m/z= 819.38-819.39+966.44-966.46+1113.51-1113.52
F: FTMS + c NSI Full ms2 663.3404@hcd30.00
[110.0000-1374.7444] MS ICIS 15_3207C
RT: 32.91
AA: 550490164
LVFFAEDVGSNK[HeavyK]
RT: 30.53
AA: 2438748
RT: 34.66
AA: 787291
RT: 39.23
AA: 1102848
NL: 5.02E7
m/z= 827.39-827.40+974.46-974.47+1121.52-1121.54
F: FTMS + c NSI Full ms2 667.3467@hcd30.00
[110.0000-1382.9173] MS ICIS 15_3207C
RT: 36.39
AA: 21841338
ASGIIDTLFQDR
RT: 29.08
AA: 4674537
RT: 32.46
AA: 125201
RT: 39.02
AA: 799844
NL: 2.10E6
m/z= 779.40-779.41+894.42-894.43+1007.51-1007.52
F: FTMS + c NSI Full ms2 668.3488@hcd30.00
[110.0000-1384.9616] MS ICIS 15_3207C
RT: 36.39
AA: 1384924419
ASGIIDTLFQDR[HeavyR]
RT: 26.81
AA: 5345109
RT: 33.54
AA: 1673660
RT: 37.62
AA: 842163
NL: 1.28E8
m/z= 789.40-789.42+904.43-904.44+1017.51-1017.53
F: FTMS + c NSI Full ms2 673.3515@hcd30.00
[110.0000-1395.1671] MS ICIS 15_3207C
100
50
0
26
28
30
32
34
36
38
40
Time (min)

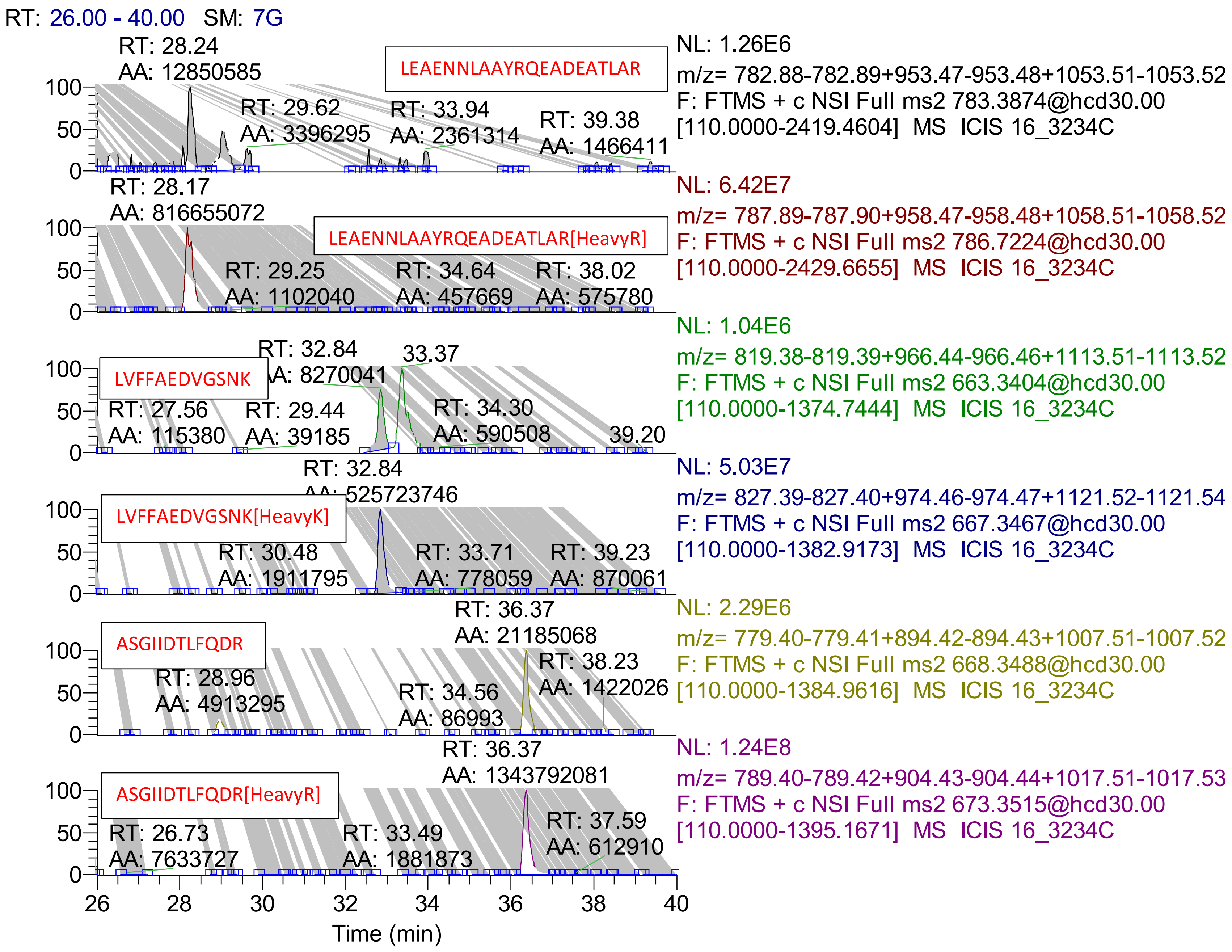

RT: 26.00 - 40.00 SM: 7G
LEAENNLAAYRQEADEATLAR
RT: 28.24
AA: 12850585
RT: 29.62
AA: 3396295
RT: 33.94
AA: 2361314
RT: 39.38
AA: 1466411
NL: 1.26E6
m/z= 782.88-782.89+953.47-953.48+1053.51-1053.52
F: FTMS + c NSI Full ms2 783.3874@hcd30.00
[110.0000-2419.4604] MS ICIS 16_3234C
LEAENNLAAYRQEADEATLAR[HeavyR]
RT: 28.17
AA: 816655072
RT: 29.25
AA: 1102040
RT: 34.64
AA: 457669
RT: 38.02
AA: 575780
NL: 6.42E7
m/z= 787.89-787.90+958.47-958.48+1058.51-1058.52
F: FTMS + c NSI Full ms2 786.7224@hcd30.00
[110.0000-2429.6655] MS ICIS 16_3234C
LVFFAEDVGSNK
RT: 32.84
AA: 8270041
33.37
RT: 27.56
AA: 115380
RT: 29.44
AA: 39185
RT: 34.30
AA: 590508
39.20
NL: 1.04E6
m/z= 819.38-819.39+966.44-966.46+1113.51-1113.52
F: FTMS + c NSI Full ms2 663.3404@hcd30.00
[110.0000-1374.7444] MS ICIS 16_3234C
LVFFAEDVGSNK[HeavyK]
RT: 32.84
AA: 525723746
RT: 30.48
AA: 1911795
RT: 33.71
AA: 778059
RT: 39.23
AA: 870061
NL: 5.03E7
m/z= 827.39-827.40+974.46-974.47+1121.52-1121.54
F: FTMS + c NSI Full ms2 667.3467@hcd30.00
[110.0000-1382.9173] MS ICIS 16_3234C
ASGIIDTLFQDR
RT: 36.37
AA: 21185068
RT: 28.96
AA: 4913295
RT: 34.56
AA: 86993
RT: 38.23
AA: 1422026
NL: 2.29E6
m/z= 779.40-779.41+894.42-894.43+1007.51-1007.52
F: FTMS + c NSI Full ms2 668.3488@hcd30.00
[110.0000-1384.9616] MS ICIS 16_3234C
ASGIIDTLFQDR[HeavyR]
RT: 36.37
AA: 1343792081
RT: 26.73
AA: 7633727
RT: 33.49
AA: 1881873
RT: 37.59
AA: 612910
NL: 1.24E8
m/z= 789.40-789.42+904.43-904.44+1017.51-1017.53
F: FTMS + c NSI Full ms2 673.3515@hcd30.00
[110.0000-1395.1671] MS ICIS 16_3234C
100
50
0
26 28 30 32 34 36 38 40
Time (min)

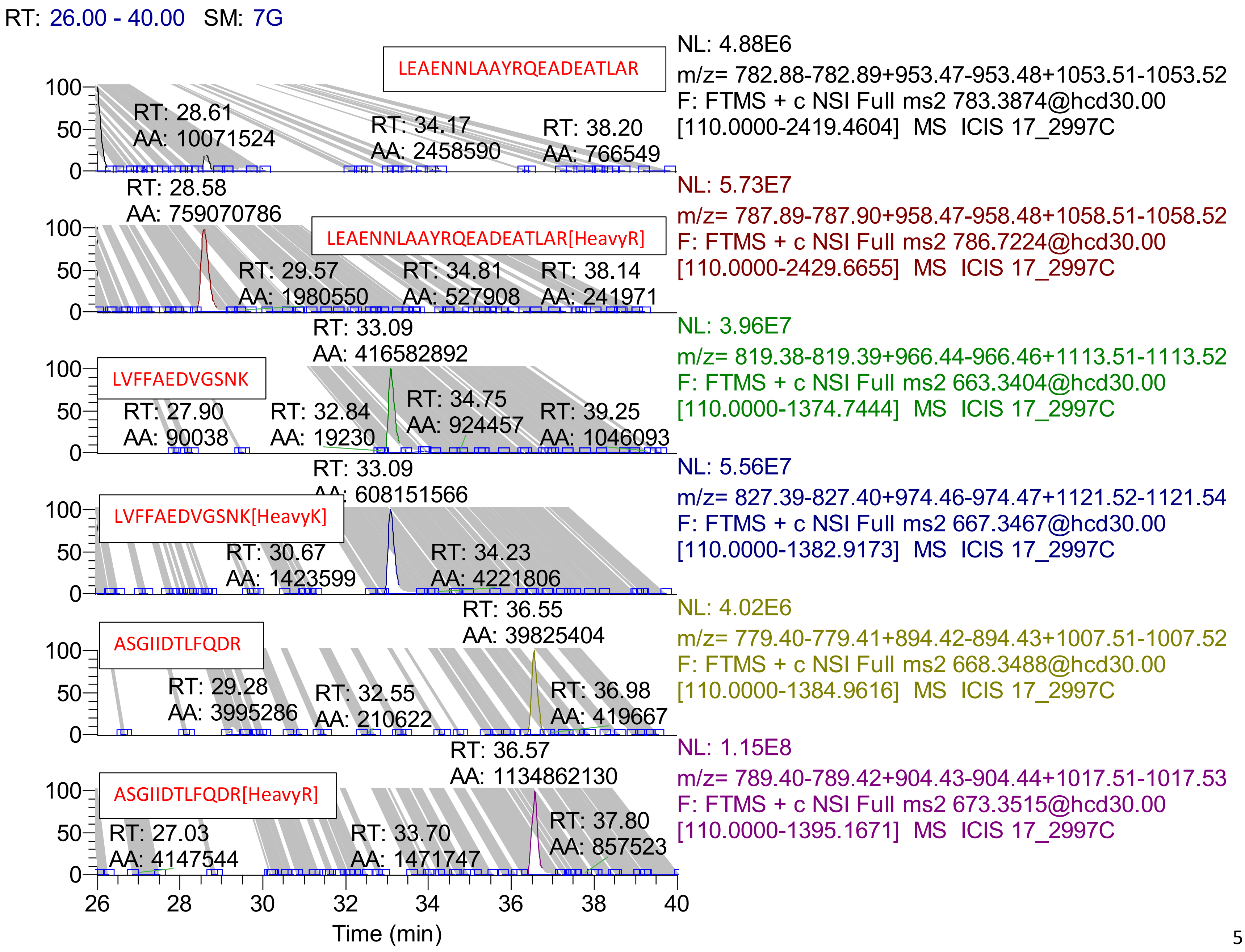
RT: 26.00 - 40.00 SM: 7G
LEAENNLAAYRQEADEATLAR
RT: 28.61
AA: 10071524
RT: 34.17
AA: 2458590
RT: 38.20
AA: 766549
NL: 4.88E6
m/z= 782.88-782.89+953.47-953.48+1053.51-1053.52
F: FTMS + c NSI Full ms2 783.3874@hcd30.00
[110.0000-2419.4604] MS ICIS 17_2997C
RT: 28.58
AA: 759070786
LEAENNLAAYRQEADEATLAR[HeavyR]
RT: 29.57
AA: 1980550
RT: 34.81
AA: 527908
RT: 38.14
AA: 241971
NL: 5.73E7
m/z= 787.89-787.90+958.47-958.48+1058.51-1058.52
F: FTMS + c NSI Full ms2 786.7224@hcd30.00
[110.0000-2429.6655] MS ICIS 17_2997C
RT: 33.09
AA: 416582892
LVFFAEDVGSNK
RT: 27.90
AA: 90038
RT: 32.84
AA: 19230
RT: 34.75
AA: 924457
RT: 39.25
AA: 1046093
NL: 3.96E7
m/z= 819.38-819.39+966.44-966.46+1113.51-1113.52
F: FTMS + c NSI Full ms2 663.3404@hcd30.00
[110.0000-1374.7444] MS ICIS 17_2997C
RT: 33.09
AA: 608151566
LVFFAEDVGSNK[HeavyK]
RT: 30.67
AA: 1423599
RT: 34.23
AA: 4221806
NL: 5.56E7
m/z= 827.39-827.40+974.46-974.47+1121.52-1121.54
F: FTMS + c NSI Full ms2 667.3467@hcd30.00
[110.0000-1382.9173] MS ICIS 17_2997C
RT: 36.55
AA: 39825404
ASGIIDTLFQDR
RT: 29.28
AA: 3995286
RT: 32.55
AA: 210622
RT: 36.98
AA: 419667
NL: 4.02E6
m/z= 779.40-779.41+894.42-894.43+1007.51-1007.52
F: FTMS + c NSI Full ms2 668.3488@hcd30.00
[110.0000-1384.9616] MS ICIS 17_2997C
RT: 36.57
AA: 1134862130
ASGIIDTLFQDR[HeavyR]
RT: 27.03
AA: 4147544
RT: 33.70
AA: 1471747
RT: 37.80
AA: 857523
NL: 1.15E8
m/z= 789.40-789.42+904.43-904.44+1017.51-1017.53
F: FTMS + c NSI Full ms2 673.3515@hcd30.00
[110.0000-1395.1671] MS ICIS 17_2997C
100
50
0
26
28
30
32
34
36
38
40
Time (min)

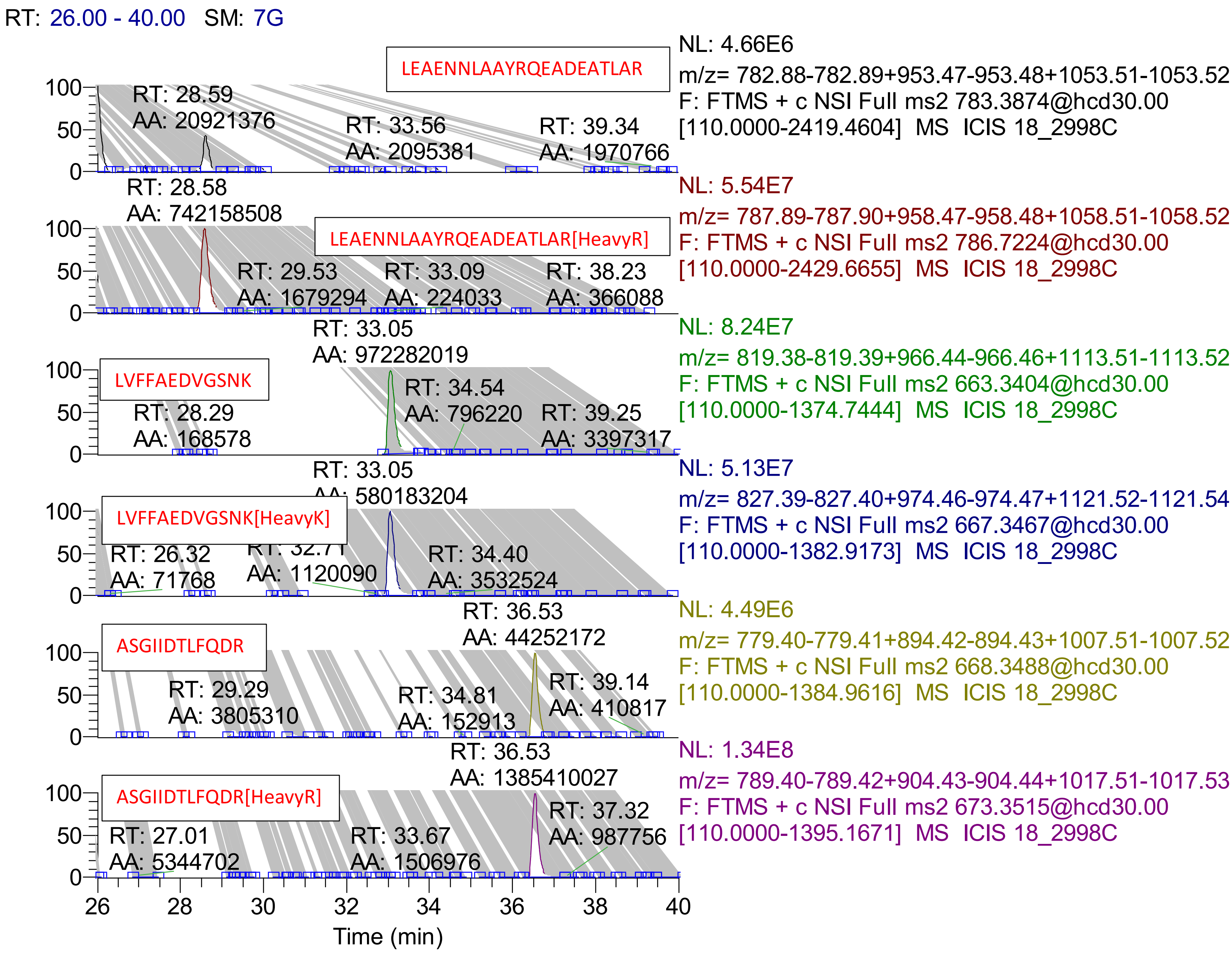
RT: 26.00 - 40.00 SM: 7G
LEAENNLAAYRQEADEATLAR
NL: 4.66E6
m/z= 782.88-782.89+953.47-953.48+1053.51-1053.52
F: FTMS + c NSI Full ms2 783.3874@hcd30.00
[110.0000-2419.4604] MS ICIS 18_2998C
RT: 28.59
AA: 20921376
RT: 33.56
AA: 2095381
RT: 39.34
AA: 1970766
RT: 28.58
AA: 742158508
LEAENNLAAYRQEADEATLAR[HeavyR]
NL: 5.54E7
m/z= 787.89-787.90+958.47-958.48+1058.51-1058.52
F: FTMS + c NSI Full ms2 786.7224@hcd30.00
[110.0000-2429.6655] MS ICIS 18_2998C
RT: 29.53
AA: 1679294
RT: 33.09
AA: 224033
RT: 38.23
AA: 366088
RT: 33.05
AA: 972282019
LVFFAEDVGSNK
NL: 8.24E7
m/z= 819.38-819.39+966.44-966.46+1113.51-1113.52
F: FTMS + c NSI Full ms2 663.3404@hcd30.00
[110.0000-1374.7444] MS ICIS 18_2998C
RT: 28.29
AA: 168578
RT: 34.54
AA: 796220
RT: 39.25
AA: 3397317
RT: 33.05
AA: 580183204
LVFFAEDVGSNK[HeavyK]
NL: 5.13E7
m/z= 827.39-827.40+974.46-974.47+1121.52-1121.54
F: FTMS + c NSI Full ms2 667.3467@hcd30.00
[110.0000-1382.9173] MS ICIS 18_2998C
RT: 26.32
AA: 71768
AA: 1120090
RT: 34.40
AA: 3532524
RT: 36.53
AA: 44252172
ASGIIDTLFQDR
NL: 4.49E6
m/z= 779.40-779.41+894.42-894.43+1007.51-1007.52
F: FTMS + c NSI Full ms2 668.3488@hcd30.00
[110.0000-1384.9616] MS ICIS 18_2998C
RT: 29.29
AA: 3805310
RT: 34.81
AA: 152913
RT: 39.14
AA: 410817
RT: 36.53
AA: 1385410027
ASGIIDTLFQDR[HeavyR]
NL: 1.34E8
m/z= 789.40-789.42+904.43-904.44+1017.51-1017.53
F: FTMS + c NSI Full ms2 673.3515@hcd30.00
[110.0000-1395.1671] MS ICIS 18_2998C
RT: 27.01
AA: 5344702
RT: 33.67
AA: 1506976
RT: 37.32
AA: 987756
100
50
0
26
28
30
32
34
36
38
40
Time (min)

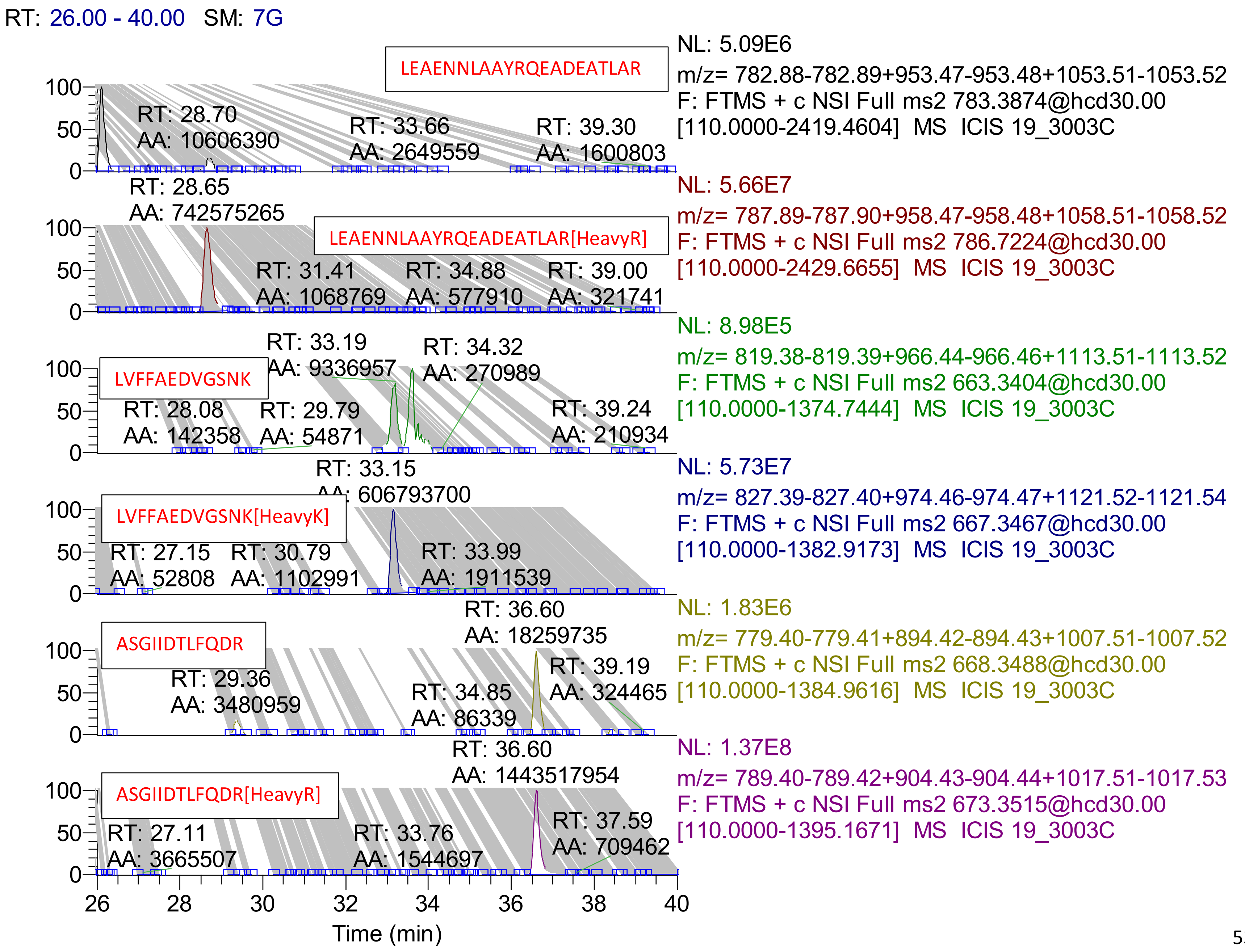

RT: 26.00 - 40.00 SM: 7G
LEAENNLAAYRQEADEATLAR
RT: 28.70
AA: 10606390
RT: 33.66
AA: 2649559
RT: 39.30
AA: 1600803
NL: 5.09E6
m/z= 782.88-782.89+953.47-953.48+1053.51-1053.52
F: FTMS + c NSI Full ms2 783.3874@hcd30.00
[110.0000-2419.4604] MS ICIS 19_3003C
RT: 28.65
AA: 742575265
LEAENNLAAYRQEADEATLAR[HeavyR]
RT: 31.41
AA: 1068769
RT: 34.88
AA: 577910
RT: 39.00
AA: 321741
NL: 5.66E7
m/z= 787.89-787.90+958.47-958.48+1058.51-1058.52
F: FTMS + c NSI Full ms2 786.7224@hcd30.00
[110.0000-2429.6655] MS ICIS 19_3003C
LVFFAEDVGSNK
RT: 33.19
AA: 9336957
RT: 34.32
AA: 270989
RT: 28.08
AA: 142358
RT: 29.79
AA: 54871
RT: 39.24
AA: 210934
NL: 8.98E5
m/z= 819.38-819.39+966.44-966.46+1113.51-1113.52
F: FTMS + c NSI Full ms2 663.3404@hcd30.00
[110.0000-1374.7444] MS ICIS 19_3003C
RT: 33.15
AA: 606793700
LVFFAEDVGSNK[HeavyK]
RT: 27.15
AA: 52808
RT: 30.79
AA: 1102991
RT: 33.99
AA: 1911539
NL: 5.73E7
m/z= 827.39-827.40+974.46-974.47+1121.52-1121.54
F: FTMS + c NSI Full ms2 667.3467@hcd30.00
[110.0000-1382.9173] MS ICIS 19_3003C
ASGIIDTLFQDR
RT: 36.60
AA: 18259735
RT: 29.36
AA: 3480959
RT: 34.85
AA: 86339
RT: 39.19
AA: 324465
NL: 1.83E6
m/z= 779.40-779.41+894.42-894.43+1007.51-1007.52
F: FTMS + c NSI Full ms2 668.3488@hcd30.00
[110.0000-1384.9616] MS ICIS 19_3003C
ASGIIDTLFQDR[HeavyR]
RT: 36.60
AA: 1443517954
RT: 27.11
AA: 3665507
RT: 33.76
AA: 1544697
RT: 37.59
AA: 709462
NL: 1.37E8
m/z= 789.40-789.42+904.43-904.44+1017.51-1017.53
F: FTMS + c NSI Full ms2 673.3515@hcd30.00
[110.0000-1395.1671] MS ICIS 19_3003C
100
50
0
26
28
30
32
34
36
38
40
Time (min)

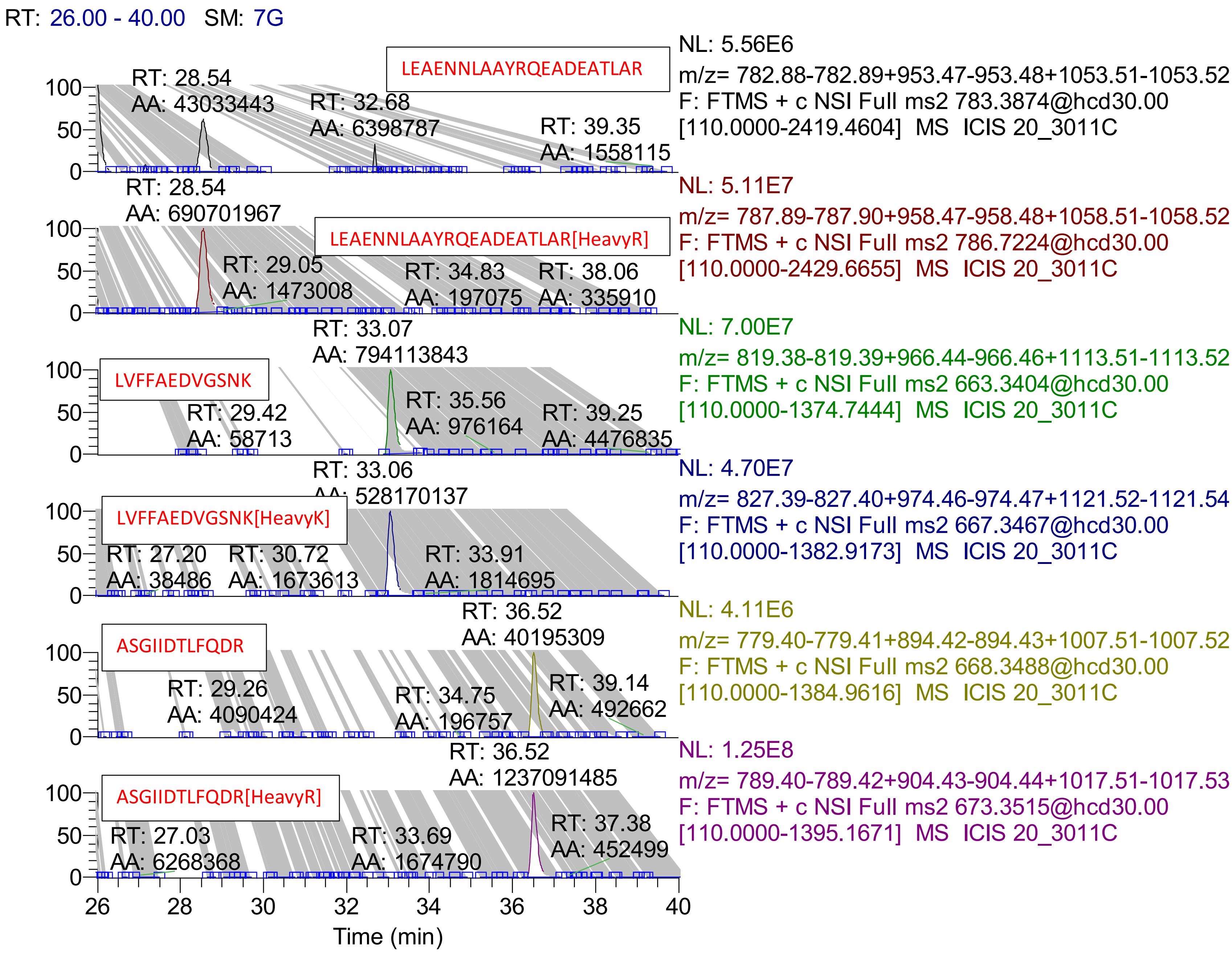

RT: 26.00 - 40.00 SM: 7G
LEAENNLAAYRQEADEATLAR
RT: 28.54
AA: 43033443
RT: 32.68
AA: 6398787
RT: 39.35
AA: 1558115
NL: 5.56E6
m/z= 782.88-782.89+953.47-953.48+1053.51-1053.52
F: FTMS + c NSI Full ms2 783.3874@hcd30.00
[110.0000-2419.4604] MS ICIS 20_3011C
RT: 28.54
AA: 690701967
LEAENNLAAYRQEADEATLAR[HeavyR]
RT: 29.05
AA: 1473008
RT: 34.83
AA: 197075
RT: 38.06
AA: 335910
NL: 5.11E7
m/z= 787.89-787.90+958.47-958.48+1058.51-1058.52
F: FTMS + c NSI Full ms2 786.7224@hcd30.00
[110.0000-2429.6655] MS ICIS 20_3011C
RT: 33.07
AA: 794113843
LVFFAEDVGSNK
RT: 29.42
AA: 58713
RT: 35.56
AA: 976164
RT: 39.25
AA: 4476835
NL: 7.00E7
m/z= 819.38-819.39+966.44-966.46+1113.51-1113.52
F: FTMS + c NSI Full ms2 663.3404@hcd30.00
[110.0000-1374.7444] MS ICIS 20_3011C
RT: 33.06
AA: 528170137
LVFFAEDVGSNK[HeavyK]
RT: 27.20
AA: 38486
RT: 30.72
AA: 1673613
RT: 33.91
AA: 1814695
NL: 4.70E7
m/z= 827.39-827.40+974.46-974.47+1121.52-1121.54
F: FTMS + c NSI Full ms2 667.3467@hcd30.00
[110.0000-1382.9173] MS ICIS 20_3011C
RT: 36.52
AA: 40195309
ASGIIDTLFQDR
RT: 29.26
AA: 4090424
RT: 34.75
AA: 196757
RT: 39.14
AA: 492662
NL: 4.11E6
m/z= 779.40-779.41+894.42-894.43+1007.51-1007.52
F: FTMS + c NSI Full ms2 668.3488@hcd30.00
[110.0000-1384.9616] MS ICIS 20_3011C
RT: 36.52
AA: 1237091485
ASGIIDTLFQDR[HeavyR]
RT: 27.03
AA: 6268368
RT: 33.69
AA: 1674790
RT: 37.38
AA: 452499
NL: 1.25E8
m/z= 789.40-789.42+904.43-904.44+1017.51-1017.53
F: FTMS + c NSI Full ms2 673.3515@hcd30.00
[110.0000-1395.1671] MS ICIS 20_3011C
100
50
0
26
28
30
32
34
36
38
40
Time (min)

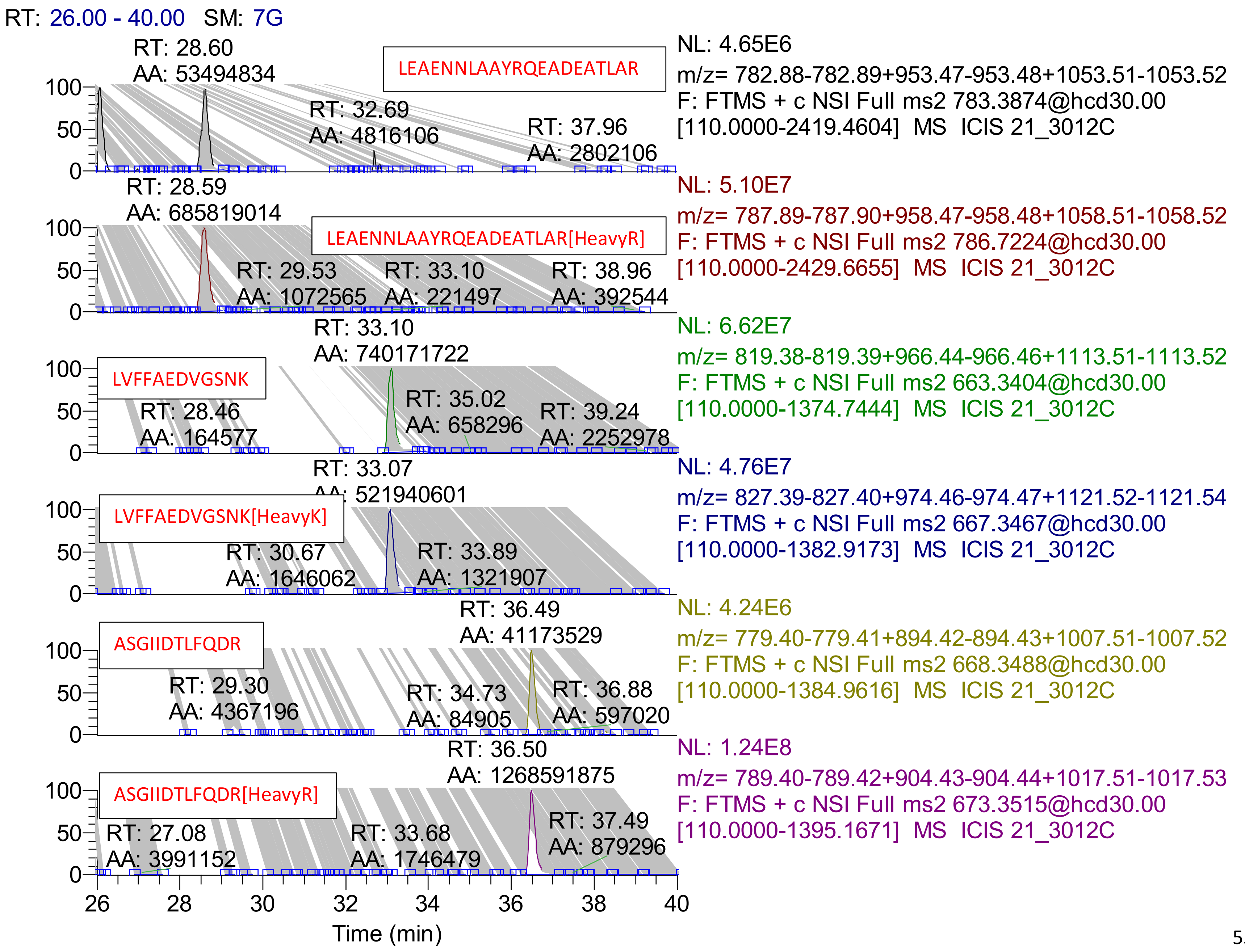
RT: 26.00 - 40.00 SM: 7G
RT: 28.60
AA: 53494834
LEAENNLAAYRQEADEATLAR
RT: 32.69
AA: 4816106
RT: 37.96
AA: 2802106
NL: 4.65E6
m/z= 782.88-782.89+953.47-953.48+1053.51-1053.52
F: FTMS + c NSI Full ms2 783.3874@hcd30.00
[110.0000-2419.4604] MS ICIS 21_3012C
RT: 28.59
AA: 685819014
LEAENNLAAYRQEADEATLAR[HeavyR]
RT: 29.53
AA: 1072565
RT: 33.10
AA: 221497
RT: 38.96
AA: 392544
NL: 5.10E7
m/z= 787.89-787.90+958.47-958.48+1058.51-1058.52
F: FTMS + c NSI Full ms2 786.7224@hcd30.00
[110.0000-2429.6655] MS ICIS 21_3012C
RT: 33.10
AA: 740171722
LVFFAEDVGSNK
RT: 28.46
AA: 164577
RT: 35.02
AA: 658296
RT: 39.24
AA: 2252978
NL: 6.62E7
m/z= 819.38-819.39+966.44-966.46+1113.51-1113.52
F: FTMS + c NSI Full ms2 663.3404@hcd30.00
[110.0000-1374.7444] MS ICIS 21_3012C
RT: 33.07
AA: 521940601
LVFFAEDVGSNK[HeavyK]
RT: 30.67
AA: 1646062
RT: 33.89
AA: 1321907
NL: 4.76E7
m/z= 827.39-827.40+974.46-974.47+1121.52-1121.54
F: FTMS + c NSI Full ms2 667.3467@hcd30.00
[110.0000-1382.9173] MS ICIS 21_3012C
RT: 36.49
AA: 41173529
ASGIIDTLFQDR
RT: 29.30
AA: 4367196
RT: 34.73
AA: 84905
RT: 36.88
AA: 597020
NL: 4.24E6
m/z= 779.40-779.41+894.42-894.43+1007.51-1007.52
F: FTMS + c NSI Full ms2 668.3488@hcd30.00
[110.0000-1384.9616] MS ICIS 21_3012C
RT: 36.50
AA: 1268591875
ASGIIDTLFQDR[HeavyR]
RT: 27.08
AA: 3991152
RT: 33.68
AA: 1746479
RT: 37.49
AA: 879296
NL: 1.24E8
m/z= 789.40-789.42+904.43-904.44+1017.51-1017.53
F: FTMS + c NSI Full ms2 673.3515@hcd30.00
[110.0000-1395.1671] MS ICIS 21_3012C
100
50
0
26
28
30
32
34
36
38
40
Time (min)

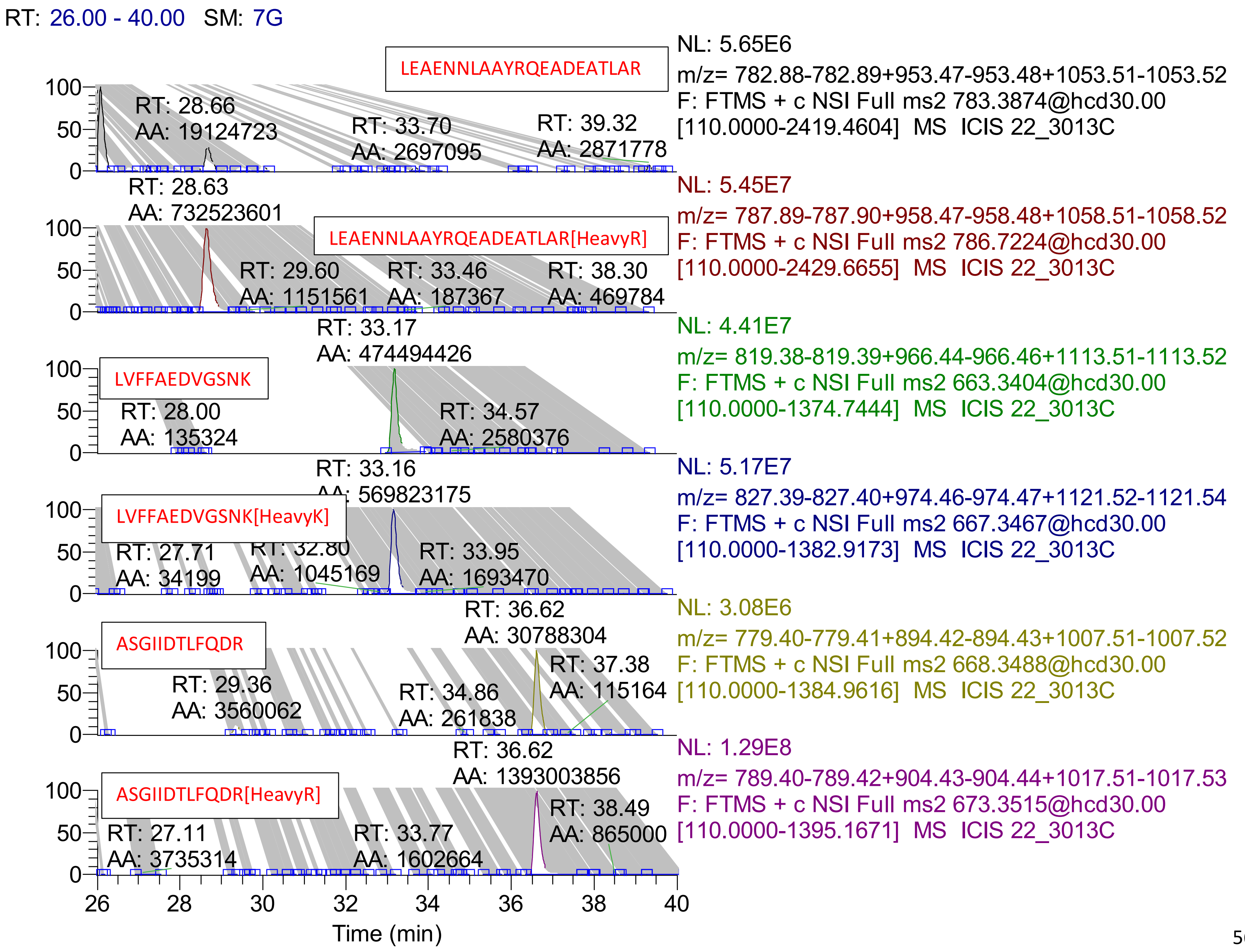
RT: 26.00 - 40.00 SM: 7G
LEAENNLAAYRQEADEATLAR
RT: 28.66
AA: 19124723
RT: 33.70
AA: 2697095
RT: 39.32
AA: 2871778
NL: 5.65E6
m/z= 782.88-782.89+953.47-953.48+1053.51-1053.52
F: FTMS + c NSI Full ms2 783.3874@hcd30.00
[110.0000-2419.4604] MS ICIS 22_3013C
RT: 28.63
AA: 732523601
LEAENNLAAYRQEADEATLAR[HeavyR]
RT: 29.60
AA: 1151561
RT: 33.46
AA: 187367
RT: 38.30
AA: 469784
NL: 5.45E7
m/z= 787.89-787.90+958.47-958.48+1058.51-1058.52
F: FTMS + c NSI Full ms2 786.7224@hcd30.00
[110.0000-2429.6655] MS ICIS 22_3013C
RT: 33.17
AA: 474494426
LVFFAEDVGSNK
RT: 28.00
AA: 135324
RT: 34.57
AA: 2580376
NL: 4.41E7
m/z= 819.38-819.39+966.44-966.46+1113.51-1113.52
F: FTMS + c NSI Full ms2 663.3404@hcd30.00
[110.0000-1374.7444] MS ICIS 22_3013C
RT: 33.16
AA: 569823175
LVFFAEDVGSNK[HeavyK]
RT: 27.71
AA: 34199
RT: 32.80
AA: 1045169
RT: 33.95
AA: 1693470
NL: 5.17E7
m/z= 827.39-827.40+974.46-974.47+1121.52-1121.54
F: FTMS + c NSI Full ms2 667.3467@hcd30.00
[110.0000-1382.9173] MS ICIS 22_3013C
RT: 36.62
AA: 30788304
ASGIIDTLFQDR
RT: 29.36
AA: 3560062
RT: 34.86
AA: 261838
RT: 37.38
AA: 115164
NL: 3.08E6
m/z= 779.40-779.41+894.42-894.43+1007.51-1007.52
F: FTMS + c NSI Full ms2 668.3488@hcd30.00
[110.0000-1384.9616] MS ICIS 22_3013C
RT: 36.62
AA: 1393003856
ASGIIDTLFQDR[HeavyR]
RT: 27.11
AA: 3735314
RT: 33.77
AA: 1602664
RT: 38.49
AA: 865000
NL: 1.29E8
m/z= 789.40-789.42+904.43-904.44+1017.51-1017.53
F: FTMS + c NSI Full ms2 673.3515@hcd30.00
[110.0000-1395.1671] MS ICIS 22_3013C
100
50
0
26
28
30
32
34
36
38
40
Time (min)

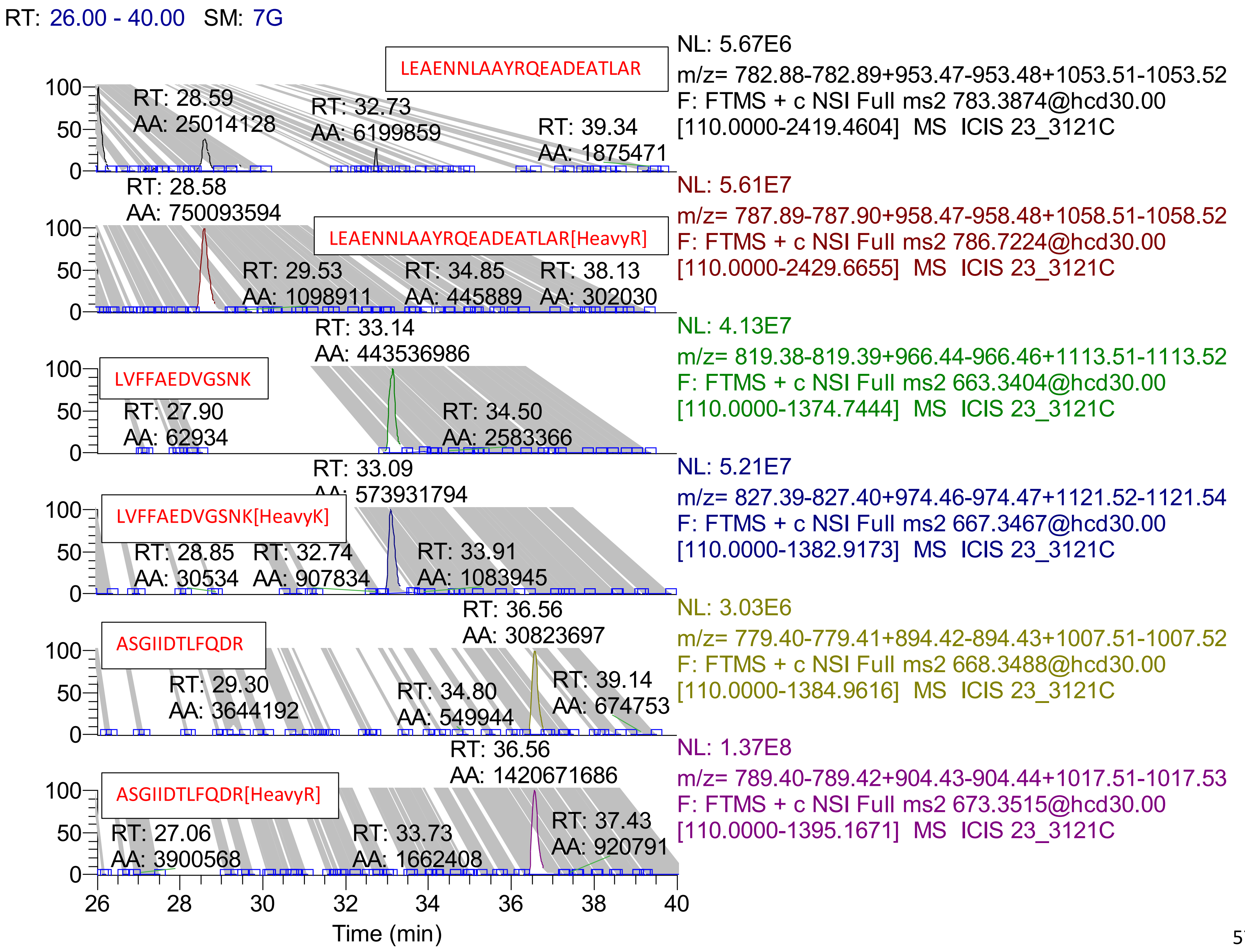
RT: 26.00 - 40.00 SM: 7G
LEAENNLAAYRQEADEATLAR
NL: 5.67E6
m/z= 782.88-782.89+953.47-953.48+1053.51-1053.52
F: FTMS + c NSI Full ms2 783.3874@hcd30.00
[110.0000-2419.4604] MS ICIS 23_3121C
RT: 28.59
AA: 25014128
RT: 32.73
AA: 6199859
RT: 39.34
AA: 1875471
LEAENNLAAYRQEADEATLAR[HeavyR]
NL: 5.61E7
m/z= 787.89-787.90+958.47-958.48+1058.51-1058.52
F: FTMS + c NSI Full ms2 786.7224@hcd30.00
[110.0000-2429.6655] MS ICIS 23_3121C
RT: 28.58
AA: 750093594
RT: 29.53
AA: 1098911
RT: 34.85
AA: 445889
RT: 38.13
AA: 302030
LVFFAEDVGSNK
NL: 4.13E7
m/z= 819.38-819.39+966.44-966.46+1113.51-1113.52
F: FTMS + c NSI Full ms2 663.3404@hcd30.00
[110.0000-1374.7444] MS ICIS 23_3121C
RT: 33.14
AA: 443536986
RT: 27.90
AA: 62934
RT: 34.50
AA: 2583366
LVFFAEDVGSNK[HeavyK]
NL: 5.21E7
m/z= 827.39-827.40+974.46-974.47+1121.52-1121.54
F: FTMS + c NSI Full ms2 667.3467@hcd30.00
[110.0000-1382.9173] MS ICIS 23_3121C
RT: 33.09
AA: 573931794
RT: 28.85
AA: 30534
RT: 32.74
AA: 907834
RT: 33.91
AA: 1083945
ASGIIDTLFQDR
NL: 3.03E6
m/z= 779.40-779.41+894.42-894.43+1007.51-1007.52
F: FTMS + c NSI Full ms2 668.3488@hcd30.00
[110.0000-1384.9616] MS ICIS 23_3121C
RT: 36.56
AA: 30823697
RT: 29.30
AA: 3644192
RT: 34.80
AA: 549944
RT: 39.14
AA: 674753
ASGIIDTLFQDR[HeavyR]
NL: 1.37E8
m/z= 789.40-789.42+904.43-904.44+1017.51-1017.53
F: FTMS + c NSI Full ms2 673.3515@hcd30.00
[110.0000-1395.1671] MS ICIS 23_3121C
RT: 36.56
AA: 1420671686
RT: 27.06
AA: 3900568
RT: 33.73
AA: 1662408
RT: 37.43
AA: 920791
100
50
0
26
28
30
32
34
36
38
40
Time (min)

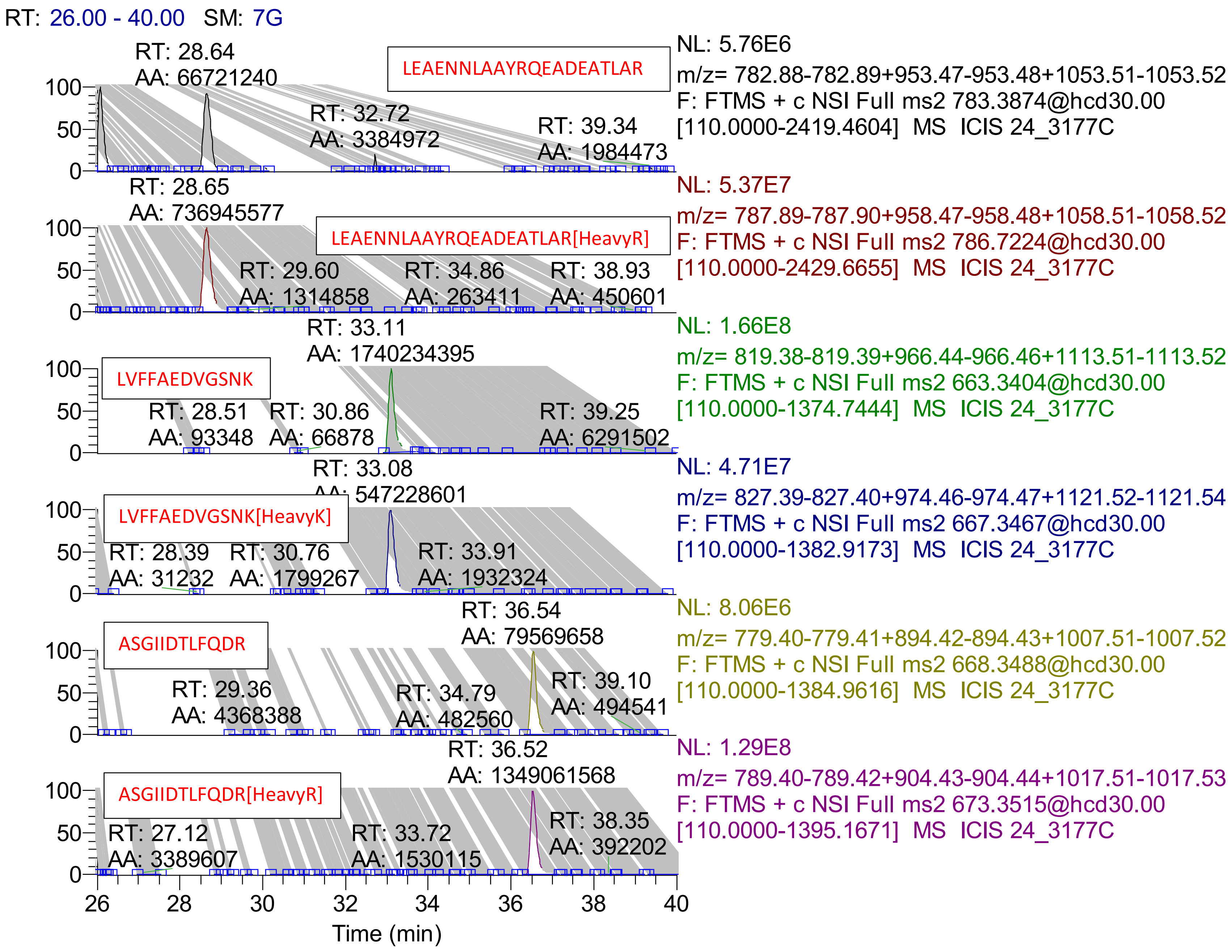
RT: 26.00 - 40.00 SM: 7G
RT: 28.64
AA: 66721240
LEAEENNLAAYRQEADEATLAR
RT: 32.72
AA: 3384972
RT: 39.34
AA: 1984473
NL: 5.76E6
m/z= 782.88-782.89+953.47-953.48+1053.51-1053.52
F: FTMS + c NSI Full ms2 783.3874@hcd30.00
[110.0000-2419.4604] MS ICIS 24_3177C
RT: 28.65
AA: 736945577
LEAEENNLAAYRQEADEATLAR[HeavyR]
RT: 29.60
AA: 1314858
RT: 34.86
AA: 263411
RT: 38.93
AA: 450601
NL: 5.37E7
m/z= 787.89-787.90+958.47-958.48+1058.51-1058.52
F: FTMS + c NSI Full ms2 786.7224@hcd30.00
[110.0000-2429.6655] MS ICIS 24_3177C
RT: 33.11
AA: 1740234395
LVFFAEDVGSNK
RT: 28.51
AA: 93348
RT: 30.86
AA: 66878
RT: 39.25
AA: 6291502
NL: 1.66E8
m/z= 819.38-819.39+966.44-966.46+1113.51-1113.52
F: FTMS + c NSI Full ms2 663.3404@hcd30.00
[110.0000-1374.7444] MS ICIS 24_3177C
RT: 33.08
AA: 547228601
LVFFAEDVGSNK[HeavyK]
RT: 28.39
AA: 31232
RT: 30.76
AA: 1799267
RT: 33.91
AA: 1932324
NL: 4.71E7
m/z= 827.39-827.40+974.46-974.47+1121.52-1121.54
F: FTMS + c NSI Full ms2 667.3467@hcd30.00
[110.0000-1382.9173] MS ICIS 24_3177C
RT: 36.54
AA: 79569658
ASGIIDTLFQDR
RT: 29.36
AA: 4368388
RT: 34.79
AA: 482560
RT: 39.10
AA: 494541
NL: 8.06E6
m/z= 779.40-779.41+894.42-894.43+1007.51-1007.52
F: FTMS + c NSI Full ms2 668.3488@hcd30.00
[110.0000-1384.9616] MS ICIS 24_3177C
RT: 36.52
AA: 1349061568
ASGIIDTLFQDR[HeavyR]
RT: 27.12
AA: 3389607
RT: 33.72
AA: 1530115
RT: 38.35
AA: 392202
NL: 1.29E8
m/z= 789.40-789.42+904.43-904.44+1017.51-1017.53
F: FTMS + c NSI Full ms2 673.3515@hcd30.00
[110.0000-1395.1671] MS ICIS 24_3177C
100
50
0
26
28
30
32
34
36
38
40
Time (min)

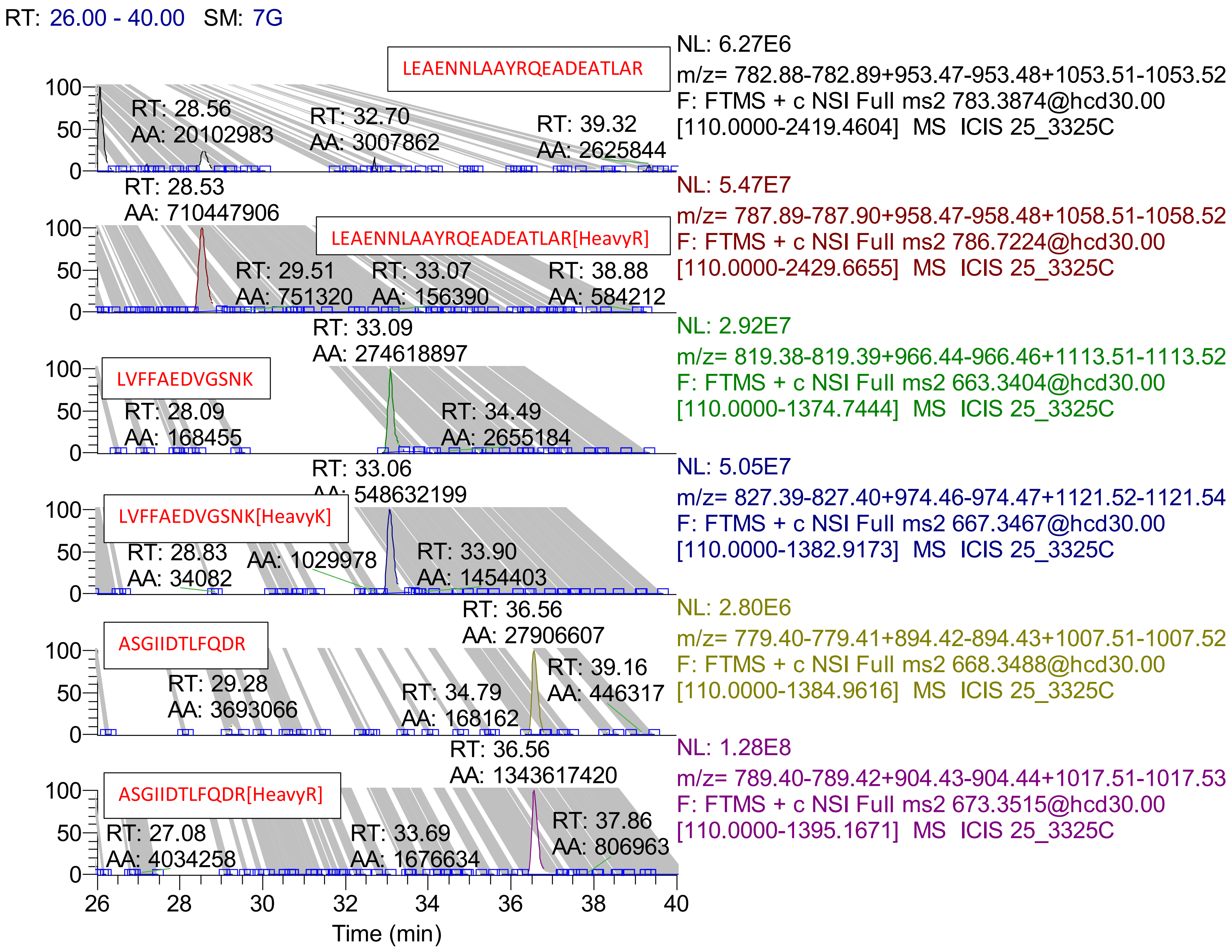
RT: 26.00 - 40.00 SM: 7G
LEAENNLAAYRQEADEATLAR
RT: 28.56
AA: 20102983
RT: 32.70
AA: 3007862
RT: 39.32
AA: 2625844
NL: 6.27E6
m/z= 782.88-782.89+953.47-953.48+1053.51-1053.52
F: FTMS + c NSI Full ms2 783.3874@hcd30.00
[110.0000-2419.4604] MS ICIS 25_3325C
RT: 28.53
AA: 710447906
LEAENNLAAYRQEADEATLAR[HeavyR]
RT: 29.51
AA: 751320
RT: 33.07
AA: 156390
RT: 38.88
AA: 584212
NL: 5.47E7
m/z= 787.89-787.90+958.47-958.48+1058.51-1058.52
F: FTMS + c NSI Full ms2 786.7224@hcd30.00
[110.0000-2429.6655] MS ICIS 25_3325C
RT: 33.09
AA: 274618897
LVFFAEDVGSNK
RT: 28.09
AA: 168455
RT: 34.49
AA: 2655184
NL: 2.92E7
m/z= 819.38-819.39+966.44-966.46+1113.51-1113.52
F: FTMS + c NSI Full ms2 663.3404@hcd30.00
[110.0000-1374.7444] MS ICIS 25_3325C
RT: 33.06
AA: 548632199
LVFFAEDVGSNK[HeavyK]
RT: 28.83
AA: 34082
AA: 1029978
RT: 33.90
AA: 1454403
NL: 5.05E7
m/z= 827.39-827.40+974.46-974.47+1121.52-1121.54
F: FTMS + c NSI Full ms2 667.3467@hcd30.00
[110.0000-1382.9173] MS ICIS 25_3325C
RT: 36.56
AA: 27906607
ASGIIDTLFQDR
RT: 29.28
AA: 3693066
RT: 34.79
AA: 168162
RT: 39.16
AA: 446317
NL: 2.80E6
m/z= 779.40-779.41+894.42-894.43+1007.51-1007.52
F: FTMS + c NSI Full ms2 668.3488@hcd30.00
[110.0000-1384.9616] MS ICIS 25_3325C
RT: 36.56
AA: 1343617420
ASGIIDTLFQDR[HeavyR]
RT: 27.08
AA: 4034258
RT: 33.69
AA: 1676634
RT: 37.86
AA: 806963
NL: 1.28E8
m/z= 789.40-789.42+904.43-904.44+1017.51-1017.53
F: FTMS + c NSI Full ms2 673.3515@hcd30.00
[110.0000-1395.1671] MS ICIS 25_3325C
100
50
0
26
28
30
32
34
36
38
40
Time (min)

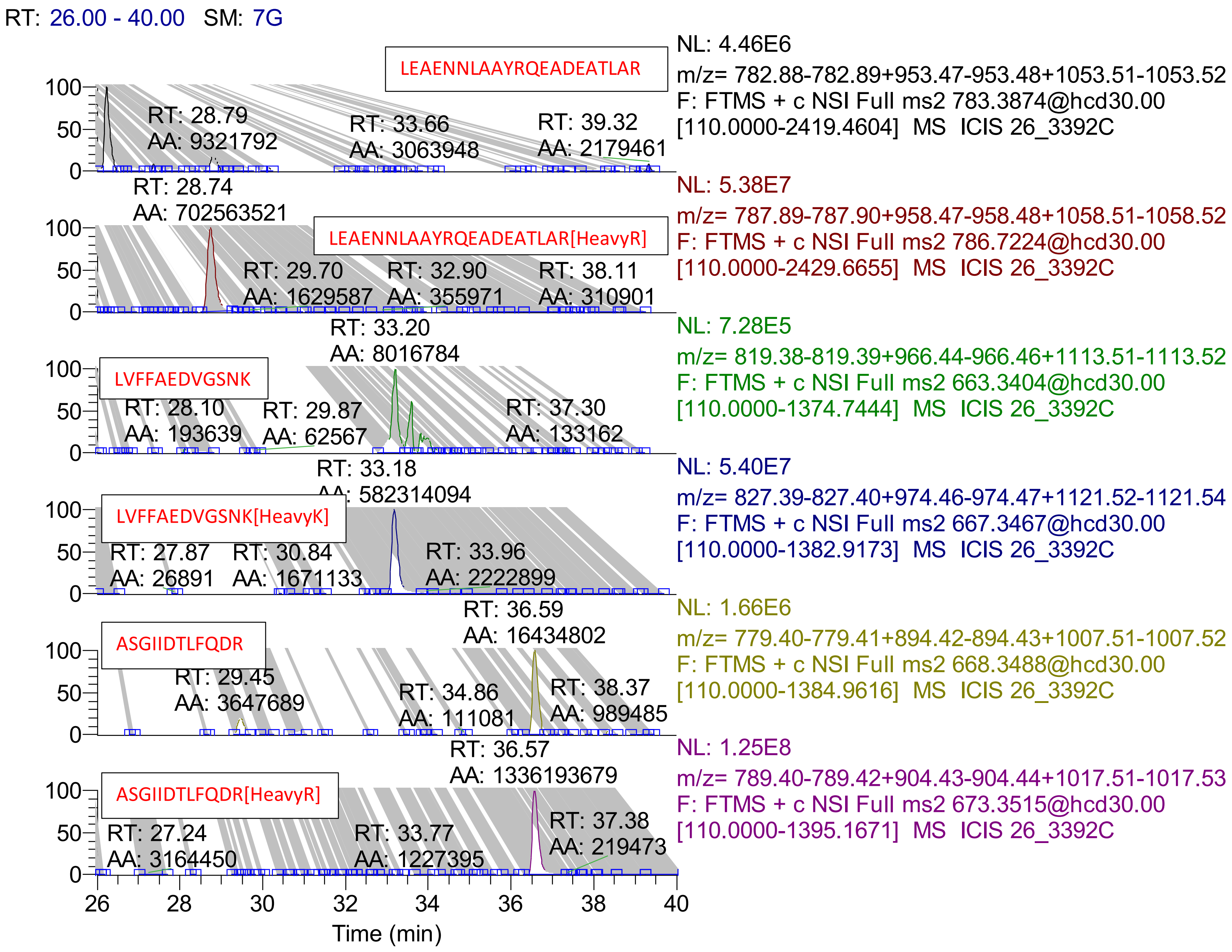
RT: 26.00 - 40.00 SM: 7G
LEAENNLAAYRQEADEATLAR
RT: 28.79 AA: 9321792
RT: 33.66 AA: 3063948
RT: 39.32 AA: 2179461
NL: 4.46E6
m/z= 782.88-782.89+953.47-953.48+1053.51-1053.52
F: FTMS + c NSI Full ms2 783.3874@hcd30.00
[110.0000-2419.4604] MS ICIS 26_3392C
RT: 28.74 AA: 702563521
LEAENNLAAYRQEADEATLAR[HeavyR]
RT: 29.70 AA: 1629587
RT: 32.90 AA: 355971
RT: 38.11 AA: 310901
NL: 5.38E7
m/z= 787.89-787.90+958.47-958.48+1058.51-1058.52
F: FTMS + c NSI Full ms2 786.7224@hcd30.00
[110.0000-2429.6655] MS ICIS 26_3392C
RT: 33.20 AA: 8016784
LVFFAEDVGSNK
RT: 28.10 AA: 193639
RT: 29.87 AA: 62567
RT: 37.30 AA: 133162
NL: 7.28E5
m/z= 819.38-819.39+966.44-966.46+1113.51-1113.52
F: FTMS + c NSI Full ms2 663.3404@hcd30.00
[110.0000-1374.7444] MS ICIS 26_3392C
RT: 33.18 AA: 582314094
LVFFAEDVGSNK[HeavyK]
RT: 27.87 AA: 26891
RT: 30.84 AA: 1671133
RT: 33.96 AA: 2222899
NL: 5.40E7
m/z= 827.39-827.40+974.46-974.47+1121.52-1121.54
F: FTMS + c NSI Full ms2 667.3467@hcd30.00
[110.0000-1382.9173] MS ICIS 26_3392C
RT: 36.59 AA: 16434802
ASGIIDTLFQDR
RT: 29.45 AA: 3647689
RT: 34.86 AA: 111081
RT: 38.37 AA: 989485
NL: 1.66E6
m/z= 779.40-779.41+894.42-894.43+1007.51-1007.52
F: FTMS + c NSI Full ms2 668.3488@hcd30.00
[110.0000-1384.9616] MS ICIS 26_3392C
RT: 36.57 AA: 1336193679
ASGIIDTLFQDR[HeavyR]
RT: 27.24 AA: 3164450
RT: 33.77 AA: 1227395
RT: 37.38 AA: 219473
NL: 1.25E8
m/z= 789.40-789.42+904.43-904.44+1017.51-1017.53
F: FTMS + c NSI Full ms2 673.3515@hcd30.00
[110.0000-1395.1671] MS ICIS 26_3392C
100
50
0
26 28 30 32 34 36 38 40
Time (min)

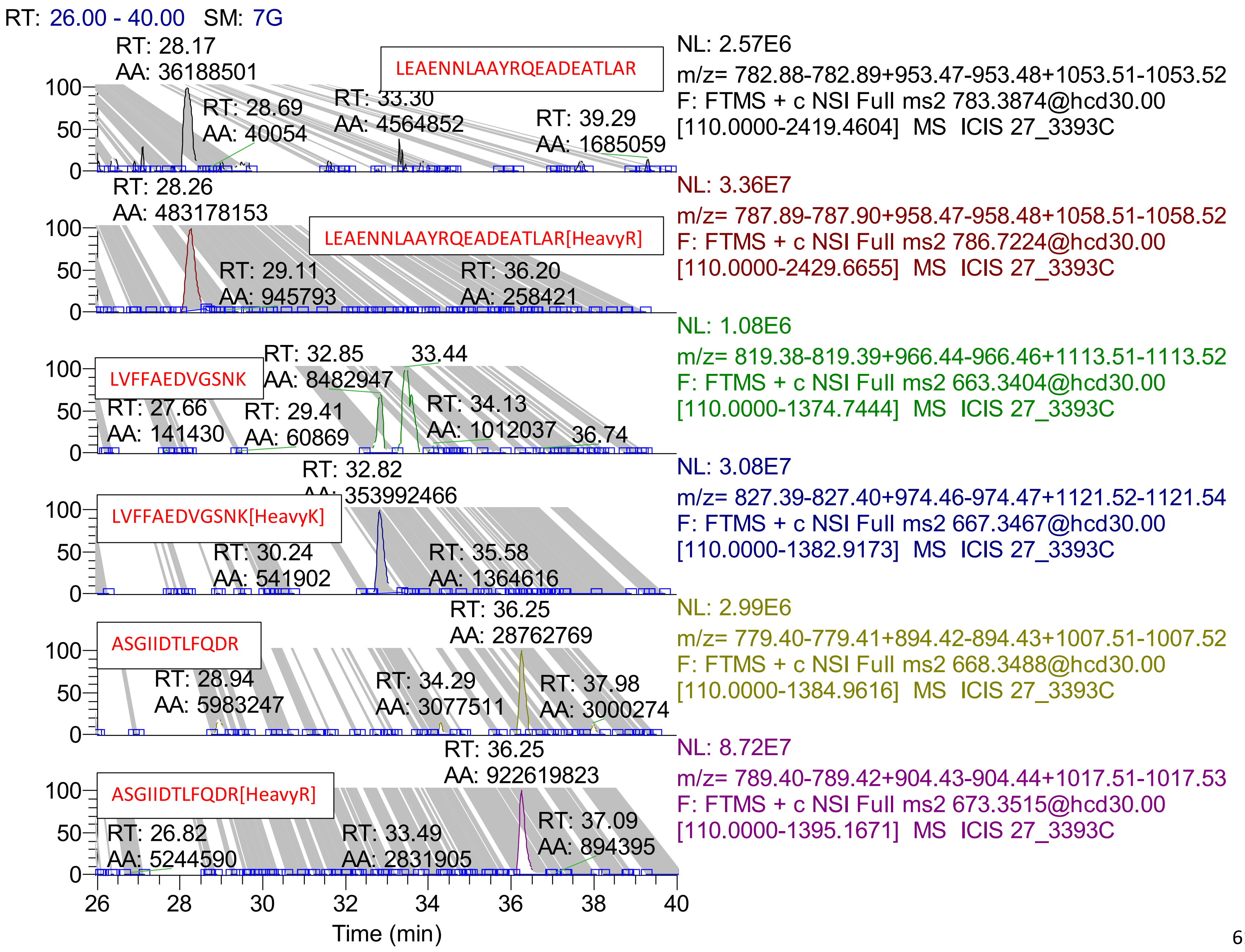
RT: 26.00 - 40.00 SM: 7G
RT: 28.17
AA: 36188501
LEAENNLAAYRQEADEATLAR
RT: 28.69
AA: 40054
RT: 33.30
AA: 4564852
RT: 39.29
AA: 1685059
NL: 2.57E6
m/z= 782.88-782.89+953.47-953.48+1053.51-1053.52
F: FTMS + c NSI Full ms2 783.3874@hcd30.00
[110.0000-2419.4604] MS ICIS 27_3393C
RT: 28.26
AA: 483178153
LEAENNLAAYRQEADEATLAR[HeavyR]
RT: 29.11
AA: 945793
RT: 36.20
AA: 258421
NL: 3.36E7
m/z= 787.89-787.90+958.47-958.48+1058.51-1058.52
F: FTMS + c NSI Full ms2 786.7224@hcd30.00
[110.0000-2429.6655] MS ICIS 27_3393C
LVFFAEDVGSNK
RT: 32.85
AA: 8482947
33.44
RT: 27.66
AA: 141430
RT: 29.41
AA: 60869
RT: 34.13
AA: 1012037
36.74
NL: 1.08E6
m/z= 819.38-819.39+966.44-966.46+1113.51-1113.52
F: FTMS + c NSI Full ms2 663.3404@hcd30.00
[110.0000-1374.7444] MS ICIS 27_3393C
RT: 32.82
353992466
LVFFAEDVGSNK[HeavyK]
RT: 30.24
AA: 541902
RT: 35.58
AA: 1364616
NL: 3.08E7
m/z= 827.39-827.40+974.46-974.47+1121.52-1121.54
F: FTMS + c NSI Full ms2 667.3467@hcd30.00
[110.0000-1382.9173] MS ICIS 27_3393C
RT: 36.25
AA: 28762769
ASGIIDTLFQDR
RT: 28.94
AA: 5983247
RT: 34.29
AA: 3077511
RT: 37.98
AA: 3000274
NL: 2.99E6
m/z= 779.40-779.41+894.42-894.43+1007.51-1007.52
F: FTMS + c NSI Full ms2 668.3488@hcd30.00
[110.0000-1384.9616] MS ICIS 27_3393C
RT: 36.25
AA: 922619823
ASGIIDTLFQDR[HeavyR]
RT: 26.82
AA: 5244590
RT: 33.49
AA: 2831905
RT: 37.09
AA: 894395
NL: 8.72E7
m/z= 789.40-789.42+904.43-904.44+1017.51-1017.53
F: FTMS + c NSI Full ms2 673.3515@hcd30.00
[110.0000-1395.1671] MS ICIS 27_3393C
100
50
0
26
28
30
32
34
36
38
40
Time (min)

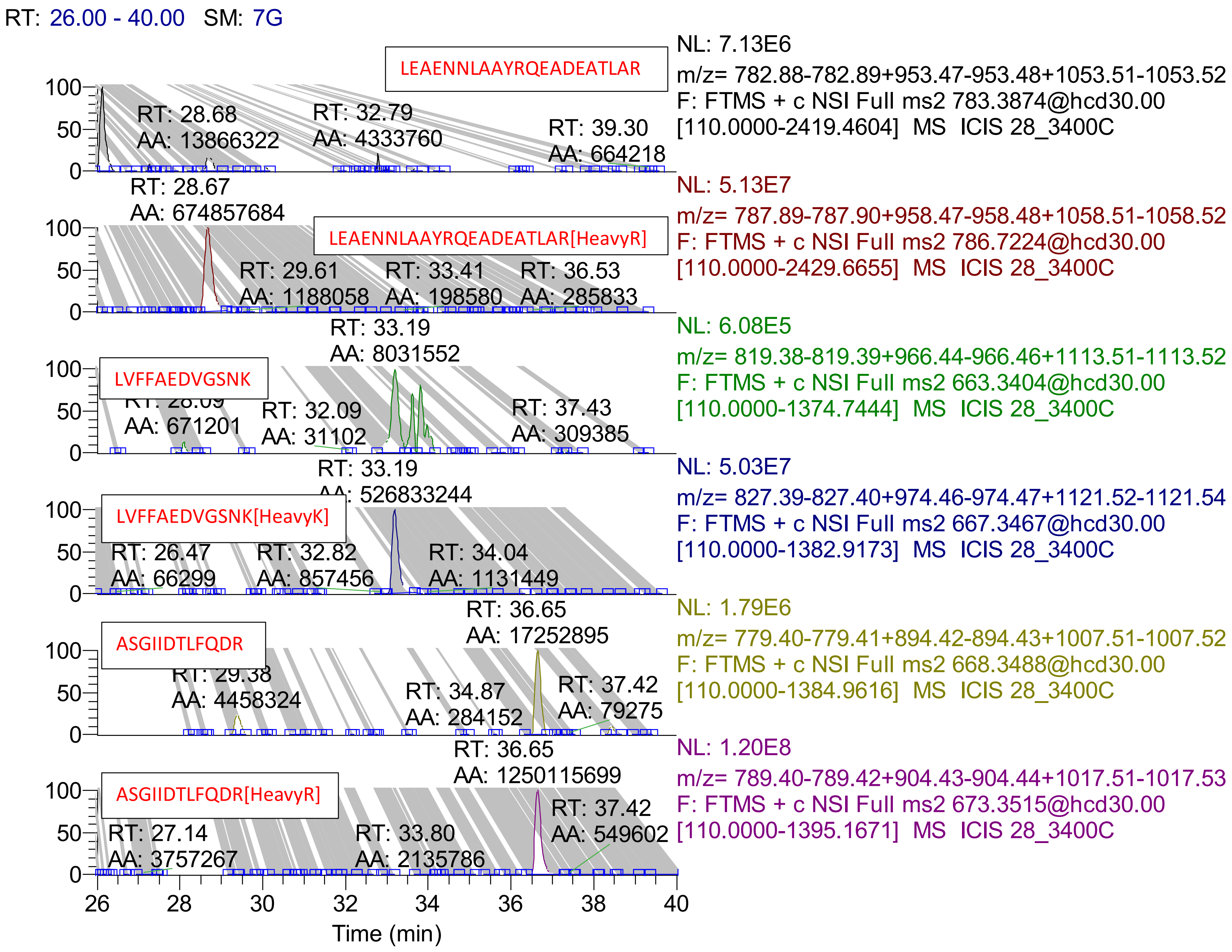

RT: 26.00 - 40.00 SM: 7G
LEAENNLAAYRQEADEATLAR
NL: 7.13E6
m/z= 782.88-782.89+953.47-953.48+1053.51-1053.52
F: FTMS + c NSI Full ms2 783.3874@hcd30.00
[110.0000-2419.4604] MS ICIS 28_3400C
RT: 28.68
AA: 13866322
RT: 32.79
AA: 4333760
RT: 39.30
AA: 664218
RT: 28.67
AA: 674857684
LEAENNLAAYRQEADEATLAR[HeavyR]
NL: 5.13E7
m/z= 787.89-787.90+958.47-958.48+1058.51-1058.52
F: FTMS + c NSI Full ms2 786.7224@hcd30.00
[110.0000-2429.6655] MS ICIS 28_3400C
RT: 29.61
AA: 1188058
RT: 33.41
AA: 198580
RT: 36.53
AA: 285833
RT: 33.19
AA: 8031552
LVFFAEDVGSNK
NL: 6.08E5
m/z= 819.38-819.39+966.44-966.46+1113.51-1113.52
F: FTMS + c NSI Full ms2 663.3404@hcd30.00
[110.0000-1374.7444] MS ICIS 28_3400C
AA: 671201
RT: 32.09
AA: 31102
RT: 37.43
AA: 309385
RT: 33.19
LVFFAEDVGSNK[HeavyK]
NL: 5.03E7
m/z= 827.39-827.40+974.46-974.47+1121.52-1121.54
F: FTMS + c NSI Full ms2 667.3467@hcd30.00
[110.0000-1382.9173] MS ICIS 28_3400C
RT: 26.47
AA: 66299
RT: 32.82
AA: 857456
RT: 34.04
AA: 1131449
RT: 36.65
AA: 17252895
ASGIIDTLFQDR
NL: 1.79E6
m/z= 779.40-779.41+894.42-894.43+1007.51-1007.52
F: FTMS + c NSI Full ms2 668.3488@hcd30.00
[110.0000-1384.9616] MS ICIS 28_3400C
AA: 4458324
RT: 34.87
AA: 284152
RT: 37.42
AA: 79275
RT: 36.65
AA: 1250115699
ASGIIDTLFQDR[HeavyR]
NL: 1.20E8
m/z= 789.40-789.42+904.43-904.44+1017.51-1017.53
F: FTMS + c NSI Full ms2 673.3515@hcd30.00
[110.0000-1395.1671] MS ICIS 28_3400C
RT: 27.14
AA: 3757267
RT: 33.80
AA: 2135786
RT: 37.42
AA: 549602
100
50
0
26 28 30 32 34 36 38 40
Time (min)

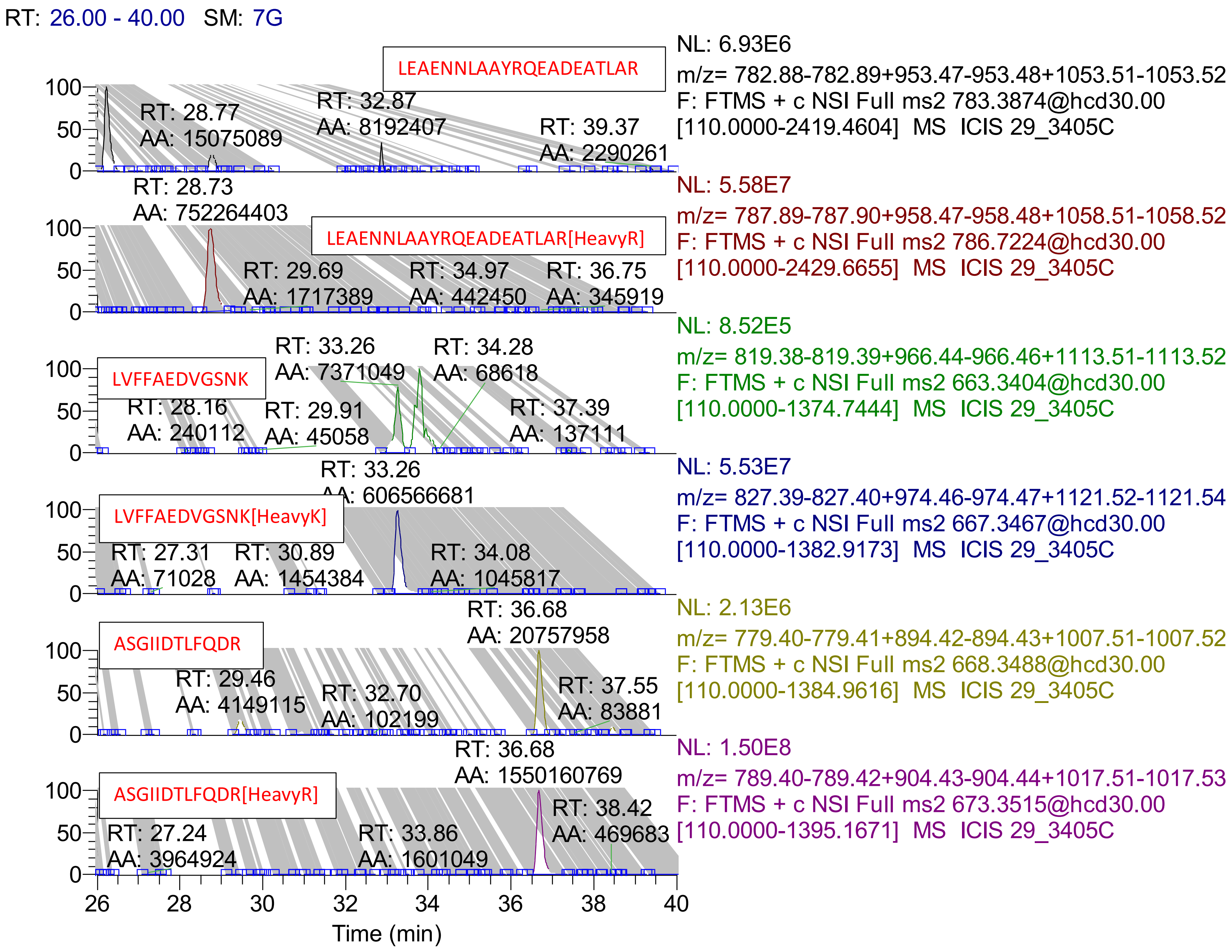

RT: 26.00 - 40.00 SM: 7G
LEAEANNLAAYRQEADEATLAR
NL: 6.93E6
m/z= 782.88-782.89+953.47-953.48+1053.51-1053.52
F: FTMS + c NSI Full ms2 783.3874@hcd30.00
[110.0000-2419.4604] MS ICIS 29_3405C
RT: 28.77
AA: 15075089
RT: 32.87
AA: 8192407
RT: 39.37
AA: 2290261
RT: 28.73
AA: 752264403
LEAEANNLAAYRQEADEATLAR[HeavyR]
NL: 5.58E7
m/z= 787.89-787.90+958.47-958.48+1058.51-1058.52
F: FTMS + c NSI Full ms2 786.7224@hcd30.00
[110.0000-2429.6655] MS ICIS 29_3405C
RT: 29.69
AA: 1717389
RT: 34.97
AA: 442450
RT: 36.75
AA: 345919
LVFFAEDVGSNK
NL: 8.52E5
m/z= 819.38-819.39+966.44-966.46+1113.51-1113.52
F: FTMS + c NSI Full ms2 663.3404@hcd30.00
[110.0000-1374.7444] MS ICIS 29_3405C
RT: 33.26
AA: 7371049
RT: 34.28
AA: 68618
RT: 28.16
AA: 240112
RT: 29.91
AA: 45058
RT: 37.39
AA: 137111
RT: 33.26
AA: 606566681
LVFFAEDVGSNK[HeavyK]
NL: 5.53E7
m/z= 827.39-827.40+974.46-974.47+1121.52-1121.54
F: FTMS + c NSI Full ms2 667.3467@hcd30.00
[110.0000-1382.9173] MS ICIS 29_3405C
RT: 27.31
AA: 71028
RT: 30.89
AA: 1454384
RT: 34.08
AA: 1045817
RT: 36.68
AA: 20757958
ASGIIDTLFQDR
NL: 2.13E6
m/z= 779.40-779.41+894.42-894.43+1007.51-1007.52
F: FTMS + c NSI Full ms2 668.3488@hcd30.00
[110.0000-1384.9616] MS ICIS 29_3405C
RT: 29.46
AA: 4149115
RT: 32.70
AA: 102199
RT: 37.55
AA: 83881
RT: 36.68
AA: 1550160769
ASGIIDTLFQDR[HeavyR]
NL: 1.50E8
m/z= 789.40-789.42+904.43-904.44+1017.51-1017.53
F: FTMS + c NSI Full ms2 673.3515@hcd30.00
[110.0000-1395.1671] MS ICIS 29_3405C
RT: 38.42
AA: 469683
RT: 27.24
AA: 3964924
RT: 33.86
AA: 1601049
100
50
0
26
28
30
32
34
36
38
40
Time (min)

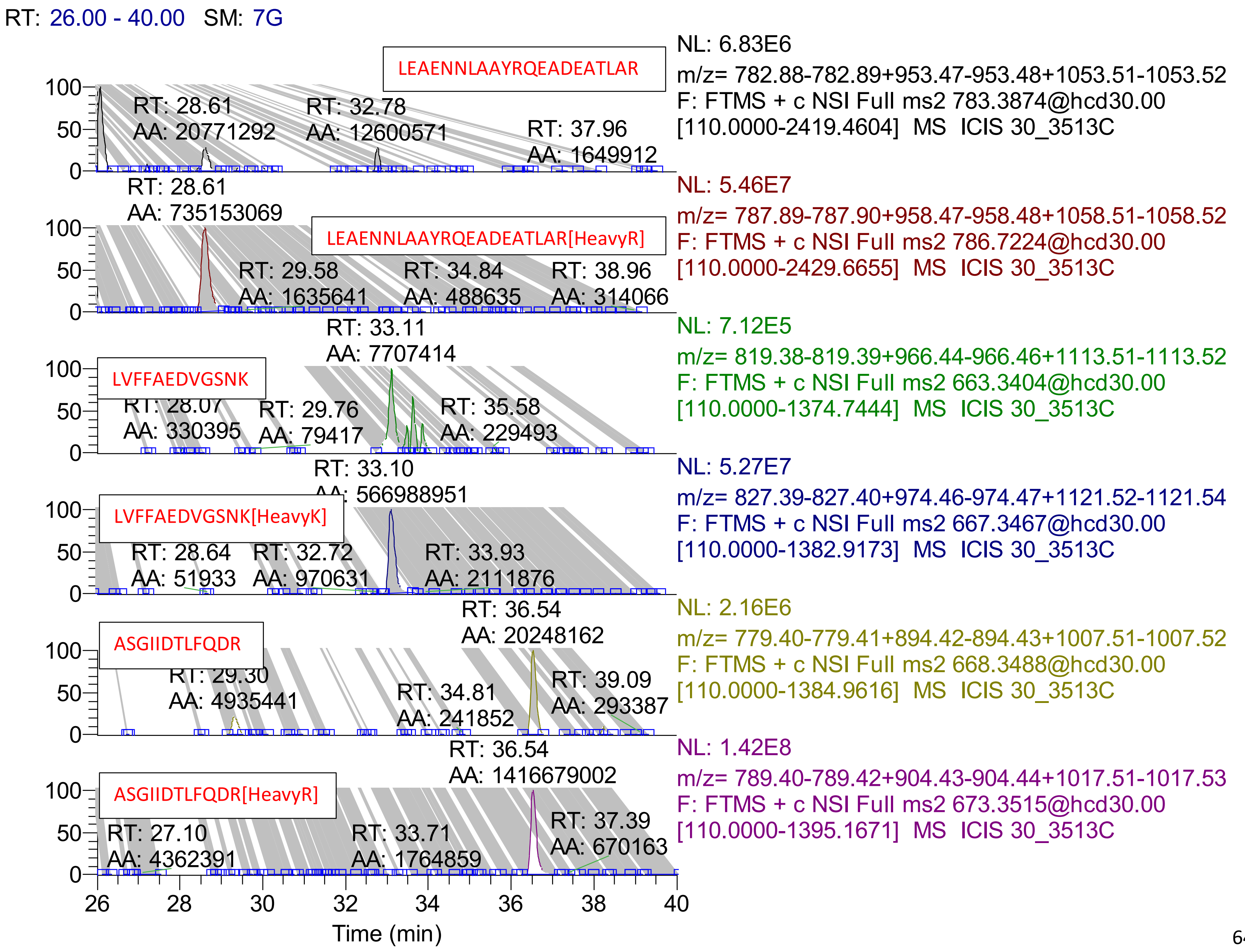

RT: 26.00 - 40.00 SM: 7G
LEAENNLAAYRQEADEATLAR
RT: 28.61 AA: 20771292
RT: 32.78 AA: 12600571
RT: 37.96 AA: 1649912
NL: 6.83E6
m/z= 782.88-782.89+953.47-953.48+1053.51-1053.52
F: FTMS + c NSI Full ms2 783.3874@hcd30.00
[110.0000-2419.4604] MS ICIS 30_3513C
RT: 28.61 AA: 735153069
LEAENNLAAYRQEADEATLAR[HeavyR]
RT: 29.58 AA: 1635641
RT: 34.84 AA: 488635
RT: 38.96 AA: 314066
NL: 5.46E7
m/z= 787.89-787.90+958.47-958.48+1058.51-1058.52
F: FTMS + c NSI Full ms2 786.7224@hcd30.00
[110.0000-2429.6655] MS ICIS 30_3513C
RT: 33.11 AA: 7707414
LVFFAEDVGSNK
AA: 330395
RT: 29.76 AA: 79417
RT: 35.58 AA: 229493
NL: 7.12E5
m/z= 819.38-819.39+966.44-966.46+1113.51-1113.52
F: FTMS + c NSI Full ms2 663.3404@hcd30.00
[110.0000-1374.7444] MS ICIS 30_3513C
RT: 33.10
LVFFAEDVGSNK[HeavyK]
RT: 28.64 AA: 51933
RT: 32.72 AA: 970631
RT: 33.93 AA: 2111876
NL: 5.27E7
m/z= 827.39-827.40+974.46-974.47+1121.52-1121.54
F: FTMS + c NSI Full ms2 667.3467@hcd30.00
[110.0000-1382.9173] MS ICIS 30_3513C
RT: 36.54 AA: 20248162
ASGIIDTLFQDR
AA: 4935441
RT: 34.81 AA: 241852
RT: 39.09 AA: 293387
NL: 2.16E6
m/z= 779.40-779.41+894.42-894.43+1007.51-1007.52
F: FTMS + c NSI Full ms2 668.3488@hcd30.00
[110.0000-1384.9616] MS ICIS 30_3513C
RT: 36.54 AA: 1416679002
ASGIIDTLFQDR[HeavyR]
RT: 27.10 AA: 4362391
RT: 33.71 AA: 1764859
RT: 37.39 AA: 670163
NL: 1.42E8
m/z= 789.40-789.42+904.43-904.44+1017.51-1017.53
F: FTMS + c NSI Full ms2 673.3515@hcd30.00
[110.0000-1395.1671] MS ICIS 30_3513C
100
50
0
26 28 30 32 34 36 38 40
Time (min)

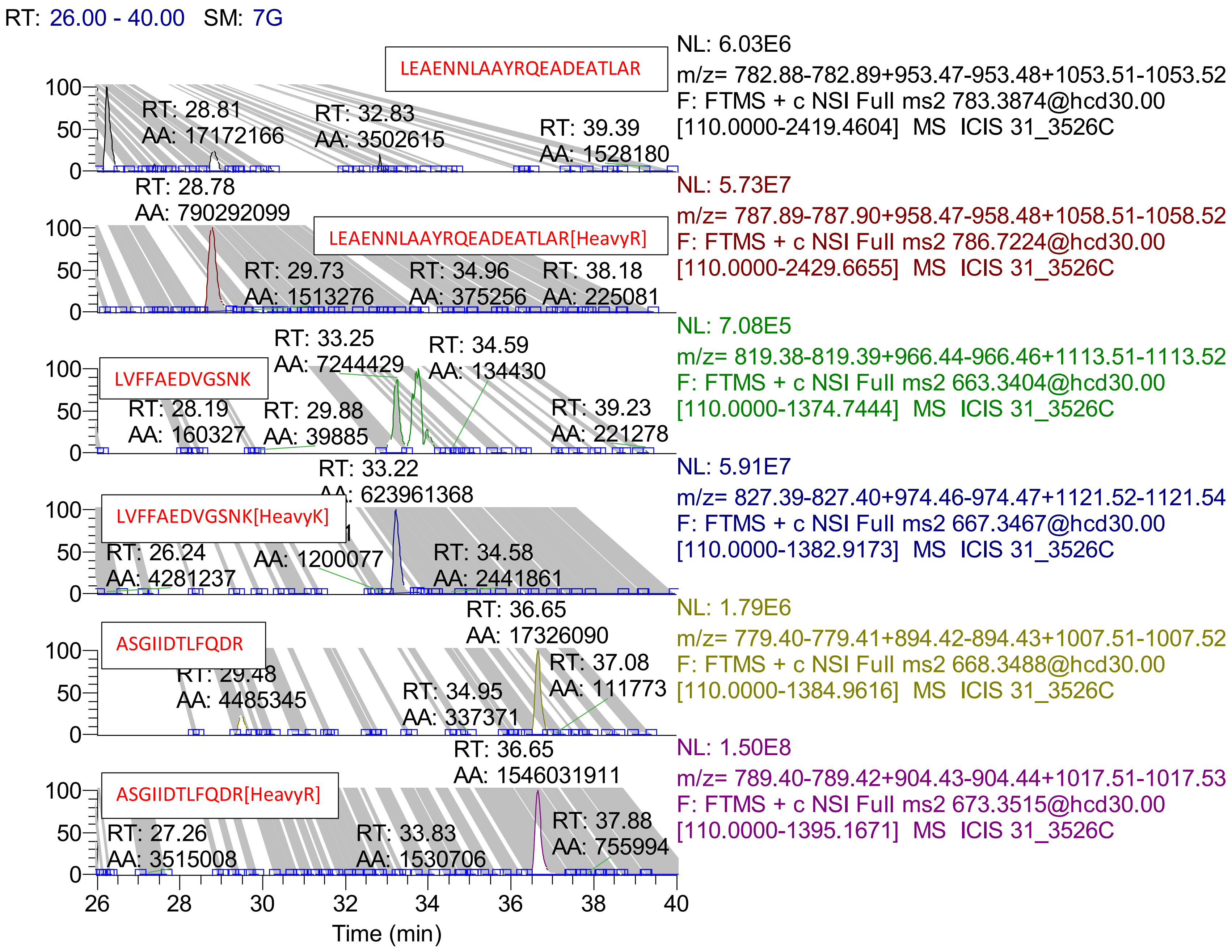

RT: 26.00 - 40.00 SM: 7G
LEAENNLAAYRQEADEATLAR
NL: 6.03E6
m/z= 782.88-782.89+953.47-953.48+1053.51-1053.52
F: FTMS + c NSI Full ms2 783.3874@hcd30.00
[110.0000-2419.4604] MS ICIS 31_3526C
RT: 28.81
AA: 17172166
RT: 32.83
AA: 3502615
RT: 39.39
AA: 1528180
RT: 28.78
AA: 790292099
LEAENNLAAYRQEADEATLAR[HeavyR]
NL: 5.73E7
m/z= 787.89-787.90+958.47-958.48+1058.51-1058.52
F: FTMS + c NSI Full ms2 786.7224@hcd30.00
[110.0000-2429.6655] MS ICIS 31_3526C
RT: 29.73
AA: 1513276
RT: 34.96
AA: 375256
RT: 38.18
AA: 225081
LVFFAEDVGSNK
NL: 7.08E5
m/z= 819.38-819.39+966.44-966.46+1113.51-1113.52
F: FTMS + c NSI Full ms2 663.3404@hcd30.00
[110.0000-1374.7444] MS ICIS 31_3526C
RT: 33.25
AA: 7244429
RT: 34.59
AA: 134430
RT: 28.19
AA: 160327
RT: 29.88
AA: 39885
RT: 39.23
AA: 221278
RT: 33.22
AA: 623961368
LVFFAEDVGSNK[HeavyK]
NL: 5.91E7
m/z= 827.39-827.40+974.46-974.47+1121.52-1121.54
F: FTMS + c NSI Full ms2 667.3467@hcd30.00
[110.0000-1382.9173] MS ICIS 31_3526C
RT: 26.24
AA: 4281237
AA: 1200077
RT: 34.58
AA: 2441861
RT: 36.65
AA: 17326090
ASGIIDTLFQDR
NL: 1.79E6
m/z= 779.40-779.41+894.42-894.43+1007.51-1007.52
F: FTMS + c NSI Full ms2 668.3488@hcd30.00
[110.0000-1384.9616] MS ICIS 31_3526C
RT: 37.08
AA: 111773
AA: 4485345
RT: 34.95
AA: 337371
RT: 36.65
AA: 1546031911
ASGIIDTLFQDR[HeavyR]
NL: 1.50E8
m/z= 789.40-789.42+904.43-904.44+1017.51-1017.53
F: FTMS + c NSI Full ms2 673.3515@hcd30.00
[110.0000-1395.1671] MS ICIS 31_3526C
RT: 27.26
AA: 3515008
RT: 33.83
AA: 1530706
RT: 37.88
AA: 755994
0
50
100
26
28
30
32
34
36
38
40
Time (min)

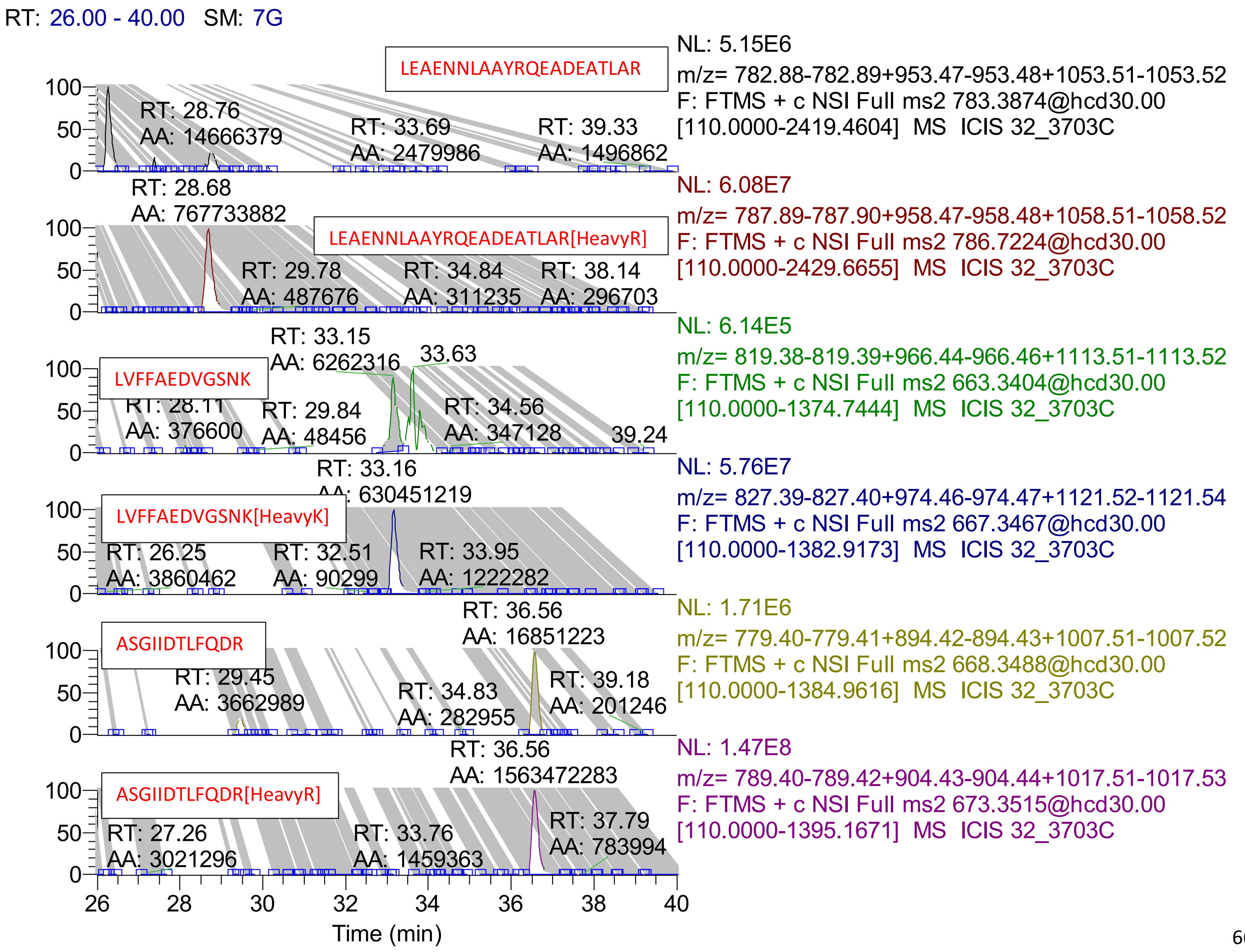

RT: 26.00 - 40.00 SM: 7G
LEAENNLAAYRQEADEATLAR
NL: 5.15E6
m/z= 782.88-782.89+953.47-953.48+1053.51-1053.52
F: FTMS + c NSI Full ms2 783.3874@hcd30.00
[110.0000-2419.4604] MS ICIS 32_3703C
RT: 28.76
AA: 14666379
RT: 33.69
AA: 2479986
RT: 39.33
AA: 1496862
RT: 28.68
AA: 767733882
LEAENNLAAYRQEADEATLAR[HeavyR]
NL: 6.08E7
m/z= 787.89-787.90+958.47-958.48+1058.51-1058.52
F: FTMS + c NSI Full ms2 786.7224@hcd30.00
[110.0000-2429.6655] MS ICIS 32_3703C
RT: 29.78
AA: 487676
RT: 34.84
AA: 311235
RT: 38.14
AA: 296703
LVFFAEDVGSNK
NL: 6.14E5
m/z= 819.38-819.39+966.44-966.46+1113.51-1113.52
F: FTMS + c NSI Full ms2 663.3404@hcd30.00
[110.0000-1374.7444] MS ICIS 32_3703C
RT: 33.15
AA: 6262316
33.63
AA: 376600
RT: 29.84
AA: 48456
RT: 34.56
AA: 347128
39.24
LVFFAEDVGSNK[HeavyK]
NL: 5.76E7
m/z= 827.39-827.40+974.46-974.47+1121.52-1121.54
F: FTMS + c NSI Full ms2 667.3467@hcd30.00
[110.0000-1382.9173] MS ICIS 32_3703C
RT: 33.16
RT: 26.25
AA: 3860462
RT: 32.51
AA: 90299
RT: 33.95
AA: 1222282
ASGIIDTLFQDR
NL: 1.71E6
m/z= 779.40-779.41+894.42-894.43+1007.51-1007.52
F: FTMS + c NSI Full ms2 668.3488@hcd30.00
[110.0000-1384.9616] MS ICIS 32_3703C
RT: 36.56
AA: 16851223
RT: 29.45
AA: 3662989
RT: 34.83
AA: 282955
RT: 39.18
AA: 201246
ASGIIDTLFQDR[HeavyR]
NL: 1.47E8
m/z= 789.40-789.42+904.43-904.44+1017.51-1017.53
F: FTMS + c NSI Full ms2 673.3515@hcd30.00
[110.0000-1395.1671] MS ICIS 32_3703C
RT: 36.56
AA: 1563472283
RT: 27.26
AA: 3021296
RT: 33.76
AA: 1459363
RT: 37.79
AA: 783994
100
50
0
26
28
30
32
34
36
38
40
Time (min)

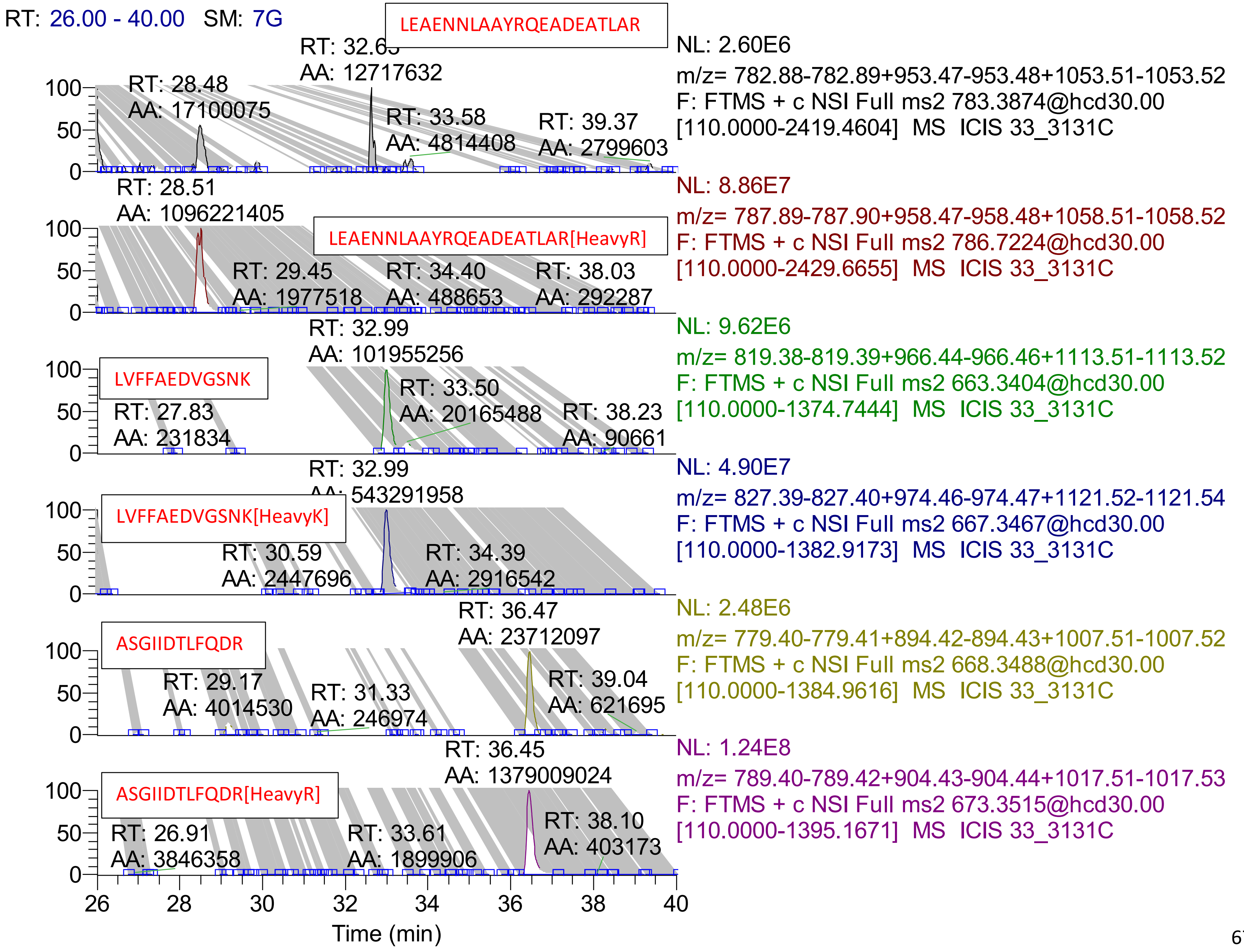
RT: 26.00 - 40.00 SM: 7G
LEAENNLAAYRQEADEATLAR
RT: 32.6
AA: 12717632
RT: 28.48
AA: 17100075
RT: 33.58
AA: 4814408
RT: 39.37
AA: 2799603
NL: 2.60E6
m/z= 782.88-782.89+953.47-953.48+1053.51-1053.52
F: FTMS + c NSI Full ms2 783.3874@hcd30.00
[110.0000-2419.4604] MS ICIS 33_3131C
RT: 28.51
AA: 1096221405
LEAENNLAAYRQEADEATLAR[HeavyR]
RT: 29.45
AA: 1977518
RT: 34.40
AA: 488653
RT: 38.03
AA: 292287
NL: 8.86E7
m/z= 787.89-787.90+958.47-958.48+1058.51-1058.52
F: FTMS + c NSI Full ms2 786.7224@hcd30.00
[110.0000-2429.6655] MS ICIS 33_3131C
RT: 32.99
AA: 101955256
LVFFAEDVGSNK
RT: 27.83
AA: 231834
RT: 33.50
AA: 20165488
RT: 38.23
AA: 90661
NL: 9.62E6
m/z= 819.38-819.39+966.44-966.46+1113.51-1113.52
F: FTMS + c NSI Full ms2 663.3404@hcd30.00
[110.0000-1374.7444] MS ICIS 33_3131C
RT: 32.99
AA: 543291958
LVFFAEDVGSNK[HeavyK]
RT: 30.59
AA: 2447696
RT: 34.39
AA: 2916542
NL: 4.90E7
m/z= 827.39-827.40+974.46-974.47+1121.52-1121.54
F: FTMS + c NSI Full ms2 667.3467@hcd30.00
[110.0000-1382.9173] MS ICIS 33_3131C
RT: 36.47
AA: 23712097
ASGIIDTLFQDR
RT: 29.17
AA: 4014530
RT: 31.33
AA: 246974
RT: 39.04
AA: 621695
NL: 2.48E6
m/z= 779.40-779.41+894.42-894.43+1007.51-1007.52
F: FTMS + c NSI Full ms2 668.3488@hcd30.00
[110.0000-1384.9616] MS ICIS 33_3131C
RT: 36.45
AA: 1379009024
ASGIIDTLFQDR[HeavyR]
RT: 26.91
AA: 3846358
RT: 33.61
AA: 1899906
RT: 38.10
AA: 403173
NL: 1.24E8
m/z= 789.40-789.42+904.43-904.44+1017.51-1017.53
F: FTMS + c NSI Full ms2 673.3515@hcd30.00
[110.0000-1395.1671] MS ICIS 33_3131C
100
50
0
26
28
30
32
34
36
38
40
Time (min)

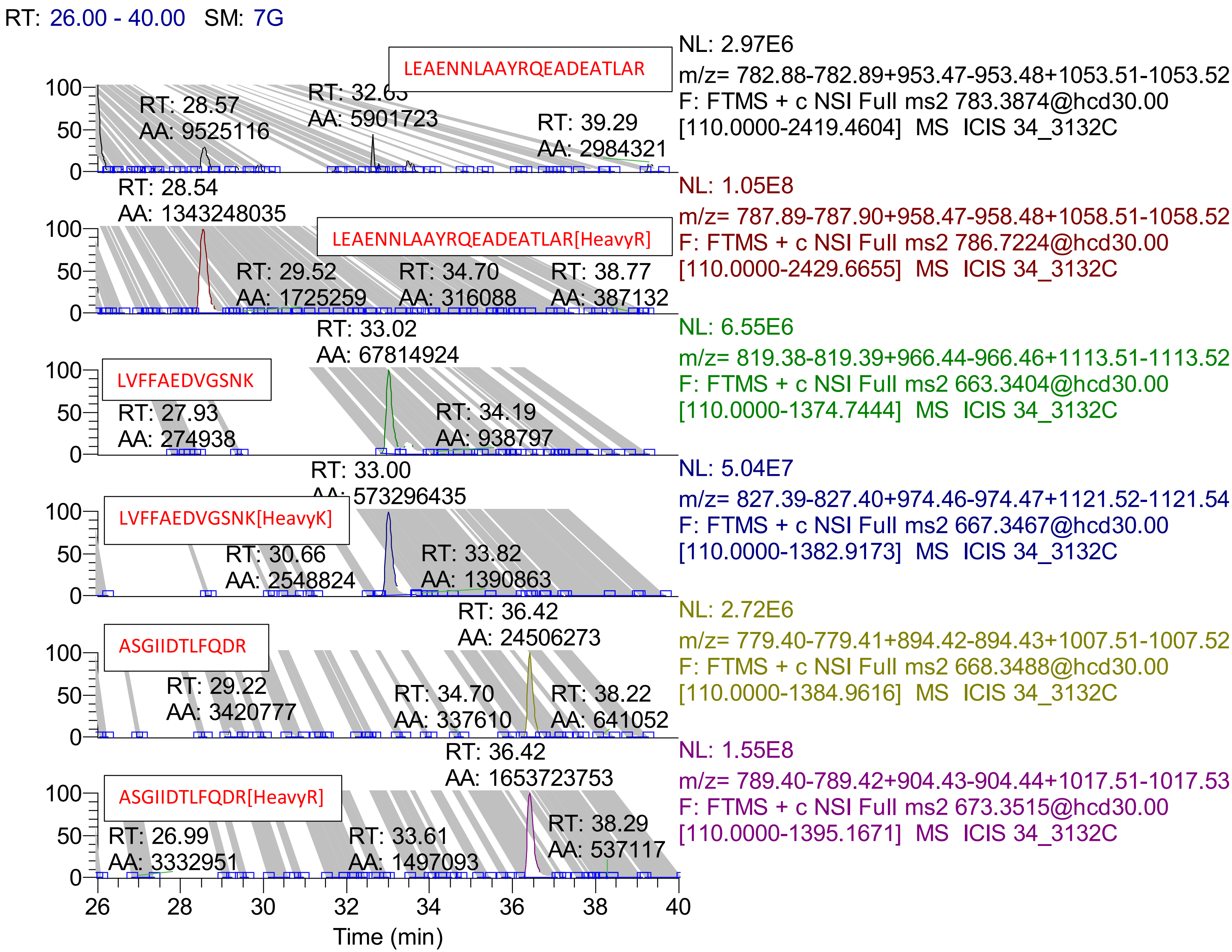

RT: 26.00 - 40.00 SM: 7G
LEAENNLAAYRQEADEATLAR
RT: 28.57
AA: 9525116
RT: 32.63
AA: 5901723
RT: 39.29
AA: 2984321
NL: 2.97E6
m/z= 782.88-782.89+953.47-953.48+1053.51-1053.52
F: FTMS + c NSI Full ms2 783.3874@hcd30.00
[110.0000-2419.4604] MS ICIS 34_3132C
RT: 28.54
AA: 1343248035
LEAENNLAAYRQEADEATLAR[HeavyR]
RT: 29.52
AA: 1725259
RT: 34.70
AA: 316088
RT: 38.77
AA: 387132
NL: 1.05E8
m/z= 787.89-787.90+958.47-958.48+1058.51-1058.52
F: FTMS + c NSI Full ms2 786.7224@hcd30.00
[110.0000-2429.6655] MS ICIS 34_3132C
RT: 33.02
AA: 67814924
LVFFAEDVGSNK
RT: 27.93
AA: 274938
RT: 34.19
AA: 938797
NL: 6.55E6
m/z= 819.38-819.39+966.44-966.46+1113.51-1113.52
F: FTMS + c NSI Full ms2 663.3404@hcd30.00
[110.0000-1374.7444] MS ICIS 34_3132C
RT: 33.00
AA: 573296435
LVFFAEDVGSNK[HeavyK]
RT: 30.66
AA: 2548824
RT: 33.82
AA: 1390863
NL: 5.04E7
m/z= 827.39-827.40+974.46-974.47+1121.52-1121.54
F: FTMS + c NSI Full ms2 667.3467@hcd30.00
[110.0000-1382.9173] MS ICIS 34_3132C
RT: 36.42
AA: 24506273
ASGIIDTLFQDR
RT: 29.22
AA: 3420777
RT: 34.70
AA: 337610
RT: 38.22
AA: 641052
NL: 2.72E6
m/z= 779.40-779.41+894.42-894.43+1007.51-1007.52
F: FTMS + c NSI Full ms2 668.3488@hcd30.00
[110.0000-1384.9616] MS ICIS 34_3132C
RT: 36.42
AA: 1653723753
ASGIIDTLFQDR[HeavyR]
RT: 26.99
AA: 3332951
RT: 33.61
AA: 1497093
RT: 38.29
AA: 537117
NL: 1.55E8
m/z= 789.40-789.42+904.43-904.44+1017.51-1017.53
F: FTMS + c NSI Full ms2 673.3515@hcd30.00
[110.0000-1395.1671] MS ICIS 34_3132C
100
50
0
26
28
30
32
34
36
38
40
Time (min)

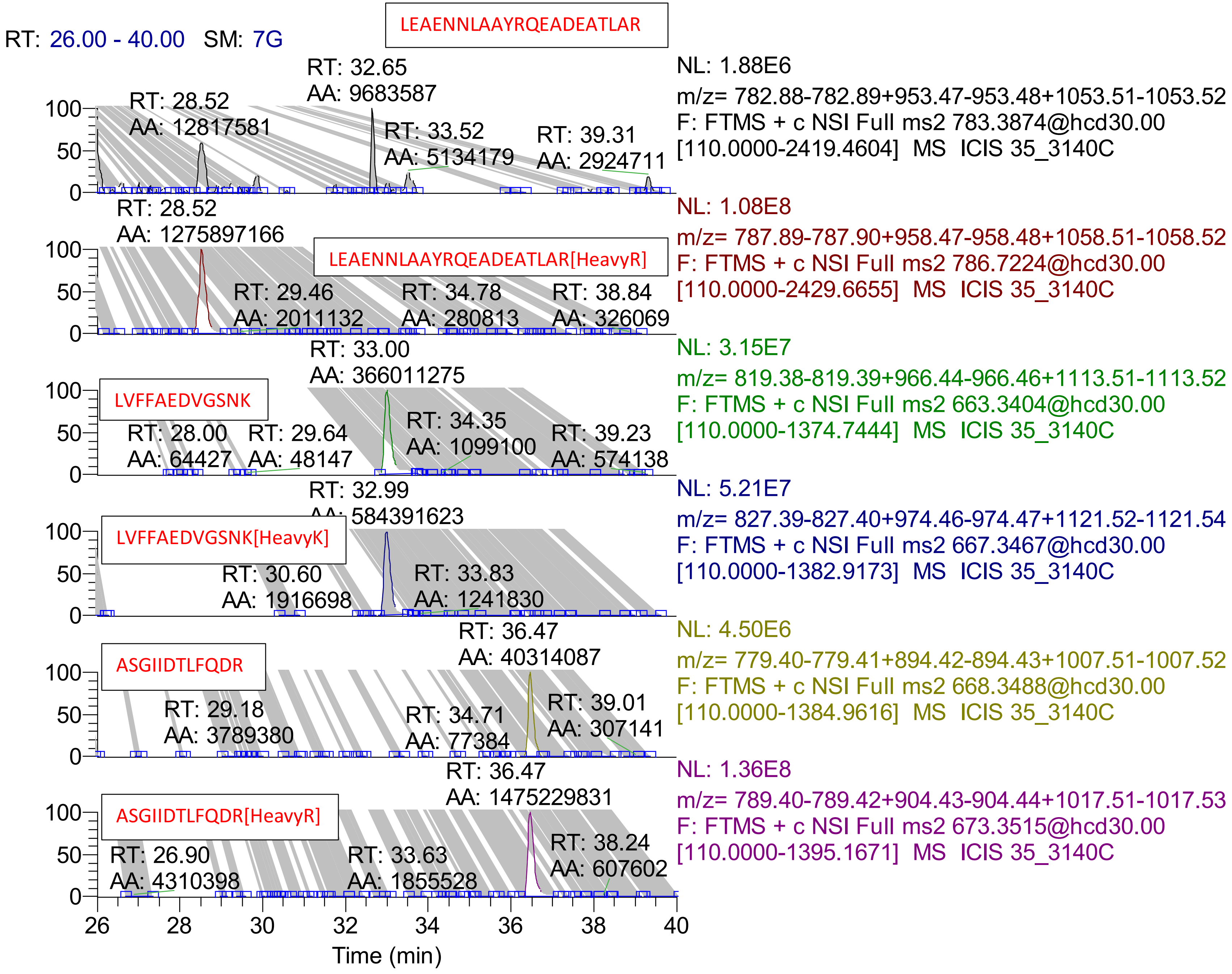

RT: 26.00 - 40.00 SM: 7G
LEAENNLAAYRQEADEATLAR
RT: 32.65
AA: 9683587
RT: 28.52
AA: 12817581
RT: 33.52
AA: 5134179
RT: 39.31
AA: 2924711
NL: 1.88E6
m/z= 782.88-782.89+953.47-953.48+1053.51-1053.52
F: FTMS + c NSI Full ms2 783.3874@hcd30.00
[110.0000-2419.4604] MS ICIS 35_3140C
RT: 28.52
AA: 1275897166
LEAENNLAAYRQEADEATLAR[HeavyR]
RT: 29.46
AA: 2011132
RT: 34.78
AA: 280813
RT: 38.84
AA: 326069
NL: 1.08E8
m/z= 787.89-787.90+958.47-958.48+1058.51-1058.52
F: FTMS + c NSI Full ms2 786.7224@hcd30.00
[110.0000-2429.6655] MS ICIS 35_3140C
RT: 33.00
AA: 366011275
LVFFAEDVGSNK
RT: 28.00
AA: 64427
RT: 29.64
AA: 48147
RT: 34.35
AA: 1099100
RT: 39.23
AA: 574138
NL: 3.15E7
m/z= 819.38-819.39+966.44-966.46+1113.51-1113.52
F: FTMS + c NSI Full ms2 663.3404@hcd30.00
[110.0000-1374.7444] MS ICIS 35_3140C
RT: 32.99
AA: 584391623
LVFFAEDVGSNK[HeavyK]
RT: 30.60
AA: 1916698
RT: 33.83
AA: 1241830
NL: 5.21E7
m/z= 827.39-827.40+974.46-974.47+1121.52-1121.54
F: FTMS + c NSI Full ms2 667.3467@hcd30.00
[110.0000-1382.9173] MS ICIS 35_3140C
RT: 36.47
AA: 40314087
ASGIIDTLFQDR
RT: 29.18
AA: 3789380
RT: 34.71
AA: 77384
RT: 39.01
AA: 307141
NL: 4.50E6
m/z= 779.40-779.41+894.42-894.43+1007.51-1007.52
F: FTMS + c NSI Full ms2 668.3488@hcd30.00
[110.0000-1384.9616] MS ICIS 35_3140C
RT: 36.47
AA: 1475229831
ASGIIDTLFQDR[HeavyR]
RT: 26.90
AA: 4310398
RT: 33.63
AA: 1855528
RT: 38.24
AA: 607602
NL: 1.36E8
m/z= 789.40-789.42+904.43-904.44+1017.51-1017.53
F: FTMS + c NSI Full ms2 673.3515@hcd30.00
[110.0000-1395.1671] MS ICIS 35_3140C
100
50
0
26 28 30 32 34 36 38 40
Time (min)

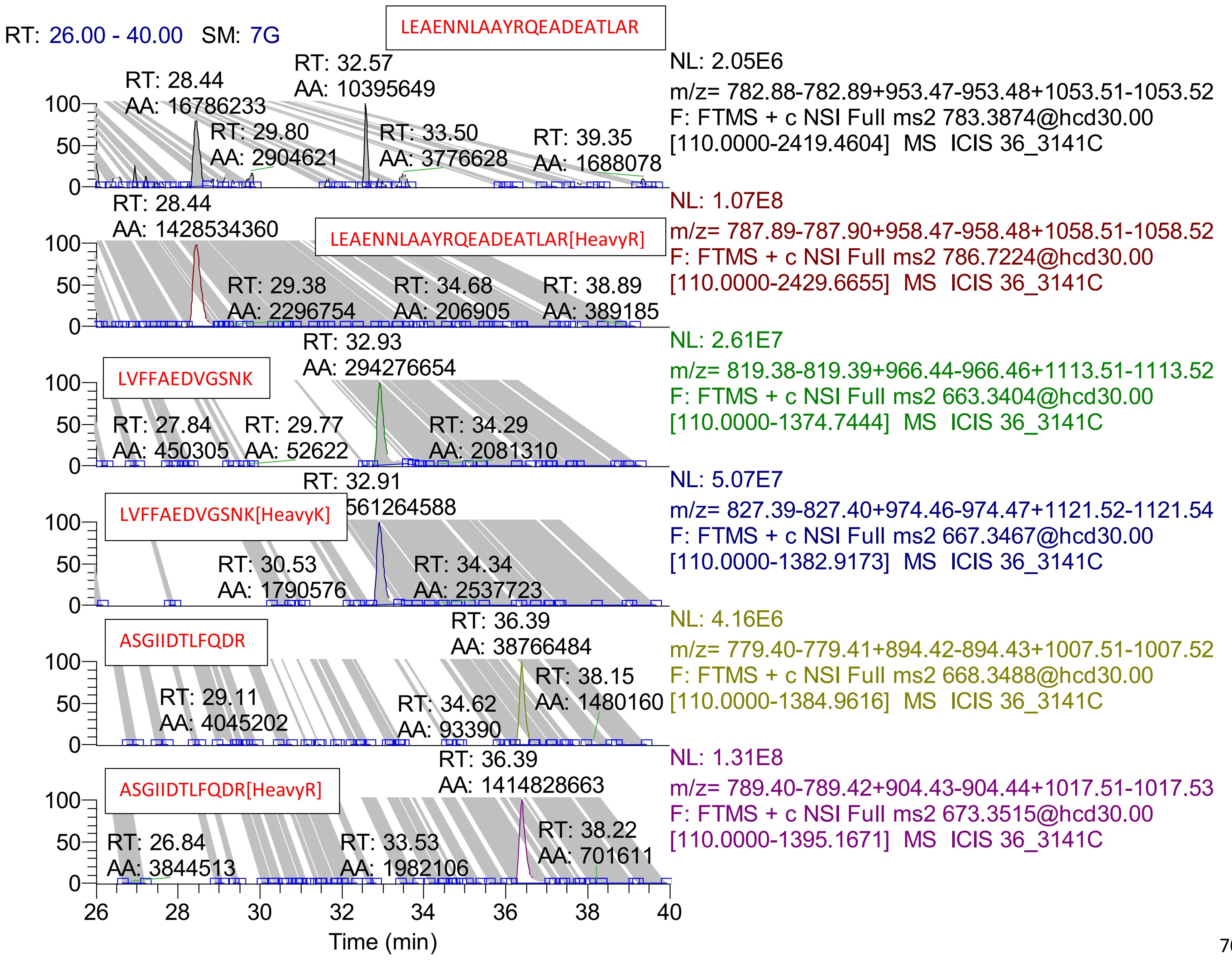

RT: 26.00 - 40.00 SM: 7G
LEAENNLAAYRQEADEATLAR
NL: 2.05E6
m/z= 782.88-782.89+953.47-953.48+1053.51-1053.52
F: FTMS + c NSI Full ms2 783.3874@hcd30.00
[110.0000-2419.4604] MS ICIS 36_3141C
RT: 28.44 AA: 16786233
RT: 32.57 AA: 10395649
RT: 29.80 AA: 2904621
RT: 33.50 AA: 3776628
RT: 39.35 AA: 1688078
LEAENNLAAYRQEADEATLAR[HeavyR]
NL: 1.07E8
m/z= 787.89-787.90+958.47-958.48+1058.51-1058.52
F: FTMS + c NSI Full ms2 786.7224@hcd30.00
[110.0000-2429.6655] MS ICIS 36_3141C
RT: 28.44 AA: 1428534360
RT: 29.38 AA: 2296754
RT: 34.68 AA: 206905
RT: 38.89 AA: 389185
LVFFAEDVGSNK
NL: 2.61E7
m/z= 819.38-819.39+966.44-966.46+1113.51-1113.52
F: FTMS + c NSI Full ms2 663.3404@hcd30.00
[110.0000-1374.7444] MS ICIS 36_3141C
RT: 32.93 AA: 294276654
RT: 27.84 AA: 450305
RT: 29.77 AA: 52622
RT: 34.29 AA: 2081310
LVFFAEDVGSNK[HeavyK]
NL: 5.07E7
m/z= 827.39-827.40+974.46-974.47+1121.52-1121.54
F: FTMS + c NSI Full ms2 667.3467@hcd30.00
[110.0000-1382.9173] MS ICIS 36_3141C
RT: 32.91 561264588
RT: 30.53 AA: 1790576
RT: 34.34 AA: 2537723
ASGIIDTLFQDR
NL: 4.16E6
m/z= 779.40-779.41+894.42-894.43+1007.51-1007.52
F: FTMS + c NSI Full ms2 668.3488@hcd30.00
[110.0000-1384.9616] MS ICIS 36_3141C
RT: 36.39 AA: 38766484
RT: 29.11 AA: 4045202
RT: 34.62 AA: 93390
RT: 38.15 AA: 1480160
ASGIIDTLFQDR[HeavyR]
NL: 1.31E8
m/z= 789.40-789.42+904.43-904.44+1017.51-1017.53
F: FTMS + c NSI Full ms2 673.3515@hcd30.00
[110.0000-1395.1671] MS ICIS 36_3141C
RT: 36.39 AA: 1414828663
RT: 26.84 AA: 3844513
RT: 33.53 AA: 1982106
RT: 38.22 AA: 701611
100
50
0
26 28 30 32 34 36 38 40
Time (min)

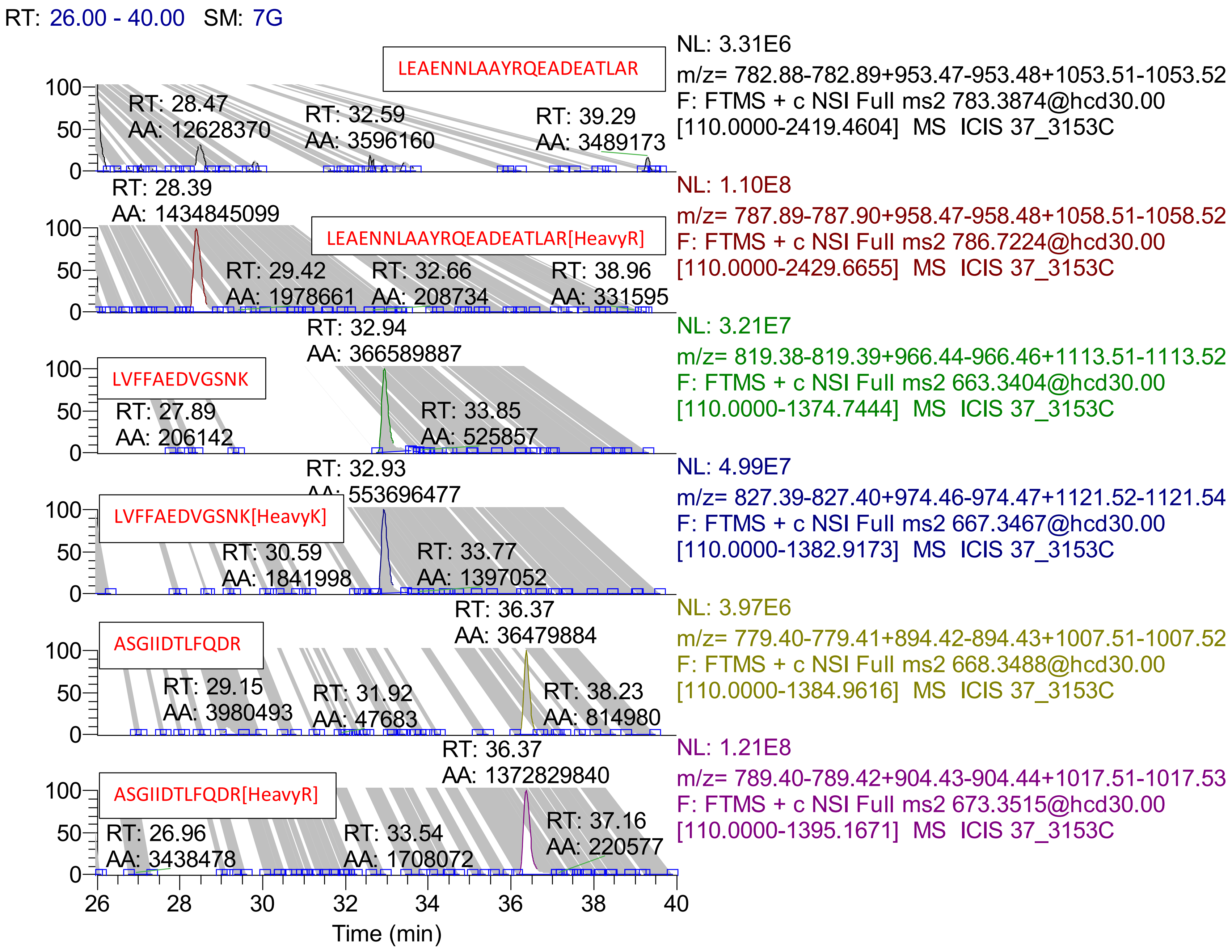

RT: 26.00 - 40.00 SM: 7G
LEAENNLAAYRQEADEATLAR
RT: 28.47
AA: 12628370
RT: 32.59
AA: 3596160
RT: 39.29
AA: 3489173
NL: 3.31E6
m/z= 782.88-782.89+953.47-953.48+1053.51-1053.52
F: FTMS + c NSI Full ms2 783.3874@hcd30.00
[110.0000-2419.4604] MS ICIS 37_3153C
RT: 28.39
AA: 1434845099
LEAENNLAAYRQEADEATLAR[HeavyR]
RT: 29.42
AA: 1978661
RT: 32.66
AA: 208734
RT: 38.96
AA: 331595
NL: 1.10E8
m/z= 787.89-787.90+958.47-958.48+1058.51-1058.52
F: FTMS + c NSI Full ms2 786.7224@hcd30.00
[110.0000-2429.6655] MS ICIS 37_3153C
RT: 32.94
AA: 366589887
LVFFAEDVGSNK
RT: 27.89
AA: 206142
RT: 33.85
AA: 525857
NL: 3.21E7
m/z= 819.38-819.39+966.44-966.46+1113.51-1113.52
F: FTMS + c NSI Full ms2 663.3404@hcd30.00
[110.0000-1374.7444] MS ICIS 37_3153C
RT: 32.93
AA: 553696477
LVFFAEDVGSNK[HeavyK]
RT: 30.59
AA: 1841998
RT: 33.77
AA: 1397052
NL: 4.99E7
m/z= 827.39-827.40+974.46-974.47+1121.52-1121.54
F: FTMS + c NSI Full ms2 667.3467@hcd30.00
[110.0000-1382.9173] MS ICIS 37_3153C
RT: 36.37
AA: 36479884
ASGIIDTLFQDR
RT: 29.15
AA: 3980493
RT: 31.92
AA: 47683
RT: 38.23
AA: 814980
NL: 3.97E6
m/z= 779.40-779.41+894.42-894.43+1007.51-1007.52
F: FTMS + c NSI Full ms2 668.3488@hcd30.00
[110.0000-1384.9616] MS ICIS 37_3153C
RT: 36.37
AA: 1372829840
ASGIIDTLFQDR[HeavyR]
RT: 26.96
AA: 3438478
RT: 33.54
AA: 1708072
RT: 37.16
AA: 220577
NL: 1.21E8
m/z= 789.40-789.42+904.43-904.44+1017.51-1017.53
F: FTMS + c NSI Full ms2 673.3515@hcd30.00
[110.0000-1395.1671] MS ICIS 37_3153C
100
50
0
26 28 30 32 34 36 38 40
Time (min)

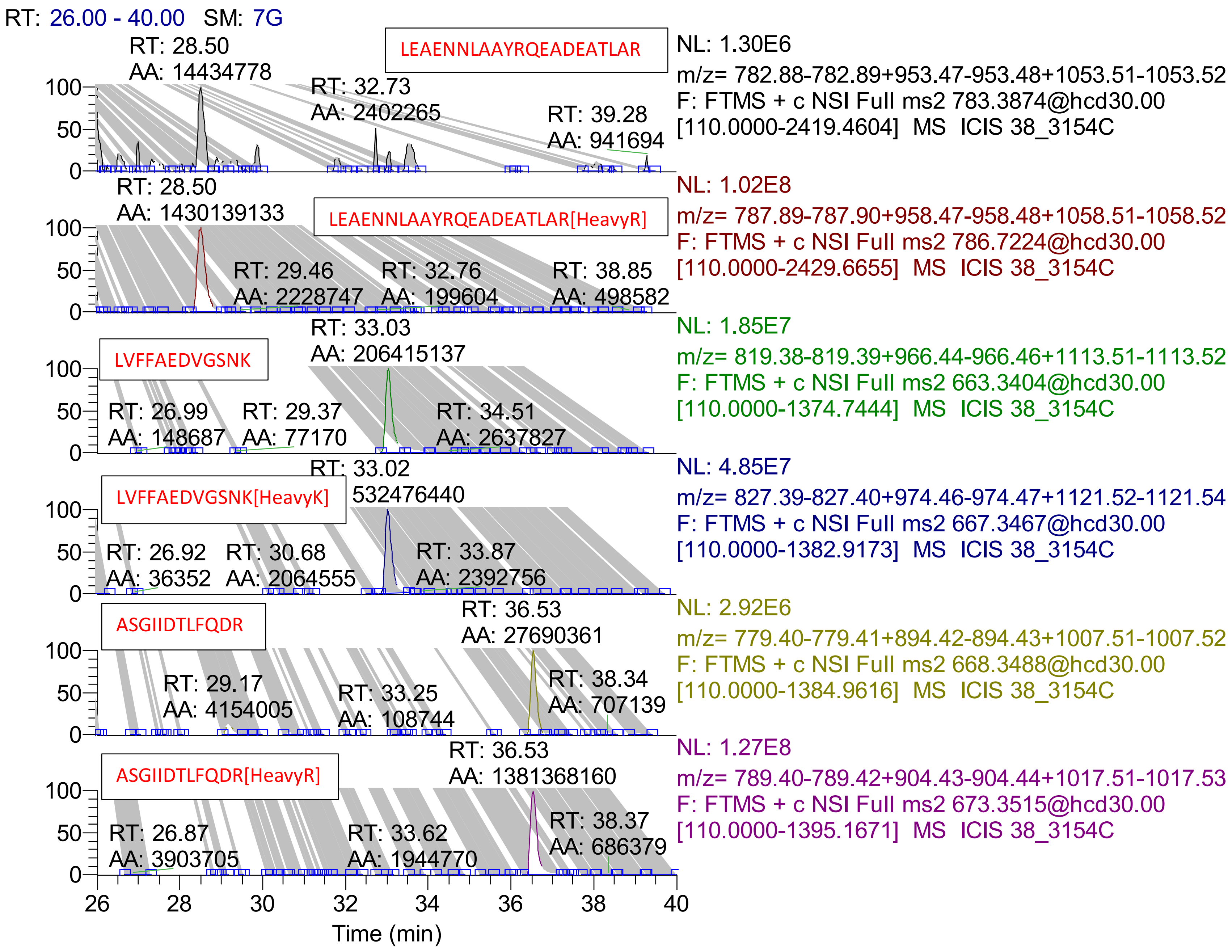
RT: 26.00 - 40.00 SM: 7G
RT: 28.50
AA: 14434778
LEAENNLAAYRQEADEATLAR
RT: 32.73
AA: 2402265
RT: 39.28
AA: 941694
NL: 1.30E6
m/z= 782.88-782.89+953.47-953.48+1053.51-1053.52
F: FTMS + c NSI Full ms2 783.3874@hcd30.00
[110.0000-2419.4604] MS ICIS 38_3154C
RT: 28.50
AA: 1430139133
LEAENNLAAYRQEADEATLAR[HeavyR]
RT: 29.46
AA: 2228747
RT: 32.76
AA: 199604
RT: 38.85
AA: 498582
NL: 1.02E8
m/z= 787.89-787.90+958.47-958.48+1058.51-1058.52
F: FTMS + c NSI Full ms2 786.7224@hcd30.00
[110.0000-2429.6655] MS ICIS 38_3154C
LVFFAEDVGSNK
RT: 33.03
AA: 206415137
RT: 26.99
AA: 148687
RT: 29.37
AA: 77170
RT: 34.51
AA: 2637827
NL: 1.85E7
m/z= 819.38-819.39+966.44-966.46+1113.51-1113.52
F: FTMS + c NSI Full ms2 663.3404@hcd30.00
[110.0000-1374.7444] MS ICIS 38_3154C
RT: 33.02
LVFFAEDVGSNK[HeavyK]
532476440
RT: 26.92
AA: 36352
RT: 30.68
AA: 2064555
RT: 33.87
AA: 2392756
NL: 4.85E7
m/z= 827.39-827.40+974.46-974.47+1121.52-1121.54
F: FTMS + c NSI Full ms2 667.3467@hcd30.00
[110.0000-1382.9173] MS ICIS 38_3154C
ASGIIDTLFQDR
RT: 36.53
AA: 27690361
RT: 29.17
AA: 4154005
RT: 33.25
AA: 108744
RT: 38.34
AA: 707139
NL: 2.92E6
m/z= 779.40-779.41+894.42-894.43+1007.51-1007.52
F: FTMS + c NSI Full ms2 668.3488@hcd30.00
[110.0000-1384.9616] MS ICIS 38_3154C
ASGIIDTLFQDR[HeavyR]
RT: 36.53
AA: 1381368160
RT: 26.87
AA: 3903705
RT: 33.62
AA: 1944770
RT: 38.37
AA: 686379
NL: 1.27E8
m/z= 789.40-789.42+904.43-904.44+1017.51-1017.53
F: FTMS + c NSI Full ms2 673.3515@hcd30.00
[110.0000-1395.1671] MS ICIS 38_3154C
100
50
0
26
28
30
32
34
36
38
40
Time (min)

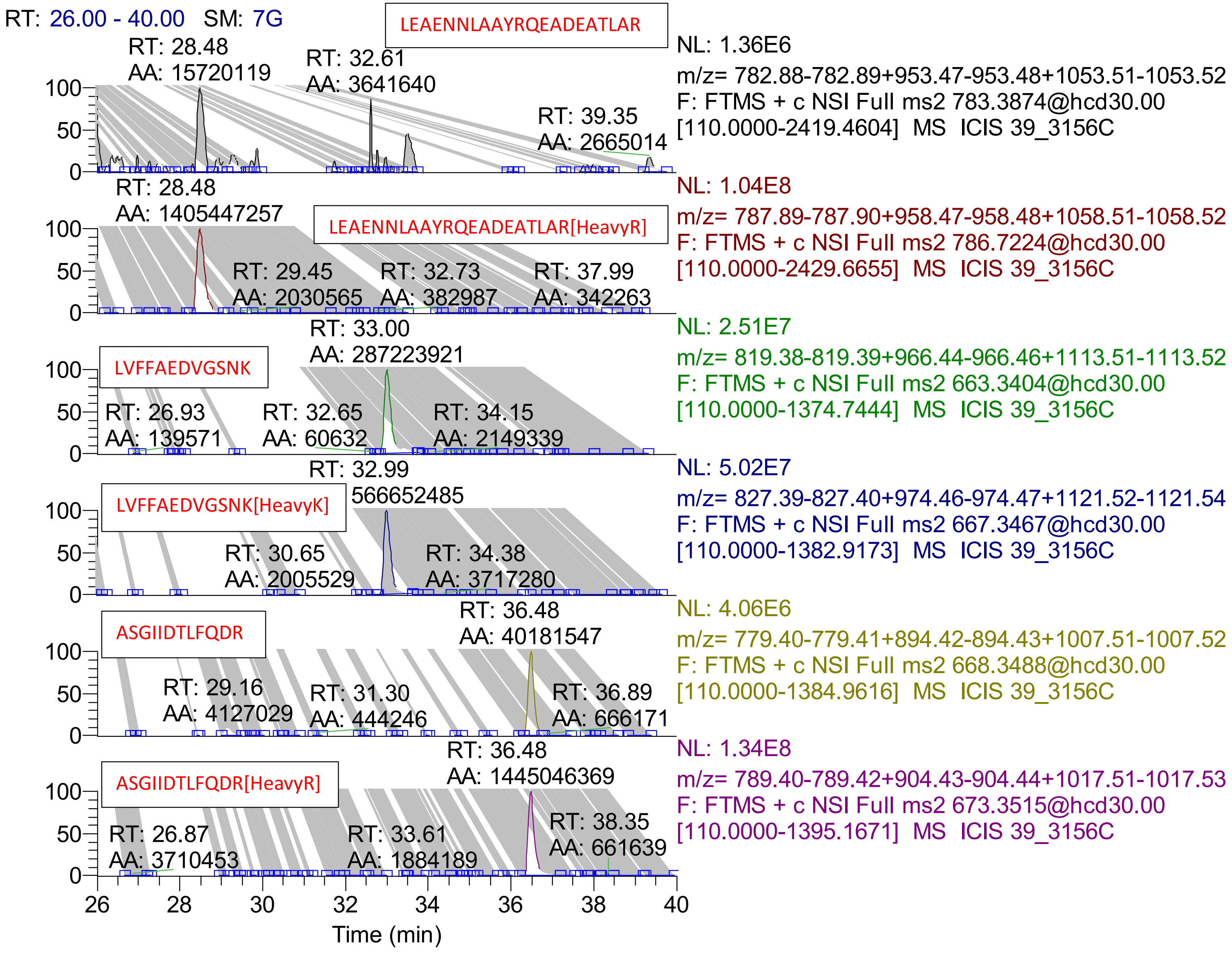

RT: 26.00 - 40.00 SM: 7G
LEAENNLAAYRQEADEATLAR
RT: 28.48 AA: 15720119
RT: 32.61 AA: 3641640
RT: 39.35 AA: 2665014
NL: 1.36E6
m/z= 782.88-782.89+953.47-953.48+1053.51-1053.52
F: FTMS + c NSI Full ms2 783.3874@hcd30.00 [110.0000-2419.4604] MS ICIS 39_3156C
LEAENNLAAYRQEADEATLAR[HeavyR]
RT: 28.48 AA: 1405447257
RT: 29.45 AA: 2030565
RT: 32.73 AA: 382987
RT: 37.99 AA: 342263
NL: 1.04E8
m/z= 787.89-787.90+958.47-958.48+1058.51-1058.52
F: FTMS + c NSI Full ms2 786.7224@hcd30.00 [110.0000-2429.6655] MS ICIS 39_3156C
LVFFAEDVGSNK
RT: 33.00 AA: 287223921
RT: 26.93 AA: 139571
RT: 32.65 AA: 60632
RT: 34.15 AA: 2149339
NL: 2.51E7
m/z= 819.38-819.39+966.44-966.46+1113.51-1113.52
F: FTMS + c NSI Full ms2 663.3404@hcd30.00 [110.0000-1374.7444] MS ICIS 39_3156C
LVFFAEDVGSNK[HeavyK]
RT: 32.99 566652485
RT: 30.65 AA: 2005529
RT: 34.38 AA: 3717280
NL: 5.02E7
m/z= 827.39-827.40+974.46-974.47+1121.52-1121.54
F: FTMS + c NSI Full ms2 667.3467@hcd30.00 [110.0000-1382.9173] MS ICIS 39_3156C
ASGIIDTLFQDR
RT: 36.48 AA: 40181547
RT: 29.16 AA: 4127029
RT: 31.30 AA: 444246
RT: 36.89 AA: 666171
NL: 4.06E6
m/z= 779.40-779.41+894.42-894.43+1007.51-1007.52
F: FTMS + c NSI Full ms2 668.3488@hcd30.00 [110.0000-1384.9616] MS ICIS 39_3156C
ASGIIDTLFQDR[HeavyR]
RT: 36.48 AA: 1445046369
RT: 26.87 AA: 3710453
RT: 33.61 AA: 1884189
RT: 38.35 AA: 661639
NL: 1.34E8
m/z= 789.40-789.42+904.43-904.44+1017.51-1017.53
F: FTMS + c NSI Full ms2 673.3515@hcd30.00 [110.0000-1395.1671] MS ICIS 39_3156C
100
50
0
26 28 30 32 34 36 38 40
Time (min)

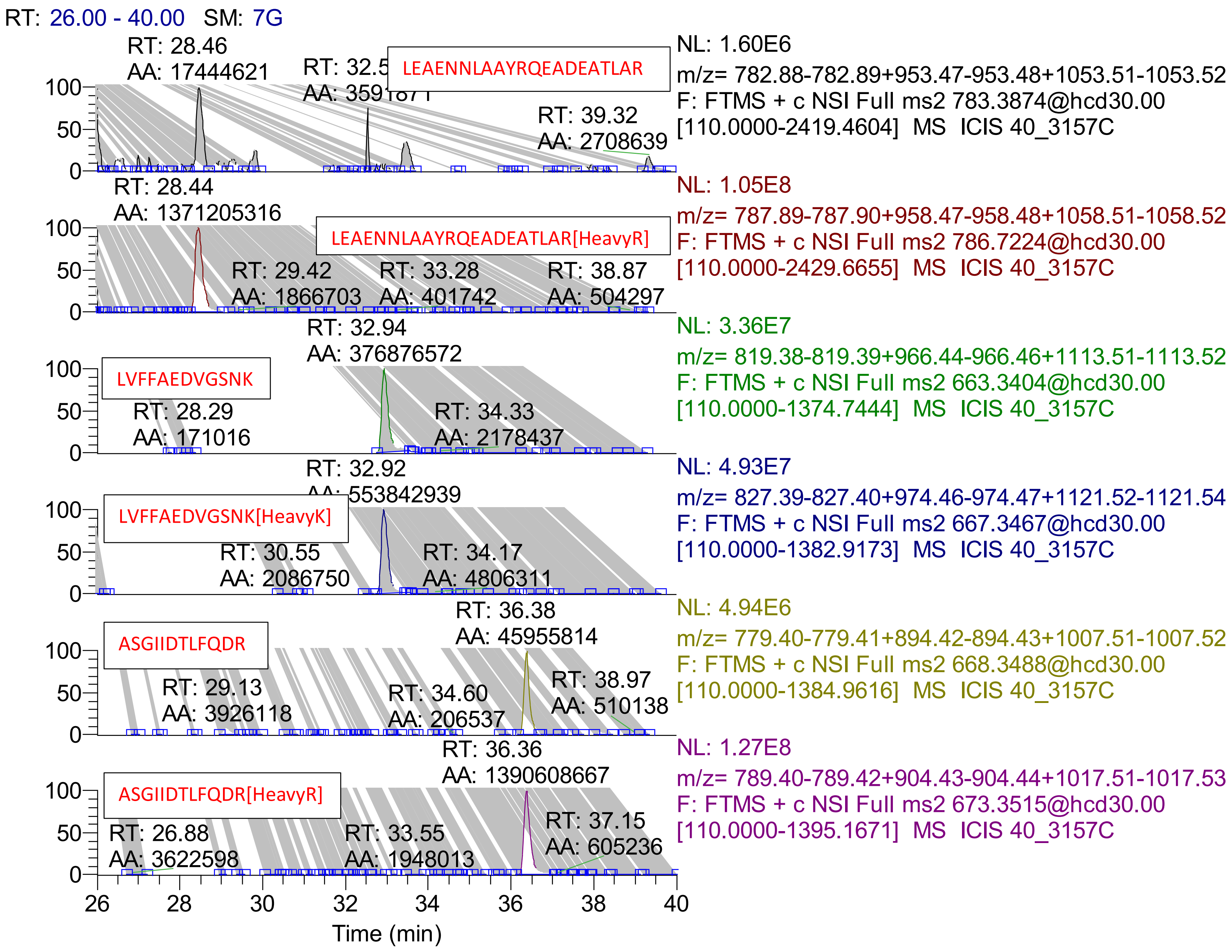
RT: 26.00 - 40.00 SM: 7G
RT: 28.46
AA: 17444621
RT: 32.5
AA: 359
LEAENNLAAYRQEADEATLAR
RT: 39.32
AA: 2708639
NL: 1.60E6
m/z= 782.88-782.89+953.47-953.48+1053.51-1053.52
F: FTMS + c NSI Full ms2 783.3874@hcd30.00
[110.0000-2419.4604] MS ICIS 40_3157C
RT: 28.44
AA: 1371205316
LEAENNLAAYRQEADEATLAR[HeavyR]
RT: 29.42
AA: 1866703
RT: 33.28
AA: 401742
RT: 38.87
AA: 504297
NL: 1.05E8
m/z= 787.89-787.90+958.47-958.48+1058.51-1058.52
F: FTMS + c NSI Full ms2 786.7224@hcd30.00
[110.0000-2429.6655] MS ICIS 40_3157C
RT: 32.94
AA: 376876572
LVFFAEDVGSNK
RT: 28.29
AA: 171016
RT: 34.33
AA: 2178437
NL: 3.36E7
m/z= 819.38-819.39+966.44-966.46+1113.51-1113.52
F: FTMS + c NSI Full ms2 663.3404@hcd30.00
[110.0000-1374.7444] MS ICIS 40_3157C
RT: 32.92
553842939
LVFFAEDVGSNK[HeavyK]
RT: 30.55
AA: 2086750
RT: 34.17
AA: 4806311
NL: 4.93E7
m/z= 827.39-827.40+974.46-974.47+1121.52-1121.54
F: FTMS + c NSI Full ms2 667.3467@hcd30.00
[110.0000-1382.9173] MS ICIS 40_3157C
RT: 36.38
AA: 45955814
ASGIIDTLFQDR
RT: 29.13
AA: 3926118
RT: 34.60
AA: 206537
RT: 38.97
AA: 510138
NL: 4.94E6
m/z= 779.40-779.41+894.42-894.43+1007.51-1007.52
F: FTMS + c NSI Full ms2 668.3488@hcd30.00
[110.0000-1384.9616] MS ICIS 40_3157C
RT: 36.36
AA: 1390608667
ASGIIDTLFQDR[HeavyR]
RT: 26.88
AA: 3622598
RT: 33.55
AA: 1948013
RT: 37.15
AA: 605236
NL: 1.27E8
m/z= 789.40-789.42+904.43-904.44+1017.51-1017.53
F: FTMS + c NSI Full ms2 673.3515@hcd30.00
[110.0000-1395.1671] MS ICIS 40_3157C
100
50
0
26
28
30
32
34
36
38
40
Time (min)

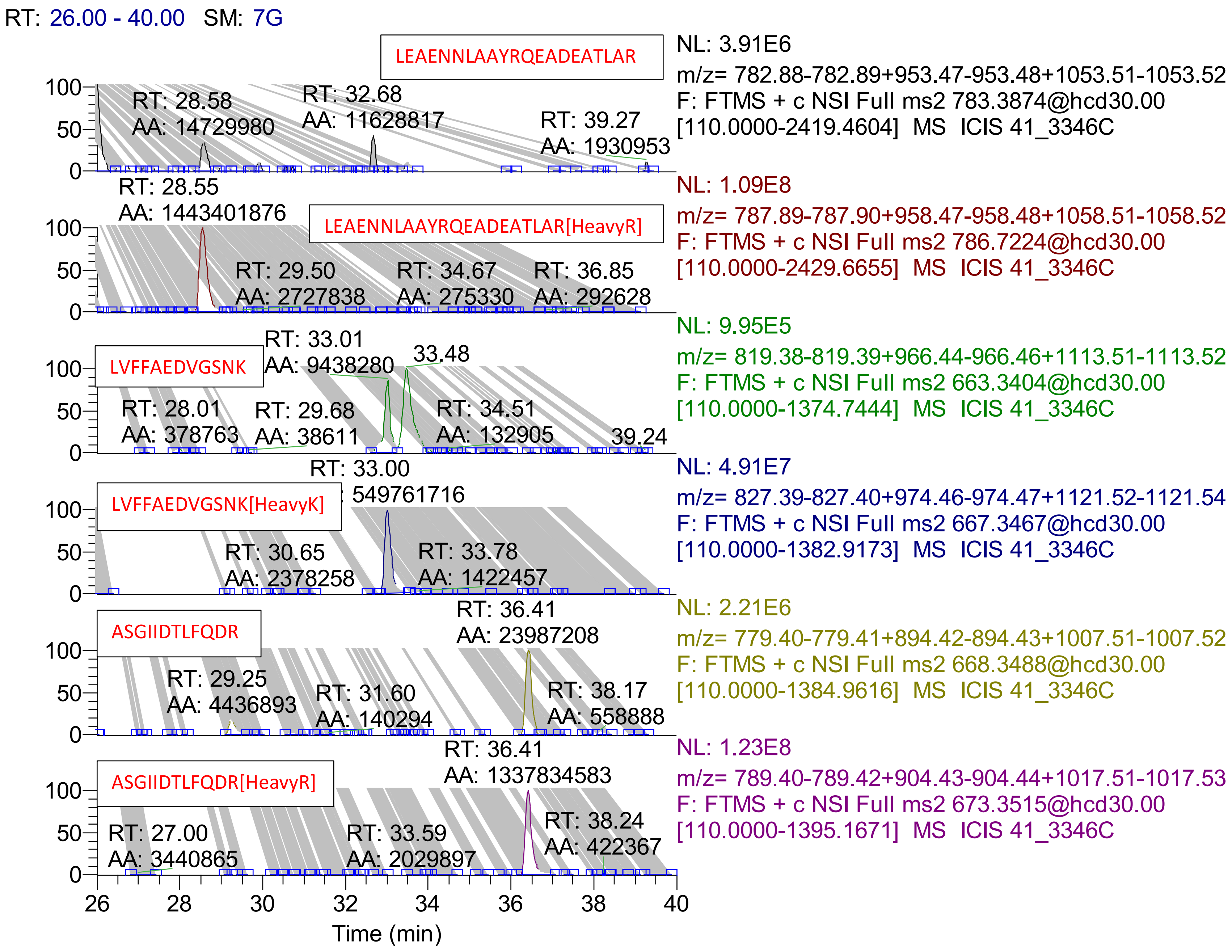

RT: 26.00 - 40.00 SM: 7G
LEAENNLAAYRQEADEATLAR
NL: 3.91E6
m/z= 782.88-782.89+953.47-953.48+1053.51-1053.52
F: FTMS + c NSI Full ms2 783.3874@hcd30.00
[110.0000-2419.4604] MS ICIS 41_3346C
RT: 28.58
AA: 14729980
RT: 32.68
AA: 11628817
RT: 39.27
AA: 1930953
RT: 28.55
AA: 1443401876
LEAENNLAAYRQEADEATLAR[HeavyR]
NL: 1.09E8
m/z= 787.89-787.90+958.47-958.48+1058.51-1058.52
F: FTMS + c NSI Full ms2 786.7224@hcd30.00
[110.0000-2429.6655] MS ICIS 41_3346C
RT: 29.50
AA: 2727838
RT: 34.67
AA: 275330
RT: 36.85
AA: 292628
LVFFAEDVGSNK
NL: 9.95E5
m/z= 819.38-819.39+966.44-966.46+1113.51-1113.52
F: FTMS + c NSI Full ms2 663.3404@hcd30.00
[110.0000-1374.7444] MS ICIS 41_3346C
RT: 33.01
AA: 9438280
33.48
RT: 28.01
AA: 378763
RT: 29.68
AA: 38611
RT: 34.51
AA: 132905
39.24
LVFFAEDVGSNK[HeavyK]
NL: 4.91E7
m/z= 827.39-827.40+974.46-974.47+1121.52-1121.54
F: FTMS + c NSI Full ms2 667.3467@hcd30.00
[110.0000-1382.9173] MS ICIS 41_3346C
RT: 33.00
549761716
RT: 30.65
AA: 2378258
RT: 33.78
AA: 1422457
ASGIIDTLFQDR
NL: 2.21E6
m/z= 779.40-779.41+894.42-894.43+1007.51-1007.52
F: FTMS + c NSI Full ms2 668.3488@hcd30.00
[110.0000-1384.9616] MS ICIS 41_3346C
RT: 36.41
AA: 23987208
RT: 29.25
AA: 4436893
RT: 31.60
AA: 140294
RT: 38.17
AA: 558888
ASGIIDTLFQDR[HeavyR]
NL: 1.23E8
m/z= 789.40-789.42+904.43-904.44+1017.51-1017.53
F: FTMS + c NSI Full ms2 673.3515@hcd30.00
[110.0000-1395.1671] MS ICIS 41_3346C
RT: 36.41
AA: 1337834583
RT: 27.00
AA: 3440865
RT: 33.59
AA: 2029897
RT: 38.24
AA: 422367
100
50
0
26
28
30
32
34
36
38
40
Time (min)

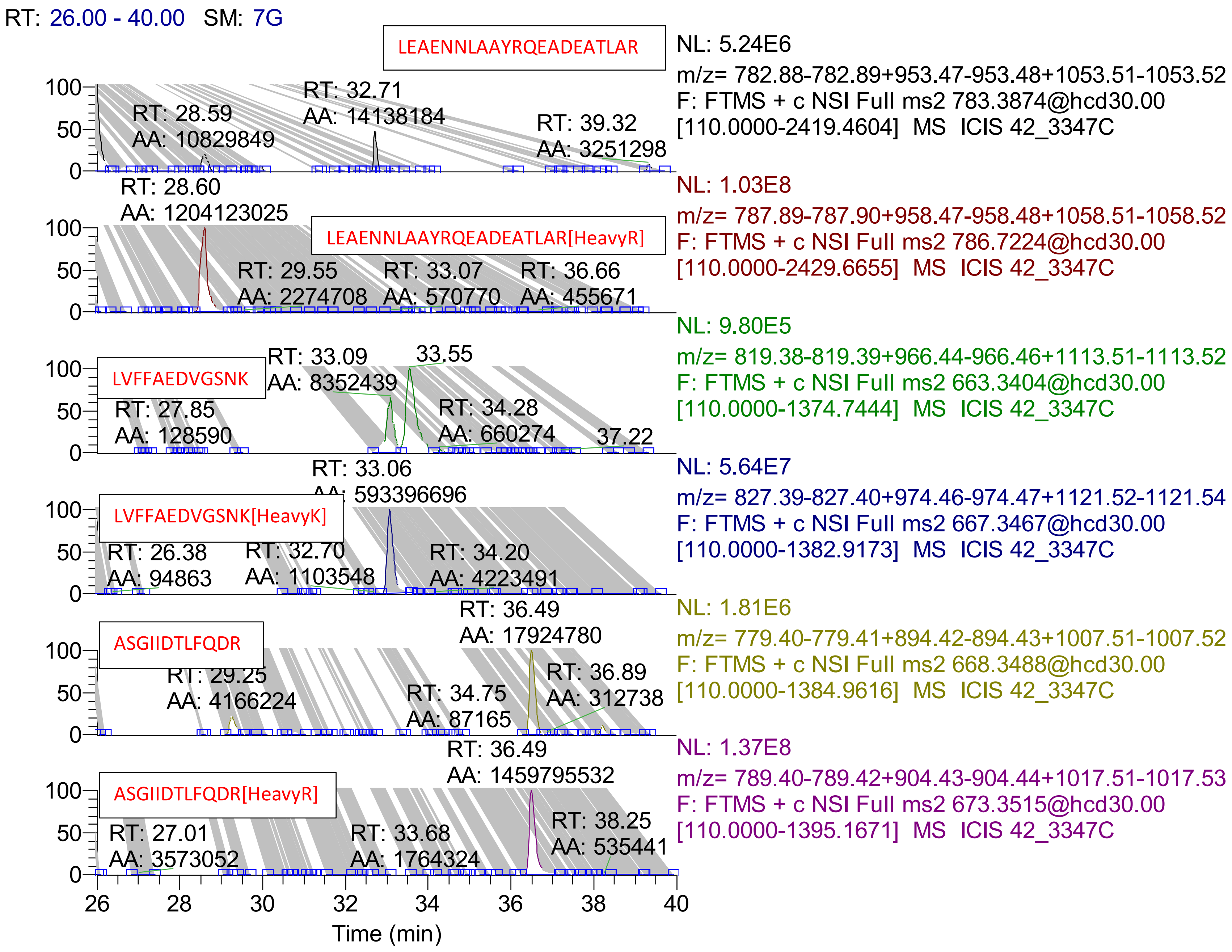
RT: 26.00 - 40.00 SM: 7G
LEAENNLAAYRQEADEATLAR
NL: 5.24E6
m/z= 782.88-782.89+953.47-953.48+1053.51-1053.52
F: FTMS + c NSI Full ms2 783.3874@hcd30.00
[110.0000-2419.4604] MS ICIS 42_3347C
RT: 28.59
AA: 10829849
RT: 32.71
AA: 14138184
RT: 39.32
AA: 3251298
RT: 28.60
AA: 1204123025
LEAENNLAAYRQEADEATLAR[HeavyR]
NL: 1.03E8
m/z= 787.89-787.90+958.47-958.48+1058.51-1058.52
F: FTMS + c NSI Full ms2 786.7224@hcd30.00
[110.0000-2429.6655] MS ICIS 42_3347C
RT: 29.55
AA: 2274708
RT: 33.07
AA: 570770
RT: 36.66
AA: 455671
LVFFAEDVGSNK
NL: 9.80E5
m/z= 819.38-819.39+966.44-966.46+1113.51-1113.52
F: FTMS + c NSI Full ms2 663.3404@hcd30.00
[110.0000-1374.7444] MS ICIS 42_3347C
RT: 33.09
AA: 8352439
33.55
RT: 27.85
AA: 128590
RT: 34.28
AA: 660274
37.22
RT: 33.06
AA: 593396696
LVFFAEDVGSNK[HeavyK]
NL: 5.64E7
m/z= 827.39-827.40+974.46-974.47+1121.52-1121.54
F: FTMS + c NSI Full ms2 667.3467@hcd30.00
[110.0000-1382.9173] MS ICIS 42_3347C
RT: 26.38
AA: 94863
RT: 32.70
AA: 1103548
RT: 34.20
AA: 4223491
RT: 36.49
AA: 17924780
ASGIIDTLFQDR
NL: 1.81E6
m/z= 779.40-779.41+894.42-894.43+1007.51-1007.52
F: FTMS + c NSI Full ms2 668.3488@hcd30.00
[110.0000-1384.9616] MS ICIS 42_3347C
RT: 29.25
AA: 4166224
RT: 34.75
AA: 87165
RT: 36.89
AA: 312738
RT: 36.49
AA: 1459795532
ASGIIDTLFQDR[HeavyR]
NL: 1.37E8
m/z= 789.40-789.42+904.43-904.44+1017.51-1017.53
F: FTMS + c NSI Full ms2 673.3515@hcd30.00
[110.0000-1395.1671] MS ICIS 42_3347C
RT: 27.01
AA: 3573052
RT: 33.68
AA: 1764324
RT: 38.25
AA: 535441
100
50
0
26
28
30
32
34
36
38
40
Time (min)

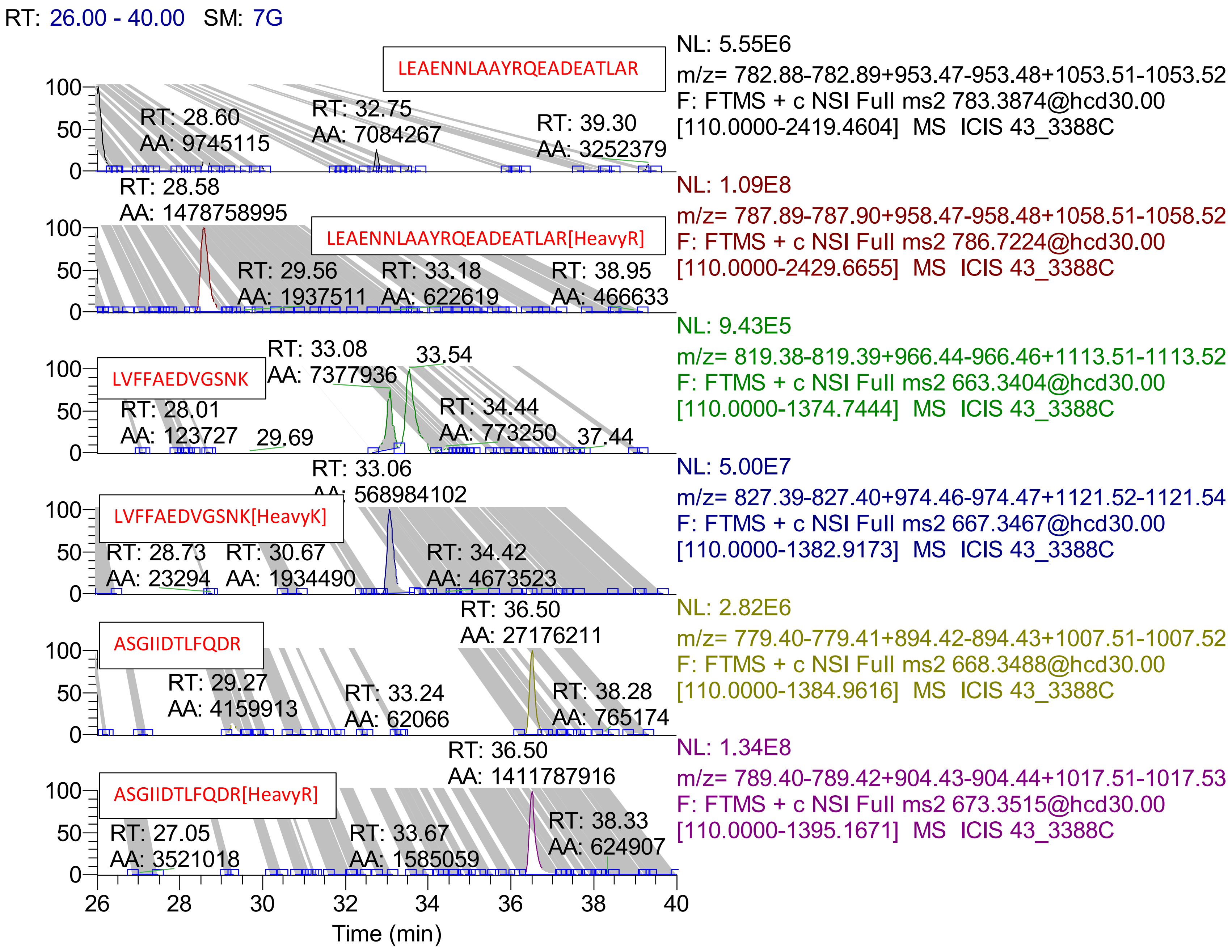
RT: 26.00 - 40.00 SM: 7G
LEAENNLAAYRQEADEATLAR
RT: 28.60
AA: 9745115
RT: 32.75
AA: 7084267
RT: 39.30
AA: 3252379
NL: 5.55E6
m/z= 782.88-782.89+953.47-953.48+1053.51-1053.52
F: FTMS + c NSI Full ms2 783.3874@hcd30.00
[110.0000-2419.4604] MS ICIS 43_3388C
RT: 28.58
AA: 1478758995
LEAENNLAAYRQEADEATLAR[HeavyR]
RT: 29.56
AA: 1937511
RT: 33.18
AA: 622619
RT: 38.95
AA: 466633
NL: 1.09E8
m/z= 787.89-787.90+958.47-958.48+1058.51-1058.52
F: FTMS + c NSI Full ms2 786.7224@hcd30.00
[110.0000-2429.6655] MS ICIS 43_3388C
LVFFAEDVGSNK
RT: 33.08
AA: 7377936
33.54
RT: 28.01
AA: 123727
29.69
RT: 34.44
AA: 773250
37.44
NL: 9.43E5
m/z= 819.38-819.39+966.44-966.46+1113.51-1113.52
F: FTMS + c NSI Full ms2 663.3404@hcd30.00
[110.0000-1374.7444] MS ICIS 43_3388C
RT: 33.06
AA: 568984102
LVFFAEDVGSNK[HeavyK]
RT: 28.73
AA: 23294
RT: 30.67
AA: 1934490
RT: 34.42
AA: 4673523
NL: 5.00E7
m/z= 827.39-827.40+974.46-974.47+1121.52-1121.54
F: FTMS + c NSI Full ms2 667.3467@hcd30.00
[110.0000-1382.9173] MS ICIS 43_3388C
ASGIIDTLFQDR
RT: 36.50
AA: 27176211
RT: 29.27
AA: 4159913
RT: 33.24
AA: 62066
RT: 38.28
AA: 765174
NL: 2.82E6
m/z= 779.40-779.41+894.42-894.43+1007.51-1007.52
F: FTMS + c NSI Full ms2 668.3488@hcd30.00
[110.0000-1384.9616] MS ICIS 43_3388C
ASGIIDTLFQDR[HeavyR]
RT: 36.50
AA: 1411787916
RT: 27.05
AA: 3521018
RT: 33.67
AA: 1585059
RT: 38.33
AA: 624907
NL: 1.34E8
m/z= 789.40-789.42+904.43-904.44+1017.51-1017.53
F: FTMS + c NSI Full ms2 673.3515@hcd30.00
[110.0000-1395.1671] MS ICIS 43_3388C
100
50
0
26
28
30
32
34
36
38
40
Time (min)

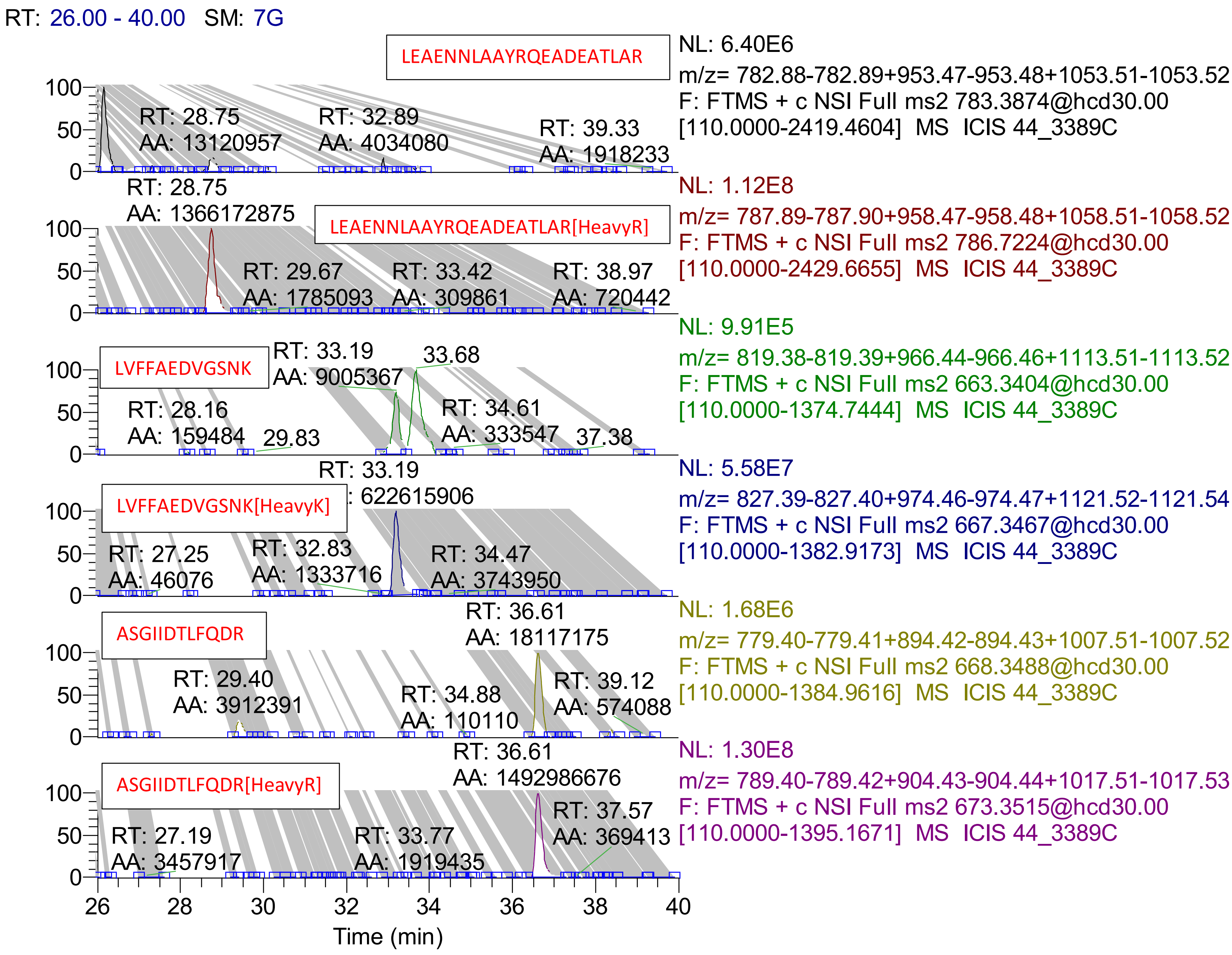
RT: 26.00 - 40.00 SM: 7G
LEAENNLAAYRQEADEATLAR
NL: 6.40E6
m/z= 782.88-782.89+953.47-953.48+1053.51-1053.52
F: FTMS + c NSI Full ms2 783.3874@hcd30.00
[110.0000-2419.4604] MS ICIS 44_3389C
RT: 28.75
AA: 13120957
RT: 32.89
AA: 4034080
RT: 39.33
AA: 1918233
RT: 28.75
AA: 1366172875
LEAENNLAAYRQEADEATLAR[HeavyR]
NL: 1.12E8
m/z= 787.89-787.90+958.47-958.48+1058.51-1058.52
F: FTMS + c NSI Full ms2 786.7224@hcd30.00
[110.0000-2429.6655] MS ICIS 44_3389C
RT: 29.67
AA: 1785093
RT: 33.42
AA: 309861
RT: 38.97
AA: 720442
LVFFAEDVGSNK
NL: 9.91E5
m/z= 819.38-819.39+966.44-966.46+1113.51-1113.52
F: FTMS + c NSI Full ms2 663.3404@hcd30.00
[110.0000-1374.7444] MS ICIS 44_3389C
RT: 33.19
AA: 9005367
33.68
RT: 28.16
AA: 159484
29.83
RT: 34.61
AA: 333547
37.38
LVFFAEDVGSNK[HeavyK]
NL: 5.58E7
m/z= 827.39-827.40+974.46-974.47+1121.52-1121.54
F: FTMS + c NSI Full ms2 667.3467@hcd30.00
[110.0000-1382.9173] MS ICIS 44_3389C
RT: 33.19
: 622615906
RT: 27.25
AA: 46076
RT: 32.83
AA: 1333716
RT: 34.47
AA: 3743950
ASGIIDTLFQDR
NL: 1.68E6
m/z= 779.40-779.41+894.42-894.43+1007.51-1007.52
F: FTMS + c NSI Full ms2 668.3488@hcd30.00
[110.0000-1384.9616] MS ICIS 44_3389C
RT: 36.61
AA: 18117175
RT: 29.40
AA: 3912391
RT: 34.88
AA: 110110
RT: 39.12
AA: 574088
ASGIIDTLFQDR[HeavyR]
NL: 1.30E8
m/z= 789.40-789.42+904.43-904.44+1017.51-1017.53
F: FTMS + c NSI Full ms2 673.3515@hcd30.00
[110.0000-1395.1671] MS ICIS 44_3389C
RT: 36.61
AA: 1492986676
RT: 37.57
AA: 369413
RT: 27.19
AA: 3457917
RT: 33.77
AA: 1919435
100
50
0
26
28
30
32
34
36
38
40
Time (min)

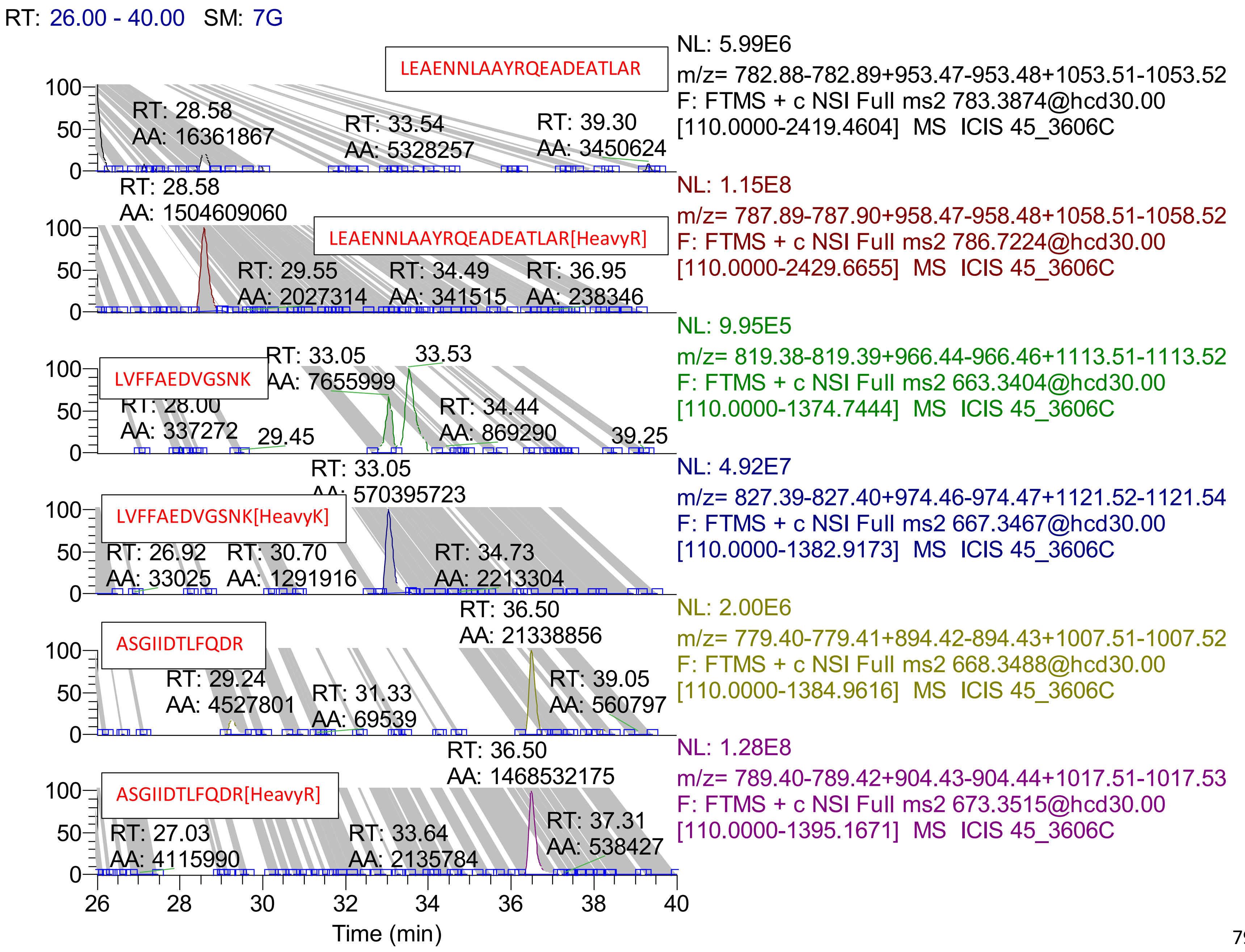

RT: 26.00 - 40.00 SM: 7G
LEAENNLAAYRQEADEATLAR
RT: 28.58
AA: 16361867
RT: 33.54
AA: 5328257
RT: 39.30
AA: 3450624
NL: 5.99E6
m/z= 782.88-782.89+953.47-953.48+1053.51-1053.52
F: FTMS + c NSI Full ms2 783.3874@hcd30.00
[110.0000-2419.4604] MS ICIS 45_3606C
RT: 28.58
AA: 1504609060
LEAENNLAAYRQEADEATLAR[HeavyR]
RT: 29.55
AA: 2027314
RT: 34.49
AA: 341515
RT: 36.95
AA: 238346
NL: 1.15E8
m/z= 787.89-787.90+958.47-958.48+1058.51-1058.52
F: FTMS + c NSI Full ms2 786.7224@hcd30.00
[110.0000-2429.6655] MS ICIS 45_3606C
LVFFAEDVGSNK
RT: 33.05
AA: 7655999
33.53
RT: 28.00
AA: 337272
29.45
RT: 34.44
AA: 869290
39.25
NL: 9.95E5
m/z= 819.38-819.39+966.44-966.46+1113.51-1113.52
F: FTMS + c NSI Full ms2 663.3404@hcd30.00
[110.0000-1374.7444] MS ICIS 45_3606C
RT: 33.05
AA: 570395723
LVFFAEDVGSNK[HeavyK]
RT: 26.92
AA: 33025
RT: 30.70
AA: 1291916
RT: 34.73
AA: 2213304
NL: 4.92E7
m/z= 827.39-827.40+974.46-974.47+1121.52-1121.54
F: FTMS + c NSI Full ms2 667.3467@hcd30.00
[110.0000-1382.9173] MS ICIS 45_3606C
RT: 36.50
AA: 21338856
ASGIIDTLFQDR
RT: 29.24
AA: 4527801
RT: 31.33
AA: 69539
RT: 39.05
AA: 560797
NL: 2.00E6
m/z= 779.40-779.41+894.42-894.43+1007.51-1007.52
F: FTMS + c NSI Full ms2 668.3488@hcd30.00
[110.0000-1384.9616] MS ICIS 45_3606C
RT: 36.50
AA: 1468532175
ASGIIDTLFQDR[HeavyR]
RT: 27.03
AA: 4115990
RT: 33.64
AA: 2135784
RT: 37.31
AA: 538427
NL: 1.28E8
m/z= 789.40-789.42+904.43-904.44+1017.51-1017.53
F: FTMS + c NSI Full ms2 673.3515@hcd30.00
[110.0000-1395.1671] MS ICIS 45_3606C
100
50
0
26
28
30
32
34
36
38
40
Time (min)

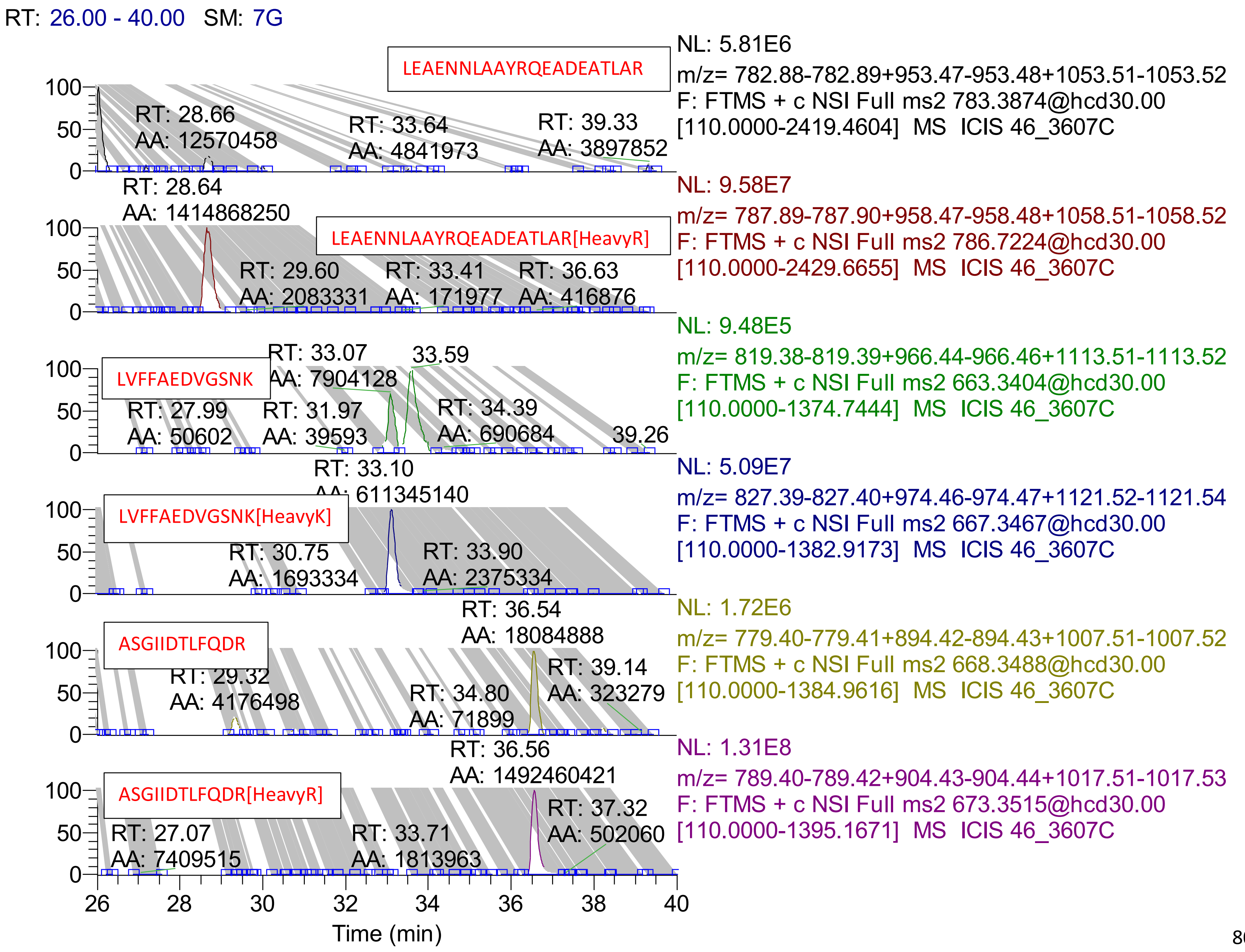
RT: 26.00 - 40.00 SM: 7G
LEAENNLAAYRQEADEATLAR
NL: 5.81E6
m/z= 782.88-782.89+953.47-953.48+1053.51-1053.52
F: FTMS + c NSI Full ms2 783.3874@hcd30.00
[110.0000-2419.4604] MS ICIS 46_3607C
RT: 28.66 AA: 12570458
RT: 33.64 AA: 4841973
RT: 39.33 AA: 3897852
LEAENNLAAYRQEADEATLAR[HeavyR]
NL: 9.58E7
m/z= 787.89-787.90+958.47-958.48+1058.51-1058.52
F: FTMS + c NSI Full ms2 786.7224@hcd30.00
[110.0000-2429.6655] MS ICIS 46_3607C
RT: 28.64 AA: 1414868250
RT: 29.60 AA: 2083331
RT: 33.41 AA: 171977
RT: 36.63 AA: 416876
LVFFAEDVGSNK
NL: 9.48E5
m/z= 819.38-819.39+966.44-966.46+1113.51-1113.52
F: FTMS + c NSI Full ms2 663.3404@hcd30.00
[110.0000-1374.7444] MS ICIS 46_3607C
RT: 33.07 AA: 7904128
33.59
RT: 27.99 AA: 50602
RT: 31.97 AA: 39593
RT: 34.39 AA: 690684
39.26
LVFFAEDVGSNK[HeavyK]
NL: 5.09E7
m/z= 827.39-827.40+974.46-974.47+1121.52-1121.54
F: FTMS + c NSI Full ms2 667.3467@hcd30.00
[110.0000-1382.9173] MS ICIS 46_3607C
RT: 33.10 AA: 611345140
RT: 30.75 AA: 1693334
RT: 33.90 AA: 2375334
ASGIIDTLFQDR
NL: 1.72E6
m/z= 779.40-779.41+894.42-894.43+1007.51-1007.52
F: FTMS + c NSI Full ms2 668.3488@hcd30.00
[110.0000-1384.9616] MS ICIS 46_3607C
RT: 36.54 AA: 18084888
RT: 29.32 AA: 4176498
RT: 34.80 AA: 71899
RT: 39.14 AA: 323279
ASGIIDTLFQDR[HeavyR]
NL: 1.31E8
m/z= 789.40-789.42+904.43-904.44+1017.51-1017.53
F: FTMS + c NSI Full ms2 673.3515@hcd30.00
[110.0000-1395.1671] MS ICIS 46_3607C
RT: 36.56 AA: 1492460421
RT: 27.07 AA: 7409515
RT: 33.71 AA: 1813963
RT: 37.32 AA: 502060
100
50
0
26 28 30 32 34 36 38 40
Time (min)

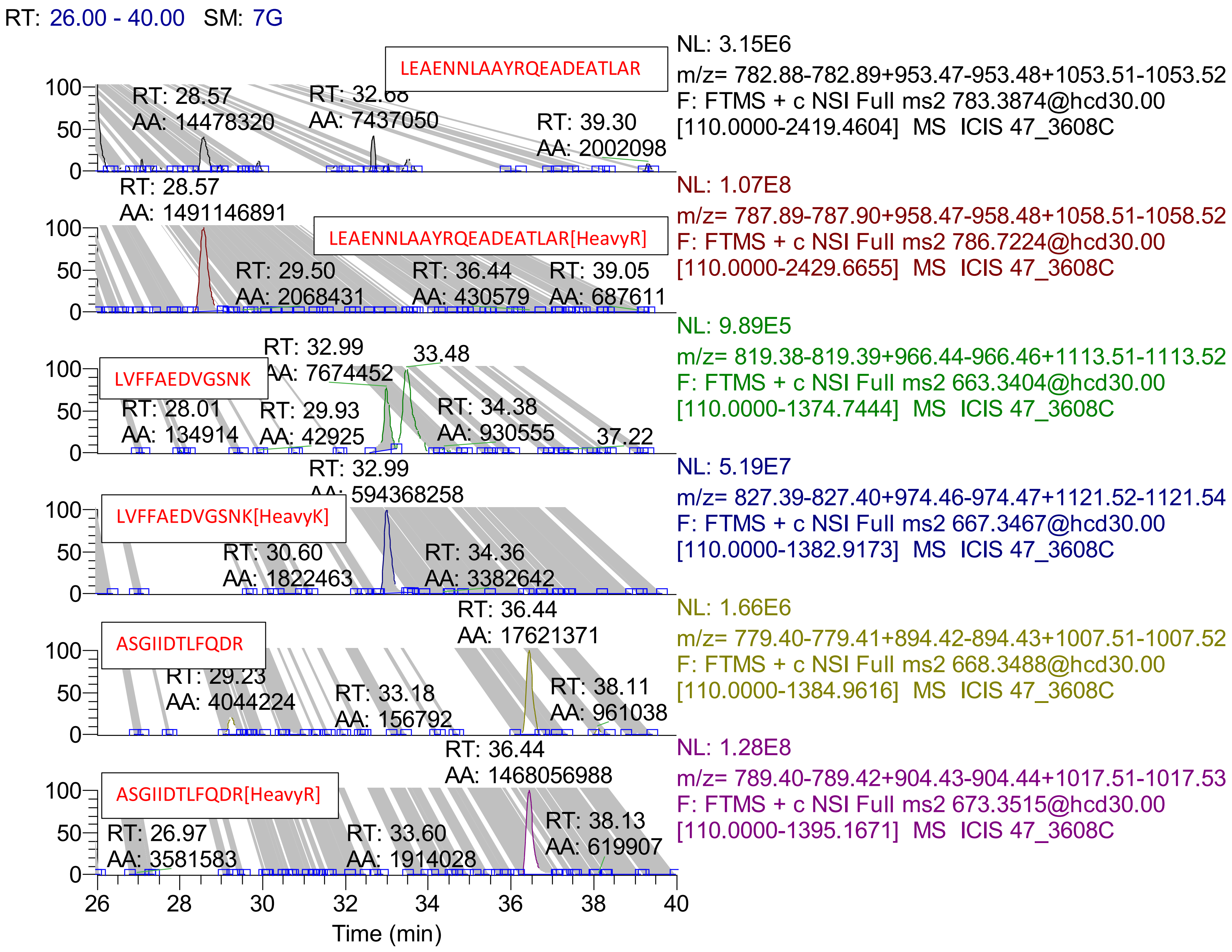
RT: 26.00 - 40.00 SM: 7G
LEAENNLAAYRQEADEATLAR
RT: 28.57
AA: 14478320
AA: 7437050
RT: 39.30
AA: 2002098
NL: 3.15E6
m/z= 782.88-782.89+953.47-953.48+1053.51-1053.52
F: FTMS + c NSI Full ms2 783.3874@hcd30.00
[110.0000-2419.4604] MS ICIS 47_3608C
RT: 28.57
AA: 1491146891
LEAENNLAAYRQEADEATLAR[HeavyR]
RT: 29.50
AA: 2068431
RT: 36.44
AA: 430579
RT: 39.05
AA: 687611
NL: 1.07E8
m/z= 787.89-787.90+958.47-958.48+1058.51-1058.52
F: FTMS + c NSI Full ms2 786.7224@hcd30.00
[110.0000-2429.6655] MS ICIS 47_3608C
LVFFAEDVGSNK
RT: 32.99
33.48
RT: 28.01
AA: 134914
RT: 29.93
AA: 42925
RT: 34.38
AA: 930555
37.22
NL: 9.89E5
m/z= 819.38-819.39+966.44-966.46+1113.51-1113.52
F: FTMS + c NSI Full ms2 663.3404@hcd30.00
[110.0000-1374.7444] MS ICIS 47_3608C
RT: 32.99
594368258
LVFFAEDVGSNK[HeavyK]
RT: 30.60
AA: 1822463
RT: 34.36
AA: 3382642
NL: 5.19E7
m/z= 827.39-827.40+974.46-974.47+1121.52-1121.54
F: FTMS + c NSI Full ms2 667.3467@hcd30.00
[110.0000-1382.9173] MS ICIS 47_3608C
ASGIIDTLFQDR
RT: 36.44
AA: 17621371
AA: 4044224
RT: 33.18
AA: 156792
RT: 38.11
AA: 961038
NL: 1.66E6
m/z= 779.40-779.41+894.42-894.43+1007.51-1007.52
F: FTMS + c NSI Full ms2 668.3488@hcd30.00
[110.0000-1384.9616] MS ICIS 47_3608C
ASGIIDTLFQDR[HeavyR]
RT: 36.44
AA: 1468056988
RT: 26.97
AA: 3581583
RT: 33.60
AA: 1914028
RT: 38.13
AA: 619907
NL: 1.28E8
m/z= 789.40-789.42+904.43-904.44+1017.51-1017.53
F: FTMS + c NSI Full ms2 673.3515@hcd30.00
[110.0000-1395.1671] MS ICIS 47_3608C
100
50
0
26
28
30
32
34
36
38
40
Time (min)

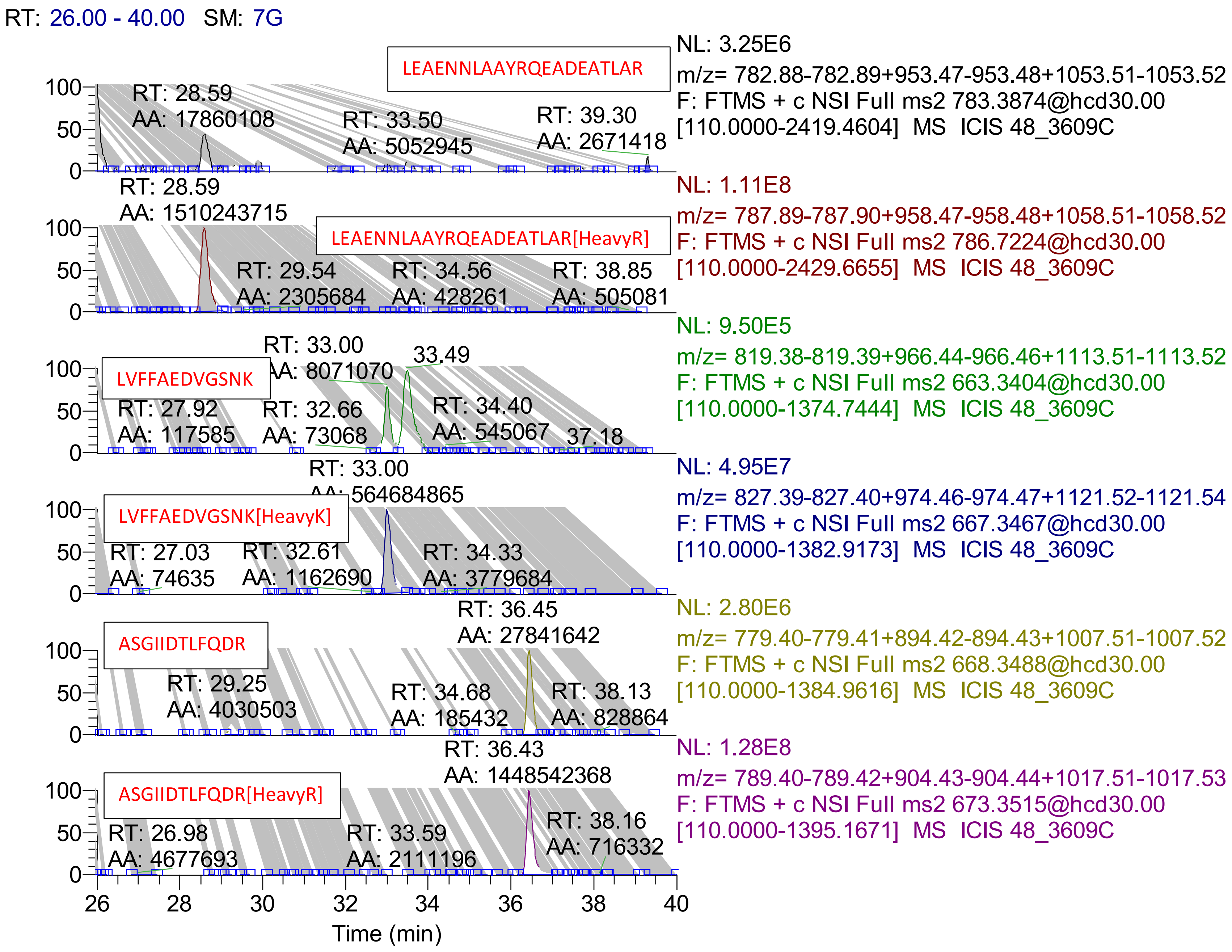

RT: 26.00 - 40.00 SM: 7G
LEAENNLAAYRQEADEATLAR
NL: 3.25E6
m/z= 782.88-782.89+953.47-953.48+1053.51-1053.52
F: FTMS + c NSI Full ms2 783.3874@hcd30.00
[110.0000-2419.4604] MS ICIS 48_3609C
RT: 28.59 AA: 17860108
RT: 33.50 AA: 5052945
RT: 39.30 AA: 2671418
LEAENNLAAYRQEADEATLAR[HeavyR]
NL: 1.11E8
m/z= 787.89-787.90+958.47-958.48+1058.51-1058.52
F: FTMS + c NSI Full ms2 786.7224@hcd30.00
[110.0000-2429.6655] MS ICIS 48_3609C
RT: 28.59 AA: 1510243715
RT: 29.54 AA: 2305684
RT: 34.56 AA: 428261
RT: 38.85 AA: 505081
LVFFAEDVGSNK
NL: 9.50E5
m/z= 819.38-819.39+966.44-966.46+1113.51-1113.52
F: FTMS + c NSI Full ms2 663.3404@hcd30.00
[110.0000-1374.7444] MS ICIS 48_3609C
RT: 33.00 AA: 8071070
33.49
RT: 27.92 AA: 117585
RT: 32.66 AA: 73068
RT: 34.40 AA: 545067
37.18
LVFFAEDVGSNK[HeavyK]
NL: 4.95E7
m/z= 827.39-827.40+974.46-974.47+1121.52-1121.54
F: FTMS + c NSI Full ms2 667.3467@hcd30.00
[110.0000-1382.9173] MS ICIS 48_3609C
RT: 33.00 AA: 564684865
RT: 27.03 AA: 74635
RT: 32.61 AA: 1162690
RT: 34.33 AA: 3779684
ASGIIDTLFQDR
NL: 2.80E6
m/z= 779.40-779.41+894.42-894.43+1007.51-1007.52
F: FTMS + c NSI Full ms2 668.3488@hcd30.00
[110.0000-1384.9616] MS ICIS 48_3609C
RT: 36.45 AA: 27841642
RT: 29.25 AA: 4030503
RT: 34.68 AA: 185432
RT: 38.13 AA: 828864
ASGIIDTLFQDR[HeavyR]
NL: 1.28E8
m/z= 789.40-789.42+904.43-904.44+1017.51-1017.53
F: FTMS + c NSI Full ms2 673.3515@hcd30.00
[110.0000-1395.1671] MS ICIS 48_3609C
RT: 36.43 AA: 1448542368
RT: 26.98 AA: 4677693
RT: 33.59 AA: 2111196
RT: 38.16 AA: 716332
100
50
0
26
28
30
32
34
36
38
40
Time (min)
